# Stereoselective nucleophilic addition reactions to cyclic *N*-acyliminium ions using the indirect cation pool method: Elucidation of stereoselectivity by spectroscopic conformational analysis and DFT calculations

**DOI:** 10.3762/bjoc.14.100

**Published:** 2018-05-24

**Authors:** Koichi Mitsudo, Junya Yamamoto, Tomoya Akagi, Atsuhiro Yamashita, Masahiro Haisa, Kazuki Yoshioka, Hiroki Mandai, Koji Ueoka, Christian Hempel, Jun-ichi Yoshida, Seiji Suga

**Affiliations:** 1 Division of Applied Chemistry, Graduate School of Natural Science and Technology, Okayama University, 3-1-1 Tsushima-naka, Kita-ku, Okayama 700-8530, Japan; 2 Department of Synthetic Chemistry and Biological Chemistry, Graduate School of Engineering, Kyoto University, Nishikyo-ku, Kyoto 615-8510, Japan

**Keywords:** cation pool, conformation, electroorganic synthesis, *N*-acyliminium ion, NMR analysis, piperidine

## Abstract

In this study, six-membered *N*-acyliminium ions were generated by the “indirect cation pool” method and reacted with several nucleophiles. These reactions afforded disubstituted piperidine derivatives with high diastereoselectivities and good to excellent yields. The conformations of the obtained *N*-acyliminium ions were studied by low temperature NMR analyses and DFT calculations and were found to be consistent with the Steven’s hypothesis.

## Introduction

**Scheme 1:**
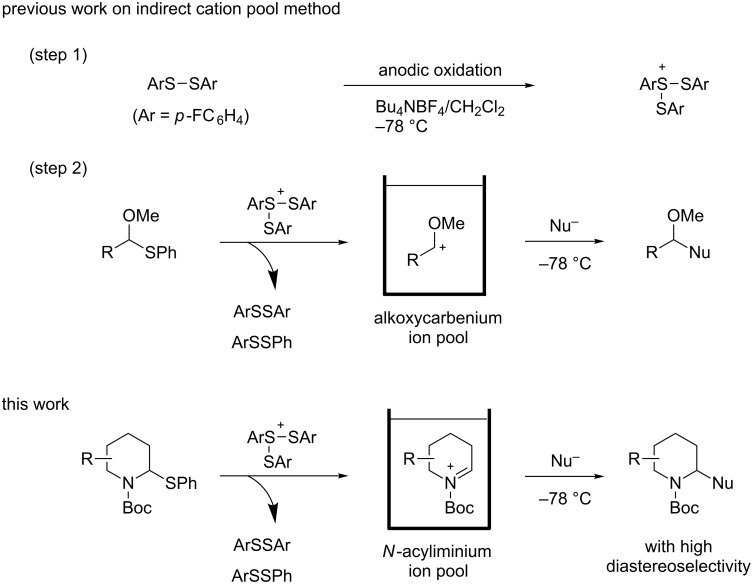
Generation and reaction of cationic species generated by “indirect cation pool” methods.

Cyclic amines are significant key motifs in pharmaceutical and natural products because a variety of compounds bearing those moieties exhibit physiological and pharmacological activities [1–3]. As many of these compounds feature asymmetric carbon atoms at the α-position of the cyclic amine, the stereoselective carbon–carbon bond formation at this position is of great importance from the perspective of drug discovery [4–5]. Meanwhile the “cation pool” method can realize the generation and accumulation of highly reactive cationic species such as *N*-acyliminium ions in relatively high concentrations by low temperature electrolysis [6–10]. The *N*-acyliminium ions thus generated can directly react with a variety of carbon nucleophiles to very efficiently yield carbon–carbon bond-formation products. In addition, this research lead to the development of an indirect cation pool method that enables the creation of the cation pool by reacting a cation precursor having a C–S bond with an electrochemically generated ArS(ArSSAr)^+^ as the cation-generating reagent. For example, a pool of alkoxycarbenium ions can be rapidly generated by the reaction of thioacetals and ArS(ArSSAr)^+^ (Ar = *p*-FC_6_H_4_, Scheme 1) [11].

The cation pool method is advantageous over the conventional Lewis acid promoted generation of carbocation species because the reactive cationic species can be detected by spectroscopy such as NMR and IR measurements. For this research, the reaction of six-membered *N*-acyliminium ions having a substituent in the 4-, 5-, or 6-position were generated by the indirect cation pool method and examined in the reaction with several nucleophiles to give the disubstituted piperidine derivatives in an excellent diastereoselective manner (Scheme 1). This result inspired us to investigate the stereochemistry of the *N*-acyliminium ions by means of NMR spectroscopy, which is challenging to achieve without using cation pool methods. The current report presents the diastereoselective synthesis of disubstituted piperidine derivatives by the indirect cation pool method and the elucidation of the stereoselectivity based on both NMR analyses of the cyclic *N*-acyliminium ions and DFT calculations.

## Results and Discussion

*N*-Acyliminium ions obtained from *N*-Boc-piperidines are attractive intermediates because they easily can be transformed to a variety of alkaloids having a piperidine skeleton. In our preliminary study, we found the indirect cation pool method suitable for the generation and accumulation of *N*-acyliminium ions. Therefore, the reaction of *N*-Boc-4-phenyl-2,3,4,5-tetrahydropyridin-1-ium (**C1**) derived from precursor **1a** and ArS(ArSSAr)^+^ (Ar = *p*-FC_6_H_4_) generated by the low temperature electrolysis was first performed with several nucleophiles (Table 1). The starting **1a** and other *N*-acyliminium ion precursors were synthesized by the modified Beak’s protocol [12] (see Supporting Information File 1). As observed in our previous studies, the Boc protecting group was found not suitable for the direct cation pool method due to its cleavage by protic acid generated during the electrolysis of the cation precursor. However, it can be utilized in the indirect cation pool method as diaryldisulfides are the exclusive side products formed during carbocation generation. Thus, the reaction of acyliminium ion **C1** with Me_3_Al afforded *trans*-1-(*tert*-butoxycarbonyl)-2-methyl-4-phenylpiperidine (**2aa**) in a good yield and virtually complete diastereoselectivity (*cis*/*trans* = <1:99). Similarly, the reaction of **C1** with diethylzinc or allyltributylstannane gave piperidine derivatives **2ab** and **2ac** with high yields and *trans*-selectivity (Table 1, entries 2 and 3). Also, the introduction of phenyl or cyano groups in the piperidine core proceeded in a highly regioselective manner and good to high yields (Table 1, entries 4 and 5).

**Table 1:** Reaction of *N-*acyliminium ion **C1** with several nucleophiles.

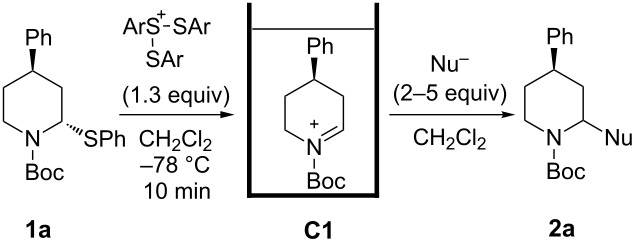				

entry	Nu^−^	**2**	yield (%)^a^	*cis*/*trans*^b^

1	Me_3_Al (5.0 equiv)	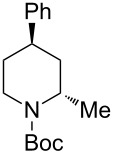 **2aa**	85	<1:99
2	Et_2_Zn (2.5 equiv)	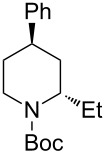 **2ab**	89	3:97
3	(allyl)SnBu_3_ (3.0 equiv)	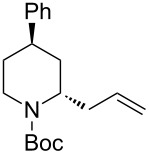 **2ac**	>99	<1:99
4	PhMgBr (2.0 equiv)	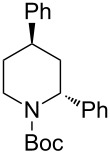 **2ad**	87	15:85
5	TMSCN (5.0 equiv)	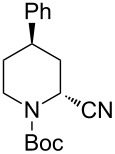 **2ae**	99	<1:99

^a^Isolated yield. ^b^Determined by GC analysis.

Next, we examined the reaction of *N*-Boc-4-methyl-2,3,4,5-tetrahydropyridin-1-ium (**C2**) with nucleophiles (Table 2). Both, the reactivity and diastereoselectivity of the reactions were similar to those of **C1** and the disubstituted piperidine derivatives **2ba**–**be** were obtained in good to high yields with excellent diastereoselectivities.

**Table 2:** Reaction of *N-*acyliminium ion **C2** with several nucleophiles.

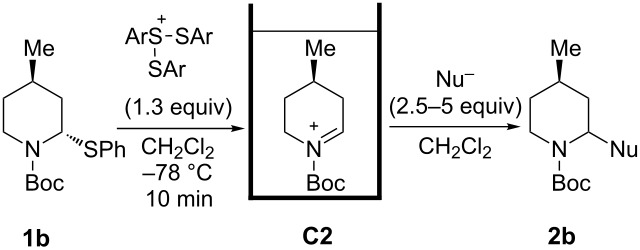				

entry	Nu^−^	**2**	yield (%)^a^	*cis*:*trans*^b^

1	Me_3_Al (2.5 equiv)	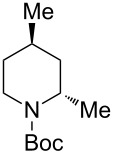 **2ba**	86	<1:99
2	Et_2_Zn (5.0 equiv)	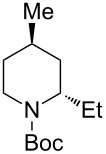 **2bb**	95	<1:99
3	(allyl)SnBu_3_ (2.5 equiv)	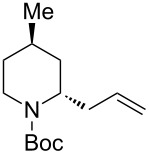 **2bc**	95	<1:99
4	PhMgBr (2.5 equiv)	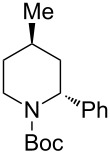 **2bd**	93	3:97
5	TMSCN (5.0 equiv)	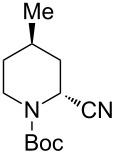 **2be**	89	<1:99

^a^Isolated yield. ^b^Determined by GC analysis.

Then, the reaction of 5-phenyl-substituted *N*-acyliminium cation **C3** was performed (Table 3). Interestingly, all reactions led to the 2,5-disubstituted piperidine derivatives **2ca–ce** in very good yields and high *cis*-selective manner. A similar trend is observed for the reactions of the 5-methyl-substituted cation **C4** and all products **2da**–**dc** were obtained in high to excellent *cis*-diastereoselectivities (Table 4).

**Table 3:** Reaction of *N-*acyliminium ion **C3** with several nucleophiles.

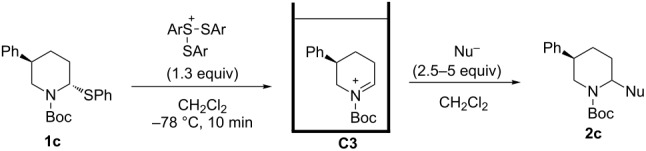				

entry	Nu^−^	**2**	yield (%)^a^	*cis*:*trans*^b^

1*^c^*	Me_3_Al (2.5 equiv)	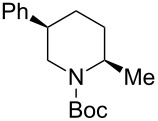 **2ca**	89	94:6
2	Et_2_Zn (2.5 equiv)	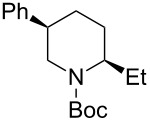 **2cb**	99	96:4
3*^c^*	(allyl)SnBu_3_ (2.5 equiv)	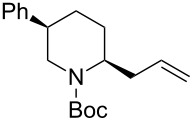 **2cc**	97	98:2
4	PhMgBr (2.5 equiv)	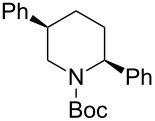 **2cd**	97	96:4*^c^*
5	TMSCN (5.0 equiv)	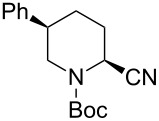 **2ce**	96	98:2

^a^Isolated yield. ^b^Determined by GC analysis. ^c^Determined by ^1^H NMR analysis.

**Table 4:** Reaction of *N-*acyliminium ion **C4** with several nucleophiles.

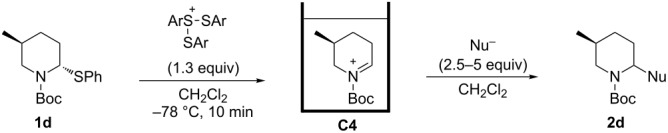				

entry	Nu^−^	**2**	yield (%)^a^	*cis*:*trans*^b^

1	Me_3_Al	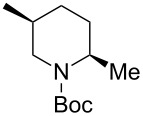 **2da**	73	98:2
2	Et_2_Zn	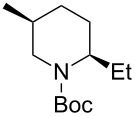 **2db**	82	95:5
3	(allyl)SnBu_3_	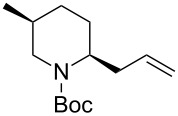 **2dc**	90	98:2
4	PhMgBr	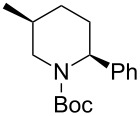 **2dd**	83	91:9
5	TMSCN	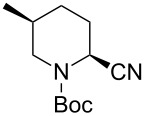 **2de**	98	96:4

^a^Isolated yield. ^b^Determined by GC analysis.

The tendency of the reactions of the 6-phenyl-substituted *N*-acyliminium cation **C5** with nucleophiles was slightly different compared to those of **C1**–**C4** (Table 5). The reactions proceeded in a *cis*-selective manner, however, the selectivity was not high except in case of product **2ed** (Table 5, entry 4). The low yield of **2ed** was due to the generation of an enamine as a byproduct. While the reason of the low selectivity has not been clear, it is probably due to steric repulsion between the phenyl and Boc groups. A distortion of the Boc group in **C5** was also suggested by DFT calculation (see Supporting Information File 1). The reaction of 6-methyl-substituted *N*-acyliminium cation **C6** bearing a methyl group, which is smaller than a phenyl group, gave the corresponding *cis*-products with high diastereoselectivities (Table 6).

**Table 5:** Reaction of *N-*acyliminium ion **C5** with several nucleophiles.

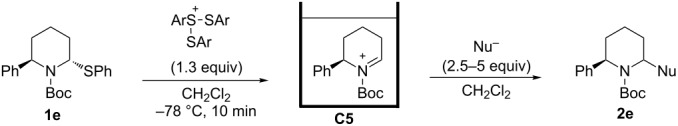				

entry	Nu^−^	**2**	yield (%)^a^	*cis*:*trans*^b^

1	Me_3_Al	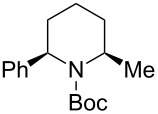 **2ea**	40	63:37
2	Et_2_Zn	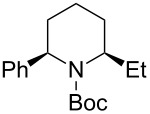 **2eb**	90	76:24
3	(allyl)SnBu_3_	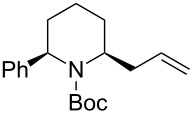 **2ec**	94	72:28
4	PhMgBr	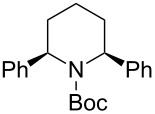 **2ed**	56	92:8
5	TMSCN	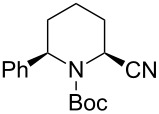 **2ee**	85	64:36

^a^Isolated yield. ^b^Determined by GC analysis.

**Table 6:** Reaction of *N-*acyliminium ion **C6** with several nucleophiles.

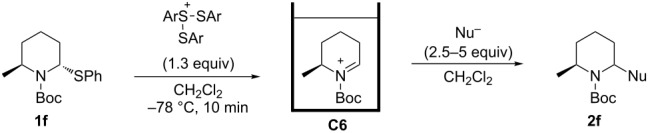				

entry	Nu^−^	**2**	yield (%)^a^	*cis*:*trans*^b^

1	Me_3_Al	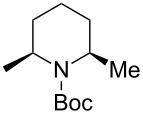 **2fa**	49	82:18
2	Et_2_Zn	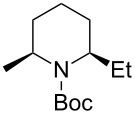 **2fb**	46	96:4
3	(allyl)SnBu_3_	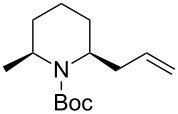 **2fc**	87	96:4
4	PhMgBr	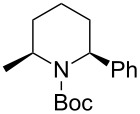 **2fd**	87	96:4
5	TMSCN	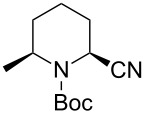 **2fe**	90	>99:1

^a^Isolated yield. ^b^Determined by GC analysis.

**Figure 1:**
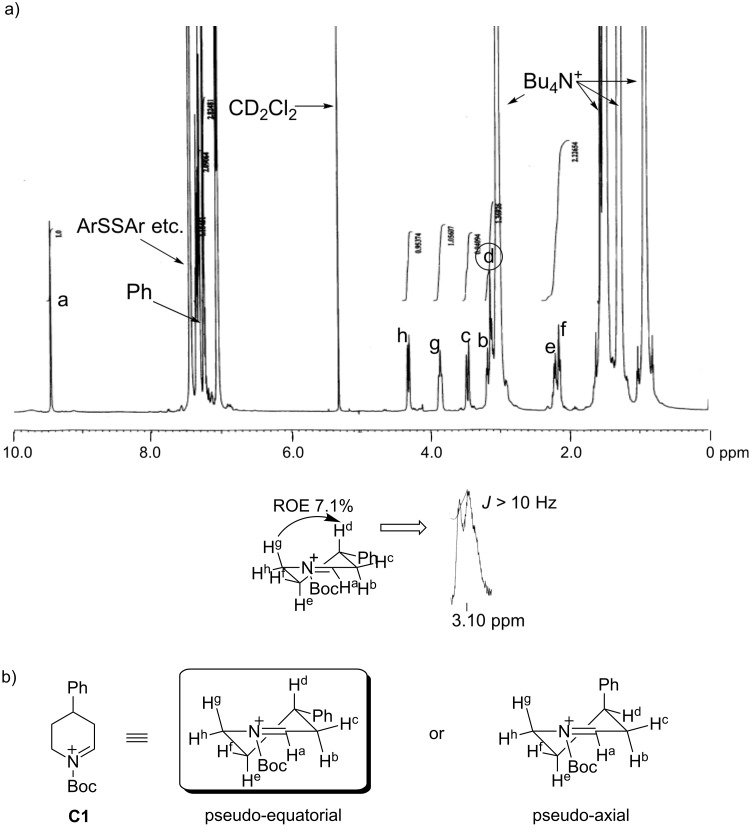
(a) ^1^H NMR of *N*-acyliminium ion **C1** in CD_2_Cl_2_ at −80 °C (600 MHz). (b) Preferred conformation of **C1**.

**Figure 2:**
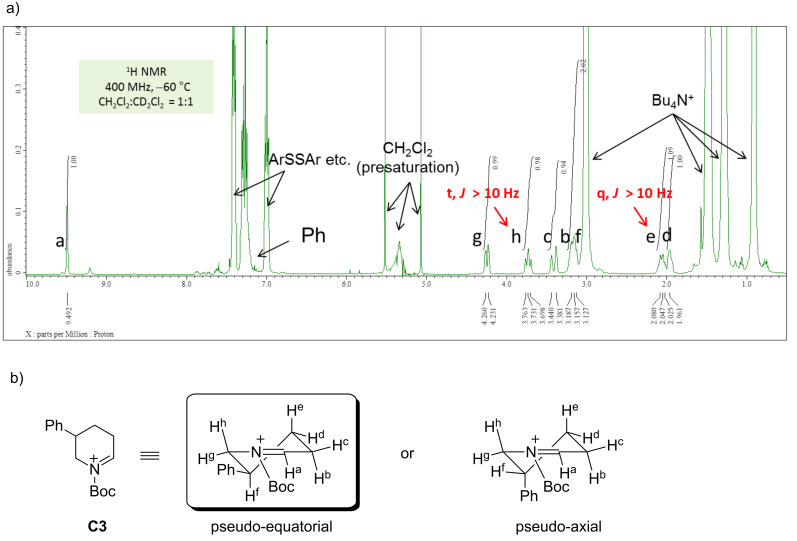
(a) ^1^H NMR spectrum of **C3** in CD_2_Cl_2_ at −60 °C (400 MHz). (b) Preferred conformation of **C3**.

**Figure 3:**
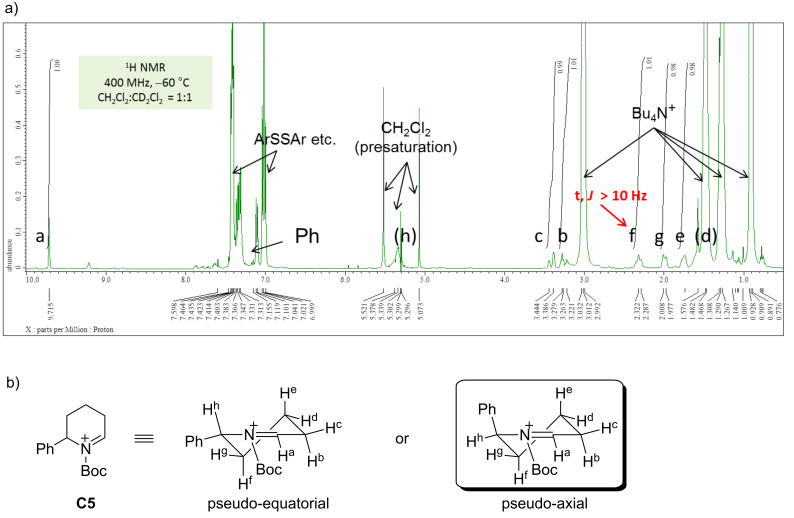
(a) ^1^H NMR spectrum of **C5** in CD_2_Cl_2_ at −60 °C (400 MHz). (b) Preferred conformation of **C5**.

As mentioned, the reaction between the cyclic *N*-acyliminium ions **C1**–**C6** and several nucleophiles proceeded diastereoselectively. To clarify the reason of the observed selectivity, ^1^H NMR analyses of **C1**–**C6** were performed at low temperature (Figures 1–3, see also Supporting Information File 1). All protons on the piperidine ring in **C1** (H^a^–H^h^) have been assigned by H–H COSY (Figure 1). In addition, the signal of H^d^ (3.10 ppm) exhibited an axial–axial coupling (*J* > 10 Hz) and 7.1% ROE was observed between H^d^ and H^g^. These results suggest that H^d^ adopts an axial and the phenyl group a pseudo-equatorial position at low temperature.

The low temperature NMR analysis of **C3**, which has a phenyl group at 5-position, was next performed and the ^1^H NMR spectrum is depicted in Figure 2. An axial–axial coupling between H^e^ and H^f^ was observed, suggesting that these two protons were located at axial positions. This result indicates that the phenyl group of **C3** was located in the pseudo-equatorial position.

**Figure 4:**
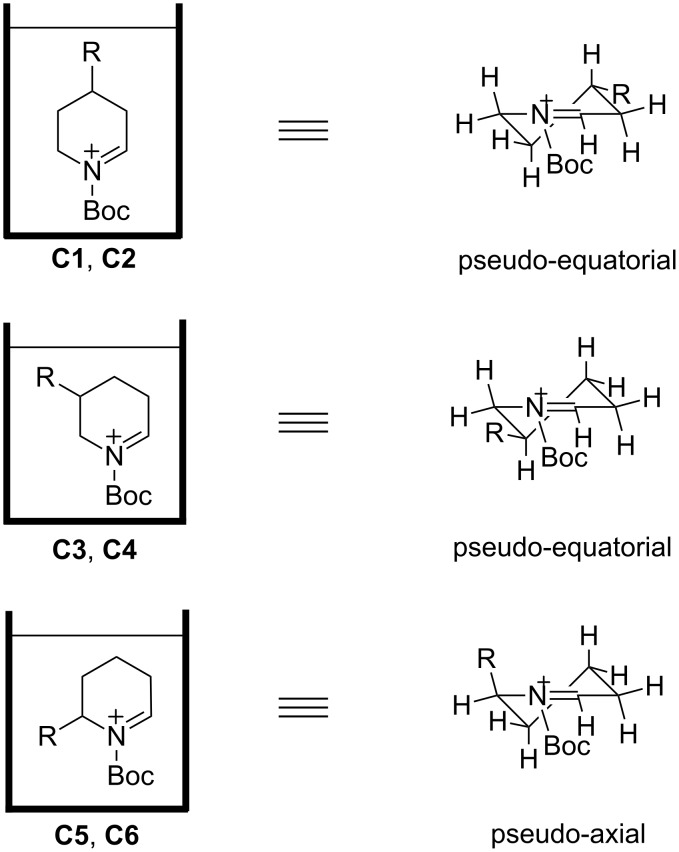
Summary of the conformations of *N*-acyliminium ions **C1**–**C6**.

The conformation of **C5**, an *N*-acyliminium ion bearing a phenyl group in the 6-position, was also examined by NMR analysis (Figure 3). The signal of H^f^ was observed as a triplet due to the axial–axial coupling with H^e^ and the geminal coupling with H^g^, and these results indicate that the phenyl group of **C5** is placed in the pseudo-axial position. The low temperature NMR measurements of *N*-acyliminium ions **C2**, **C4**, and **C6** were also performed. The conformations of **C2** and **C6** were similar to those of **C1** and **C5**, respectively (**C2**: pseudo-equatorial, **C6**: pseudo-axial). Although the conformation of **C4** could not be determined by the NMR analysis, the conformation is assumed to be similar to that of **C3** (pseudo-equatorial), because both ions led to similar stereoselectivities in the reactions with nucleophiles. As mentioned, the conformation of *N*-acyliminium cation **C1–C4** was pseudo-equatorial and that of **C5** and **C6** was pseudo-equatorial (Figure 4).

**Figure 5:**
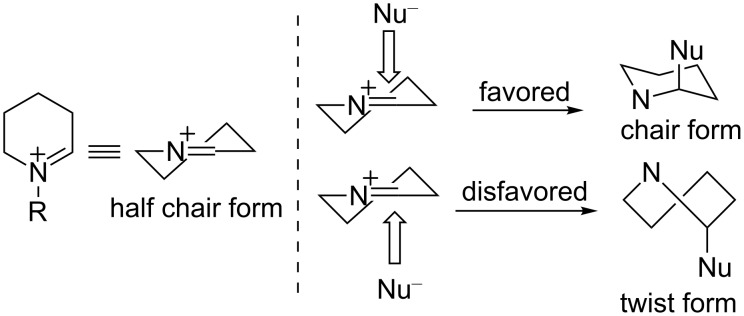
Stevens’ hypothesis on the tendency of the addition of nucleophiles to *N*-acyliminium ions. The substituent at the nitrogen atom is omitted for clarity.

**Figure 6:**
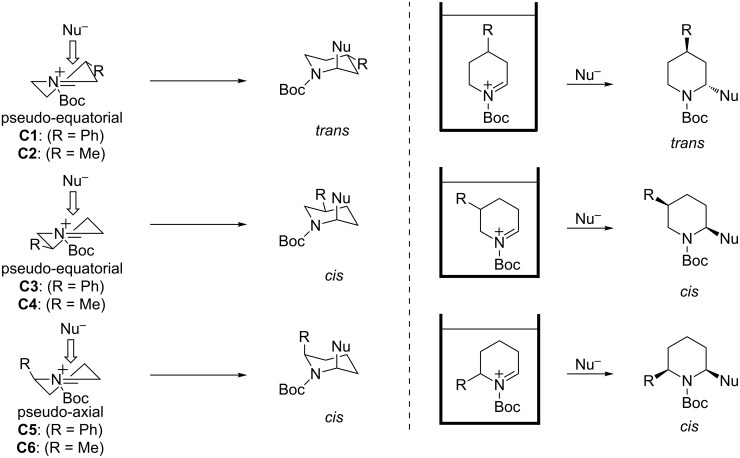
A plausible mechanism of the observed diastereoselective reaction of the *N*-acyliminium ions.

It is commonly assumed that the diastereoselectivity of the disubstituted piperidine derivatives **2a**–**f** was influenced by the conformation of the *N*-acyliminium ions **C1**–**C6**. In this context Stevens proposed that the conformation of the six-membered *N*-acyliminium ion would be a half-chair form and that the attack of the nucleophile proceeds from that side that generates a stable chair form product, because an attack from another side would lead to a more unstable product with a twist form [13]. As a close study, Woerpel reported a diastereoselective substitution reaction for synthesizing disubstituted tetrahydropyrans via six-membered oxocarbenium ions generated in situ from tetrahydropyran acetals [14]. If the Stevens’ hypothesis is true, a nucleophilic reaction with **C1**, having a pseudo-equatorial phenyl group, should give a *trans*-2,4-disubstituted product (Figure 5). Similarly, acyliminium ions **C3** and **C5** should give the *cis*-2,5- or *cis*-2,6-disubstituted piperidine derivatives, respectively. These explanations are consistent with above mentioned conformational analyses results, and the hypothesis was in agreement with the experimental results (Figure 6).

**Figure 7:**
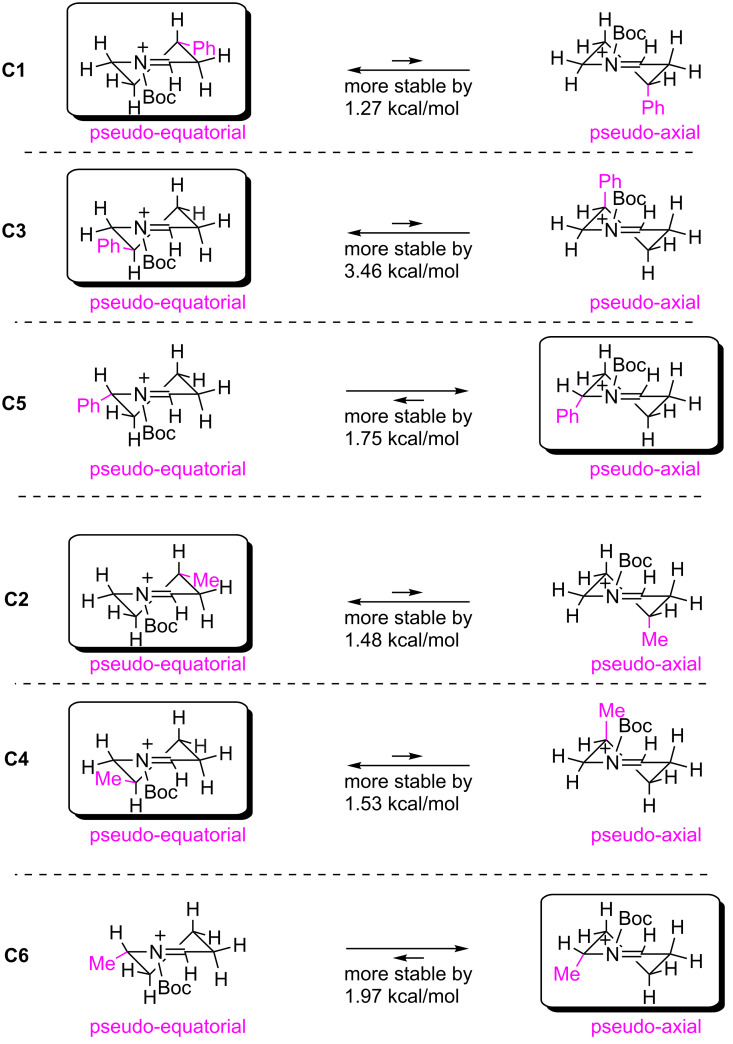
Comparison of Δ*G* for the pseudo-equatorial and pseudo-axial conformations of **C1**–**C6** at the B3LYP/6-31G(d) level.

To obtain further insight in the conformation of the cyclic *N*-acyliminium ions, DFT calculations of **C1**–**C6** were performed (Figure 7). First, Δ*G* of pseudo-equatorial and pseudo-axial conformations of **C1** was calculated at the B3LYP/6-31G(d) level of theory. The pseudo-equatorial conformation of **C1** was more stable than the pseudo-axial conformation by 1.27 kcal/mol. Similarly, DFT calculations of **C3** and **C5** were performed, and the results show that the pseudo-equatorial conformation of **C3** and the pseudo-axial conformation of **C5** were more stable than the other conformations, respectively. The more stable conformations that the calculations implied are consistent with the conformations determined by the low temperature NMR analyses, and the DFT calculations suggest that the preferred conformations of **C2**, **C4**, and **C6** were similar to those of **C1**, **C3**, and **C5**.

## Conclusion

In this study, we presented an efficient method for the highly diastereoselective synthesis of disubstituted piperidine derivatives through the reaction of *N*-acyliminium ions with nucleophiles. The starting *N*-acyliminium ions were generated by the indirect cation pool method and their conformations were confirmed by low temperature NMR analyses for the first time. The experimental results were fully consistent with DFT calculations. The correlation between the stereochemistry of the *N*-acyliminium ions and the reaction products is in agreement with common interpretation and further synthetic applications and more detailed investigations of the reaction mechanism are in progress in our laboratory.

## Supporting Information

File 1 Experimental details, ORTEP drawings of **1a**, **1b**, **1d**–**1f**, theoretical calculations of **C1**–**C6**, and NMR spectra of all new compounds and **C1**–**C6**.

File 2 X-ray structure analysis data for **1a** (CCDC-1813600), **1b** (CCDC-1813582), **1d** (CCDC-1813594), **1e** (CCDC-1813595), **1f** (CCDC-1813596). These data can be obtained free of charge from The Cambridge Crystallographic Data Centre via http://www.ccdc.cam.ac.uk/data_request/cif.
X-ray analysis of **1a**–**f**.
