# Supplementary material for: Stereoselective nucleophilic addition reactions to cyclic N-acyliminium ions using the indirect cation pool method: Elucidation of stereoselectivity by spectroscopic conformational analysis and DFT calculations
Source: Beilstein J Org Chem. 2018 May 24;14:1192–202. doi: 10.3762/bjoc.14.100 (PMC6009180; doi:10.3762/bjoc.14.100)
Supplement: File 1 — Experimental details, ORTEP drawings of 1a, 1b, 1d–1f, theoretical calculations of C1–C6, and NMR spectra of all new compounds and C1–C6. [file Beilstein_J_Org_Chem-14-1192-s001.pdf]

Supporting Information

for

Stereoselective nucleophilic addition reactions to cyclic *N*-acyliminium ions using the indirect cation pool method: Elucidation of stereoselectivity by spectroscopic conformational analysis and DFT calculations

Koichi Mitsudo,[1] Junya Yamamoto,[1] Tomoya Akagi,[1] Atsuhiro Yamashita,[1] Masahiro Haisa,[1] Kazuki Yoshioka,[1] Hiroki Mandai,[1] Koji Ueoka,[2] Christian Hempel,[2] Jun-ichi Yoshida,[2] and Seiji Suga*[1]

Address: [1]Division of Applied Chemistry, Graduate School of Natural Science and Technology, Okayama University, 3-1-1 Tsushima-naka, Kita-ku, Okayama 700-8530, Japan and [2]Department of Synthetic Chemistry and Biological Chemistry, Graduate School of Engineering, Kyoto University, Nishikyo-ku, Kyoto 615-8510, Japan

Email: Seiji Suga - suga@cc.okayama-u.ac.jp

* Corresponding author

Experimental details, ORTEP drawings of **1a**,**1b**, **1d**–**1f**, theoretical calculations of **C1**–**C6**, and NMR spectra of all new compounds and **C1**–**C6**.

Table of Contents

Experimental section S2
X-ray crystallography S21
Theoretical calculations S32
NMR spectra of products and *N*-acyliminium ions S62
References S113

General remarks

GC analysis was performed on a gas chromatograph (SHIMADZU GC-14B) equipped with a flame ionization detector using a fused silica capillary. Nuclear magnetic resonance (NMR) spectra were recorded on a JEOL JNM-ECS400 (^{1}H 400 MHz, ^{13}C 100 MHz), Varian VNMRS-400 (^{1}H 400 MHz, ^{13}C 100 MHz), Varian VNMRS-600 (^{1}H 600 MHz, ^{13}C 150 MHz), or JEOL ECA-600P (^{1}H 600 MHz, ^{13}C 150 MHz) spectrometer. Chemical shifts for ^{1}H NMR are expressed in parts per million (ppm) relative to tetramethylsilane (δ 0.00 ppm) or residual $CHCl_3$ in $CDCl_3$ (δ 7.26 ppm). Chemical shifts for ^{13}C NMR are expressed in ppm relative to residual $CDCl_3$ (δ 77.0 ppm). IR spectra were measured using a JASCO FT/IR-4100 spectrometer. Thin-layer chromatography (TLC) was carried out on Merck pre-coated silica gel F_{254} plates (thickness 0.25 mm). Flash chromatography was carried out on KANTO CHEMICAL Silica Gel 60N (40–50 μm). All reactions were carried out under Ar atmosphere unless otherwise noted.

Materials

Dichloromethane was washed with water, distilled from P_2O_5, redistilled from dried K_2CO_3 to remove trace amounts of acid, and stored over molecular sieves 4 Å. Trifluoromethanesulfonic acid was purchased from Tokyo Chemical Industries (TCI) and used without further purification. Tetrabutylammonium tetrafluoroborate (Bu_4NBF_4) was purchased from TCI and dried over P_4O_{10} under reduced pressure. Dry diethyl ether (Et_2O) and tetrahydrofuran (THF) were purchased from Wako Pure Chemical Industries. ArSSAr (Ar = *p*-FC_6H_4) was prepared according to the procedure in the literature.[1]

Preparation of *N*-acyliminium ion precursors

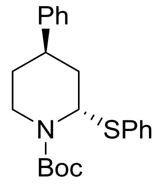

***N*-Boc-*trans*-4-phenyl-2-phenylthiopiperidine (1a).** To a solution of *N*-Boc-4-phenylpiperidine (2.61 g, 10.0 mmol) in dry Et_2O (18 mL) was slowly added TMEDA (1.94 mL, 1.15 g, 13.0 mmol) at −78 °C. To this mixture was added dropwise *sec*-BuLi (1.03 M in cyclohexane, 12.6 mL, 13.0 mmol) at the same temperature. The mixture was slowly warmed to −40 °C, stirred for 30 min, and then cooled again to −78 °C. To this mixture was added dropwise diphenyl disulfide (PhSSPh, 4.37 g, 20.0 mmol) in Et_2O (17 mL). Then the mixture was slowly allowed to warm to room temperature and stirred for 3 h. The reaction mixture was quenched with saturated aqueous $NaHCO_3$ (20 mL), extracted with Et_2O (3 × 10 mL). The combined organic phase was dried over $MgSO_4$, and concentrated under reduced pressure. The residue was purified by flash chromatography on silica gel (hexane/EtOAc 30:1) and subsequent recrystallization from hexane to afford the title compound as a colorless solid (2.52 g, 6.82 mmol, 68% yield); ^{1}H NMR (600 MHz, $CDCl_3$) (rotamer) δ 7.57–7.49 (m, 2H), 7.34–7.20 (m, 8H), 6.17 and 5.90 (d, *J* = 4.1 Hz, 1H), 4.22 and 4.00 (d, *J* = 13.4 Hz, 1H), 3.48 (ddd, *J* = 2.8, 13.2, 13.2 Hz, 1H), 3.25 and 3.20 (dddd, *J* = 3.4, 3.4, 12.7, 12.7 Hz, 1H), 2.19 and 2.14 (d, *J* = 13.4 Hz, 1H), 2.08–2.00 (m, 1H), 1.95 and 1.89 (d, *J* = 13.0 Hz, 1H), 1.68 and 1.64 (dddd, *J* = 4.5, 12.7, 12.7, 12.7 Hz, 1H), 1.33 and 1.18 (s, 9H),); ^{13}C NMR (150 MHz, $CDCl_3$) (rotamer) δ 153.9 and 153.7, 145.1 and 145.0, 135.1 and 134.1, 133.6 and 133.1, 129.0, 128.8, 128.6, 128.2 and 127.7,

126.8 and 126.5, 80.2 and 80.1, 64.1 and 61.9, 39.6 and 38.6, 38.3 and 37.6, 37.4 and 37.3, 33.0 and 32.9, 28.2 and 27.9; IR (KBr) 2978, 1682, 1412, 1171 cm^{-1}; HRMS (FAB^+) calcd for $C_{22}H_{28}NO_2S$ $[M + H]^+$ 370.1841, found: 370.1843. mp 71.9–72.3 °C.

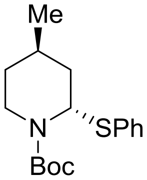

***N*-Boc-*trans*-4-methyl-2-phenylthiopiperidine (1b).** To a solution of 4-methylpiperidine (1.98 g, 20.0 mmol) and DMAP (0.0999 g, 0.818 mmol) in toluene (30 mL) was added slowly $(Boc)_2O$ (4.59 mL, 4.80 g, 22.0 mmol) at 0 °C. After being stirred at room temperature for 30 min, the reaction was quenched with water. The solvent was removed under reduced pressure. The residue was quickly filtered through a short column of alumina. The combined solution was concentrated to give crude *tert*-butyl 4-methylpiperidinecarboxylate (3.35 g, 12.9 mmol, 65%) which was used directly for the next step. To a solution of *tert*-butyl 4-methylpiperidinecarboxylate (3.35 g, 12.9 mmol) in dry Et_2O (17 mL) was slowly added TMEDA (2.51 mL, 1.95 g, 16.8 mmol) at −78 °C, followed by dropwise addition of *sec*-BuLi (1.02 M in hexane, 16.5 mL, 16.8 mmol) at the same temperature. Then the mixture was slowly warmed to −40 °C, stirred for 30 min, and then cooled again to −78 °C. To this mixture was added dropwise diphenyl disulfide (PhSSPh, 11.3 g, 51.6 mmol) in Et_2O (22 mL). Then the mixture was slowly allowed to warm to room temperature and stirred for 24 h. The reaction mixture was quenched with water (30 mL), extracted with Et_2O (3×30 mL). The combined organic phase was washed with saturated aqueous $NaHCO_3$ (30 mL), dried over $MgSO_4$, and concentrated under reduced pressure. The residue was purified by flash chromatography (hexane/EtOAc 30:1 to 10:1) to obtain the title compound (9.90 g, 3.22 mmol, 25%) as a colorless solid; ^1H NMR (600 MHz, $CDCl_3$) δ 7.50–7.47 (m, 2H), 7.29–7.22 (m, 3H), 6.05 and 5.78 (d, J = 4.2 Hz, 1H) (rotamer), 4.07 and 3.85 (d, J = 12.6 Hz, 1H) (rotamer), 3.33 (ddd, J = 13.2, 13.2, 2.4 Hz, 1H), 2.09–1.95 (m, 1H), 1.72 and 1.65 (d, J = 12.6 Hz, 1H), 1.51 (ddd, J = 13.8, 13.2, 6.0 Hz, 1H), 1.30 and 1.13 (s, 9H) (rotamer), 1.14–1.06 (m, 1H), 0.94–0.92 (m, 3H); ^{13}C NMR (150 MHz, $CDCl_3$) δ 153.7, 139.4, 128.9, 128.0, 79.8, 64.1, 39.6, 38.0, 33.9, 28.2, 27.9, 26.3, 21.9; IR (KBr) 2929, 1698, 1404, 1159, 1088 cm^{-1}; HRMS (FAB^+) calcd for $C_{17}H_{25}NO_2SNa$ $[M + Na]^+$ 330.1504, found 330.1508; mp 79.9–80.8 °C.

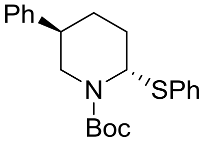

***N*-Boc-*trans*-5-phenyl-2-phenylthiopiperidine (1c).** A solution of 3-phenylpiperidine (1.00 g, 6.2 mmol) and trimethylamine (0.86 mL, 1.0 equiv) in dry THF (6.2 mL) was cooled to 0 °C, and $(Boc)_2O$ (1.42 mL, 6.2 mmol) was added in portions. The mixture was stirred for 10 min, and warmed to room temperature. After being stirred for 4 h at room temperature, the mixture was diluted with water (5 mL), and extracted with Et_2O (3 × 10 mL). The combined organic phase was dried over $MgSO_4$ and the solvent removed to obtain *N*-Boc-3-phenylpiperidine (1.78 g, 6.7 mmol, >98% yield) which was used without further

purification. To a solution of *N*-Boc-3-phenylpiperidine (1.98 g, 7.6 mmol) in dry Et_2O (14 mL) was slowly added TMEDA (1.5 mL, 1.15 g, 9.9 mmol) at −78 °C followed by dropwise addition of *sec*-BuLi (1.07 M in cyclohexane, 9.3 mL, 9.9 mmol) at the same temperature. The mixture was slowly warmed to −40 °C, stirred for 30 min, and then cooled again to −78 °C. To this mixture was added dropwise diphenyl disulfide (PhSSPh, 3.34 g, 15.3 mmol) in Et_2O (13 mL). Then the mixture was slowly allowed to warm to room temperature and stirred for 24 h. The reaction mixture was quenched with water (20 mL), extracted with Et_2O (3 × 10 mL). The combined organic phase was washed with saturated aqueous $NaHCO_3$ (20 mL), dried over $MgSO_4$, and concentrated under reduced pressure. The residue was purified by flash chromatography on silica gel (hexane/EtOAc/Et_3N 30:1:0.03) and subsequent recrystallization from hexane to afford the title compound (1.02 g, 2.77 mmol, 41% yield, 2 steps) as a colorless solid; ^1H NMR (400 MHz, $CDCl_3$) δ 7.53–7.51 (m, 2H), 7.32–7.23 (m, 7H), 7.18–7.12 (t, *J* = 7.2 Hz, 1H), 5.90 (s, 1H), 4.39 (d, *J* = 14.0 Hz), 3.74 (dd, *J* = 14.0, 4.4 Hz, 1H), 3.12 (s, 1H), 2.42 (tt, *J* = 13.9 Hz, 4.7 Hz, 1H), 2.05–1.85 (m, 2H), 1.77 (d, *J* = 14.0 Hz, 1H); ^{13}C NMR (100 MHz, $CDCl_3$) δ 154.1, 143.3, 134.7, 133.8, 128.9, 128.8, 127.8, 127.6, 126.0, 80.2, 63.1, 41.6, 37.4, 31.8, 28.1, 26.2, 25.8; IR (KBr) 2982, 2882, 1697, 1364, 1169 cm^{-1}, mp 54.3–54.8 °C.

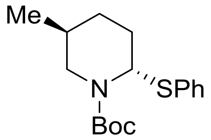

***N*-Boc-*trans*-5-methyl-2-phenylthiopiperidine (1d).** To a solution of 3-methylpiperidine (2.92 mL, 2.48 g, 25.0 mmol) in dry Et_2O (25 mL) was added slowly $(Boc)_2O$ (5.61 mL, 5.33 g, 23.1 mmol) at 0 °C. After being stirred at room temperature for 1 h, the reaction was quenched with water. The mixture was extracted with Et_2O (3×20 mL) and the combined organic phase was washed with saturated aqueous $NaHCO_3$, and drying over $MgSO_4$. After removal of solvent, *tert*-butyl 3-methylpiperidinecarboxylate (4.56 g, 22.8 mmol, 99%) was obtained. To a solution of *tert*-butyl 3-methylpiperidinecarboxylate (1.99 g, 10.0 mmol) in dry Et_2O (18.5 mL) was slowly added TMEDA (1.95 mL, 1.51 g, 13.0 mmol) at −78 °C followed by dropwise addition of *sec*-BuLi (1.07 M in cyclohexane, 12.1 mL, 13.0 mmol) at the same temperature. The mixture was slowly warmed to −40 °C, stirred for 30 min, and then cooled again to −78 °C. To this mixture was added dropwise diphenyl disulfide (PhSSPh, 6.55 g, 30.0 mmol) in Et_2O (25 mL). Then the mixture was slowly allowed to warm to room temperature and stirred for 24 h. The reaction was quenched with water and extracted with Et_2O. The combined organic phase was washed with saturated aqueous $NaHCO_3$, dried over $MgSO_4$, filtered and concentrated under reduced pressure. The residue was purified by flash chromatography on silica gel (hexane/EtOAc 10:1) and subsequent recrystallization to afford the title compound (1.29 g, 4.20 mmol, 42%) as a colorless solid; ^1H NMR (400 MHz, $CDCl_3$, 60 °C) δ 7.52–7.40 (m, 2H), 7.31–7.15 (m, 3H), 5.89 (s, 1H), 3.70 (d, *J* = 14.0 Hz, 1H), 3.50 (dd, *J* = 13.2, 3.2 Hz, 1H), 2.20–2.12 (m, 2H), 2.20–1.98 (m, 2H), 1.81–1.64 (m, 1H), 1.51–1.34 (m, 1H), 0.98 (d, *J* = 6.8 Hz, 3H); ^{13}C NMR (100 MHz, $CDCl_3$, 60 °C) δ 154.6, 134.2, 134.1, 128.8, 127.6, 79.7, 62.9, 43.8, 28.2, 27.7, 25.7, 25.6, 16.3; IR (KBr) 2970, 1683, 1408, 862, 754 cm^{-1}; HRMS (FAB^+) calcd for $C_{17}H_{25}NO_2SNa$ $[M + Na]^+$ 330.1500, found 330.1504, mp 79.9–80.8 °C.

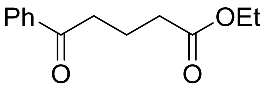

Ethyl 5-oxo-5-phenylpentanoate (S1).[2] A mixture of glutaric anhydride (3.42 g, 30.0 mmol) and Fe(acac)$_3$ (0.32 g, 0.90 mmol, 3 mol %) and dry THF (36.0 mL) was cooled to 0 °C, and PhMgBr (1.1 M in THF, 25.9 mL, 28.5 mmol) was added over a period of 60 min with the aid of syringe pump. The reaction mixture was stirred overnight at the same temperature. Then the mixture was acidified with 2 M HCl aqueous solution (20 mL) and extracted with Et_2O (3 × 10 mL). The combined organic phase was extracted with a 1 M aqueous NaOH solution (3 × 15 mL), discarding the organic layer. The combined aqueous basic phase was acidified with 2 M aqueous HCl in order to obtain pH around 1, and this mixture was extracted with Et_2O (3 × 20 mL). The combined organic phase was dried over $MgSO_4$. After removal of solvent, the crude (5.0 mmol) was dissolved in EtOH (25.0 mL), concentrated H_2SO_4 (0.79 mL, 15.0 mmol) was added at room temperature, and then the reaction mixture was refluxed overnight. After evaporation of the solvent, H_2O (10 mL) was added, and the mixture was neutralized with a 2 M aqueous NaOH solution (5 mL) and extracted with Et_2O (3 × 20 mL). The combined organic phase was dried over $MgSO_4$, and concentrated under reduced pressure. The residue was purified by flash chromatography on silica gel (hexane/EtOAc 10:1) to afford **S1** (2.2158 g, 2.45 mmol, 49% yield, 2 steps) as a colorless oil: ^{1}H NMR (400 MHz, $CDCl_3$) δ 7.95 (m, 2H), 7.54 (m, 1H), 7.50–7.37 (m, 2H), 4.13 (q, J = 7.2 Hz, 2H), 3.05 (t, J = 7.2 Hz, 2H), 2.42 (t, J = 7.2 Hz, 2H), 2.06 (quin, J = 7.2 Hz, 2H), 1.24 (t, J = 7.2 Hz, 3H); ^{13}C NMR (100 MHz, $CDCl_3$) δ 199.4, 173.2, 136.8, 133.0, 128.5, 130.0, 60.3, 37.4, 33.3, 19.3, 14.1; IR (neat) 2974, 1680, 1416, 1366, 1169 cm^{-1}.

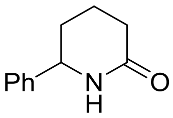

6-Phenyl-2-piperidone (S2).[3] A mixture of **S1** (2.78 g, 12.6 mmol), HCO_2NH_4 (2.39 g, 37.8 mmol), AcOH (0.72 mL, 12.6 mmol), and chloro[*N*-[4-(dimethylamino)phenyl]-2-pyridinecarboxamidato](pentamethyl-cyclopentadienyl)iridium(III) (15.1 mg, 0.025 mmol, 0.2 mol %) in CH_2Cl_2 (126 mL) was heated to 60 °C. After being stirred at 60 °C for 8 h, the mixture was quenched by saturated aqueous $NaHCO_3$ (20 mL), and extracted with CH_2Cl_2 (3 × 20 mL). To the combined organic phase was added TsOH·H_2O (0.60 g, 2.9 mmol) at room temperature, and then the mixture was stirred overnight. The reaction mixture was quenched by saturated aqueous $NaHCO_3$ (20 mL), and extracted with CH_2Cl_2 (3 × 20 mL). The combined organic phase was dried over $MgSO_4$ and concentrated under reduced pressure. The residue was purified by filtration to afford **S2** (1.77 g, 10.1 mmol, 80% yield, 2 steps) as a colorless solid: ^{1}H NMR (400 MHz, $CDCl_3$) δ 7.39–7.29 (m, 2H), 7.29–7.16 (m, 3H), 5.94 (brs, 1H), 4.51 (dd, J = 8.7, 4.6 Hz, 1H), 2.53–2.29 (m, 2H), 2.17–1.99 (m, 1H), 1.97–1.82 (m, 1H), 1.82–1.71 (m, 1H), 1.70–1.56 (m, 1H); ^{13}C NMR (100 MHz, $CDCl_3$) δ 172.2, 142.5, 128.8, 127.9, 126.0, 57.7, 32.1, 31.2, 19.6; IR (KBr) 3065, 1645, 1402, 1306, 1150 cm^{-1}.

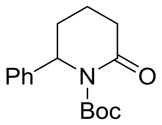

***N*-Boc-6-phenyl-2-piperidone (S3).**[3] To a solution of **S2** (1.76 g, 10.1 mmol) in dry tetrahydrofuran (100 mL) was added dropwise *n*-BuLi (1.6 M in hexane, 12.5 mL, 20.0 mmol) at −78 °C. After 10 min, a solution of $(Boc)_2O$ (4.6 mL, 20.0 mmol) in tetrahydrofuran (40 mL) was transferred via syringe. After stirring at −78 °C for 2 h, the reaction mixture was quenched with saturated aqueous NH_4Cl (20 mL). The reaction mixture was extracted with Et_2O (3 × 20 mL). The combined organic phase was dried over $MgSO_4$ and concentrated under reduced pressure. The residue was purified by flash chromatography on silica gel (hexane/EtOAc 3:1) to afford **S3** (2.6635 g, 9.7 mmol, 96% yield) as a colorless solid: ^{1}H NMR (400 MHz, $CDCl_3$) δ 7.32 (t, *J* = 7.3 Hz, 2H), 7.28–7.23 (m, 1H), 7.23–7.15 (m, 2H), 5.20 (t, *J* = 5.3 Hz, 1H), 2.70–2.49 (m, 2H), 2.23–2.08 (m, 1H), 1.99–1.82 (m, 1H), 1.82–1.62 (m, 2H), 1.29–1.16 (m, 9H); ^{13}C NMR (100 MHz, $CDCl_3$) δ 171.4, 151.9, 142.5, 128.5, 127.2, 125.7, 82.9, 60.7, 34.5, 3.6, 27.5, 17.4; IR (KBr) 3049, 1757, 1668, 1296, 1142 cm^{-1}.

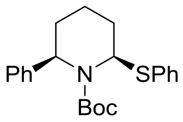

***N*-Boc-*cis*-6-phenyl-2-phenylthiopiperidine (1e).**[4,5] To a solution of **S3** (0.5509 g, 2.0 mmol) in dry THF (4 mL) was added dropwise DIBAL (1.0 M in hexane, 3.0 mL, 3.0 mmol) at −78 °C. The reaction mixture was stirred for 2 h and then quenched with a saturated potassium acetate solution (4.0 mL) at −78 °C. To the mixture was added saturated NH_4Cl/ether 1:3 (30 mL). The solution was warmed to room temperature and stirred until a thick white gel formed at the bottom, and the mixture was then filtered through a Celite. The aqueous layer was extracted with Et_2O (3 × 10 mL), and the combined organic phase was washed with saturated aqueous NH_4Cl (10 mL) and dried over $MgSO_4$, and concentrated under reduced pressure to afford crude intermediate, which was used directly in the next step. The solution of a mixture of the crude (2.0 mmol), thiophenol (2.0 mL, 20.0 mmol, 10.0 equiv), *p*-toluenesulfonate acid monohydrate (50.2 mg, 0.2 mmol, 0.1 equiv), PPTS (38.2 mg, 0.2 mmol, 0.1 equiv) in anhydrous CH_2Cl_2 (2.0 mL) was stirred at room temperature for 24 h. The reaction was quenched with 3 M aqueous NaOH solution (1 mL) and the mixture was extracted with CH_2Cl_2 (3 × 10 mL). The combined organic phase was dried over $MgSO_4$ and concentrated under reduced pressure. The residue was purified by flash chromatography on silica gel (hexane/EtOAc/Et_3N 30:1:0.03) and subsequent recrystallization to afford **1e** (0.18 g, 0.50 mmol, 25% yield, 2 steps) as a colorless solid: ^{1}H NMR (400 MHz, $CDCl_3$) δ 7.53–7.53 (m, 2H), 7.32–7.23 (m, 7H), 7.18–7.12 (m, 1H), 6.06 and 5.82 (s, 1H) (rotamer), 5.29 and 4.98 (t, *J* = 4.8 Hz, 1H) (rotamer), 2.59–2.36 (m, 1H), 2.24–2.08 (m, 1H), 2.08–1.80 (m, 3H), 1.72–1.57 (m, 1H), 1.39 and 1.17 (s, 9H) (rotamer); ^{13}C NMR (100 MHz, $CDCl_3$) δ 155.0, 144.4 and 142.6 (rotamer), 136.0 and 135.5 (rotamer), 133.6 and 133.0 (rotamer), 128.9 and 128.7 (rotamer), 128.1 and 128.1 (rotamer), 127.3 and 127.0 (rotamer), 126.6, 126.2, 125.5, 80.7, 64.3 and 62.0 (rotamer), 55.7 and 53.6 (rotamer), 30.9 and 29.4 (rotamer), 27.8, 15.9 and 14.5 (rotamer); IR (KBr) 2968, 1688, 1481, 1366, 1161 cm^{-1}; HRMS (ESI$^+$) calcd for $C_{22}H_{28}NO_2S$ $[M + H]^+$ 302.2115, found 302.2114, mp 68.9–69.4 °C.

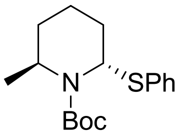

***N*-Boc-*trans*-6-methyl-2-phenylthiopiperidine (1f).** To a solution of 2-methylpiperidine (5.86 mL, 4.96 g, 50.0 mmol) in dry Et_2O (50 mL) was added slowly $(Boc)_2O$ (11.0 mL, 10.5 g, 48.0 mmol) at 0 °C. After being stirred at room temperature for 1 h, the reaction was quenched with water. The mixture was extracted with Et_2O (3 × 20 mL) and the combined organic phase was washed with saturated aqueous $NaHCO_3$ and dried over $MgSO_4$. After removal of solvent, *tert*-butyl 2-methylpiperidinecarboxylate (9.00 g, 45.0 mmol, 94%) was obtained. To a solution of *tert*-butyl 2-methylpiperidinecarboxylate (1.99 g, 10.0 mmol) in dry Et_2O (18.5 mL) was slowly added TMEDA (1.95 mL, 1.51 g, 13.0 mmol) at −78 °C followed by dropwise addition of *sec*-BuLi (1.07 M in cyclohexane, 12.1 mL, 13.0 mmol) at the same temperature. The mixture was slowly warmed to −40 °C, stirred for 30 min, and then cooled again to −78 °C. To this mixture was added dropwise diphenyl disulfide (PhSSPh) (4.36 g, 20.0 mmol) in Et_2O (16.7 mL). Then the mixture was slowly allowed to warm to room temperature and stirred for 24 h. The reaction was quenched with water and extracted with ether. The combined organic phase was washed with saturated aqueous $NaHCO_3$, dried over $MgSO_4$, and concentrated under reduced pressure. The residue was purified by flash chromatography on silica gel (hexane/EtOAc = 10/1) and subsequent recrystallization to afford the title compound (1.73 g, 5.64 mmol, 56%) as a colorless solid; ^{1}H NMR (400 MHz, $CDCl_3$, 60 °C) δ 7.54 (d, *J* = 7.2, Hz, 2H), 7.36–7.16 (m, 3H), 5.74 (brs, 1H), 4.39–4.25 (m, 1H), 2.13 (qt, *J* = 13.3, 4.2 Hz, 1H), 1.96 (dt, *J* = 13.7, 1.6, 1H), 1.84–1.56, (m, 2H), 1.56–1.33, (m, 14H); ^{13}C NMR (100 MHz, $CDCl_3$,60 °C) δ 154.4, 136.2, 133.4, 128.9, 127.3, 80.2, 63.0, 46.7, 31.2, 30.1, 28.4, 19.9, 14.4; IR (KBr) 2943, 1699, 1087, 752, 478 cm^{-1}; HRMS (FAB^+) calcd for $C_{17}H_{26}NO_2S$ $[M + H]^+$ 308.1684, found 308.1709, mp 56.3–57.0 °C.

Electrochemical generation and accumulation of $ArS(ArSSAr)^+$ (Ar = *p*-FC_6H_4). Analogous as described in our previous report.[6] The anodic oxidation was carried out in an H-type divided cell (4G glass filter) equipped with a carbon felt anode (Nippon Carbon JF-20-P7, ca. 320 mg, dried at 250 °C/1 mmHg for 1 h before use) and a platinum plate cathode (40 mm × 20 mm). In the anodic chamber was placed a solution of ArSSAr (Ar = *p*-FC_6H_4) (101.9 mg, 0.401 mmol) in 0.3 M Bu_4NBF_4/CH_2Cl_2 (8.0 mL). In the cathodic chamber were placed 0.3 M Bu_4NBF_4/CH_2Cl_2 (8.0 mL) and trifluoromethanesulfonic acid (41.0 mg, 0.273 mmol). The constant current electrolysis (8 mA) was carried out at –78 °C with magnetic stirring until 0.67 F/mol of electricity was consumed. The solution of $ArS(ArSSAr)^+$ (0.0377 M at −80 °C) thus obtained was used for the subsequent reaction.

Generation of *N*-acyliminium ions and reactions with carbon nucleophiles

Typical procedure of the indirect cation pool method. To a solution of a cation precursor (0.150 mmol) in 0.3 M Bu_4NBF_4/CH_2Cl_2 (1.0 mL) was added a solution of $ArS(ArSSAr)^+$ (0.0377 M, 5.0 ml, 0.189 mmol) at −78 °C and the reaction mixture was stirred for 10 min. To the “cation pool” thus generated, was added the corresponding nucleophile at −78 °C and the reaction mixture was stirred for 5 min and immediately quenched by the addition of Et_3N (1 mL) at the same temperature. Then the mixture was warmed to room temperature and the solvent was removed under reduced pressure. The residue was quickly filtered through a

short column (2 cm) of silica gel to remove Bu_4NBF_4. The silica gel was washed with Et_2O (50 mL). The combined solution was concentrated to give the crude product, which was purified by flash chromatography.

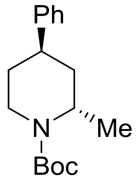

***N*-Boc-*trans*-2-methyl-4-phenylpiperidine (2aa).** Prepared from **1a** (57.5 mg, 0.156 mmol) and trimethylaluminum (1.08 M in toluene, 0.722 ml, 0.156 mmol) following the typical procedure; isolated yield 85% (colorless oil, 36.6 mg, 0.132 mmol, purified with flash chromatography, hexane/EtOAc = 20/1); ^{1}H NMR (600 MHz, $CDCl_3$) δ 7.23–7.20 (m, 1H), 4.62 and 4.47 (s, 1H), (rotamer), 4.14 and 4.02 (d, *J* = 13.2 Hz, 1H) (rotamer), 3.18–2.92 (m, 1H), 2.89 (dd, *J* = 12.8, 12.4 Hz, 1H), 1.83 (ddd, *J* = 12.8, 9.6, 5.6 Hz, 2H), 1.74–1.68 (m, 1H), 1.64–1.50 (m, 1H), 1.49 (s, 9H), 1.23 (d, *J* = 7.2 Hz, 3H); ^{13}C NMR (150 MHz, $CDCl_3$) δ 155.0 and 154.7 (rotamer), 145.8, 128.4, 126.8, 126.2, 79.2, 46.9 and 45.5 (rotamer), 39.3 and 38.3 (rotamer), 38.0 and 37.7 (rotamer), 33.3 and 33.0 (rotamer), 16.2 and 15.9 (rotamer); IR (neat) 2973, 1690, 1411, 1167, 699 cm^{-1}; Anal Calcd for $C_{17}H_{25}NO_2$ C, 74.14; H, 9.15; O, 5.09. Found C, 74.34; H, 8.92; O, 4.95.

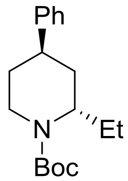

***N*-Boc-*trans*-2-ethyl-4-phenylpiperidine (2ab).** Prepared from **1a** (55.1 mg, 0.149 mmol) and diethyl zinc (1.0 M in hexane, 0.450 ml, 0.450 mmol) following the typical procedure; isolated yield 89% (colorless oil, 38.6 mg, 0.135 mmol, purified with flash chromatography, hexane/EtOAc = 20/1); ^{1}H NMR (600 MHz, $CDCl_3$) δ 7.32–7.29 (m, 2H), 7.22–7.19 (m, 3H), 4.37–4.04 (m, 2H), (rotamer), 2.98–2.82 (m, 2H), 1.85–1.74 (m, 4H), 1.62–1.52 (m, 2H), 1.49 (s, 9H), 0.91 (t, *J* = 7.5 Hz, 3H); ^{13}C NMR (150 MHz, $CDCl_3$) δ 145.9, 128.5, 126.8, 126.3, 79.2 and 79.1 (rotamer), 39.5, 38.2, 36.8, 36.3 and 35.3 (rotamer), 33.4 and 33.1 (rotamer), 28.4, 23.3 and 22.8 (rotamer), 11.0 and 10.8 (rotamer); IR (neat) 2927, 1689, 1417, 1163, 1074 cm^{-1}; Anal Calcd for $C_{18}H_{27}NO_2$ C, 74.70; H, 9.40; N, 4.84. Found C, 74.36; H, 9.64; N, 4.76.

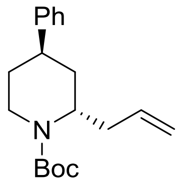

***N*-Boc-*trans*-2-allyl-4-phenylpiperidine (2ac).** Prepared from **1a** (55.4 mg, 0.150 mmol) and (allyl)tributyltin (115 μL, 0.375 mmol, 2.5 equiv) following the typical procedure. Isolated yield >99% (colorless oil, 43.7 mg, 0.144 mmol, purified by flash chromatography hexane/EtOAc = 30/1): ^{1}H NMR (400 MHz, $CDCl_3$) δ 7.35–7.28 (m, 2H), 7.24–7.16 (m, 3H), 5.87–5.70 (m, 1H), 5.10 (dd, *J* = 17.4, 1.4 Hz, 1H), 5.04 (d, *J* = 10.1 Hz, 1H), 4.53 and 4.36 (dddm, *J* = 6.4, 6.4, 6.4 Hz, 1H) (rotamer), 4.21 and 4.05 (dm, *J* =

12.8 Hz, 1H) (rotamer), 3.05–2.81 (m, 2H), 2.56–2.44 (m, 1H), 2.43–2.26 (m, 1H), 1.92–1.69 (m, 3H), 1.69–1.56 (m, 1H), 1.48 (s, 9H); ^{13}C NMR (100 MHz, $CDCl_3$) δ 155.0 and 154.9 (rotamer), 145.7, 135.4 and 135.3 (rotamer), 128.5, 126.8, 126.3, 117.0 and 116.7 (rotamer), 79.4 and 79.3 (rotamer), 50.9 and 49.6 (rotamer), 39.6 and 38.5 (rotamer), 36.6, 35.7, 35.0 and 34.6 (rotamer), 33.1 and 32.9 (rotamer), 28.4; IR (neat) 2982, 2066, 1705, 1418, 1167 cm^{-1}; HRMS (FAB^+) *m/z* calcd for $C_{19}H_{28}NO_2$ $[M + H]^+$ 302.2120, found 302.2124.

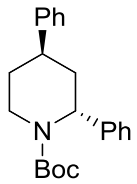

***N*-Boc-*trans*-2,4-diphenylpiperidine (2ad).** Prepared from **1a** (55.1 mg, 0.149 mmol) and PhMgBr (1.0 M in Et_2O, 300 μL, 0.300 mmol, 2.0 equiv) following the typical procedure. Isolated yield 87% (colorless solid, 44.0 mg, 0.130 mmol, purified with flash chromatography, hexane/EtOAc = 20/1); ^{1}H NMR (500 MHz, $CDCl_3$) δ 7.39 (t, *J* = 7.5 Hz, 2H), 7.35–7.26 (5H), 7.23–7.20 (m, 3H), 5.71 and 5.55 (brs, 1H) (rotamer), 4.29 and 4.15 (brs, 1H) (rotamer), 2.93 (brs, 1H), 2.73 (brs, 1H), 2.56 (brs, 1H), 2.09 (ddd, *J* = 13.5, 13.0, 3.3 Hz 1H), 1.74 (br, 2H), 1.62–1.35 (m, 9H); ^{13}C NMR (150 MHz, $CDCl_3$) δ 145.7, 128.7, 128.5, 126.7, 126.5, 126.4, 79.8, 53.9 and 52.5 (rotamer), 40.8 and 39.7 (rotamer), 37.1 and 36.9 (rotamer), 35.7 and 35.1 (rotamer), 33.4 and 33.0 (rotamer), 28.5 and 28.4 (rotamer); IR (neat) 2959, 1701, 1457, 1377, 724 cm^{-1}; Anal Calcd for $C_{22}H_{27}NO_2$ C, 78.30; H, 8.06; N, 4.15. Found C, 78.03; H, 8.39; N, 4.17, mp 68.1–68.7 °C.

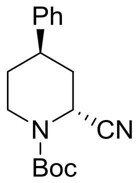

***N*-Boc-*trans*-2-cyano-4-phenylpiperidine (2ae).** Prepared from **1a** (111 mg, 0.301 mmol) and trimethylsilylcyanide (149 mg, 0.188 ml, 1.50 mmol) following the typical procedure. Isolated yield 99% (colorless oil, 85.2 mg, 0.299 mmol, purified by flash chromatography, hexane/EtOAc = 20/1); ^{1}H NMR (600 MHz, $CDCl_3$) δ 7.35–7.32 (m, 2H), 7.27–7.24 (m, 1H), 7.23–7.20 (m, 2H), 5.46 and 5.28 (s, 1H) (rotamer), 4.33–4.15 (m, 1H), 3.23–3.05 (m, 1H), 3.02 (dd, *J* = 12.6, 12.0 Hz, 1H), 2.12 and 2.10 (d, *J* = 13.2 Hz, 1H) (rotamer), 1.90 (ddd, *J* = 13.2, 13.2, 5.4 Hz, 1H), 1.65 (dddd, *J* = 12.6, 12.6, 4.8, 4.2 Hz, 1H), 1.51 (s, 9H); ^{13}C NMR (150 MHz, $CDCl_3$) δ 153.8, 143.6, 128.7, 126.9, 126.7, 81.7, 43.9, 41.8, 38.5, 35.4, 32.1, 28.2; IR (neat) 2977, 1698, 1393, 1024, 700 cm^{-1}; Anal Calcd for $C_{17}H_{22}N_2O_2$ C, 71.30; H, 7.74; N, 9.76. Found C, 71.61; H, 7.84; N, 9.78.

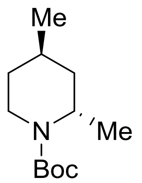

N-Boc-*trans*-2,4-dimethylpiperidine (2ba). Prepared from **1b** (46.4 mg, 0.151 mmol) and trimethylaluminium (1.08 M in toluene, 0.722 ml, 0.156 mmol) following the typical procedure. Isolated yield 86% (colorless oil, 27.5 mg, 0.129 mmol, purified with flash chromatography, hexane/EtOAc = 20/1); ^{1}H NMR (400 MHz, $CDCl_3$) δ 4.39 (brs, 1H), 3.92 (brs, 1H), 2.82 (t, *J* = 13.0 Hz, 1H), 1.80–1.63 (m, 1H), 1.52–1.41 (m, 12H), 1.26 (td, *J* = 12.8, 5.6 Hz, 1H), 1.11 (d, *J* = 6.8 Hz, 3H), 1.01 (qd, *J* = 13.3, 4.4 Hz, 1H), 0.89 (d, *J* = 6.4 Hz, 3H); ^{13}C NMR (100 MHz, $CDCl_3$, 60 °C) δ 154.6, 79.0, 46.5, 39.1, 38.8, 34.4, 28.6, 25.1, 22.2, 16.3; IR (neat) 2972, 2621, 2351, 1693, 1410 cm^{-1}; HRMS (ESI^+) calcd for $C_{12}H_{23}NO_2Na$ $[M + H]^+$ 236.1621, found 236.1625.

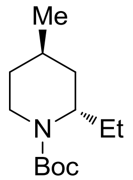

N-Boc-*trans*-2-ethyl-4-methylpiperidine (2bb). Prepared from **1b** (46.4 mg, 0.151 mmol) and diethylzinc (1.0 M in hexane, 0.450 ml, 0.450 mmol) following the typical procedure. Isolated yield 95% (colorless oil, 32.7 mg, 0.144 mmol, purified with flash chromatography, hexane/EtOAc = 20/1); ^{1}H NMR (600 MHz, $CDCl_3$) δ 4.20 and 4.07 (s,1H) (rotamer), 4.07 and 3.90 (s, 1H) (rotamer), 2.81–2.66 (m, 1H),1.71–1.63 (m, 2H), 1.60–1.49 (m, 2H), 1.44 (s, 9H), 1.41–1.34 (m, 1H), 1.19 (ddd, *J* = 13.2, 12.6, 5.4 Hz, 1H), 1.07–0.96 (m, 1H), 0.87 (d, *J* = 6.6 Hz, 3H), 0.84 (t, *J* = 7.2 Hz, 3H); ^{13}C NMR (150 MHz, $CDCl_3$) δ 155.0 and 154.6 (rotamer), 80.5 and 79.8 (rotamer), 79.8 and 79.5 (rotamer), 62.1 and 61.6 (rotamer), 39.1 and 39.0 (rotamer), 38.8 and 37.8 (rotamer), 33.8 and 33.6 (rotamer), 28.4 and 28.3 (rotamer), 25.0 and 24.9 (rotamer), 22.0, 15.2 and 15.1 (rotamer); IR (neat) 2927, 2872, 1700, 1404, 1160 cm^{-1}; HRMS (ESI^+) calcd for $C_{13}H_{26}NO_2$ $[M + H]^+$ 228.1964, found 228.1988.

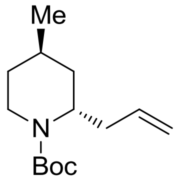

N-Boc-*trans*-2-allyl-4-methylpiperidine (2bc). Prepared from **1b** (46.3 mg, 0.151 mmol) and allyltributyltin (149.0 mg, 0.450 mmol) following the typical procedure. Isolated yield 95% (pale yellow oil, 34.4 mg, 0.144 mmol, purified by flash chromatography hexane/EtOAc 20:1); ^{1}H NMR (600 MHz, $CDCl_3$) δ 5.80–5.68 (m, 1H), 5.02 (d, *J* = 17.4 Hz,1H), 4.99 (d, *J* = 10.2 Hz,1H), 4.38 and 4.22 (s, 1H) (rotamer), 4.04 and 3.90 (d, *J* = 10.8 Hz, 1H) (rotamer), 2.83–2.70 (m, 1H), 2.36 (ddd, *J* = 14.4, 8.4, 7.2 Hz, 1H), 2.24–2.14 (m, 1H), 1.74–1.66 (m, 1H), 1.64–1.55 (m, 2H), 1.49 (s, 9H), 1.19 (ddd, *J* = 12.6, 7.2, 5.4 Hz, 1H), 1.08–0.98 (m, 1H), 0.89 (d, *J* = 6.6 Hz, 3H); ^{13}C NMR (150 MHz, $CDCl_3$) δ 155.0 135.6, 116.6 and 116.4 (rotamer), 79.1, 50.9 and 49.6 (rotamer), 39.5 and 38.3 (rotamer), 36.7 and 36.1 (rotamer), 35.3 and 34.9

(rotamer), 34.2 and 33.9 (rotamer), 28.4, 25.1, 22.2; IR (neat) 2980, 1680, 1363, 1178, 744 cm^{-1}; HRMS (FAB^+) calcd for $C_{14}H_{25}NO_2$ $[M + H]^+$ 240.1964, found 240.1975.

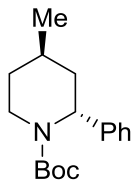

***N*-Boc-*trans*-4-methyl-2-phenylpiperidine (2bd).** Prepared from **1b** (46.3 mg, 0.151 mmol) and PhMgBr (1.0 M in Et_2O, 375 μL, 0.375 mmol) following the typical procedure. Isolated yield 93% (pale yellow oil, 38.5 mg, 0.140 mmol, purified by flash chromatography hexane/EtOAc = 20/1); ^{1}H NMR (400 MHz, $CDCl_3$) δ 7.34 (t, *J* = 7.7 Hz, 2H), 7.22 (t, *J* = 7.7 Hz, 1H), 7.21 (d, *J* = 7.7, 2H), 5.46 (brs, 1H), 4.09 (brs, 1H) 2.76 (td, *J* = 13.2, 2.4 Hz, 1H), 2.29 (d, *J* = 10.8 Hz, 1H), 1.56–1.50 (m, 3H), 1.47 (s, 9H), 1.04 (qd, *J* = 13.3, 4.8 Hz, 1H), 0.92 (d, *J* = 5.6 Hz, 3H); ^{13}C NMR (100 MHz, $CDCl_3$, 60 °C) δ 155.5, 140.9, 128.4, 126.5, 126.3, 79.4, 53.5, 40.3, 36.9, 34.2, 28.5, 25.7, 22.1; IR (neat) 2951, 2357, 1695, 1414, 1157 cm^{-1}; HRMS (ESI^+) calcd for $C_{14}H_{25}NO_2$ $[M + Na]^+$ 298.1778, found 298.1789.

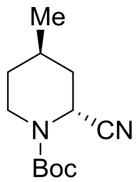

***N*-Boc-*trans*-2-cyano-4-methylpiperidine (2be).** Prepared from **1b** (46.1 mg, 0.150 mmol) and trimethylsilylcyanide (74.4 mg, 93μl, 0.750 mmol) following the typical procedure. Isolated yield 89% (colorless oil, 85.2 mg, 0.134 mmol, purified by flash chromatography hexane/EtOAc 20:1); ^{1}H NMR (400 MHz, $CDCl_3$) δ 5.32 and 5.16 (brs, 1H) (rotamer), 4.05 (brs, 1H), 2.97 (brs, 1H), 2.00–1.79 (m, 2H), 1.71 (brd, *J* = 12.8 Hz, 1H), 1.48 (s, 9H), 1.36 (td *J* = 13.6, 5.2 Hz, 1H), 1.10 (qd, *J* = 13.6, 5.2 Hz, 1H), 0.99 (d, *J* = 6.4 Hz, 3H); ^{13}C NMR (100 MHz, $CDCl_3$, 60 °C) δ 153.9, 117.8, 81.4, 44.4, 41.4, 36.5, 33.2, 28.3, 27.3, 21.3; IR (neat) 2957, 2874, 2255, 1697, 1395 cm^{-1}; Anal Calcd for $C_{17}H_{22}N_2O_2$ C, 71.30; H, 7.74; N, 9.76. Found C, 71.61; H, 7.84; N, 9.78.

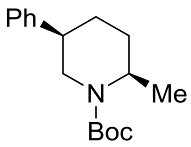

***N*-Boc-*cis*-2-methyl-5-phenylpiperidine (2ca).** Prepared from **1c** (55.4 mg, 0.150 mmol) and trimethylaluminium (1.08 M in toluene, 0.69 mL, 0.75 mmol) following the typical procedure. Isolated yield 89% (colorless solid, 36.8 mg, 0.134 mmol, purified by flash chromatography hexane/EtOAc = 30/1): ^{1}H NMR (400 MHz, $CDCl_3$) δ 7.32 (t, *J* = 7.3 Hz, 2H), 7.28–7.17 (m, 3H), 4.50 (s, 1H), 4.10 (d, *J* = 12.5 Hz, 1H), 2.89 (t, *J* = 12.5 Hz, 1H), 2.71–2.57 (m, 1H), 1.95–1.75 (m, 3H), 1.67 (dd, *J* = 8.9, 2.5 Hz, 1H), 1.51 (s,

9H), 1.23 (d, J = 6.9 Hz, 3H); ^{13}C NMR (100 MHz, $CDCl_3$) δ 154.9, 143.9, 128.5, 127.0, 126.6, 79.3, 45.5, 45.0, 43.0, 30.3, 28.6, 26.2, 15.7; IR (KBr) 2970, 1688, 1342, 1165, 1136 cm^{-1}; HRMS (ESI$^+$) calcd for $C_{17}H_{26}NO_2$ $[M + H]^+$ 276.1958, found 276.1967, mp 99.7–100.2 °C.

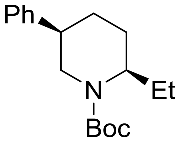

***N*-Boc-*cis*-2-ethyl-5-phenylpiperidine (2cb).** Prepared from **1c** (55.4 mg, 0.150 mmol) and diethyl zinc (1.0 M in hexane, 0.750 mL, 0.750 mmol) following the typical procedure. Isolated yield >99% (colorless solid, 43.7 mg, 0.144 mmol, purified by flash chromatography hexane/EtOAc = 30/1): ^{1}H NMR (400 MHz, $CDCl_3$, 60 °C) δ 7.33 (t, J = 7.1 Hz, 2H), 7.29–7.18 (m, 3H), 4.24 (d, J = 11.9 Hz, 1H), 4.17 (s, 1H), 2.82 (t, J = 11.9 Hz, 1H), 2.74–2.58 (m, 1H), 1.90–1.71 (m, 5H), 1.68–1.53 (m, 1H), 1.49 (s, 9H), 0.94 (t, J = 7.3 Hz, 3H); ^{13}C NMR (100 MHz, $CDCl_3$, 60 °C) δ 155.2, 143.9, 128.5, 128.2, 127.0, 126.5, 79.2, 51.6, 45.5, 42.9, 28.6, 26.5, 22.8, 10.8; IR (KBr) 3003, 2864, 1694, 1396, 1130 cm^{-1}; HRMS (ESI$^+$) calcd for $C_{18}H_{28}NO_2$ $[M + H]^+$ 290.2115, found 290.2125, mp 65.5–66.0 °C.

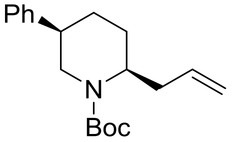

***N*-Boc-*cis*-2-allyl-5-phenylpiperidine (2cc).** Prepared from **1c** (55.4 mg, 0.150 mmol) and allyltributylstannane (1.0 M in Et_2O, 375 μL, 0.375 mmol, 2.5 equiv) following the typical procedure. Isolated yield 97% (pale yellow oil, 43.7 mg, 0.145 mmol, purified by flash chromatography hexane/EtOAc = 30/1): ^{1}H NMR (400 MHz, $CDCl_3$, 60 °C) δ 7.33 (t, J = 7.5 Hz, 2H), 7.28–7.20 (m, 4H), 5.90–5.72 (m, 1H), 5.09 (dd, J = 16.0, 1.4 Hz, 1H), 5.06 (dd, J = 10.1, 1.4 Hz, 1H), 4.39 (brs, 1H), 4.16 (brs, 1H), 2.84 (t, J = 12.1 Hz, 1H), 2.74–2.58 (m, 1H), 2.56–2.42 (m, 1H), 2.42–2.24 (m, 1H), 1.88–1.76 (m, 4H), 1.48 (s, 9H); ^{13}C NMR (100 MHz, $CDCl_3$, 60 °C) δ 155.0, 143.8, 135.5, 128.5, 127.1, 126.6, 116.7, 79.4, 49.7, 45.3, 42.7, 34.5, 28.6, 27.8, 26.3; IR (neat) 3003, 2934, 2864, 1682, 1173 cm^{-1}; HRMS (ESI$^+$) calcd for $C_{19}H_{28}NO_2$ $[M + H]^+$ 302.2115, found 302.2143.

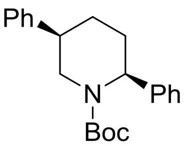

***N*-Boc-*cis*-2,5-diphenylpiperidine (2cd).** Prepared from **1c** (55.4 mg, 0.150 mmol) and PhMgBr (1.0 M in Et_2O, 375 μL, 0.375 mmol, 2.5 equiv) following the typical procedure. Isolated yield 97% (colorless solid, 48.9 mg, 0.145 mmol, purified by flash chromatography hexane/EtOAc = 30/1): ^{1}H NMR (400 MHz, $CDCl_3$, 60 °C) δ 7.37 (t, J = 7.4 Hz, 2H), 7.31–7.20 (m, 6H), 7.17 (t, J = 7.4 Hz, 1H), 7.11 (d, J = 7.4 Hz, 2H), 5.52 (s, 1H), 4.21 (d, J = 6.9 Hz, 1H), 2.88–2.70 (m, 2H), 2.49 (dd, J = 14.2, 4.8 Hz, 1H), 2.19–1.97 (dddd, J =

14.2, 10.8, 4.0, 3.7 Hz, 1H), 1.87 (dd, J = 13.0, 3.7 Hz, 1H), 1.72 (m, 1H), 1.45 (s, 9H); ^{13}C NMR (100 MHz, $CDCl_3$, 60 °C) δ 155.6, 143.6, 140.3, 128.7, 128.5 (2), 127.0, 126.6 (2), 79.8, 52.7, 46.5, 42.8, 28.6, 28.2, 26.9; IR (KBr) 2976, 1688, 1452, 1366, 1146 cm^{-1}; HRMS (ESI$^+$) calcd for $C_{22}H_{28}NO_2$ [M + H]$^+$ 338.2115, found 338.2123, mp 107.5–108.0 °C.

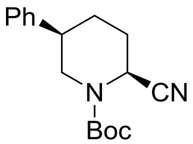

***N*-(*tert*-Butyloxycarbonyl)-*cis*-2-cyano-5-phenylpiperidine (2ce).** Prepared from **1c** (55.4 mg, 0.150 mmol) and trimethylsilylcyanide (74.4 mg, 0.093 ml, 0.750 mmol) following the typical procedure. Isolated yield 96% (colorless solid, 41.2 mg, 0.144 mmol, purified by flash chromatography hexane/EtOAc = 30/1): ^{1}H NMR (400 MHz, $CDCl_3$, 60 °C) δ 7.34 (t, J = 7.3 Hz, 2H), 7.29–7.21 (m, 3H), 5.32 (s, 1H), 4.19 (d, J = 12.7 Hz, 1H), 3.03 (t, J = 12.7 Hz, 1H), 2.75–2.60 (m, 1H), 2.13–1.97 (m, 3H), 1.97–1.84 (m, 1H), 1.50 (s, 9H); ^{13}C NMR (100 MHz, $CDCl_3$, 60 °C) δ 153.8, 142.2, 128.8, 127.1 (2), 117.5, 81.7, 47.5, 43.8, 42.0, 28.7, 28.3 27.8; IR (KBr) 2976, 2862, 1694, 1366, 1155 cm^{-1}; HRMS (ESI$^+$) calcd for $C_{17}H_{22}N_2O_2$ [M + H]$^+$ 287.1754, found 287.1754, 140.9–141.4 °C.

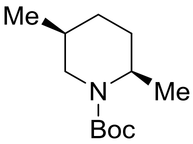

***N*-Boc-*cis*-2,5-dimethylpiperidine (2da).** Prepared from **2d** (46.1 mg, 0.150 mmol) and trimethylaluminum (1.08 M in toluene, 0.69 mL, 0.75 mmol) following the typical procedure. Isolated yield 73% (colorless oil, 23.4 mg, 0.110 mmol, purified by flash chromatography hexane/EtOAc = 30/1); ^{1}H NMR (400 MHz, $CDCl_3$, 60 °C) δ 4.17 (dd, J = 6.0 Hz, 1H), 3.87 (brd, J = 12.8 Hz, 1H), 2.40 (t, J = 12.4 Hz, 1H), 1.59–1.43 (m, 12H), 1.33–1.16 (m, 1H), 1.10 (d, J = 6.4 Hz, 3H), 0.89 (d, J = 6.4 Hz, 3H); ^{13}C NMR (100 MHz, $CDCl_3$, 60 °C) δ 155.0, 79.0, 45.7, 45.5, 31.4, 30.2, 28.6, 27.8, 19.2, 15.6; IR (neat) 2934, 2864, 1682, 1416, 1146 cm^{-1}; HRMS (ESI$^+$) calcd for $C_{12}H_{23}NO_2Na$ [M + Na]$^+$ 236.1621, found 236.1631.

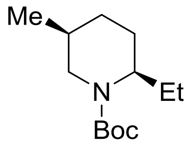

***N*-Boc-*cis*-2-ethyl-5-methylpiperidine (2db).** Prepared from **2d** (46.1 mg, 0.150 mmol) and diethyl zinc (1.0 M in hexane, 0.750 mL, 0.750 mmol) following the typical procedure. Isolated yield 82% (colorless oil, 27.9 mg, 0.123 mmol, purified by flash chromatography hexane/EtOAc = 30/1); ^{1}H NMR (400 MHz, $CDCl_3$, 60 °C) δ 4.15 and 4.02 (s, 1H) (rotamer), 3.96 and 3.77 (d, *J* = 12.4 Hz, 1H) (rotamer), 2.34 and 2.26 (t, *J* = 12.4 Hz, 1H) (rotamer), 1.43 (s, 9H), 1.84–1.10(m, 7H), 0.85–0.80 (m, 6H); ^{13}C NMR (100 MHz, $CDCl_3$, 60 °C) δ 155.3, 78.9, 51.7 and 50.5 (rotamer), 46.3 and 44.7 (rotamer), 31.2, 28.2, 27.9, 22.7 and 20.2 (rotamer), 19.3, 10.9; IR (neat) 2965, 1692, 1418, 1152, 880 cm^{-1}, HRMS (ESI$^+$) calcd for $C_{13}H_{25}NO_2Na$ [M + Na]$^+$ 250.1778, found 250.1784.

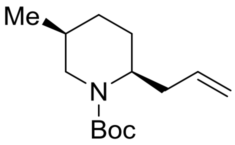

***N*-Boc-*cis*-2-allyl-5-methylpiperidine (2dc).** Prepared from **1d** (46.1 mg, 0.150 mmol) and allyltributyltin (230 μL, 248.0 mg, 0.750 mmol) following the typical procedure. Isolated yield 90% (pale yellow oil, 32.4 mg, 0.135 mmol, purified by flash chromatography hexane/EtOAc = 30/1); ^{1}H NMR (400 MHz, $CDCl_3$, 60 °C) δ 5.80–5.64 (m, 1H), 5.02 (d, *J* = 17.6 Hz, 1H), 4.98 (d, *J* = 10.4 Hz, 1H), 4.35 and 4.20 (s, 1H) (rotamer), 3.96 and 3.79 (d, *J* = 11.6 Hz, 1H) (rotamer), 2.42–2.26 (m, 2H), 2.26–2.10 (m, 1H), 1.61–1.60 (m, 4H), 1.43 (s, 9H), 1.24–1.12 (m, 1H), 0.87 (d, *J* = 6.4 Hz, 3H); ^{13}C NMR (100 MHz, $CDCl_3$, 60 °C) δ 155.1, 135.6, 116.6, 79.1, 50.0 and 48.6 (rotamer), 46.4 and 45.2 (rotamer), 34.5, 34.1, 31.1, 28.4, 27.7, 19.3; IR (neat) 2927, 1693, 1411, 1151, 983 cm^{-1}; HRMS (FAB$^+$) calcd for $C_{14}H_{25}NO_2Na$ [M + Na]$^+$ 262.1786, found 262.1783.

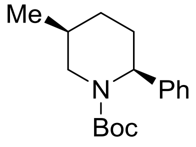

***N*-Boc-*cis*-5-methy-2-phenylpiperidine (2dd).** Prepared from **1d** (55.4 mg, 0.150 mmol) and PhMgBr (1.0 M in Et_2O, 375 μL, 0.375 mmol, 2.5 equiv) following the typical procedure. Isolated yield 83% (colorless oil, 34.1 mg, 0.124 mmol, purified by flash chromatography hexane/EtOAc = 30/1); ^{1}H NMR (400 MHz, $CDCl_3$, 60 °C) δ 7.28–7.19 (m, 2H), 7.19–7.06 (m, 3H), 5.32 (brs, 1H), 3.91 (brd, *J* = 13.2 Hz, 1H), 2.36–2.14 (m, 2H), 1.84 (dddd, *J* = 13.7, 13.7, 5.5, 3.4 Hz, 1H), 1.65–1.45 (m, 2H), 1.45–1.28 (m, 9H), 1.11–0.90 (m, 1H), 0.72 (d, *J* = 6.4 Hz, 3H); ^{13}C NMR (100 MHz, $CDCl_3$, 60 °C) δ 155.6, 140.6, 128.5, 126.6, 126.3, 79.5, 52.8, 47.2, 31.3, 28.5, 28.2, 19.1; IR (neat) 2976, 1688, 1452, 1366, 1146 cm^{-1}; HRMS (ESI$^+$) calcd for $C_{17}H_{25}NO_2Na$ [M + Na]$^+$ 298.1778, found 298.1782.

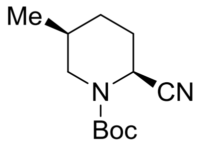

***N*-Boc-*cis*-2-cyano-5-methylpiperidine (2de).** Prepared from **1d** (46.1 mg, 0.150 mmol) and trimethylsilylcyanide (74.4 mg, 0.093 ml, 0.750 mmol) following the typical procedure. Isolated yield 98% (colorless oil, 32.9 mg, 0.147 mmol, purified by flash chromatography hexane/EtOAc = 10/1); ^{1}H NMR (400 MHz, $CDCl_3$, 60 °C) δ 5.20 (s, 1H), 3.98 (d, J = 12.7 Hz, 1H), 2.55 (dd, J = 12.7 Hz, 1H), 1.98–1.88 (m, 1H), 1.86–1.65 (m, 2H), 1.60–1.30 (m, 11H), 0.95 (d, J = 6.8 Hz, 3H); ^{13}C NMR (100 MHz, $CDCl_3$, 60 °C) δ 153.8, 117.6, 81.4, 48.2, 43.8, 30.6, 29.1, 28.7, 28.3, 18.8; IR (neat) 3394, 2978, 2234, 1699, 1395 cm^{-1}; HRMS (FAB$^+$) calcd for $C_{12}H_{21}N_2O_2Na$ $[M + Na]^+$ 247.1422, found 247.1426.

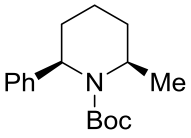

***N*-Boc-*cis*-2-methyl-6-phenylpiperidine (2ea).** Prepared from **1e** (55.8 mg, 0.151 mmol) and trimethylaluminium (1.08 M in hexane, 347 μL, 0.375 mmol, 2.5 equiv) following the typical procedure. Isolated yield 40% (pale yellow oil, *cis*/*trans* = 63/37, 30.8 mg, 0.102 mmol, purified by flash chromatography hexane/EtOAc = 30/1): ^{1}H NMR (400 MHz, $CDCl_3$, 60 °C) δ 7.38–7.25 (m, 6H), 7.24–7.09 (m, 6H), 5.38 (s, 1H, *trans*) and 4.98 (t, J = 4.8 Hz, 1H, *cis*), 4.48 (t, J = 5.3 Hz, 1H, *trans*) and 4.29–4.11 (m, 1H, *cis*), 2.36 (d, J = 10.5 Hz, 1H), 2.24–2.08 (m, 1H), 2.07–1.93 (m, 1H), 1.92–1.70 (m, 4H), 1.70–1.56 (m, 2H), 1.56–1.37 (m, 16H), 1.37–1.20 (m, 17H), 0.90 (d, J = 6.9 Hz, 4H); ^{13}C NMR (100 MHz, $CDCl_3$, 60 °C) δ 156.0, 144.7 and 143.7, 128.1 and 128.0, 126.6, 126.2 and 126.0, 125.7, 79.4 and 79.3, 55.0 and 52.1, 47.8 and 46.6, 30.5, 28.5 and 28.4, 28.5 and 27.5, 20.9 and 20.6, 15.5 and 14.7; IR (neat) 2972, 1682, 1454, 1366, 1173 cm^{-1}; HRMS (ESI$^+$) calcd for $C_{17}H_{26}NO_2$ $[M + H]^+$ 276.1958, found 276.1971.

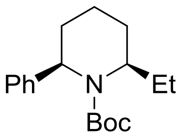

***N*-Boc-*cis*-2-ethyl-6-phenylpiperidine (2eb).** Prepared from **1e** (56.1 mg, 0.152 mmol) and diethylzinc (1.0 M in heptane, 354 μL, 0.375 mmol, 2.5 equiv) following the typical procedure. Isolated yield 90% (colorless oil, *cis*/*trans* = 76/24, 31.4 mg, 0.131 mmol, purified by flash chromatography hexane/EtOAc = 30/1): ^{1}H NMR (400 MHz, $CDCl_3$, 60 °C) δ 7.40 (d, J = 7.3 Hz, 2H), 7.35-7.23 (m, 14H), 7.22–7.14 (m, 4H), 5.44 (d, J = 4.1 Hz, 1H, *trans*), 5.01 (t, J = 4.6 Hz, 3H, *cis*), 4.26–4.13 (m, 1H, *trans*), 3.95–3.83 (m, 3H, *cis*), 2.35 (d, J = 14.2 Hz, 1H), 2.21–1.99 (m, 6H), 1.96-1.80 (m, 4H), 1.80–1.53 (m, 16H), 1.53-1.39 (m, 14H), 1.39-1.22 (m, 27H), 1.19-1.01 (m, 1H), 0.97 (t, J = 6.9 Hz, 9H), 0.73 (t, J = 7.3 Hz, 3H); ^{13}C NMR (100 MHz, $CDCl_3$, 60 °C) δ 156.3 and 156.1, 144.3 and 143.7, 128.1 and 128.0, 126.8 and 126.3, 126.0 and 125.8, 79.4 and 79.2, 55.1 and 54.1, 53.1 and 51.8, 28.5 and 28.0, 28.3, 27.7 and 27.3, 23.9, 15.8 and 15.0, 11.6 and 11.4; IR (neat): 2976, 2862, 1694, 1366, 1155 cm^{-1}; HRMS (ESI) m/z calcd for $C_{18}H_{28}NO_2$ $[M + H]^+$ 290.2115, found 290.2129.

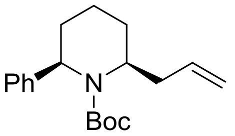

***N*-Boc-*cis*-2-allyl-6-phenylpiperidine (2ec).** Prepared from **1e** (56.0 mg, 0.15 mmol) and (allyl)tributyltin (115 μL, 0.375 mmol, 2.5 equiv) following the typical procedure. Isolated yield 94% (pale yellow oil, *cis*/*trans* = 72/28, 42.5 mg, 0.141 mmol, purified by flash chromatography hexane/EtOAc = 30/1): ^{1}H NMR (400 MHz, $CDCl_3$, 60 °C) δ 7.35 (d, *J* = 7.3 Hz, 2H), 7.31–7.23 (m, 8H), 7.23–7.07 (m, 8H), 5.92–5.71 (m, 2H), 5.62 (d, *J* = 6.9 Hz, 1H), 5.41 (d, *J* = 3.4 Hz, 1H), 5.18–4.94 (m, 7H), 4.89 (d, *J* = 10.5 Hz, 1H), 4.79 (d, *J* = 16.9 Hz, 1H), 4.41–4.26 (m, 1H), 4.14–3.96 (m, 2H), 2.71–2.52 (m, 2H), 2.44–2.22 (m, 3H), 2.21–1.97 (m, 5H), 1.92–1.50 (m, 13H), 1.50–1.37 (m, 14H), 1.32 (s, 20H); ^{13}C NMR (100 MHz, $CDCl_3$, 60 °C) δ 156.0 and 155.9, 144.3 and 143.4, 136.4 and 136.1, 128.1 and 128.1, 126.8 and 126.4, 126.0 and 125.7, 79.6 and 79.4, 55.0 and 51.1, 52.1 and 51.8, 39.1 and 38.8, 28.5 and 28.3, 28.2, 27.3 and 27.2, 23.6, 15.5 and 14.6; IR (neat) 2974, 1693, 1416, 1366, 1168 cm^{-1}; HRMS (ESI^+) calcd for $C_{19}H_{28}NO_2$ $[M + H]^+$ 302.2115, found 302.2143.

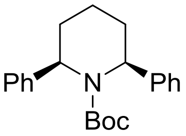

***N*-Boc *cis*-2,6-diphenylpiperidine (2ed).** Prepared from **1e** (55.2 mg, 0.149 mmol) and phenylmagnesium bromide (3.0 M in Et_2O, 340 μL, 0.375 mmol, 2.5 equiv) following the typical procedure. Isolated yield 56% (colorless solid, 29.2 mg, 0.077 mmol, purified by flash chromatography hexane/EtOAc = 30/1): ^{1}H NMR (400 MHz, $CDCl_3$, 60 °C) δ 7.32–7.22 (m, 7H), 7.22–7.10 (m, 3H), 5.22 (s, 2H), 2.19–2.03 (m, 2H), 2.03–1.88 (m, 2H), 1.47–1.32 (m, 3H), 1.13 (s, 9H); ^{13}C NMR (100 MHz, $CDCl_3$, 60 °C) δ 156.2, 144.9, 128.3, 126.1, 125.8, 79.6, 55.6, 28.3, 28.1, 14.7; IR (KBr) 2955, 2941, 1450, 1364, 1173 cm^{-1}; HRMS (ESI^+) calcd for $C_{22}H_{28}NO_2$ $[M + H]^+$ 338.2115, found 338.2115, mp 71.4–71.9 °C.

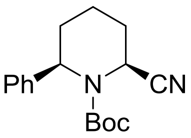

***N*-Boc-*cis*-2-cyano-6-phenylpiperidine (2ee).** Prepared from **1e** (55.2 mg, 0.149 mmol) and trimethylsilyl cyanide (93 μL, 0.75 mmol, 5.0 equiv) following the typical procedure. Isolated yield 85% (colorless solid, *cis*/*trans* = 64/36, 36.5 mg, 0.127 mmol, purified by flash chromatography hexane/EtOAc = 30/1): ^{1}H NMR (400 MHz, $CDCl_3$) δ 7.32–7.22 (m, 4H), 7.22–7.14 (m, 1H), 5.31–5.21 (m, 2H), 2.33 (dd, *J* = 10.7, 3.4 Hz, 1H), 1.94 (dt, *J* = 9.1, 2.1 Hz, 1H), 1.88–1.71 (m, 3H), 1.63 (d, *J* = 3.2 Hz, 1H), 1.35 (s, 9H); ^{13}C NMR (100 MHz, $CDCl_3$) δ 154.6, 139.9, 128.4, 127.2, 126.6, 118.8, 81.8, 53.4, 40.8, 28.6, 28.2, 27.4, 16.1; IR (KBr) 2963, 1678, 1393, 1368, 1163 cm^{-1}; HRMS (ESI^+) calcd for $C_{17}H_{23}N_2O_2$ $[M + H]^+$ 287.1754, found 287.1768, mp 88.8–89.3 °C.

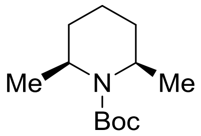

***N*-Boc-*cis*-2,6-dimethylpiperidine (2fa).** Prepared from **1f** (46.1 mg, 0.150 mmol) and trimethylalminium (1.08 M in hexane, 347 μL, 0.375 mmol, 2.5 equiv) following the typical procedure. Isolated yield 49% (colorless oil, 15.8 mg, 0.074 mmol, purified by flash chromatography hexane/EtOAc = 30/1): ^{1}H NMR (400 MHz, $CDCl_3$) δ 4.34–4.15(m, 2H),1.85–1.65 (m, 1H), 1.65–1.48 (m, 3H), 1.45 (s, 9H), 1.38–1.23, (m, 1H), 1.16 (d, *J* = 6.8 Hz, 6H), 0.95–0.85 (m, 1H); ^{13}C NMR (100 MHz, $CDCl_3$, 60 °C) δ 155.3, 78.9, 45.8, 30.3, 28.6, 20.8, 14.0; IR (neat) 2960, 2932, 1728, 1275, 1123 cm^{-1}; HRMS (ESI$^+$) calcd for $C_{12}H_{23}NO_2Na$ [M + Na]$^+$ 236.1621 found 236.1631.

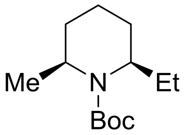

***N*-Boc-*cis*-2-ethyl-6-methylpiperidine (2fb).** Prepared from **1f** (46.1 mg, 0.150 mmol) and diethyl zinc (1.0 M in hexane, 230 μL, 0.750 mmol) following the typical procedure. Isolated yield 46% (colorless oil, 15.7 mg, 0.069 mmol, purified by flash chromatography hexane/EtOAc = 30/1); ^{1}H NMR (400 MHz, $CDCl_3$, 60 °C) δ 4.35–4.18 (m,1H), 4.03–3.88 (m, 1H), 1.73–1.59 (m, 3H), 1.59–1.48 (m, 4H), 1.45 (s, 9H), 1.43–1.36 (m, 1H), 1.14 (d, *J* = 6.8Hz, 3H), 0.88 (t, *J* = 7.6 Hz, 3H); ^{13}C NMR (100 MHz, $CDCl_3$) δ 156.5, 78.8, 52.1, 45.8, 30.5, 28.6, 28.1, 27.2, 20.5, 14.4, 11.9; IR (neat) 2965, 2932, 1694, 1368, 1173 cm^{-1}; HRMS (FAB$^+$) calcd for $C_{13}H_{26}NO_2$ [M + H$^+$] 228.1958, found 228.1958.

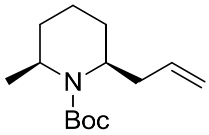

***N*-Boc-*cis*-2-allyl-6-methylpiperidine (2fc).** Prepared from **1f** (46.1 mg, 0.150 mmol) and (allyl)tributyltin (230 μL,248.0 mg, 0.750 mmol) following the typical procedure. Isolated yield 87% (pale yellow oil, 31.4 mg, 0.131 mmol, purified by flash chromatography hexane/EtOAc = 30/1); ^{1}H NMR (400 MHz, $CDCl_3$) δ 5.74 (ddt, *J* = 17.4, 10.2, 7.2 Hz, 1H), 5.02 (dd, *J* = 17.4, 1.2 Hz, 1H), 4.99 (dd, *J* = 1.2 Hz, 1H), 4.28–4.24 (m, 1H), 4.12–4.07 (m, 1H), 2.30–2.24 (m, 2H), 1.44 (s, 9H), 1.70–1.39 (m, 6H), 1.15 (d, *J* = 7.6 Hz, 3H); ^{13}C NMR (100 MHz, $CDCl_3$) δ 155.3, 136.8, 116.6, 79.1, 50.0, 45.6, 39.6, 30.2, 28.6, 26.7, 20.5, 13.9; IR (neat) 3075, 2936, 1688, 1458, 1080 cm^{-1}; HRMS (FAB$^+$) calcd for $C_{14}H_{25}NO_2Na$ [M + Na]$^+$ 262.1756, found 262.1729.

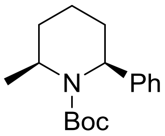

***N*-Boc-*cis*-6-methy-2-phenylpiperidine (2fd).** Prepared from **1f** (55.4 mg, 0.150 mmol) and PhMgBr (1.0 M in Et_2O, 375 μL, 0.375 mmol, 2.5 equiv) following the typical procedure. Isolated yield 87% (pale yellow oil, 35.9 mg, 0.131 mmol, purified by flash chromatography hexane/EtOAc = 30/1): ^{1}H NMR (400 MHz, $CDCl_3$) δ:7.32–7.19 (m, 4H), 7.19–7.09 (m, 1H), 5.39 and 5.31 (brs, 1H) (rotamer), 4.45 and 4.43 (dq, *J* = 6.9, 2.2 Hz, 1H) (rotamer), 2.41–2.28 (m, 1H), 1.84–1.64 (m, 3H), 1.54–1.36 (m, 11H), 0.79 (d, *J* = 7.2 Hz, 3H); ^{13}C NMR (100 MHz, $CDCl_3$, 60 °C) δ155.9, 143.6, 128.0, 126.6, 126.2, 79.4, 52.1, 46.6, 30.5, 28.5, 27.5, 20.9, 15.4; IR (neat) 2972, 1682, 1404, 1175, 1069 cm^{-1}; HRMS (ESI+) calcd for $C_{17}H_{26}NO_2$ $[M + H]^+$ 276.1958, found 276.1958.

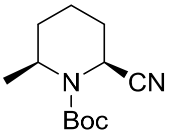

***N*-Boc-*cis*-2-cyano-6-methylpiperidine (2fe).** Prepared from **1f** (55.4 mg, 0.150 mmol) and trimethylsilylcyanide (74.4 mg, 0.093 ml, 0.750 mmol) following the typical procedure. Isolated yield 90% (colorless oil, 30.2 mg, 0.135 mmol, purified by flash chromatography hexane/EtOAc = 30/1): ^{1}H NMR (400 MHz, $CDCl_3$) δ 5.05(d, *J* = 4.4 Hz, 1H), 4.39–4.27 (m, 1H), 2.06–1.96 (m, 1H), 1.96–1.82 (m, 1H), 1.74–1.59 (m, 4H), 1.48 (s, 9H), 1.33 (d, *J* = 6.8 Hz, 3H); ^{13}C NMR (100 MHz, $CDCl_3$, 60 °C) δ 154.8, 120.2, 81.3, 47.0, 40.7, 29.4, 28.3, 28.0,17.3, 15.3; IR (neat) 2976, 2943, 1697, 1368, 1173 cm^{-1}; HRMS (ESI+) calcd for $C_{12}H_{21}N_2O_2$ $[M + H]^+$ 225.1598, found 225.1608.

NMR analysis of *N*-acyliminium ion generated by the indirect cation pool method.

Typical procedure of NMR analysis of *N*-acyliminium ions. Analogous as described in our previous report.[6] The anodic oxidation was carried out in a divided cell equipped with a carbon felt anode and a platinum plate cathode. In the anodic chamber was placed a solution of ArSSAr (Ar = *p*-FC_6H_4) (75 μL, 0.401 mmol) in 4.0 mL of 0.3 M Bu_4NBF_4/CH_2Cl_2. In the cathodic chamber were placed trifluoromethanesulfonic acid (24 μL, 0.273 mmol) and 0.3 M Bu_4NBF_4 in CH_2Cl_2 (4.0 mL). The constant current electrolysis (8.0 mA) was carried out at −78 °C with magnetic stirring until 0.67 F/mol of electricity was consumed. To the solution of the cation precursor (13.0 mg, 0.0352 mmol) in CD_2Cl_2 (0.35 mL) which was placed in a 5 mm ∅ NMR tube equipped with a septum cap was transferred the reaction mixture of the anodic chamber (0.35 mL) under Ar atmosphere at −78 °C. After the mixture was quickly shaken at −78 °C, the NMR measurement was carried out at −60 °C. Exclusively ion **C1** was measured at −80 °C

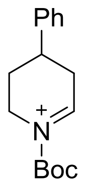

NMR analysis of 4-Ph-*N*-acyliminium ion (C1). ^{1}H NMR (600 MHz, CD_2Cl_2 δ 9.46 (s, 1H), 4.32 (d, *J* = 11.3 Hz, 1H), 3.86 (t, *J* = 12.7 Hz, 1H), 3.47 (d, *J* = 20.9 Hz, 1H), 3.22–3.08 (m, 2H), 2.27–2.10 (m, 2H); ^{13}C NMR (150 MHz, CD_2Cl_2) δ 186.4, 144.8, 141.5, 91.1, 47.6, 36.4, 32.4, 26.7, 26.3; Other signals could not be assigned because of overlap with the signals of Bu_4NBF_4 used as the electrolyte or the signals of ArSSAr (Ar = *p*-FC_6H_4).

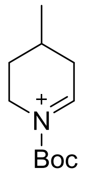

NMR analysis of 4-Me-*N*-acyliminium ion (C2). ^{1}H NMR (400 MHz, CD_2Cl_2) δ 9.32 (s, 1H), 4.10 (d, *J* = 13.2 Hz, 1H), 3.66 (t, *J* = 13.2 Hz, 1H), 3.21 (d, *J* = 22.8 Hz, 1H), 2.54 (dd, *J* = 22.8, 8.5 Hz, 1H), 1.99–1.84 (m, 2H), 0.94 (d, *J* = 6.4 Hz, 3H); ^{13}C NMR (100 MHz, CD_2Cl_2) δ 186.6, 144.7, 91.2, 53.8, 47.1, 36.4, 27.0, 26.0, 21.5, 19.3; Other signals could not be assigned because of overlap with the signals of Bu_4NBF_4 used as the electrolyte or the signals of ArSSAr (Ar = *p*-FC_6H_4).

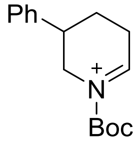

NMR analysis of 5-Ph-*N*-acyliminium ion (C3). ^{1}H NMR (400 MHz, CD_2Cl_2) δ 9.49 (s, 1H), 4.25 (d, *J* = 12.2 Hz, 1H), 3.73 (t, *J* = 12.2 Hz, 1H), 3.41 (d, *J* = 23.3 Hz, 1H), 3.25–3.09 (m, 2H), 2.07 (q, *J* = 11.1 Hz, 1H), 2.00–1.89 (m, 1H); ^{13}C NMR (100 MHz, CD_2Cl_2) δ 186.4, 144.6, 91.1, 53.8, 51.5, 36.3, 30.3, 26.1, 21.2; Other signals could not be assigned because of overlap with the signals of Bu_4NBF_4 used as the electrolyte or the signals of ArSSAr (Ar = *p*-FC_6H_4).

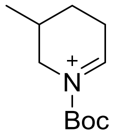

NMR analysis of 5-Me-*N*-acyliminium ion (C4). ^{1}H NMR (400 MHz, CD_2Cl_2) δ 9.36 (s, 1H), 4.05 (d, *J* = 11.4 Hz, 1H), 3.27–3.14 (m, 2H), 1.94 (m, 1H), 1.74 (m, 1H), 0.96 (d, *J* = 6.4 Hz, 3H); ^{13}C NMR (100 MHz, CD_2Cl_2) δ 187.1, 144.7, 91.2, 52.0, 29.7, 26.2, 25.5, 22.3, 17.4; Other signals could not be assigned because of overlap with the signals of Bu_4NBF_4 used as the electrolyte or the signals of ArSSAr (Ar = *p*-FC_6H_4).

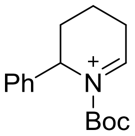

NMR analysis of 6-Ph-*N*-acyliminium ion (C5). ^{1}H NMR (400 MHz, CD_2Cl_2) δ 9.72 (s, 1H), 3.42 (d, *J* = 23.6 Hz, 1H), 3.25 (dt, *J* = 23.6, 7.0 Hz, 1H), 2.32 (t, *J* = 12.9 Hz, 1H), 1.99 (d, *J* = 12.9 Hz, 1H), 1.83–1.70 (m, 1H); ^{13}C NMR (100 MHz, CD_2Cl_2) δ 189.8, 144.5, 91.6, 60.8, 29.7, 27.9, 25.8, 11.1; Other signals could not be assigned because of overlap with the signals of Bu_4NBF_4 used as the electrolyte or the signals of ArSSAr (Ar = *p*-FC_6H_4).

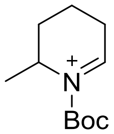

NMR analysis of 6-Me-*N*-acyliminium ion (C6). ^{1}H NMR (400 MHz, CD_2Cl_2) δ 9.44 (s, 1H), 4.58 (s, 1H), 3.28 (d, *J* = 23.8 Hz, 1H), 3.09 (dt, *J* = 23.8, 8.8 Hz, 1H), 1.99–1.82 (m, 2H), 1.82–1.67 (m, 2H), 1.34 (d, *J* = 6.7 Hz, 3H); ^{13}C NMR (100 MHz, CD_2Cl_2) δ 188.2, 144.1, 91.5, 53.8, 29.4, 26.0, 24.8, 18.4, 10.4; Other signals could not be assigned because of overlap with the signals of Bu_4NBF_4 used as the electrolyte or the signals of ArSSAr (Ar = *p*-FC_6H_4).

2. X-ray crystallography

Details of the crystal data and a summary of the intensity data collection parameters for cation precursors **1a**, **1b**, and **1d–f** are listed in Table S1–S5 and Figure S1–S5. X-ray single crystal analysis was conducted using a Rigaku VariMax with Saturn. Graphite-monochromated MoKα radiation (λ = 0.71075 Å) was used. The structures were solved by direct methods with SIR2014[7] or SHELXT[8] and refined by full-matrix least-squares techniques against F^2 (SHELXL).[8] The non-hydrogen atoms were refined anisotropically. Hydrogen atoms were placed using AFIX instructions. In the subsequent refinement, the function $\Sigma w(F_o^2 - F_c^2)^2$ was minimized, where $|F_o|$ and $|F_c|$ are the observed and calculated structure factor amplitudes, respectively. The agreement indices are defined as $R_1 = \Sigma(||F_o| - |F_c||)/\Sigma|F_o|$ and $wR_2 = [\Sigma w(F_o^2 - F_c^2)^2/\Sigma(wF_o^4)]^{1/2}$. All calculations were performed by using Yadokari-XG[9] and illustrations were drawn by using ORTEP-3[10] and colored by Adobe Illustrator. These cif data can also be obtained free of charge from The Cambridge Crystallographic Data Centre at www.ccdc.cam.ac.uk/data_request/cif (**1a**: CCDC-1813600, **1b**: CCDC-1813582, **1d**: CCDC-1813594, **1e**: CCDC-1813595, **1f**: CCDC-1813596).

Table S1. Crystal data and structure refinement for **1a**

Empirical formula	$C_{22}H_{27}NO_2S$	
Formula weight	369.50	
Temperature	100 K	
Wavelength	0.71075 Å	
Crystal system	Triclinic	
Space group	P-1 (#2)	
Unit cell dimensions	a = 6.334(6) Å	α = 89.62(3)°.
	b = 9.919(8) Å	β = 88.65(3)°.
	c = 16.374(16) Å	γ = 75.52(2)°.
Volume	995.8(16) Å^3	
Z	2	
Density (calculated)	1.232 Mg/m^3	
Absorption coefficient	0.178 mm^{-1}	
F(000)	396	
Crystal size	0.09 x 0.08 x 0.05 mm^3	
Theta range for data collection	1.244 to 31.404°.	
Index ranges	-9<=h<=8, -14<=k<=14, -22<=l<=23	
Reflections collected	13987	
Independent reflections	5818 [R(int) = 0.0905]	
Completeness to theta = 25.242°	99.7 %	
Absorption correction	Semi-empirical from equivalents	
Max. and min. transmission	1.000 and 0.567	
Refinement method	Full-matrix least-squares on F^2	
Data / restraints / parameters	5818 / 0 / 238	
Goodness-of-fit on F^2	1.049	
Final R indices [I>2sigma(I)]	R_1 = 0.0753, wR_2 = 0.1651	
R indices (all data)	R_1 = 0.1455, wR_2 = 0.2375	
Extinction coefficient	n/a	
Largest diff. peak and hole	0.429 and -0.578 e.Å^{-3}	

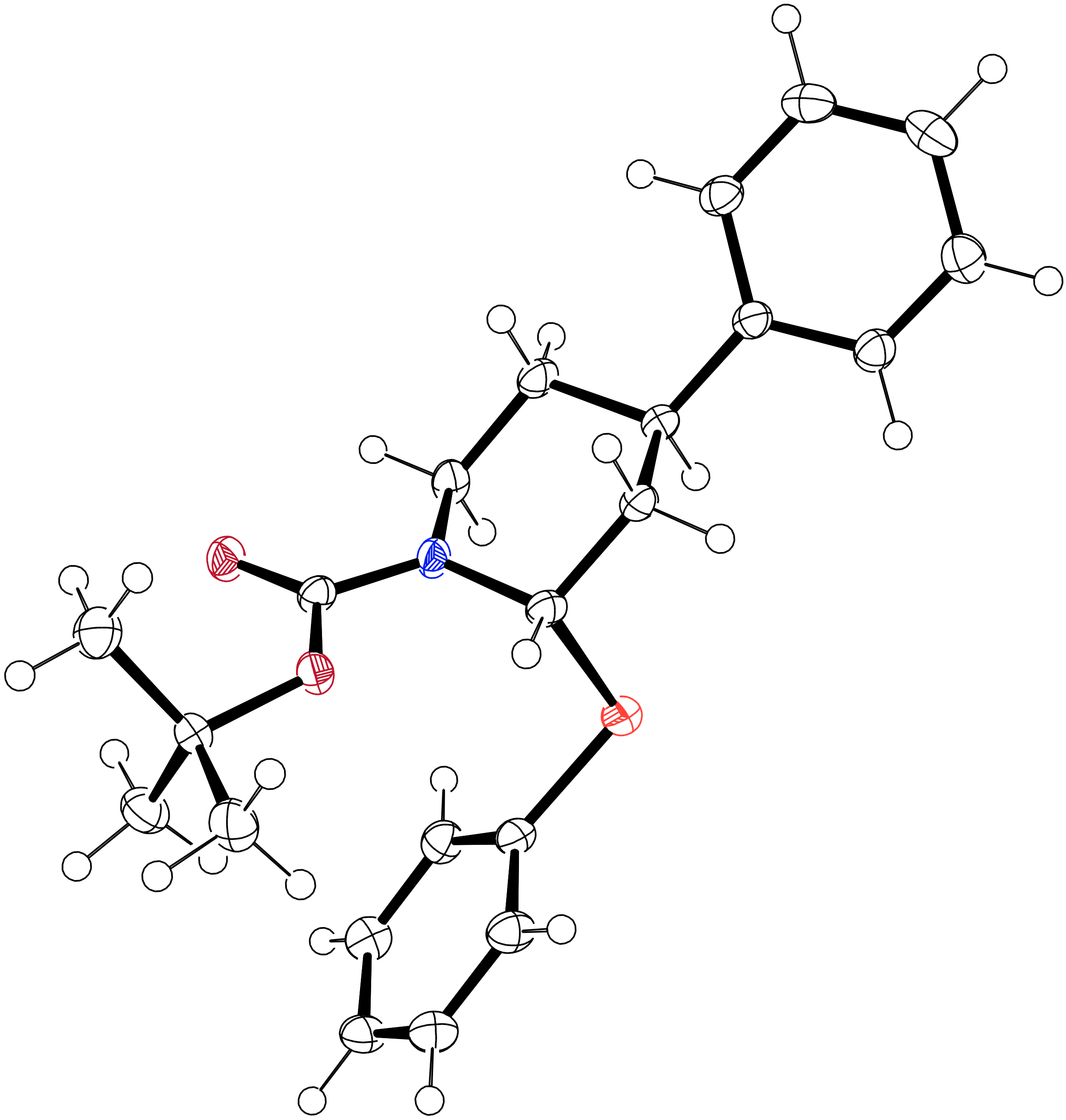

Figure S1. ORTEP drawing of **1a** with 50% thermal ellipsoids.

Table S2. Crystal data and structure refinement for **1b**

Empirical formula	$C_{17}H_{25}NO_2S$	
Formula weight	307.44	
Temperature	100(2) K	
Wavelength	0.71075 Å	
Crystal system	Orthorhombic	
Space group	Pbca	
Unit cell dimensions	*a* = 10.541(4) Å	α = 90°.
	b = 16.901(6) Å	β = 90°.
	c = 18.710(6) Å	γ = 90°.
Volume	3333(2) $Å^3$	
Z	8	
Density (calculated)	1.225 Mg/m^3	
Absorption coefficient	0.199 mm^{-1}	
F(000)	1328	
Crystal size	0.200 x 0.200 x 0.200 mm^3	
Theta range for data collection	2.177 to 27.495°.	
Index ranges	-13<=h<=11, -20<=k<=19, -24<=l<=13	
Reflections collected	12485	
Independent reflections	3720 [R(int) = 0.0451]	
Completeness to theta = 25.242°	98.3 %	
Refinement method	Full-matrix least-squares on F^2	
Data / restraints / parameters	3720 / 0 / 194	
Goodness-of-fit on F^2	1.161	
Final R indices [I>2sigma(I)]	R1 = 0.0564, wR2 = 0.1343	
R indices (all data)	R1 = 0.0686, wR2 = 0.1421	
Extinction coefficient	n/a	
Largest diff. peak and hole	0.685 and -0.444 e.$Å^{-3}$	

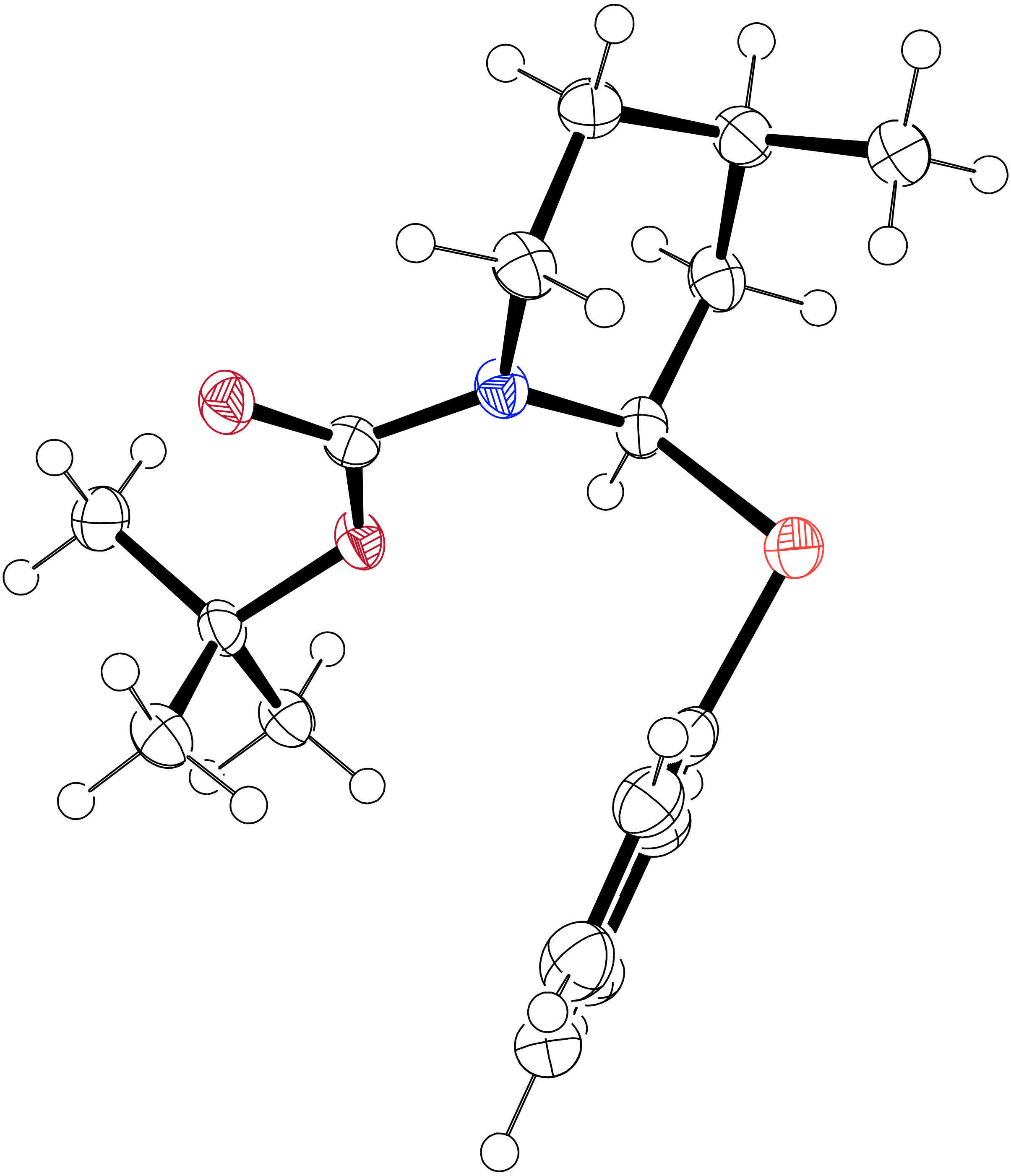

Figure S2. ORTEP drawing of **1b** with 50% thermal ellipsoids.

Table S3. Crystal data and structure refinement for **1d**

Empirical formula	$C_{17}H_{25}NO_2S$	
Formula weight	307.44	
Temperature	100(2) K	
Wavelength	0.71075 Å	
Crystal system	Trigonal	
Space group	$P3_1$	
Unit cell dimensions	*a* = 10.154(4) Å	α = 90°.
	b = 10.154(4) Å	β = 90°.
	c = 14.189(5) Å	γ = 120°.
Volume	1267.0(10) $Å^3$	
Z	3	
Density (calculated)	1.209 Mg/m^3	
Absorption coefficient	0.196 mm^{-1}	
F(000)	498	
Crystal size	0.580 x 0.320 x 0.200 mm^3	
Theta range for data collection	3.690 to 27.437°.	
Index ranges	-13<=h<=13, -13<=k<=13, -18<=l<=18	
Reflections collected	20112	
Independent reflections	3847 [R(int) = 0.0350]	
Completeness to theta = 25.242°	99.7 %	
Refinement method	Full-matrix least-squares on F^2	
Data / restraints / parameters	3847 / 1 / 194	
Goodness-of-fit on F^2	0.987	
Final R indices [I>2sigma(I)]	R1 = 0.0255, wR2 = 0.0653	
R indices (all data)	R1 = 0.0278, wR2 = 0.0662	
Absolute structure parameter	-0.020(19)	
Extinction coefficient	n/a	
Largest diff. peak and hole	0.160 and -0.137 e.$Å^{-3}$	
Largest diff. peak and hole	0.159 and -0.138 e.$Å^{-3}$	

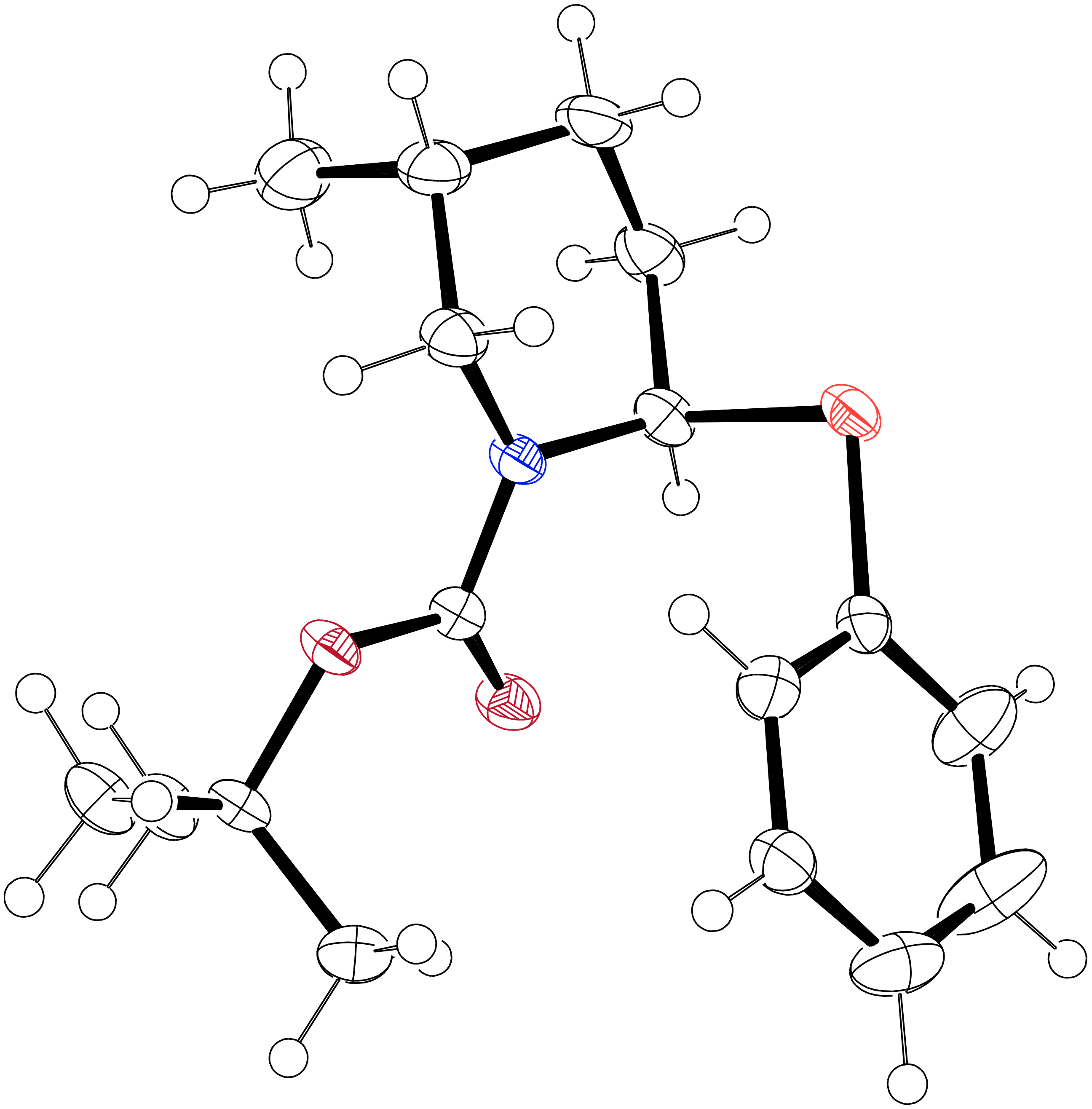

Figure S3. ORTEP drawing of **1d** with 50% thermal ellipsoids.

Table S4. Crystal data and structure refinement for **1e**

Empirical formula	$C_{22}H_{27}NO_2S$	
Formula weight	369.50	
Temperature	110(2) K	
Wavelength	0.71075 Å	
Crystal system	Orthorhombic	
Space group	Pbca	
Unit cell dimensions	*a* = 10.555(2) Å	α = 90°.
	b = 21.275(4) Å	β = 90°.
	c = 17.615(3) Å	γ = 90°.
Volume	3955.6(13) $Å^3$	
Z	8	
Density (calculated)	1.241 Mg/m^3	
Absorption coefficient	0.179 mm^{-1}	
F(000)	1584	
Crystal size	0.220 x 0.180 x 0.130 mm^3	
Theta range for data collection	1.914 to 27.500°.	
Index ranges	-13<=h<=13, -27<=k<=27, -22<=l<=22	
Reflections collected	60370	
Independent reflections	4540 [R(int) = 0.0541]	
Completeness to theta = 25.242°	99.9 %	
Refinement method	Full-matrix least-squares on F^2	
Data / restraints / parameters	4540 / 0 / 238	
Goodness-of-fit on F^2	1.300	
Final R indices [I>2sigma(I)]	R_1 = 0.0500, wR2 = 0.1426	
R indices (all data)	R_1 = 0.0581, wR2 = 0.1601	
Extinction coefficient	n/a	
Largest diff. peak and hole	0.427 and -0.545 e.$Å^{-3}$	

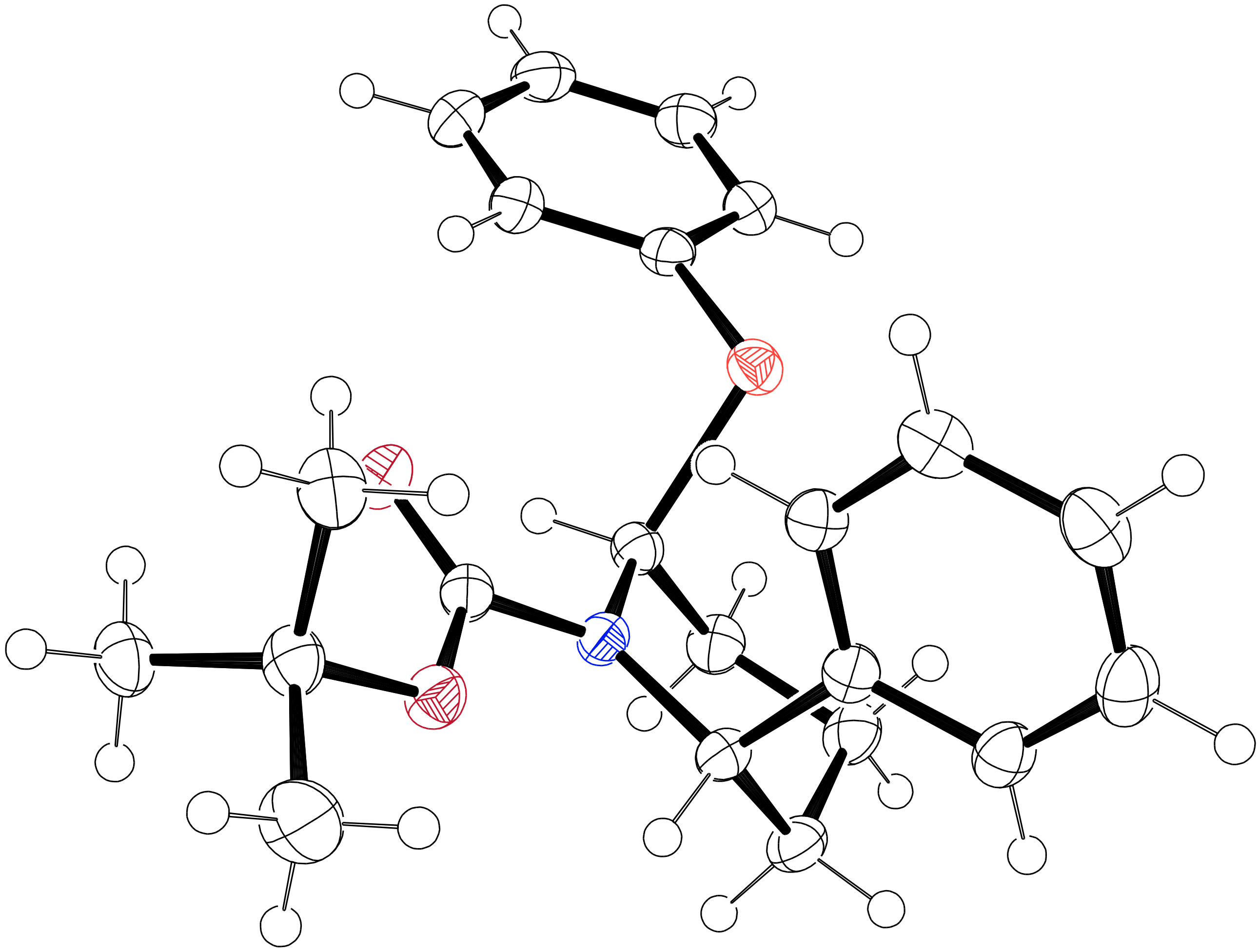

Figure S4. ORTEP drawing of **1e** with 50% thermal ellipsoids.

Table S5. Crystal data and structure refinement for **1f**

Empirical formula	$C_{17}H_{25}NO_2S$	
Formula weight	307.44	
Temperature	100(2) K	
Wavelength	0.71075 Å	
Crystal system	Orthorhombic	
Space group	$Pna2_1$	
Unit cell dimensions	a = 27.491(19) Å	α = 90°.
	b = 5.651(4) Å	β = 90°.
	c = 21.462(14) Å	γ = 90°.
Volume	3334(4) $Å^3$	
Z	8	
Density (calculated)	1.225 Mg/m^3	
Absorption coefficient	0.199 mm^{-1}	
F(000)	1328	
Crystal size	0.190 x 0.060 x 0.050 mm^3	
Theta range for data collection	1.481 to 27.498°.	
Index ranges	-32<=h<=35, -7<=k<=7, -27<=l<=19	
Reflections collected	21267	
Independent reflections	6444 [R(int) = 0.0864]	
Completeness to theta = 25.242°	99.8 %	
Refinement method	Full-matrix least-squares on F^2	
Data / restraints / parameters	6444 / 1 / 387	
Goodness-of-fit on F^2	1.158	
Final R indices [I>2sigma(I)]	R_1 = 0.0694, wR_2 = 0.1637	
R indices (all data)	R_1 = 0.0817, wR_2 = 0.1822	
Absolute structure parameter	0.31(10)	
Extinction coefficient	n/a	
Largest diff. peak and hole	0.338 and -0.423 e.$Å^{-3}$	

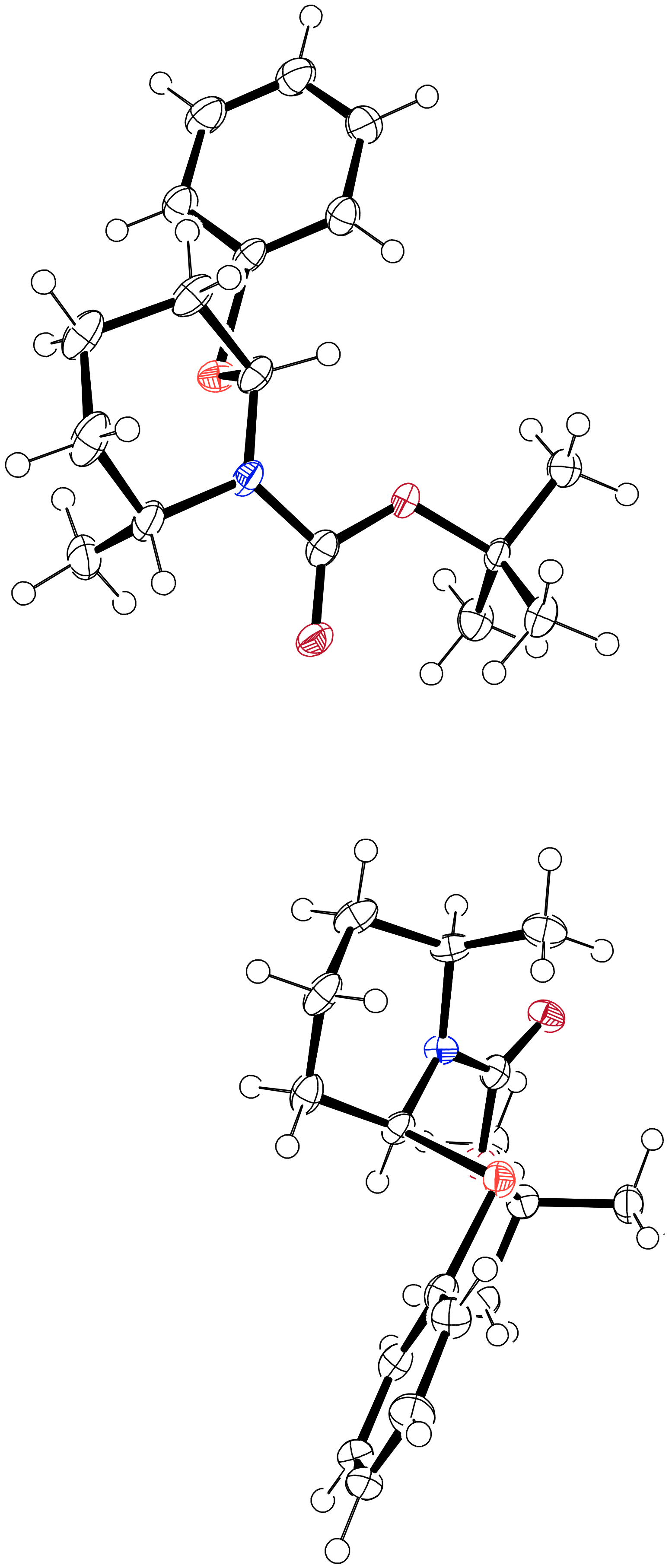

Figure S5. ORTEP drawing of **1f** with 50% thermal ellipsoids.

3. Theoretical calculations

Density functional theory (DFT) calculations were performed using the Gaussian 09 program.[11] Geometries were optimized at the B3LYP/6-31G(d) level of theory.[12] Thermochemical corrections were obtained from frequency calculations at the same level of theory. The data were summarized in Figure S6. Calculated structures are illustrated using ChemDraw and CYLView.[13]

Underneath the Cartesian coordinates for the optimized geometries are listed the following energies:
B3LYP/6-31G(d) electronic energy (E)
B3LYP/6-31G(d) enthalpy at 298.15 K (H)
B3LYP/6-31G(d) Gibbs free energy at 298.15 K (G),

Otherwise noted, all energies are given in Hartree.

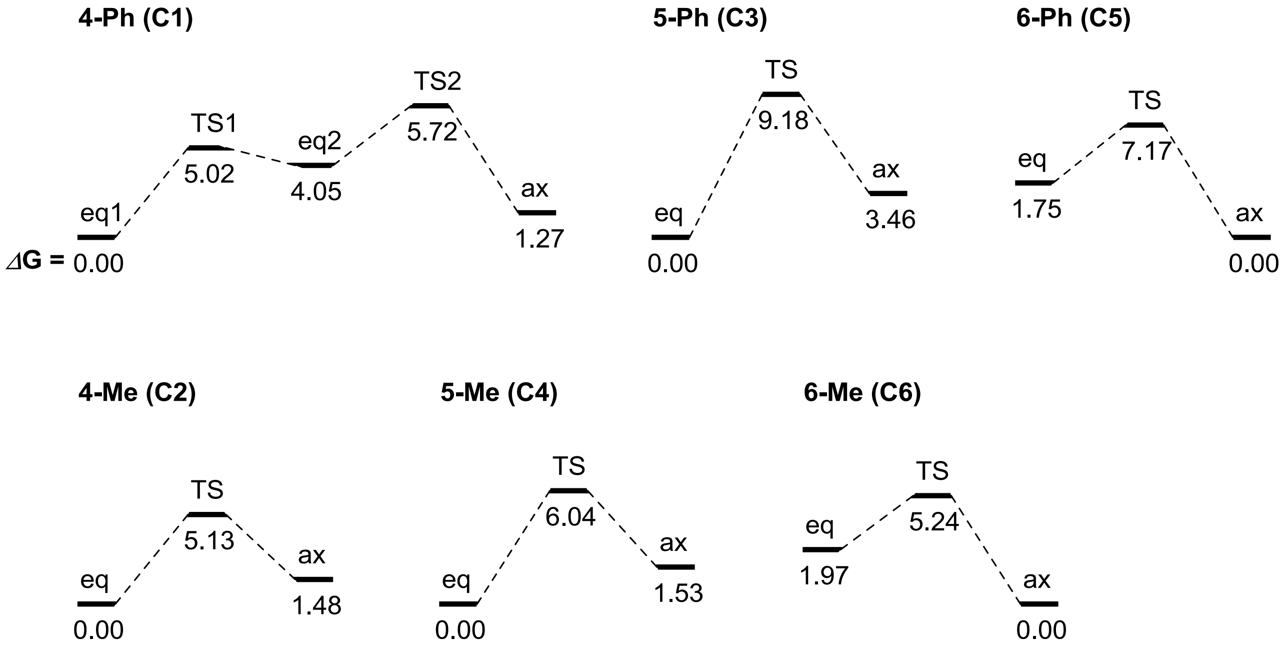

Figure S6. Energy diagram of flipping of *N*-acyliminium ions. The values of ⊿G are given in kcal/mol

Computed Geometries and Energies

C1 (equatorial 1)

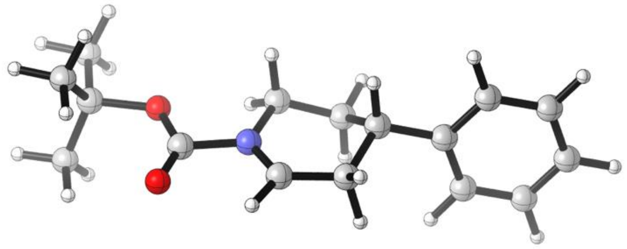

C	0.389375386580	-0.925826268684	0.266307212243
C	-1.089482583195	-1.092909892558	-0.075066307855
C	-1.932433912162	0.088991677650	0.441751597215
C	-1.382846334846	1.385140238545	-0.187974548971
C	0.091833287239	1.444555156692	-0.292946937300
N	0.882950843623	0.440793679462	-0.086430903721
H	-1.775624563540	0.161167234928	1.525924354019
H	-1.716304819198	2.284515613828	0.348760073010
H	-1.766724785615	1.530303611549	-1.212321016625
H	0.593973473332	2.374103686592	-0.559881721060
H	-1.208652719791	-1.196092204902	-1.160445113716
C	2.365341933239	0.713156196941	-0.199433090422
O	2.731690793504	1.812414296464	-0.514612586780
O	3.005018271918	-0.376870428768	0.097191621979
C	4.545801085063	-0.434055040051	0.089633144794
H	-1.435419038547	-2.030412659231	0.370570758003
H	1.016987324721	-1.632377839726	-0.276915852733
H	0.587170001095	-1.062159253609	1.334402605200
C	5.059557682653	0.557096941741	1.128062353759
H	6.146210649762	0.446705304285	1.203858338007
H	4.843939076428	1.590485878649	0.849146432396
H	4.633552864804	0.348481768859	2.114606543451
C	5.021368431176	-0.137230908466	-1.328279467006
H	6.106129862527	-0.280350893266	-1.366736453628
H	4.568136482602	-0.827239225426	-2.047128031163
H	4.805983501585	0.891143341435	-1.625669005695
C	4.798199134117	-1.880756646257	0.495450099415
H	5.877371754263	-2.057455210628	0.533837966244
H	4.383187218682	-2.092227188169	1.485402193279
H	4.365456647831	-2.577261267852	-0.229003931456
C	-3.423188251995	-0.078159259985	0.197329245241
C	-4.307887487369	-0.127572893449	1.280769269824

C	-3.938614144004	-0.192877343833	-1.101927086076
C	-5.678968320322	-0.288605937294	1.073924495441
H	-3.924529368562	-0.039917088299	2.295040486816
C	-5.307990135274	-0.353583330842	-1.310896215456
H	-3.275095583457	-0.163466749243	-1.964689429008
C	-6.181649154118	-0.401598371902	-0.222353202743
H	-6.351543681309	-0.324333669127	1.925822521427
H	-5.692114381929	-0.442412486499	-2.322871857946
H	-7.247972441514	-0.526071569553	-0.385389552404

0 imaginary frequencies

E = -827.951452

H = -827.575528

G = -827.642879

C1 (TS1)

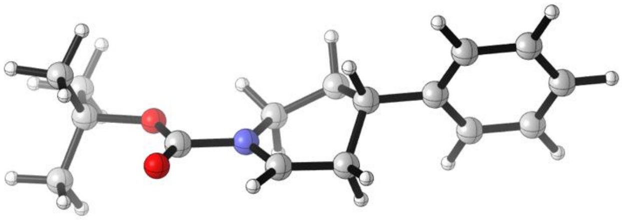

C	-1.087406044671	1.047326087886	0.579894247943
C	-2.506669226381	1.421324584947	0.072332352530
C	-3.382267108218	0.230438916485	-0.380661347300
C	-2.983133942468	-1.047565432760	0.423742553229
C	-1.544411028341	-1.308927547592	0.220034511502
N	-0.678946717090	-0.344037290359	0.240638736651
H	-3.149094889679	0.013038761833	-1.429300345688
H	-3.570886805625	-1.919897903392	0.129216580114
H	-3.170828971846	-0.879284564122	1.495684950617
H	-1.140832901043	-2.293423055969	-0.010910641127
H	-3.008281901468	1.956972013447	0.882638153693
C	0.761099111323	-0.711526739770	-0.020117385382
O	1.024020324318	-1.851107006153	-0.293280931878
O	1.495233453684	0.350594776455	0.117357532733
C	3.023397033734	0.303885931597	-0.078201508581
H	-2.417642426892	2.126883669421	-0.758161660566
H	-1.029636257296	1.113879998098	1.671413086482
H	-0.322443330873	1.705425633211	0.175580059555

C	3.301170138725	-0.103838566292	-1.520926349517
H	4.380052722919	-0.042499461986	-1.696745976058
H	2.984183797452	-1.128701363786	-1.724826356999
H	2.809445590378	0.576968407811	-2.223030900982
C	3.603703102360	-0.657934006975	0.952925761878
H	4.695958260347	-0.607252031252	0.896611374972
H	3.308694428193	-0.370787629994	1.967188930041
H	3.303248173115	-1.690568098939	0.764784742110
C	3.409399195849	1.751482271604	0.199705192631
H	4.493136740333	1.858374802036	0.093281698868
H	2.929549748050	2.432759652164	-0.509493337509
H	3.136232173302	2.045743430560	1.217454222074
C	-4.873839478528	0.503547487656	-0.288920982638
C	-5.661582423776	0.455300292553	-1.445064742616
C	-5.488173820484	0.796104316612	0.937348311812
C	-7.034246389686	0.701555136389	-1.381328035023
H	-5.200563429829	0.228378843995	-2.403771502766
C	-6.859224746451	1.039084042993	1.003531585889
H	-4.901049992777	0.839702621936	1.853131179489
C	-7.635593936283	0.992923346066	-0.157137678197
H	-7.630501463930	0.664618049534	-2.288191260682
H	-7.321224607199	1.264793582752	1.960104938192
H	-8.703465093251	1.182800961304	-0.105191309494

1 imaginary frequency

E = -827.944177

H = -827.568899

G = -827.634877

C1 (equatorial 2)

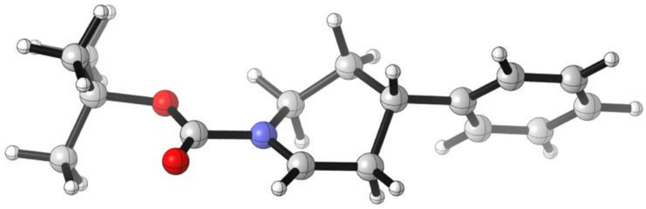

C	-0.248761581977	0.927106277350	-0.139707483561
C	0.988574579207	0.789476763755	0.768635488868
C	1.906006542683	-0.420372453360	0.420618806263
C	1.373466264268	-1.165101462064	-0.857800792991
C	-0.083318885928	-1.380312188062	-0.761933154943

N	-0.857860206811	-0.402151407639	-0.406239718538
H	1.828124489362	-1.145009663191	1.236996480317
H	1.895777331864	-2.110293299141	-1.016447404300
H	1.572206304520	-0.533703879887	-1.738174424733
H	-0.573387973718	-2.335450699185	-0.942265987546
H	1.544848597312	1.727503308713	0.692096883600
C	-2.332552579211	-0.680806384943	-0.300081675634
O	-2.724561705735	-1.792276534246	-0.531869894569
O	-2.942327114349	0.413252009222	0.043221232622
C	-4.471942735195	0.458158904211	0.225719571216
H	0.655074054666	0.706081037306	1.807318760078
H	0.019600231148	1.345730444464	-1.116044102894
H	-1.013221780262	1.560915326698	0.301776934410
C	-4.843235772065	-0.505764948918	1.347276154536
H	-5.912617532209	-0.398189018720	1.555979810470
H	-4.655566567484	-1.545615007369	1.072355091205
H	-4.298449428425	-0.267115646610	2.266265039471
C	-5.115780732719	0.117239970070	-1.113739384373
H	-6.198734438141	0.248874832527	-1.021391769019
H	-4.763433515800	0.791117677460	-1.901083116255
H	-4.925626987935	-0.916977289517	-1.408004936883
C	-4.688534758074	1.914425204128	0.618436472398
H	-5.757243495067	2.086031446460	0.778835173465
H	-4.162585497205	2.156826520673	1.546839460873
H	-4.349934148773	2.591834589530	-0.171206555223
C	3.376691746838	-0.076220969276	0.251694738651
C	4.339869752368	-0.745986838955	1.014688617820
C	3.797380757468	0.892183164396	-0.671391019183
C	5.696633170899	-0.451351613260	0.865219047378
H	4.029919146830	-1.499227813186	1.735591015251
C	5.151364993668	1.186732637880	-0.823431008147
H	3.069546724662	1.429663148702	-1.277346178416
C	6.104692833181	0.514483392815	-0.054114059263
H	6.431151558316	-0.976388038167	1.468607976555
H	5.462364730719	1.941085155987	-1.540171803723
H	7.159270627103	0.744815343350	-0.171895285250

0 imaginary frequencies

E = -827.944675

H = -827.568486

G = -827.636432

C1 (TS2)

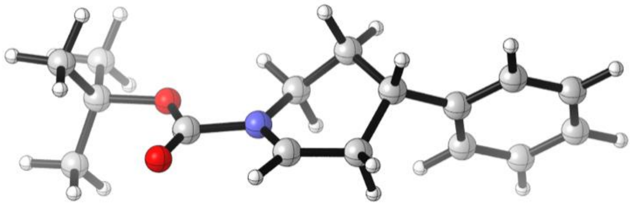

C	-0.109988722594	0.658891349062	0.268832577035
C	1.007554879021	0.358395095034	1.272089380025
C	2.018568487792	-0.719924479192	0.787103827628
C	1.371941727138	-1.669717258728	-0.278041744954
C	-0.104828836782	-1.678961376062	-0.314139305224
N	-0.801692701494	-0.609160230470	-0.091086029025
H	2.243970497018	-1.349827264205	1.652469008164
H	1.743169351189	-2.694362755278	-0.191535810363
H	1.677303571392	-1.337255255839	-1.286363041448
H	-0.679107867567	-2.568020581161	-0.570254322858
H	1.530112930163	1.293923102122	1.488644696398
C	-2.299039065798	-0.719743859192	-0.219445756312
O	-2.775107015346	-1.785010561681	-0.504346727794
O	-2.825001696960	0.443728915914	0.016306653174
C	-4.349980021454	0.663134253133	-0.036010910281
H	0.537283260055	0.030383747539	2.206211323324
H	0.273504841180	1.094313091219	-0.659738836982
H	-0.857885075260	1.333551360007	0.677999130253
C	-4.991184874811	-0.225100391834	1.024520687255
H	-6.060675849120	0.005077836279	1.068351279862
H	-4.883283710399	-1.285970603582	0.789825801992
H	-4.566169200596	-0.025007692626	2.013273318037
C	-4.820385370892	0.361315111910	-1.454481813692
H	-5.883251839559	0.613710506266	-1.528122998651
H	-4.280924128761	0.972121905209	-2.185299733890
H	-4.705704701533	-0.694817312440	-1.706513200881
C	-4.456367080864	2.143877761412	0.307036510325
H	-5.510629542056	2.436916223513	0.300251568717
H	-4.053126244855	2.350137686551	1.303164469355
H	-3.927357826228	2.759296261791	-0.426754449802
C	3.339836535651	-0.148889974747	0.289489436112
C	4.511877557025	-0.391114814552	1.015149706871

C	3.418044493689	0.638494804916	-0.869416396604
C	5.733894448717	0.139577976807	0.597007088516
H	4.472208131133	-1.000646163090	1.915087360693
C	4.636916222475	1.171260476784	-1.288604688441
H	2.527712400916	0.845616383664	-1.461051358630
C	5.799058752893	0.922499007878	-0.555191776333
H	6.632927175639	-0.062855055291	1.171553767874
H	4.679026882250	1.779111492338	-2.187705695639
H	6.748621227591	1.334708280622	-0.882912993806

1 imaginary frequency

E = -827.944054

H = -827.568990

G = -827.633759

C1 (axial)

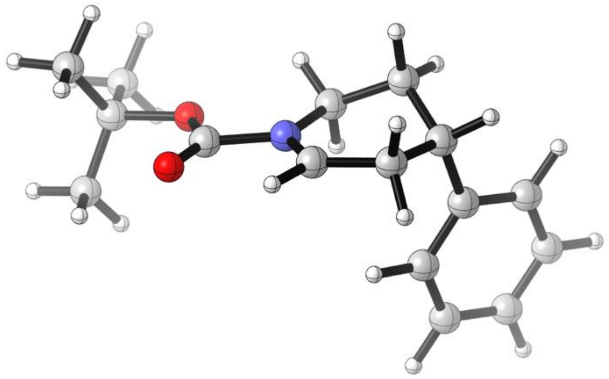

C	0.025754355738	-0.007572516180	1.219630979347
C	1.173008936232	-0.691199581459	1.957096552907
C	2.190007960186	-1.319970241068	0.976503921052
C	1.433571237555	-2.336959402641	0.103417460909
C	0.057719717379	-1.943959029269	-0.275887770462
N	-0.581493555078	-0.929713978072	0.214426267605
H	2.917672853897	-1.881225464963	1.571903499785
H	1.321573100201	-3.301233494226	0.628531892757
H	1.975691657096	-2.593662579044	-0.816854445606
H	-0.504135687595	-2.531439586633	-1.001254452547
H	1.661515770200	0.057677521443	2.587072759607
C	-1.973456956052	-0.687842900624	-0.316703446235
O	-2.433114561721	-1.459656431597	-1.114161695967
O	-2.439134727445	0.395188558386	0.227247802027

C	-3.844093689722	0.926461901366	-0.114553265391
H	0.770507719201	-1.463755558785	2.624982898604
H	0.364446069719	0.876293418328	0.669117134554
H	-0.774092495613	0.296317819847	1.893401728168
C	-4.862447183078	-0.127854956170	0.305710523918
H	-5.866865252425	0.284670681740	0.165219056583
H	-4.783405806771	-1.035986152832	-0.295364131157
H	-4.750756394545	-0.380256016810	1.364949382246
C	-3.876923887819	1.250994482236	-1.604162853769
H	-4.833552772616	1.731834073794	-1.832867532790
H	-3.079061408879	1.951616828237	-1.870227544182
H	-3.794252542030	0.353657019621	-2.220779290323
C	-3.915127041832	2.178029833910	0.751643316781
H	-4.882650922067	2.665025958124	0.596702986093
H	-3.825317336719	1.930491186619	1.813693143838
H	-3.128290252334	2.889425281183	0.483842695424
C	2.954361645709	-0.262949263192	0.186417786720
C	4.043851929076	0.370176168423	0.804902195718
C	2.605623586002	0.131347800231	-1.112286421248
C	4.757357405386	1.371113124679	0.149311058692
H	4.342508780601	0.068645455142	1.806814529016
C	3.319067446752	1.135939943939	-1.772637528489
H	1.785453265029	-0.349286542325	-1.642298494433
C	4.395220322633	1.758769570765	-1.143349967483
H	5.601868476875	1.842140347744	0.643593656907
H	3.036527179671	1.421059539356	-2.781848779817
H	4.954950059203	2.533669180777	-1.658262609362

0 imaginary frequencies

E = -827.950390

H = -827.574353

G = -827.640861

C2 (equatorial)

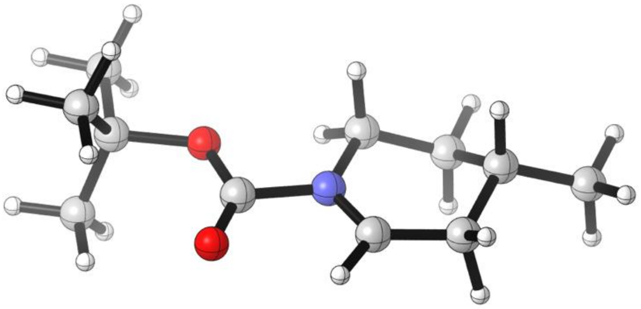

C	-1.066825608949	1.120341641434	0.004046430355
C	-2.527167677793	1.295312991725	0.414270145768
C	-3.459320468031	0.359132088304	-0.368727763692
C	-3.009039492695	-1.090836482186	-0.121146614163
C	-1.542156397868	-1.284738359994	-0.063641483415
N	-0.677553672871	-0.323045795548	-0.017654052082
H	-3.333037412161	0.568507750511	-1.440242140160
H	-3.405646746491	-1.791825525981	-0.869486029099
H	-3.398226452820	-1.475144143543	0.839091332117
H	-1.112876193696	-2.286181053429	-0.052697956567
H	-2.632309006185	1.111916473607	1.491919815732
C	0.781324439329	-0.724555204486	-0.001409374094
O	1.060432486618	-1.892184284266	0.012823867273
O	1.501842420048	0.354554281771	-0.014729552466
C	3.043713035064	0.294604542832	-0.011019051498
H	-2.798482114749	2.342441634181	0.244599990576
H	-0.383772308156	1.616003586066	0.693619177303
H	-0.867485829088	1.512133137080	-0.998518127072
C	-4.934252746853	0.555022354310	-0.005407360854
H	-5.258765612958	1.572988164815	-0.242302360334
H	-5.574836283260	-0.136616232678	-0.562663108546
H	-5.104455853241	0.387123811835	1.064865976701
C	3.489342765982	-0.425789352804	-1.278550809250
H	4.581636428442	-0.379498771543	-1.336950827712
H	3.196408097235	-1.477745251070	-1.276410228224
H	3.086433028657	0.063722949800	-2.170878067093
C	3.484577709099	-0.396557249038	1.274417527438
H	4.576778715819	-0.349665511036	1.335094688307
H	3.079659665166	0.114248686405	2.153867562228
H	3.190323766000	-1.447831148759	1.295923847366
C	3.404876404226	1.774646119986	-0.027000234940
H	4.494354697110	1.875896979170	-0.026427615428
H	3.017792908658	2.267376209232	-0.923846480661

H	3.015411310413	2.286963963293	0.857895876185

0 imaginary frequencies

E = -636.216332

H = -635.896457

G = -635.954971

C2 (TS)

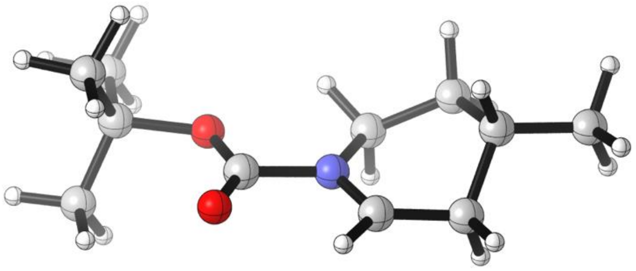

C	-1.079112146074	1.081009599490	0.500002880323
C	-2.491459937009	1.390085329793	-0.066249297208
C	-3.369943948278	0.156022029575	-0.364076370004
C	-2.956109998081	-1.024757451607	0.558017018652
C	-1.514933227556	-1.299596452675	0.375336545125
N	-0.657237441940	-0.332362356622	0.298957343014
H	-3.172897597465	-0.170423457009	-1.392923050847
H	-3.535496600092	-1.930500344758	0.362419357092
H	-3.134391240649	-0.749138299028	1.610811558726
H	-1.104989491882	-2.300348106081	0.249134469974
H	-2.994593352444	2.027571729679	0.668379269120
C	0.789012631466	-0.714325284218	0.079666026628
O	1.058654242596	-1.874869336184	-0.068878635676
O	1.512894375768	0.362215846848	0.102782018322
C	3.044219569301	0.307479487523	-0.085450196314
H	-2.389596134188	1.984044209580	-0.978773132916
H	-1.044835548352	1.248215185513	1.581618738851
H	-0.311424697005	1.705957577333	0.051016340855
C	-4.865009328038	0.464060109040	-0.235623666644
H	-5.142898448186	1.289659538105	-0.898747278444
H	-5.473511717184	-0.403086111073	-0.511533648114
H	-5.123152939211	0.753064854073	0.789838553591
C	3.327255899361	-0.261786557913	-1.470953724169
H	4.405195054069	-0.205137166565	-1.654270894989
H	3.025331034625	-1.307913163557	-1.552849908343

H	2.825490228152	0.325099010108	-2.246793036779
C	3.628388240557	-0.524730635800	1.050508598426
H	4.720400368171	-0.479362817213	0.985914984704
H	3.334823413128	-0.121066480507	2.024680198100
H	3.330024193211	-1.573050576533	0.985461858955
C	3.415703936642	1.780966121091	0.024612433429
H	4.499202206820	1.885029198214	-0.087157541604
H	2.935002316091	2.371034006201	-0.761544895275
H	3.133837083676	2.189004765177	0.999888083441

1 imaginary frequency

E = -636.208696

H = -635.889371

G = -635.946794

C2 (axial)

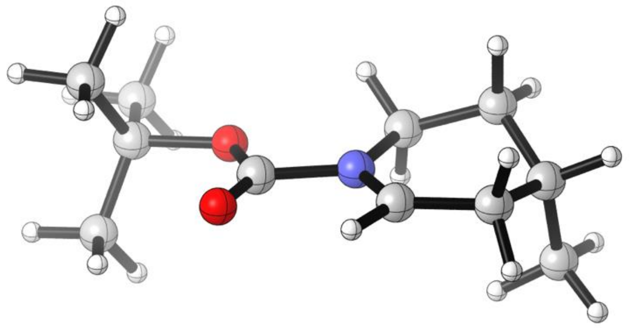

C	-1.219836722836	1.078650825075	-0.374570483905
C	-2.627694835133	1.214725952961	-0.953304374243
C	-3.623917466286	0.234413176115	-0.308172665227
C	-3.094881991586	-1.195481932603	-0.528038334063
C	-1.627539984119	-1.339479351989	-0.385663938049
N	-0.793787647025	-0.351748617182	-0.316140619280
H	-4.577246466362	0.320584888612	-0.839804070008
H	-3.327192617228	-1.570111845252	-1.540431310520
H	-3.563907043873	-1.928817209317	0.143120986664
H	-1.168469208155	-2.326837123960	-0.345316145033
H	-2.950872970735	2.249998526732	-0.804403914843
C	0.666199444464	-0.710313183679	-0.146416405008
O	0.980664835766	-1.868988992426	-0.134903784438
O	1.345746812491	0.388972252248	-0.029238839440
C	2.875895380460	0.371958873470	0.168493359769

H	-2.589157819941	1.048278289618	-2.037119662121
H	-1.143471167017	1.469468241188	0.644401291741
H	-0.479585822423	1.600286942273	-0.980973677956
C	-3.877720578673	0.523507197599	1.179708284353
H	-4.236292039346	1.548524579449	1.318227455773
H	-4.639887553369	-0.151254679192	1.582875023085
H	-2.975349217222	0.398616141393	1.791453329744
C	3.501217041528	-0.287862681364	-1.055461545494
H	4.590047316863	-0.207214277986	-0.973700758071
H	3.245843007286	-1.347207593365	-1.125483703880
H	3.197399033924	0.222519196058	-1.974949788337
C	3.173335761574	-0.353697797984	1.476010117938
H	4.246961846413	-0.278319826546	1.676874655188
H	2.644179055176	0.112651422968	2.313128973047
H	2.913571862368	-1.413099993397	1.425543920677
C	3.188330601576	1.861029722155	0.248087637711
H	4.264896831257	1.992084103098	0.393158985221
H	2.904710009883	2.375252464192	-0.675110384481
H	2.671510310300	2.329635311037	1.090847383486

0 imaginary frequencies

E = -636.214309

H = -635.894322

G = -635.952616

C3 (equatorial)

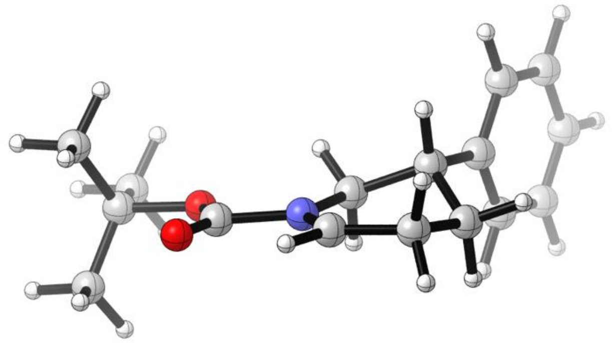

C	0.312549910145	0.268867295578	-0.073157143066
C	1.644586665402	0.816552365792	-0.610681844140
C	1.944782915524	2.186015561616	0.025052433894
C	0.804267930352	3.168742886862	-0.267180996245
C	-0.546400248632	2.565506058711	-0.211311395821
N	-0.776045859422	1.296039528688	-0.113353376289

H	2.885905375088	2.583696870490	-0.363211731780
H	0.803691681215	4.036100150252	0.408327841140
H	-1.437249424084	3.191069013203	-0.255494455940
C	-2.227878884170	0.878104504552	-0.030532350400
O	-3.080061581588	1.720698468471	-0.107157865108
O	-2.273960178950	-0.408927493764	0.129248518165
C	-3.615344946479	-1.156627728980	0.262757232841
H	1.521719854617	0.967487028302	-1.691906406748
C	-4.402781066980	-0.946635982150	-1.025820149810
H	-5.307620631875	-1.561592452622	-0.982572600424
H	-4.707712804258	0.093947941995	-1.154002497414
H	-3.822852529841	-1.267152017361	-1.897112380552
C	-4.325481643650	-0.633197264422	1.506378169445
H	-5.225004452308	-1.235462146500	1.670135124052
H	-3.690644662064	-0.733902269871	2.392417841459
H	-4.632678820670	0.408672418959	1.394861804142
C	-3.144476291261	-2.596370137395	0.426920068165
H	-4.017187508257	-3.247965495483	0.532001520365
H	-2.574793140555	-2.928386819761	-0.446222562242
H	-2.524114112244	-2.710502332180	1.320807162006
H	2.069036638558	2.079876190717	1.108608742264
H	-0.035200864161	-0.579148667077	-0.662494848949
H	0.889590712767	3.613948995368	-1.273611969377
H	0.395544957461	-0.062984241962	0.965980530002
C	2.745596456505	-0.213161632631	-0.406739259660
C	3.343521762005	-0.824143225971	-1.515771508831
C	3.177919685624	-0.572078632100	0.878429510696
C	4.355274041741	-1.770475662581	-1.347537370107
H	3.020907403380	-0.555321638789	-2.519247980807
C	4.188407617680	-1.518358090270	1.048139484537
H	2.734894960987	-0.111166018871	1.759089109406
C	4.779460860987	-2.119533750712	-0.065003773233
H	4.811448393843	-2.231930058250	-2.218361077600
H	4.515417439020	-1.783655795516	2.049229919209
H	5.567505388548	-2.854760724339	0.067677532757

0 imaginary frequencies

E = -827.951768

H = -827.575720

G = -827.642924

C3 (TS)

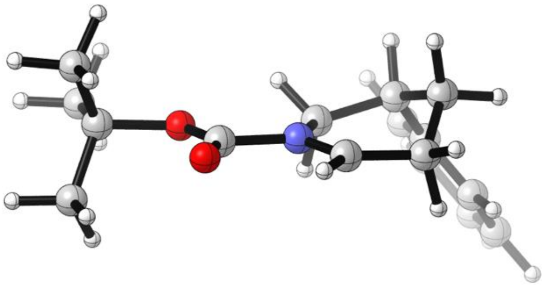

C	1.344400138405	0.817488479400	0.024918180380
C	2.841235681933	0.956055440284	0.476687377154
C	3.502287595344	-0.401256689791	0.795296793059
C	3.026990968402	-1.545124120319	-0.118496390390
C	1.551363793023	-1.622902700887	-0.133571622062
N	0.804534956467	-0.572840458226	-0.025351519031
H	3.271347369473	-0.684709408041	1.827819484385
H	3.351287035505	-1.387183930536	-1.160041424827
H	1.013987160596	-2.566074795228	-0.220076636633
C	-0.692303169028	-0.808489903679	-0.051285230964
O	-1.097729789434	-1.936280459512	-0.122927816966
O	-1.293273800337	0.339735615807	0.008991848563
C	-2.831624074407	0.445008247242	0.000744512952
H	2.808988895932	1.508801412266	1.421220729858
C	-3.361697190399	-0.286213430348	1.229108848008
H	-4.442822875699	-0.123777823877	1.288569143383
H	-3.184585392385	-1.362259677832	1.174014860859
H	-2.914044171473	0.110661170088	2.145851942114
C	-3.335773301033	-0.127443187841	-1.319594586701
H	-4.415997351730	0.040453597103	-1.379438720578
H	-2.871407280262	0.381249959770	-2.170393271553
H	-3.157132942508	-1.201990215271	-1.396058679464
C	-3.032954986213	1.952539687552	0.091797747966
H	-4.105358249224	2.169704292532	0.087755128843
H	-2.608195605264	2.353869036489	1.016862572638
H	-2.578391886966	2.464606203467	-0.761480151232
H	4.589034462607	-0.309192882180	0.731845949454
H	0.675454724256	1.373703836774	0.678925471357
H	3.433632869943	-2.516061833868	0.180859164596
H	1.214547944867	1.216904725641	-0.984886142221
C	3.595845860715	1.840523673555	-0.516310093475

C	3.381742438666	3.226305184205	-0.465094042190
C	4.469155948547	1.336997873008	-1.488527689650
C	4.013750175455	4.083992317332	-1.364593383998
H	2.726823480478	3.642703570571	0.298059551152
C	5.106889097061	2.194145202308	-2.388370540631
H	4.685158055095	0.273819597097	-1.548077700082
C	4.878142095708	3.568629147509	-2.332143140025
H	3.840818306895	5.154307078398	-1.300942798369
H	5.786734205923	1.784082216953	-3.129501305047
H	5.377304315065	4.234299822086	-3.029765330630

1 imaginary frequency

E = -827.939885

H = -827.939885

G = -827.628297

C3 (axial)

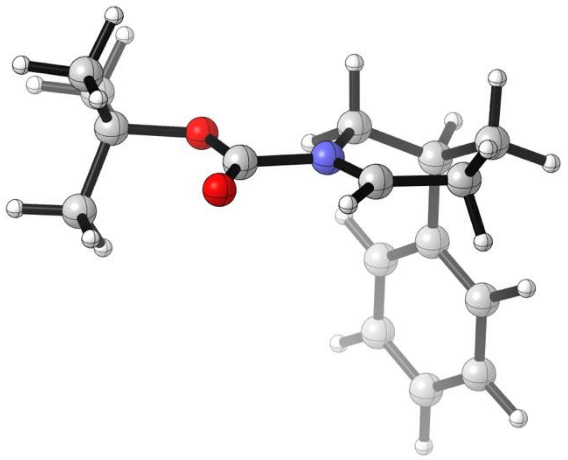

C	0.191844944326	0.352873448071	-1.362480743163
C	1.704981857313	0.635200639541	-1.427029998175
C	1.906142154628	2.162452385368	-1.449942478377
C	1.256114405291	2.842576282727	-0.234932820525
C	0.021081352812	2.189311426101	0.246965555738
N	-0.464538085442	1.096229049604	-0.244564936963
H	1.452271217396	2.558249141052	-2.365160339257
H	1.922217285134	2.885622845395	0.640529843003
H	-0.549992906301	2.619098052859	1.068925742480
C	-1.760319358778	0.605090930499	0.357453348315
O	-2.242418265053	1.215763773906	1.272195281399
O	-2.139417357554	-0.456266906684	-0.287174260906

C	-3.444114099947	-1.192442547379	0.072573012228
H	2.019225003940	0.249433032224	-2.405896954208
C	-4.605238244175	-0.229931359320	-0.151488229969
H	-5.541991436973	-0.775436053300	0.002145629208
H	-4.583513109311	0.606811513814	0.549661147542
H	-4.606086997956	0.152800914598	-1.177059198647
C	-3.327704477867	-1.687422817184	1.509979856260
H	-4.201758954783	-2.307502033252	1.734327204237
H	-2.435371340428	-2.307887569406	1.640453666531
H	-3.305257035328	-0.863457895737	2.225997715739
C	-3.436344588439	-2.334683270319	-0.935995626614
H	-4.333590793091	-2.944313467233	-0.792519671372
H	-3.442314922174	-1.956364356078	-1.962568205956
H	-2.562619948429	-2.978841635477	-0.798158680405
H	2.966538185297	2.420729782146	-1.496907977957
H	-0.313442998562	0.661986378119	-2.283391846867
H	1.006451192721	3.896298136906	-0.431002621965
H	-0.028548080619	-0.698600495979	-1.199536896873
C	2.490309697784	-0.128752951853	-0.352304314073
C	2.314448797725	-1.517825734802	-0.235094138695
C	3.434658125332	0.484219309330	0.480856364096
C	3.033981259201	-2.261013709080	0.698219744215
H	1.628313605344	-2.040167238787	-0.898543907734
C	4.157907319307	-0.257360162654	1.419339666765
H	3.645606057631	1.545689312278	0.396586021543
C	3.955241659056	-1.630437615504	1.537212353379
H	2.883410011534	-3.334776115538	0.761906733154
H	4.885218142580	0.243954957002	2.051176510434
H	4.518361726858	-2.207422375975	2.264425452431

0 imaginary frequencies

E = -827.946431

H = -827.946431

G = -827.946431

C4 (equatorial)

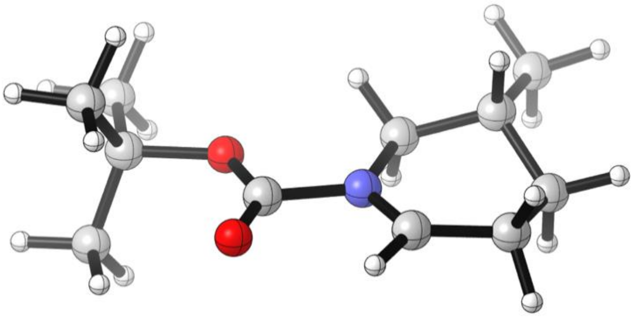

C	1.338442716751	0.764192643883	-0.009664186169
C	2.805211940740	0.793648101755	0.433098474890
C	3.594119485897	-0.301394277769	-0.298580538603
C	2.982069304184	-1.679991480708	-0.023106901884
C	1.502397787170	-1.684458424184	0.020562005259
N	0.768833461857	-0.618738796293	0.016472932956
H	4.641297005475	-0.296157702273	0.016968229200
H	3.291670304224	-2.440906814281	-0.753426806284
H	0.948890892819	-2.621885829755	0.063336371828
C	-0.730032289096	-0.832257513084	0.026236102501
O	-1.154579697720	-1.954100738267	0.078487904006
O	-1.308858527065	0.327217607159	-0.036689478078
C	-2.846333339354	0.459396677342	-0.060469742185
H	2.831996676486	0.578224949094	1.511265927461
C	-3.389313666158	-0.121078366041	1.240517435354
H	-4.467520961863	0.065486860816	1.278890806600
H	-3.230983534947	-1.199475919958	1.305081977315
H	-2.935446930848	0.367434701251	2.108622699262
C	-3.359744321755	-0.249121155841	-1.309003607895
H	-4.436902442538	-0.070622509597	-1.389775784391
H	-2.886586922301	0.151904765414	-2.210958364330
H	-3.199506539335	-1.328282060254	-1.262805969838
C	-3.019806428883	1.970880366655	-0.138348659235
H	-4.088143935380	2.206271244942	-0.163193429069
H	-2.583547412247	2.465347854994	0.734803456560
H	-2.560764288671	2.376448506965	-1.044854338592
H	3.587942736386	-0.103430713325	-1.377961916486
H	0.707721502200	1.370974199000	0.640075790500
H	3.309170140786	-2.091303473931	0.947539337493
C	3.387680717934	2.193303086202	0.201944956736
H	2.808073679362	2.960793915275	0.725794323545
H	4.415505811964	2.242402894847	0.573926296387
H	3.403578561665	2.446299663434	-0.864587464693

H	1.214449512260	1.135911736531	-1.032819840119

0 imaginary frequencies

E = -636.216270

H = -635.896331

G = -635.954871

C4 (TS)

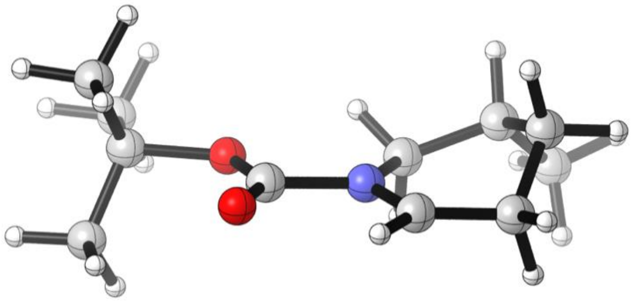

C	-1.091441727158	1.058035885696	0.449278266626
C	-2.545545195649	1.427127264510	0.016010874861
C	-3.360460399016	0.209729960409	-0.465420626696
C	-2.989981688437	-1.074709854107	0.306471512109
C	-1.538938623402	-1.322626640143	0.161561786873
N	-0.685789568011	-0.349481327254	0.172004128700
H	-3.188561043607	0.028564961753	-1.531918032812
H	-3.214001258833	-0.956842991750	1.379366183510
H	-1.115255294474	-2.312872059006	0.002026861231
C	0.770281982552	-0.722483904981	-0.016369459083
O	1.046675749350	-1.873863809257	-0.214332568644
O	1.490959654394	0.351230278675	0.085610237162
C	3.028060255425	0.306694906193	-0.055481559421
H	-2.455180760646	2.113823877668	-0.832126452265
C	3.357125122826	-0.194278275325	-1.457164975467
H	4.440616689017	-0.131088785830	-1.601504135655
H	3.058009516955	-1.234576312589	-1.600921591539
H	2.882045606117	0.430971482650	-2.219760636650
C	3.576214142827	-0.579648869622	1.057181057004
H	4.669706770286	-0.531114446569	1.030436542705
H	3.250640478187	-0.223433216050	2.039686868653
H	3.281518877698	-1.623445263560	0.931580768839
C	3.392608418688	1.773059173544	0.138313095611
H	4.478631139329	1.883380658347	0.063102489768
H	2.933759362282	2.401068097237	-0.631347027037

H	3.081810076540	2.131688074552	1.124090867230
H	-4.429849806971	0.404350816459	-0.345675293920
H	-0.351774855468	1.692211148285	-0.033988335422
H	-3.548259325059	-1.950690543265	-0.033805456200
C	-3.244860640126	2.177963870200	1.160334814059
H	-2.646925865019	3.029320316549	1.503516304246
H	-4.211495857522	2.566766339679	0.826332002234
H	-3.422975948954	1.525714475793	2.024780502674
H	-0.969243804116	1.186126241109	1.530555126716

1 imaginary frequency

E = -636.207129

H = -635.887930

G = -635.945242

C4 (axial)

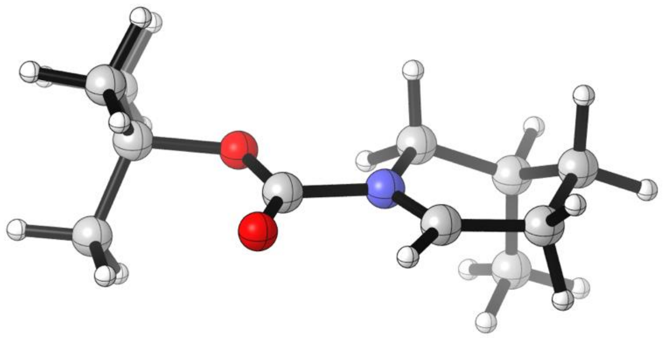

C	1.296603570671	0.925077515673	0.558927545792
C	2.795072421524	1.138618825882	0.301032201717
C	3.584015455206	-0.057964903871	0.863082694352
C	3.111493760825	-1.371912381039	0.226535638914
C	1.651870855392	-1.437933999912	-0.008075017740
N	0.843814944311	-0.437691415591	0.136100988793
H	3.446794733953	-0.114138018752	1.949297827313
H	3.579290959266	-1.557726660269	-0.754984441708
H	1.180912616688	-2.365206533533	-0.332843318314
C	-0.622482211633	-0.714171527488	-0.120926797840
O	-0.949381080052	-1.810120493448	-0.486345569775
O	-1.293579701785	0.367055286974	0.131836339519
C	-2.828887141367	0.416849197149	-0.016240392125
H	3.068418262376	2.029419047377	0.878643049759
C	-3.427455504025	-0.589124816794	0.960438414971
H	-4.517562412223	-0.490346334608	0.934329802345

H	-3.175857908619	-1.617800626002	0.694387548241
H	-3.098400907682	-0.385592337928	1.984394141631
C	-3.172019901737	0.135030324569	-1.474808711575
H	-4.249507574128	0.275056894021	-1.609513828002
H	-2.659087050296	0.835402572492	-2.141600160478
H	-2.926735165062	-0.888915658564	-1.764226524676
C	-3.124798267177	1.857382273381	0.381336992290
H	-4.203888755551	2.028860727291	0.322799939603
H	-2.803124014430	2.058908486273	1.407405599242
H	-2.631304388766	2.564069206381	-0.292664985085
H	4.655450261328	0.070055230548	0.686195721311
H	1.042881011626	1.019020138849	1.619674957612
H	3.388110072414	-2.253698799086	0.821860932389
C	3.096434336247	1.420966871241	-1.180242255368
H	2.574175175259	2.321265056019	-1.519902529059
H	4.168559613258	1.586081752097	-1.326029222710
H	2.796208472131	0.598584698693	-1.840689930636
H	0.683446462058	1.637374401978	0.007391349297

0 imaginary frequencies

E = -636.213751

H = -635.893765

G = -635.952439

C5 (equatorial)

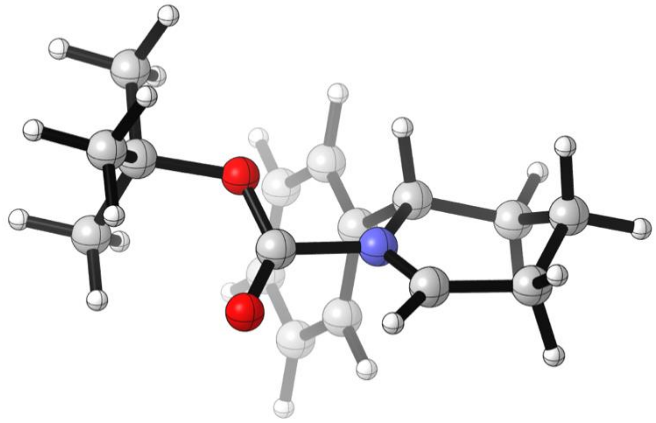

C	1.123980556411	-0.812675419547	0.762865818159
C	2.432420645405	-1.617033218956	0.863051316023
C	2.196204674064	-3.124184561842	0.971915821858
C	1.443477071985	-3.604030623628	-0.273175441846
C	0.336538858248	-2.695434011824	-0.659215485617
N	0.159466405615	-1.507966334925	-0.178510278598
H	1.611901984342	-3.360053709450	1.869584860310
H	1.016158040081	-4.609334614813	-0.159087377135
H	-0.412424612366	-3.017104079612	-1.381973298499
H	3.052103213867	-1.398321910988	-0.014835319309
C	-1.112041369751	-0.806479878231	-0.611928061832
O	-1.588356662824	-1.082851156752	-1.678557222348
O	-1.491567848827	-0.024268265198	0.350521712442
C	-2.734374248703	0.870854326298	0.211393005365
H	2.978099602124	-1.234839141641	1.730879646050
H	0.609273651608	-0.800557353494	1.727801327632
C	-3.953974311333	-0.029738135375	0.045861755210
H	-4.851897176179	0.596658047379	0.057835994334
H	-3.934665458075	-0.574686351327	-0.900079783638
H	-4.031835692808	-0.740467989431	0.874840723814
C	-2.505126212257	1.814709078547	-0.964464341293
H	-3.328832128842	2.535425520647	-0.996328276758
H	-1.571545528747	2.372235238375	-0.841044714899
H	-2.486532703009	1.282084464784	-1.917787172273
C	-2.735691694342	1.602334068572	1.548360570607
H	-3.592519487120	2.282058873780	1.583324628566
H	-2.824378732992	0.899474089941	2.382339626337
H	-1.823585736647	2.192535773091	1.674187246696
H	3.146259353777	-3.657692055790	1.060973949589

H	2.108542678671	-3.682729878201	-1.149696695614
C	1.367673341635	0.613949630593	0.301900544873
C	1.290364588417	1.657346067965	1.231489074201
C	1.729896028929	0.896621108548	-1.021933519998
C	1.573659148519	2.967402335403	0.843826765265
H	1.012309699579	1.446268414223	2.261336925020
C	1.996659215851	2.209535146181	-1.412354364971
H	1.804761398728	0.098548831168	-1.757538916872
C	1.921149734967	3.246227027226	-0.479950142749
H	1.520601364184	3.768883389216	1.574619917004
H	2.270228822076	2.419876546716	-2.441880077237
H	2.136845525739	4.266407712374	-0.782717737870

0 imaginary frequencies

E = -827.950776

H = -827.574869

G = -827.641063

C5 (TS)

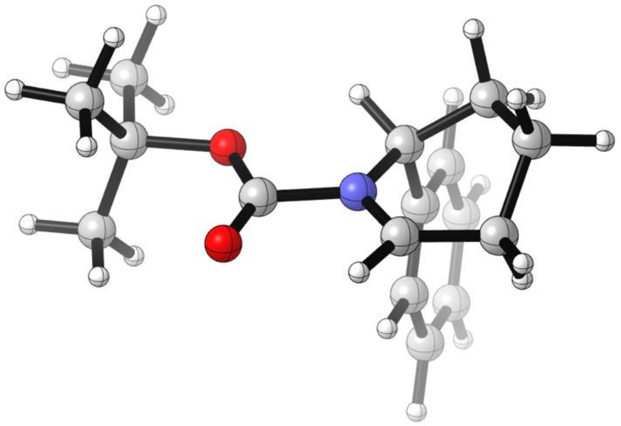

C	-1.396528137570	1.056192724126	-0.007839380656
C	-2.690732924757	1.249808830912	-0.866117745247
C	-3.630280086906	0.027371166213	-0.944621959791
C	-3.342334586056	-1.005925564004	0.169694974443
C	-1.895639669931	-1.316381815303	0.180922568335
N	-1.007605542322	-0.382728711804	0.051374650285
H	-3.516934522493	-0.475652732185	-1.909984967720
H	-3.919584466047	-1.925273867623	0.046921074990
H	-1.501156972625	-2.327728526444	0.265989531151
H	-3.218788332051	2.105548547675	-0.440202980231
C	0.440527307215	-0.814712617734	0.073254282010

O	0.689606465487	-1.977067910726	0.246853541285
O	1.192551702358	0.224928331326	-0.116353777793
C	2.729121989988	0.118784687519	-0.117776319806
H	-2.390874872884	1.534184784654	-1.878723890449
H	-0.568918830905	1.544589576951	-0.519848204103
C	3.139439565816	-0.797875642929	-1.264842171762
H	4.231831049556	-0.792774067733	-1.338414789494
H	2.816125952377	-1.827723686255	-1.100665119937
H	2.738988719613	-0.437291390349	-2.217687701263
C	3.166128345430	-0.382065898099	1.254544805952
H	4.259551916097	-0.352650859743	1.302261643277
H	2.778326909978	0.263997166671	2.048449509380
H	2.848023187079	-1.411055339333	1.434243549953
C	3.133212628082	1.567896606318	-0.360081425164
H	4.225193796678	1.636364290460	-0.376703513087
H	2.755435167207	1.928402920308	-1.321702572946
H	2.762230876130	2.220011155852	0.436228973471
H	-4.675404797822	0.338027071020	-0.873060672067
H	-3.605875364244	-0.582973924852	1.151057517147
C	-1.481844390344	1.649369213659	1.404934665768
C	-1.563886521235	3.047106648798	1.498870266813
C	-1.469191526134	0.893635079993	2.581152771178
C	-1.655099465867	3.670789387459	2.740907185239
H	-1.553966415347	3.654044674511	0.596303641662
C	-1.559272695631	1.520966147447	3.827792535766
H	-1.378412988762	-0.188929735004	2.556787452741
C	-1.656152075914	2.907978300291	3.911552668175
H	-1.719675525072	4.753443244854	2.793931801841
H	-1.549862204600	0.917721200339	4.730683516642
H	-1.725517783569	3.393554362760	4.880042104014

1 imaginary frequency

E = -827.942131

H = -827.566711

G = -827.632432

C5 (axial)

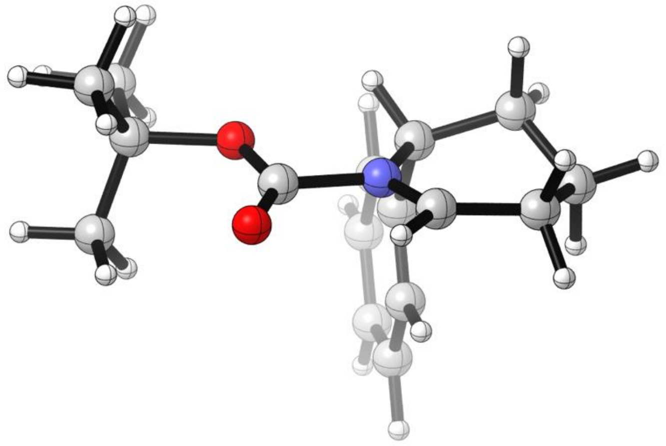

C	0.843355382779	-0.789647365689	0.907249765212
C	1.887981432217	-1.796118036232	1.429121200873
C	2.644084151842	-2.480559513225	0.288623731085
C	1.648017742108	-3.207086342856	-0.621585276217
C	0.360979364216	-2.496474441413	-0.806313060617
N	-0.009537822352	-1.450919099599	-0.138845002410
H	3.373484182277	-3.193775015288	0.681169970859
H	1.376440415437	-4.200407891100	-0.223478580357
H	-0.362981707355	-2.851154225096	-1.539059268549
H	2.574223041592	-1.250336827460	2.082346553402
C	-1.363318506159	-0.875174998244	-0.495169286402
O	-1.949208904095	-1.321580834286	-1.444102422515
O	-1.660763833330	0.049697811295	0.363801118763
C	-2.959145477133	0.866552346937	0.249826787756
H	1.380669688630	-2.546813232607	2.049289192271
H	0.150281584460	-0.549528806480	1.714763333391
C	-4.136096574676	-0.090539573868	0.404020984167
H	-5.060096744268	0.496440313988	0.426009869647
H	-4.201990153562	-0.793634050904	-0.428805858826
H	-4.070204815407	-0.645282771250	1.345378960967
C	-2.938338870031	1.601485222485	-1.086266268902
H	-3.798701165903	2.277602841948	-1.123535821358
H	-2.031255637202	2.205838208170	-1.185557399996
H	-3.012184846277	0.914704686884	-1.932001601170
C	-2.825192104952	1.816568427674	1.433863318311
H	-3.700940272129	2.471753806525	1.467835250895
H	-2.775929850794	1.266093145710	2.378356243008
H	-1.932523189850	2.441830364002	1.338441561917
H	3.202207459412	-1.736338073452	-0.289076268620

H	2.053384023295	-3.422126254082	-1.620257232756
C	1.424438870993	0.511329825578	0.369034322534
C	1.589143779861	1.575044731589	1.267237406486
C	1.843482923346	0.669279346318	-0.957833464586
C	2.178878037141	2.767209914983	0.850912901783
H	1.260381736148	1.468364463134	2.298552363846
C	2.427004770103	1.867762367936	-1.376158862887
H	1.711285152611	-0.128639323399	-1.684333703166
C	2.598371117999	2.916665698079	-0.473338906542
H	2.305079190698	3.580986952812	1.558733218637
H	2.744696222415	1.978021025032	-2.408638599487
H	3.052166205896	3.847492175450	-0.799558170447

0 imaginary frequencies

E = -827.952556

H = -827.576636

G = -827.643854

C6 (equatorial)

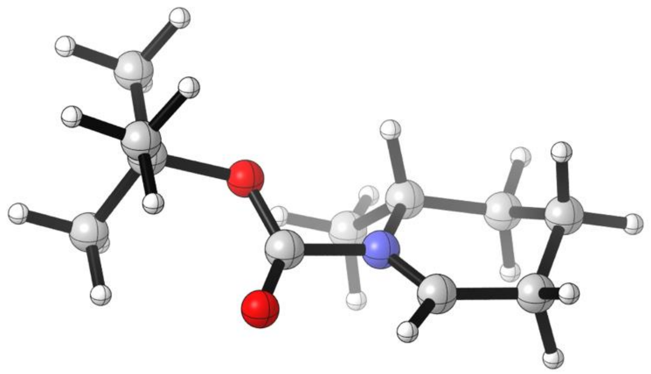

C	-1.473436100426	1.084551878025	0.016386150798
C	-2.998880573343	1.202473754450	-0.147189659170
C	-3.612525163671	0.089385471742	-0.995910985290
C	-3.291888833238	-1.261770506936	-0.348924087262
C	-1.861947635551	-1.371625574397	0.025865249499
N	-1.052540068449	-0.366336924685	0.126873397538
H	-3.212056010229	0.111902627908	-2.016568296514
H	-3.529628064551	-2.118111197263	-0.992843765441
H	-1.407649135443	-2.345907400037	0.201877265312
H	-3.464559953717	1.199135121559	0.846833556472
C	0.406173171249	-0.732936531574	0.326054533349
O	0.675468908329	-1.809438383483	0.783221619954

O	1.141146410769	0.243205941208	-0.114691244344
C	2.682009619166	0.151676792644	-0.114813410300
H	-3.202081275902	2.185766819648	-0.582790081717
H	-0.957724985090	1.451206168550	-0.875410516694
C	3.083408617739	-1.008416151388	-1.019195816789
H	4.174021277293	-1.011226883038	-1.115369943316
H	2.779129900600	-1.973057217823	-0.607888522113
H	2.660749181905	-0.888510296958	-2.021844818416
C	3.149902104295	-0.007590391635	1.327487328870
H	4.244180253047	0.027771535147	1.341467164911
H	2.783174773559	0.811481531641	1.954506783872
H	2.837341010604	-0.961632581242	1.756608884990
C	3.066485531901	1.502856605167	-0.704728810062
H	4.156829719740	1.565058005526	-0.772585355174
H	2.655121695913	1.626683603647	-1.710890823876
H	2.718383327973	2.326243239976	-0.073764053083
H	-4.695156900294	0.217693541334	-1.074178250351
H	-3.883108798395	-1.424421228288	0.567966710096
C	-1.002466726245	1.859451479630	1.253339302384
H	-1.429554897575	1.433778044656	2.167443676800
H	0.083548604370	1.889101382302	1.342373469904
H	-1.361181986336	2.889621723986	1.164769345161

0 imaginary frequencies

E = -636.213410

H = -635.893441

G = -635.951698

C6 (TS)

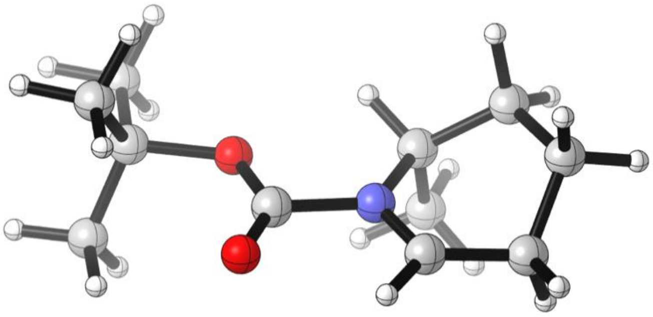

C	-1.002669416476	1.171784629179	0.256604588599
C	-2.350079201544	1.403171781135	-0.480347120562
C	-3.317545366818	0.196048300220	-0.516876326449
C	-2.895766899992	-0.954384386984	0.428305441633
C	-1.447257516936	-1.228580913259	0.293119851511
N	-0.586723590408	-0.269449262813	0.171033895192
H	-3.363414823758	-0.209855636465	-1.531761750407
H	-3.461606751079	-1.870256166598	0.239908062301
H	-1.034163851085	-2.235775583361	0.265937042939
H	-2.826714821095	2.259361840461	0.005504432000
C	0.862173677532	-0.676583535164	0.026020104518
O	1.130607004632	-1.846572973537	-0.006744081839
O	1.595188782979	0.393322208791	-0.036172104463
C	3.127360382199	0.310466082605	-0.199073891555
H	-2.132739102083	1.713276901756	-1.506239259574
H	-0.214555598019	1.719914075481	-0.255433147362
C	3.424461946076	-0.395435113404	-1.517436809477
H	4.504423813934	-0.358035483493	-1.693655263632
H	3.121778517170	-1.444250792815	-1.498380550049
H	2.932443741050	0.111573449879	-2.353580157473
C	3.692034096935	-0.407258097028	1.021637470389
H	4.785036477222	-0.378748134657	0.964918661601
H	3.392890598652	0.095938993802	1.946646266953
H	3.382954649520	-1.453758688960	1.059998406917
C	3.509043404404	1.784991184826	-0.233337095272
H	4.594364328205	1.870005184599	-0.343703039552
H	3.040611255040	2.296372063876	-1.079744444968
H	3.220806429723	2.290614707246	0.693160851252
H	-4.333754151871	0.502687444271	-0.258338514646
H	-3.088730360444	-0.681794495234	1.477709807024
C	-1.008352415997	1.596942580965	1.732724696390

H	-1.779677237746	1.081588566611	2.313475736880
H	-0.036025595454	1.411765705633	2.198202534428
H	-1.208708404468	2.670984562438	1.787604706753

1 imaginary frequency

E = -636.208822

H = -635.889329

G = -635.946476

C6 (axial)

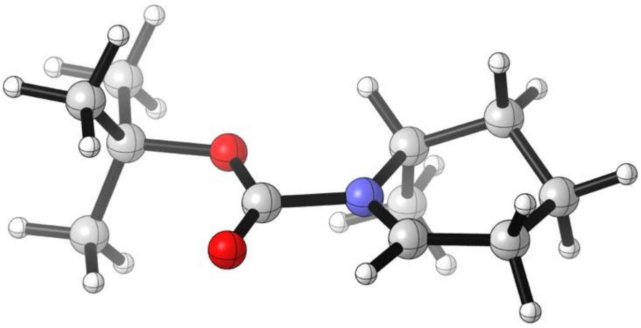

C	-1.409022020951	1.023009635186	-0.231318066058
C	-2.791266532039	1.052858918310	-0.899731386614
C	-3.811155296791	0.181597708213	-0.158122117575
C	-3.300562893695	-1.262382777749	-0.078686583884
C	-1.831641391812	-1.392305660090	0.054711141477
N	-0.989449668744	-0.412385615626	-0.023763974339
H	-4.776996642040	0.202086309061	-0.669551025466
H	-3.556621139158	-1.841268721609	-0.983161125965
H	-1.381942826605	-2.372633903999	0.208649515408
H	-3.124712605151	2.094318188640	-0.937280558486
C	0.472608883434	-0.784897256280	0.108155173362
O	0.758581055996	-1.921927662346	0.368009094768
O	1.195771252592	0.271412518178	-0.109335292645
C	2.736834206429	0.213682945106	-0.067097698568
H	-2.696005820569	0.713462567813	-1.939010829452
H	-0.662170118896	1.430653736918	-0.913529914788
C	3.202889786508	-0.754977369529	-1.148484407816
H	4.295943207399	-0.717094211338	-1.198764847141
H	2.910514627222	-1.784049637968	-0.930276563646
H	2.813960842108	-0.464977031996	-2.129737135123
C	3.155426458247	-0.193441942223	1.341254292713
H	4.246480282944	-0.132773243454	1.409501784735
H	2.736850181447	0.488393725955	2.088507268798
H	2.859047805734	-1.217680818832	1.576188480303
C	3.103689803104	1.657468745285	-0.386760335840
H	4.193452001573	1.756183309393	-0.389352426283
H	2.733222918250	1.951021947419	-1.373531170454
H	2.702138243708	2.344664474508	0.364054092209
H	-3.983847887289	0.568461254267	0.851462869143
H	-3.758541635485	-1.836251735163	0.739634259464
C	-1.332790309469	1.773064578890	1.104013216210

H	-2.049795046080	1.384008772052	1.833339689775
H	-0.330954180254	1.715940426720	1.536416189259
H	-1.561648541667	2.828421826289	0.927884392519

0 imaginary frequencies

E = -636.216746

H = -635.896753

G = -635.954832

NMR spectra of products and *N*-acyliminium ions

^{1}H NMR

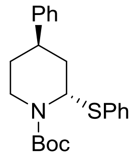

1a

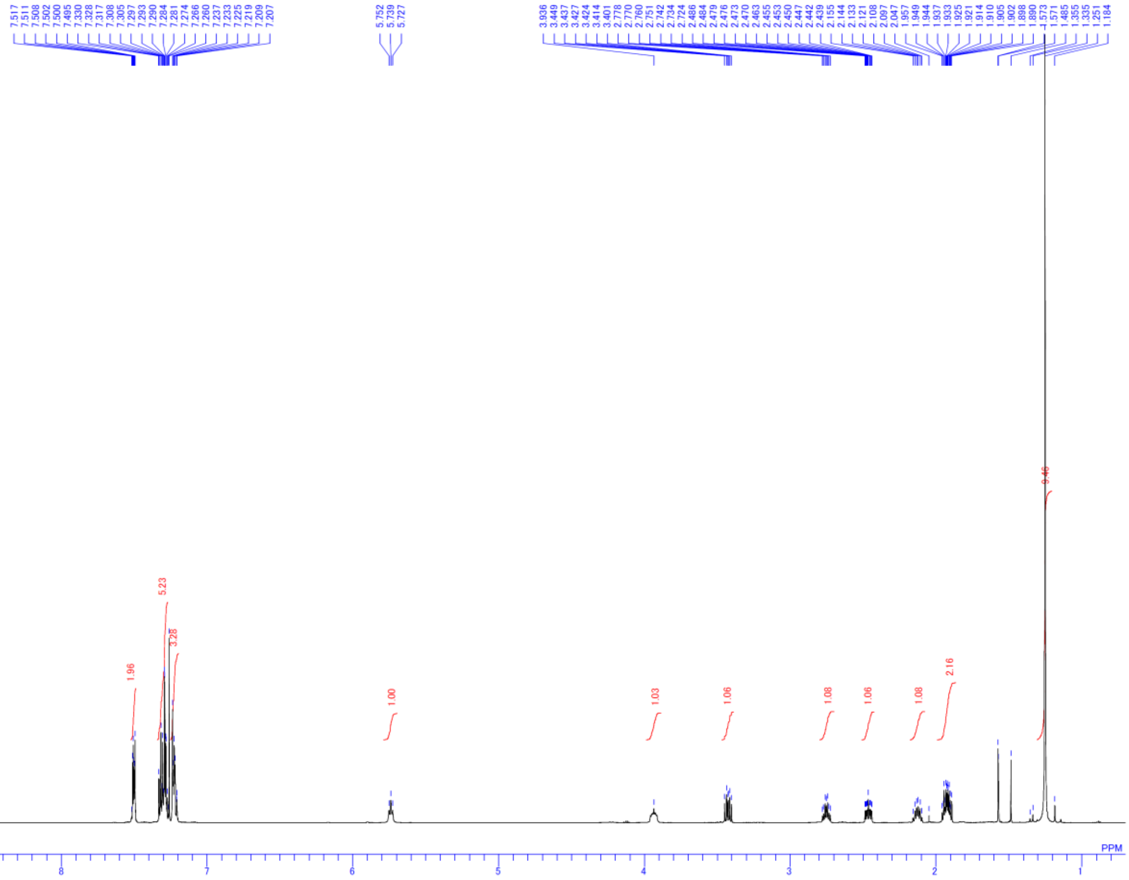

^{13}C NMR

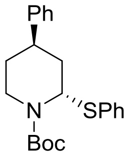

1a

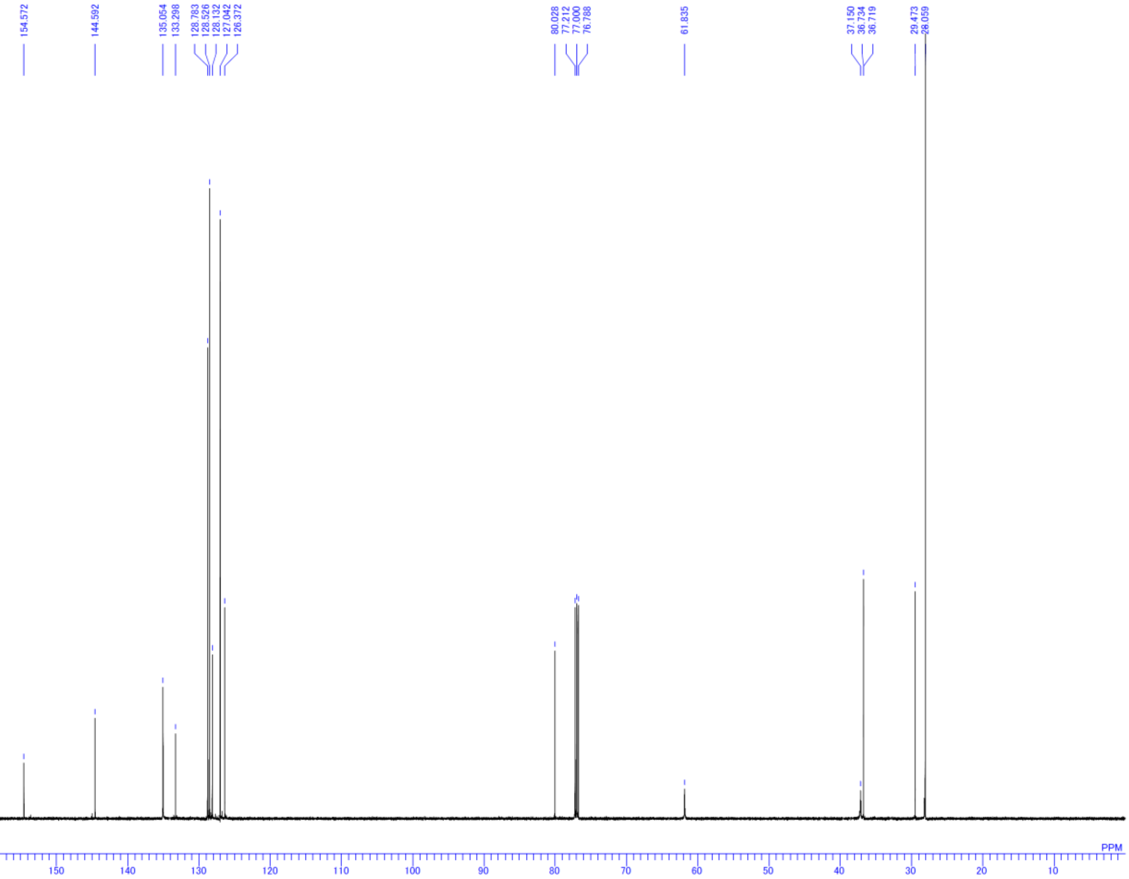

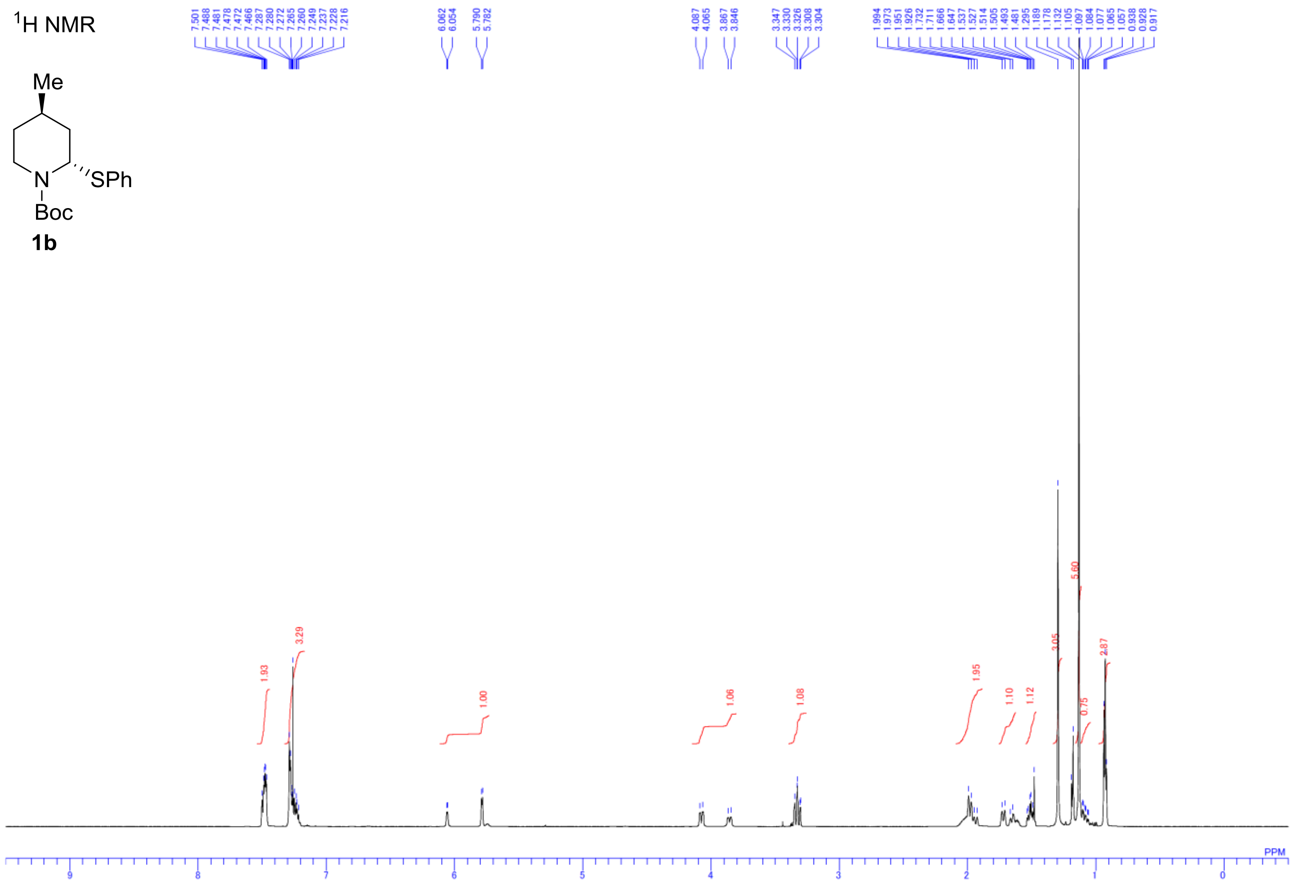
^{1}H NMR
Me
N
SPh
Boc
1b
PPM

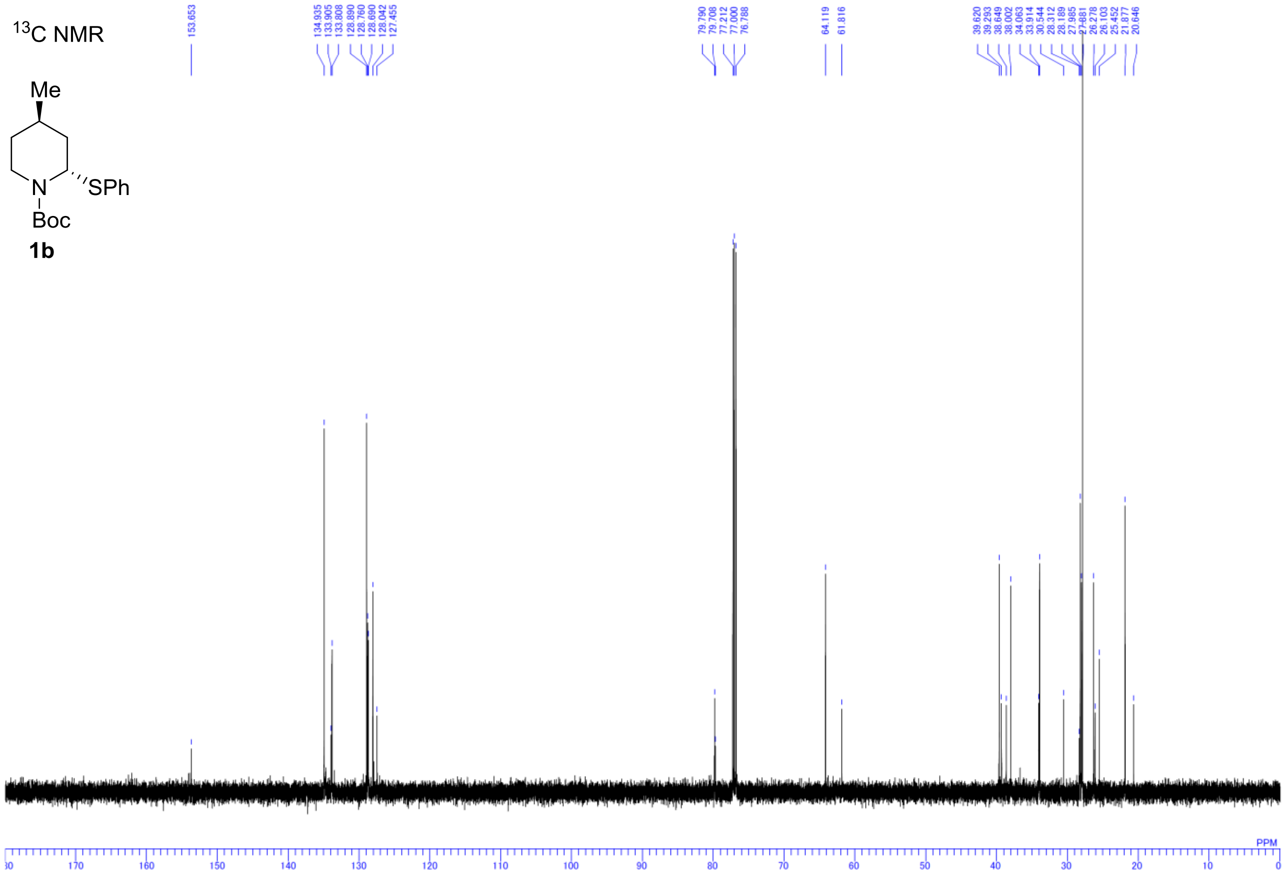
^{13}C NMR
Me
N
SPh
Boc
1b
PPM

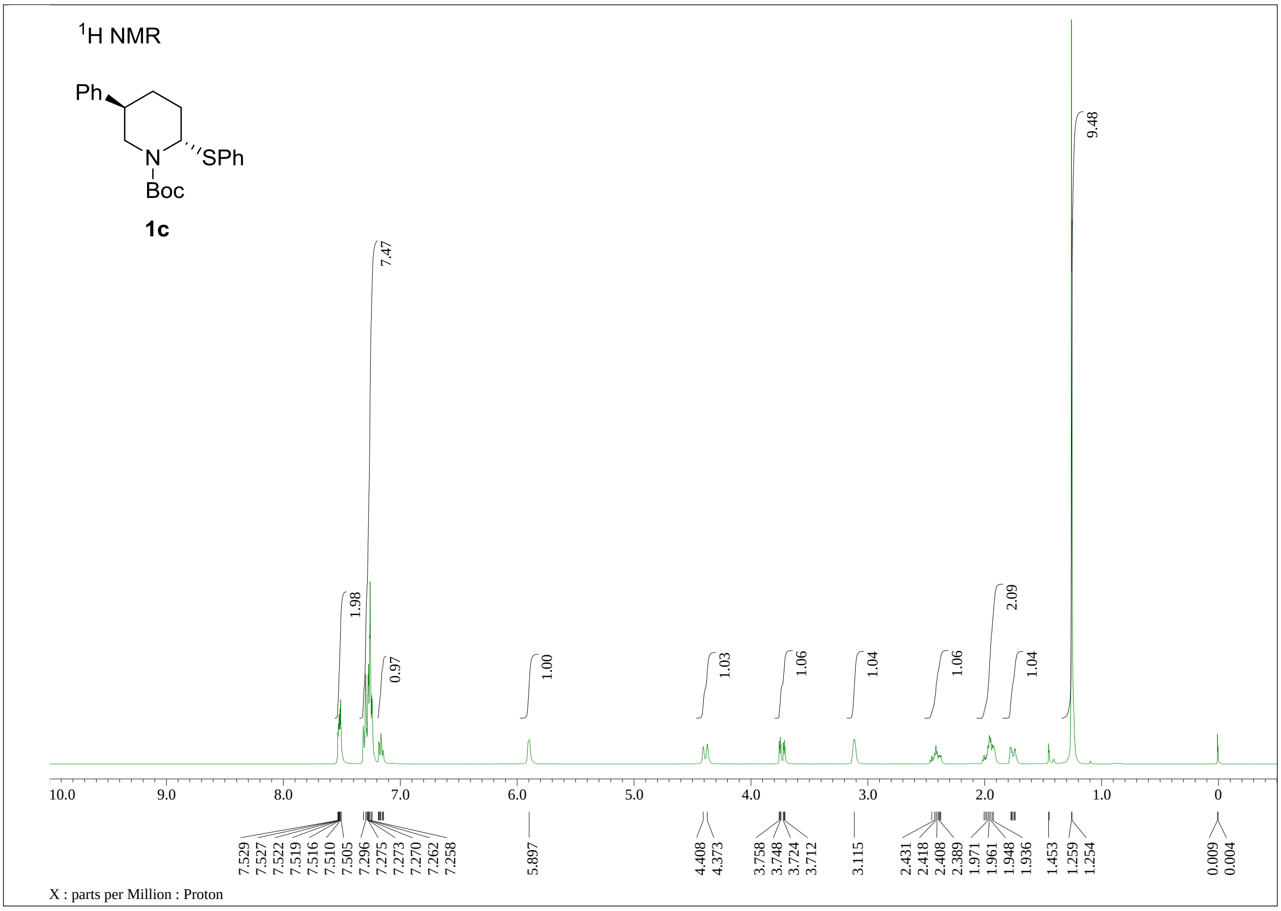

1H NMR
Ph
N
SPh
Boc
1c
9.48
7.47
1.98
0.97
1.00
1.03
1.06
1.04
1.06
2.09
1.04
10.0
9.0
8.0
7.0
6.0
5.0
4.0
3.0
2.0
1.0
0
7.529
7.527
7.522
7.519
7.516
7.510
7.505
7.296
7.275
7.273
7.270
7.262
7.258
5.897
4.408
4.373
3.758
3.748
3.724
3.712
3.115
2.431
2.418
2.408
2.389
1.971
1.961
1.948
1.936
1.453
1.259
1.254
0.009
0.004
X : parts per Million : Proton

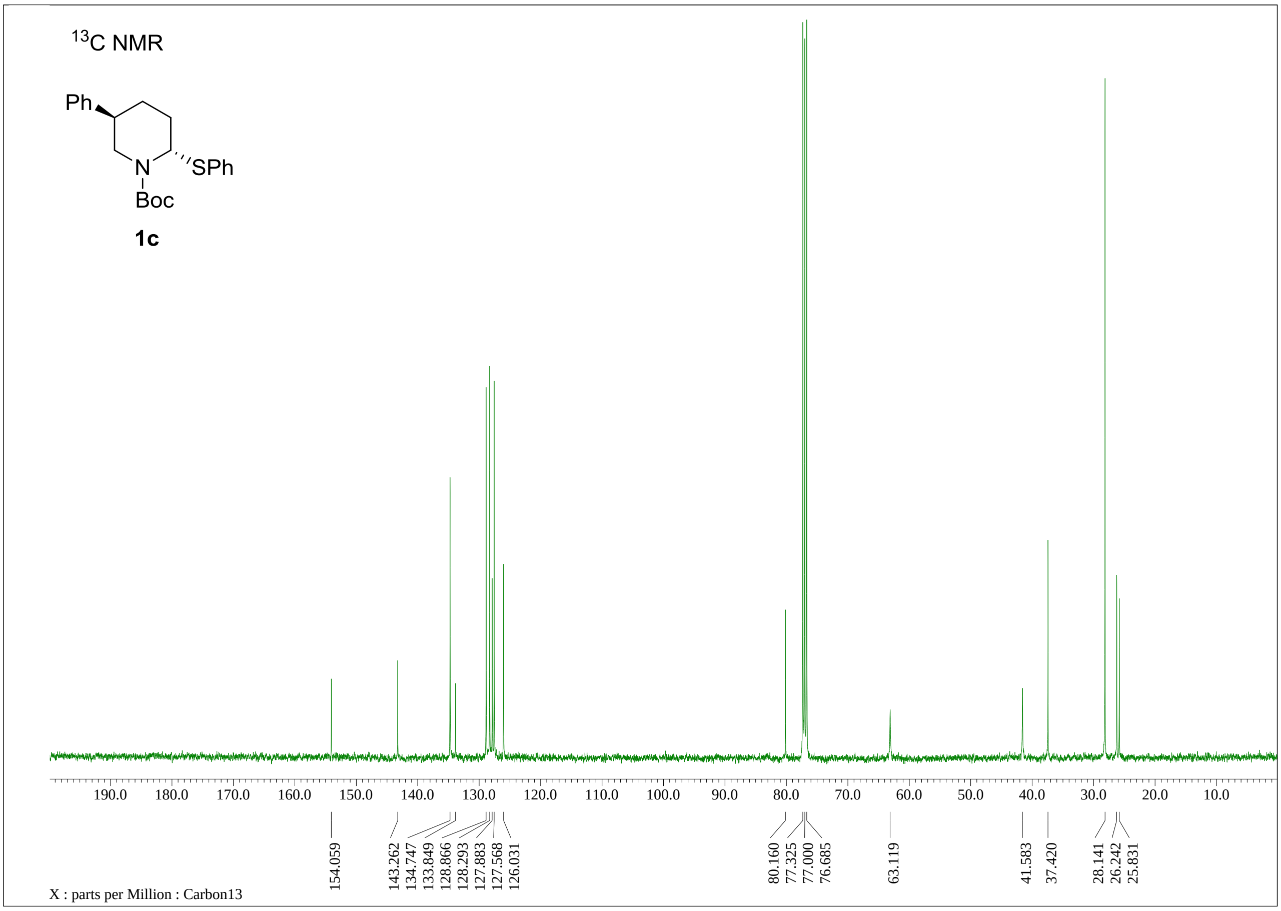

13C NMR
Ph
N
SPh
Boc
1c
190.0
180.0
170.0
160.0
150.0
140.0
130.0
120.0
110.0
100.0
90.0
80.0
70.0
60.0
50.0
40.0
30.0
20.0
10.0
154.059
143.262
134.747
133.849
128.866
128.293
127.883
127.568
126.031
80.160
77.325
77.000
76.685
63.119
41.583
37.420
28.141
26.242
25.831
X : parts per Million : Carbon13

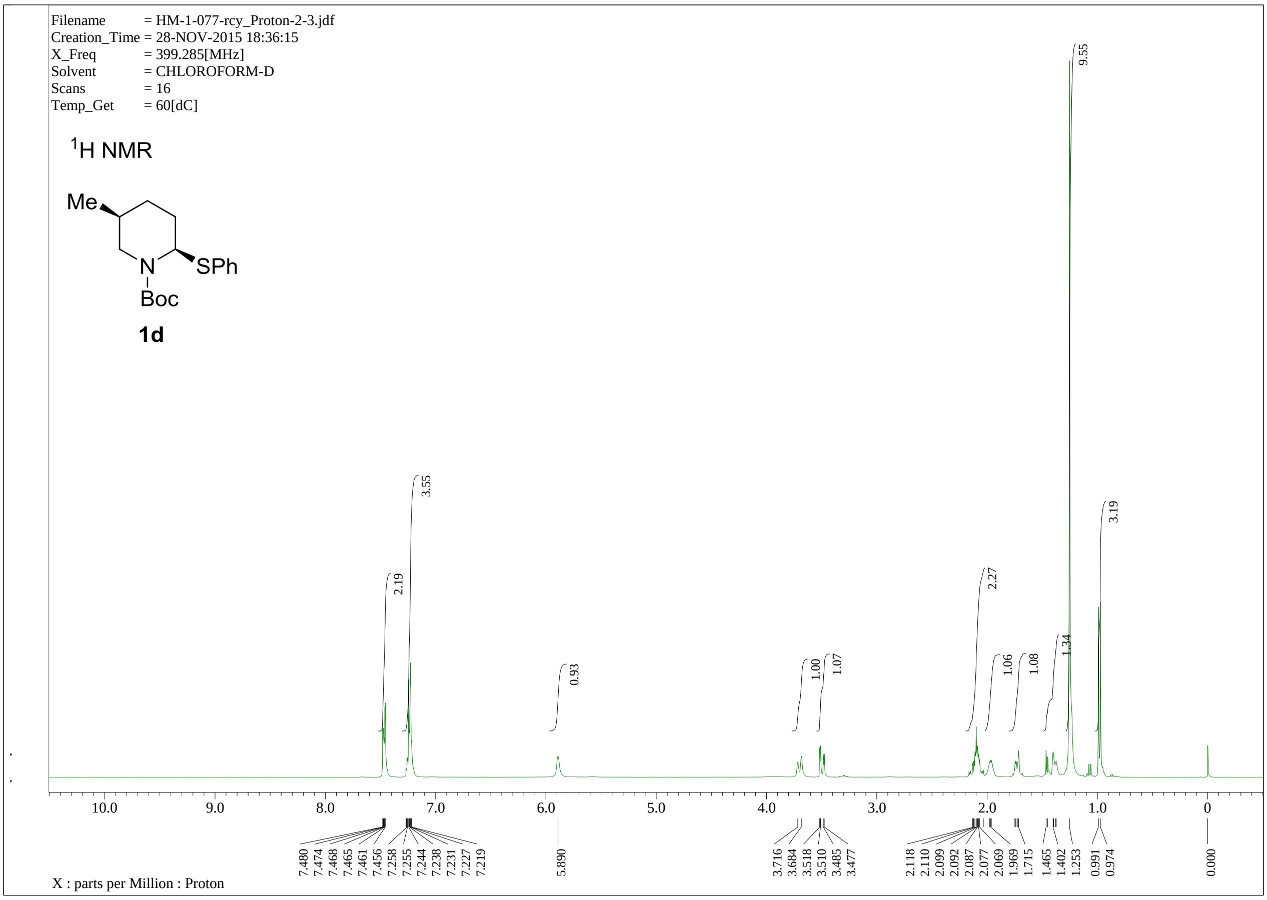
Filename = HM-1-077-rcy_Proton-2-3.jdf
Creation_Time = 28-NOV-2015 18:36:15
X_Freq = 399.285[MHz]
Solvent = CHLOROFORM-D
Scans = 16
Temp_Get = 60[dC]
1H NMR
Me
N
SPh
Boc
1d
X : parts per Million : Proton

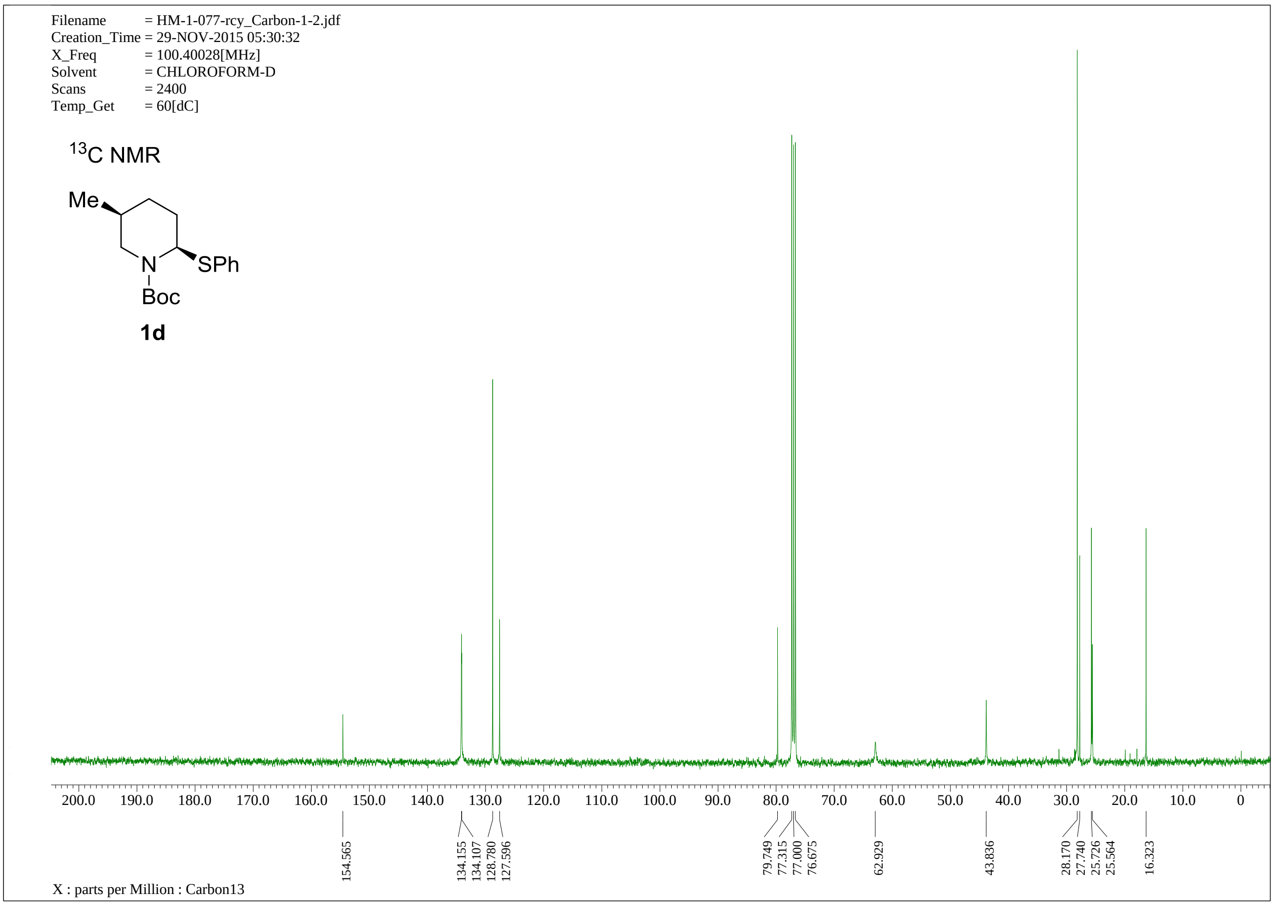
Filename = HM-1-077-rcy_Carbon-1-2.jdf
Creation_Time = 29-NOV-2015 05:30:32
X_Freq = 100.40028[MHz]
Solvent = CHLOROFORM-D
Scans = 2400
Temp_Get = 60[dC]
13C NMR
Me
N
SPh
Boc
1d
X : parts per Million : Carbon13

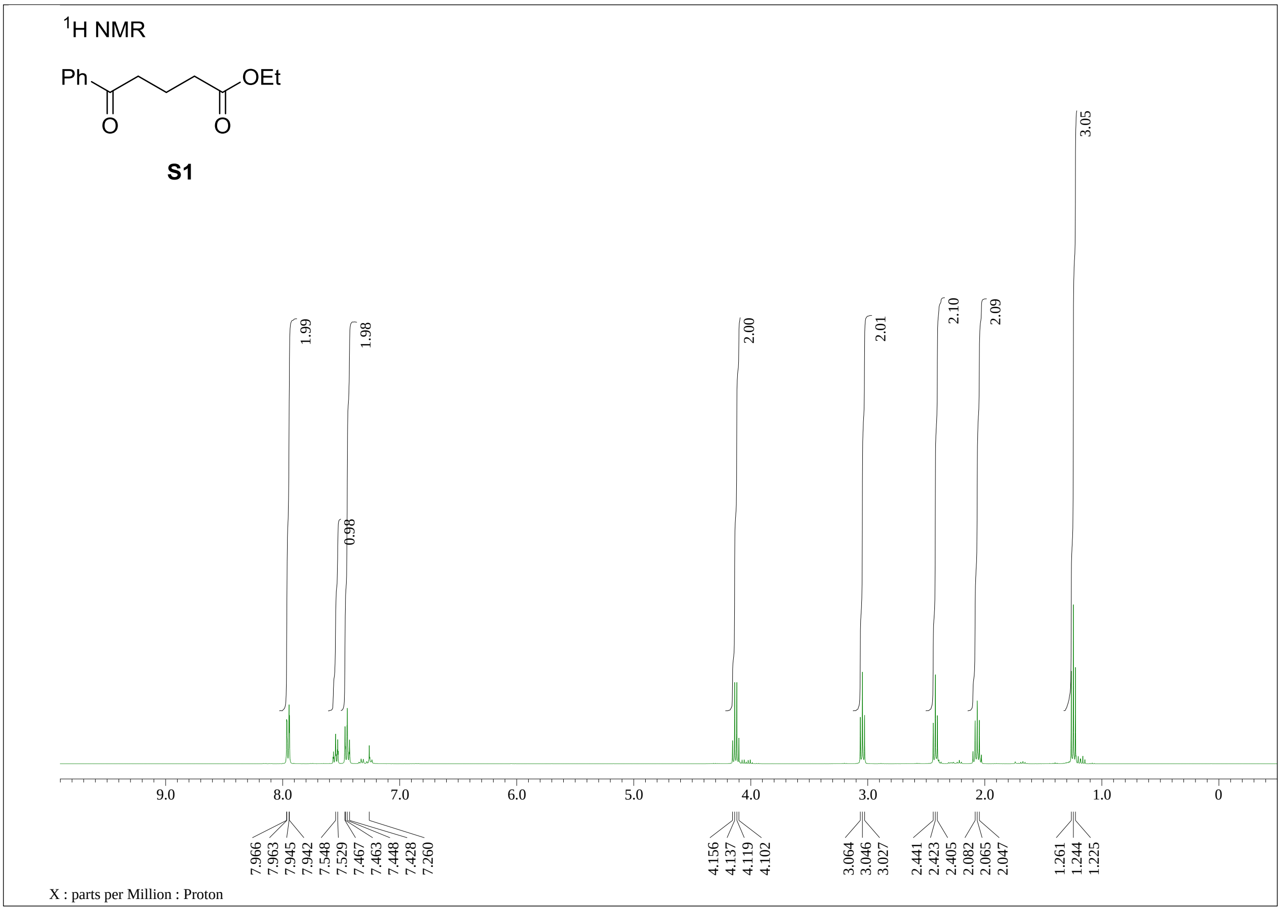

^{1}H NMR
Ph
OEt
O
O
S1
X : parts per Million : Proton

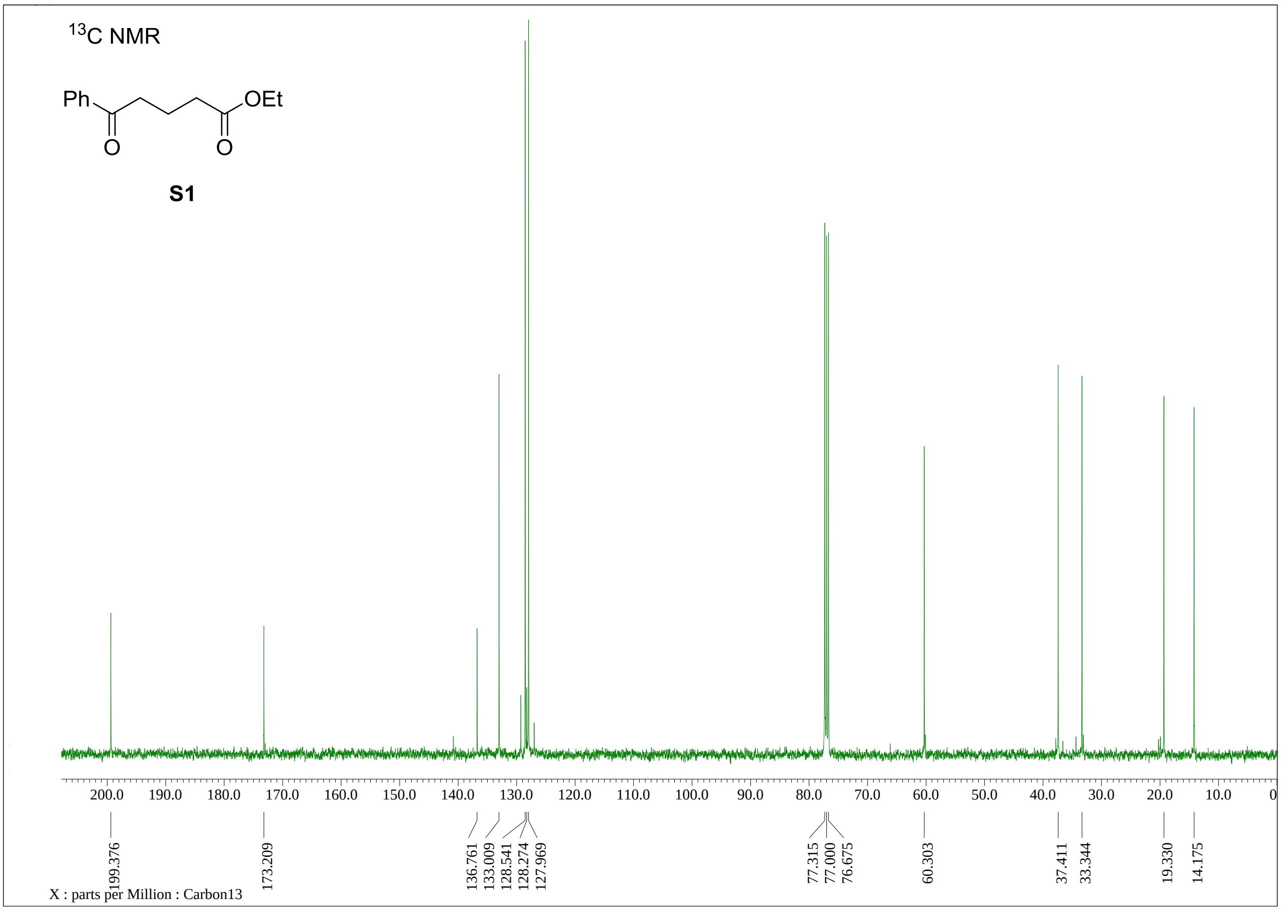

^{13}C NMR
Ph
OEt
O
O
S1
X : parts per Million : Carbon13

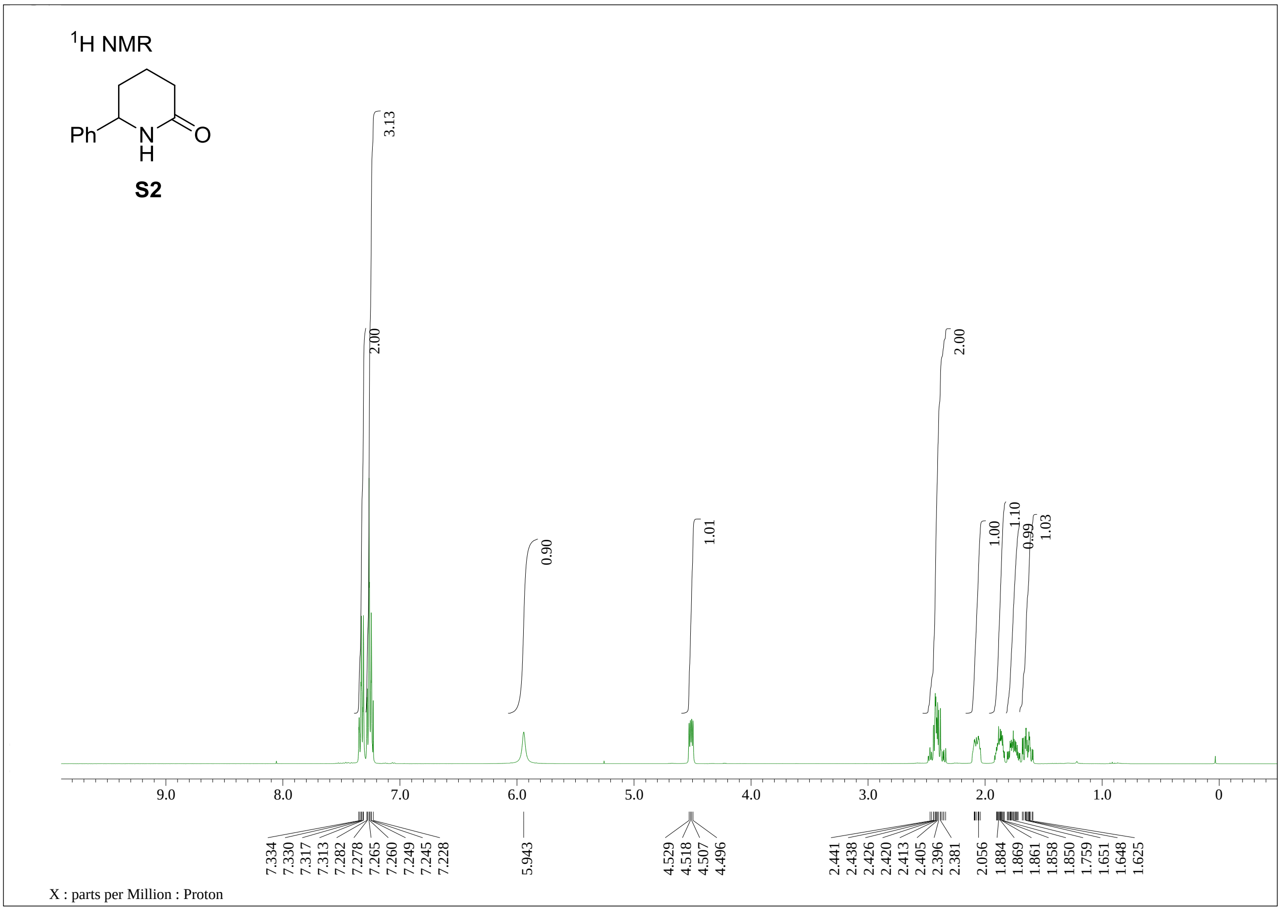
1H NMR
Ph
N
H
O
S2
X : parts per Million : Proton
9.0
8.0
7.0
6.0
5.0
4.0
3.0
2.0
1.0
0
3.13
2.00
0.90
1.01
2.00
1.00
1.10
0.99
1.03
7.334
7.330
7.317
7.313
7.282
7.278
7.265
7.260
7.249
7.245
7.228
5.943
4.529
4.518
4.507
4.496
2.441
2.438
2.426
2.420
2.413
2.405
2.396
2.381
2.056
1.884
1.869
1.861
1.858
1.850
1.759
1.651
1.648
1.625

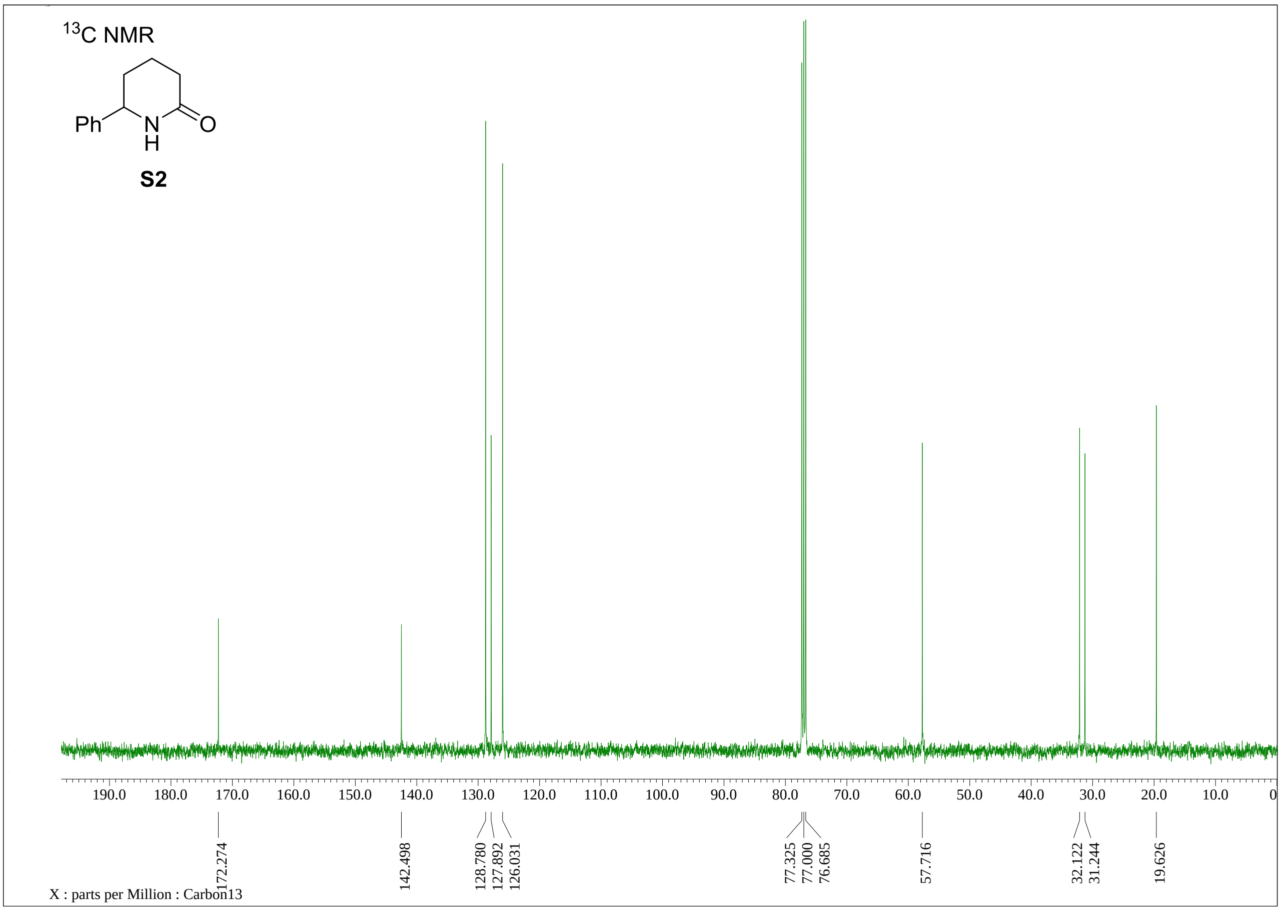
13C NMR
Ph
N
H
O
S2
X : parts per Million : Carbon13
190.0
180.0
170.0
160.0
150.0
140.0
130.0
120.0
110.0
100.0
90.0
80.0
70.0
60.0
50.0
40.0
30.0
20.0
10.0
0
172.274
142.498
128.780
127.892
126.031
77.325
77.000
76.685
57.716
32.122
31.244
19.626

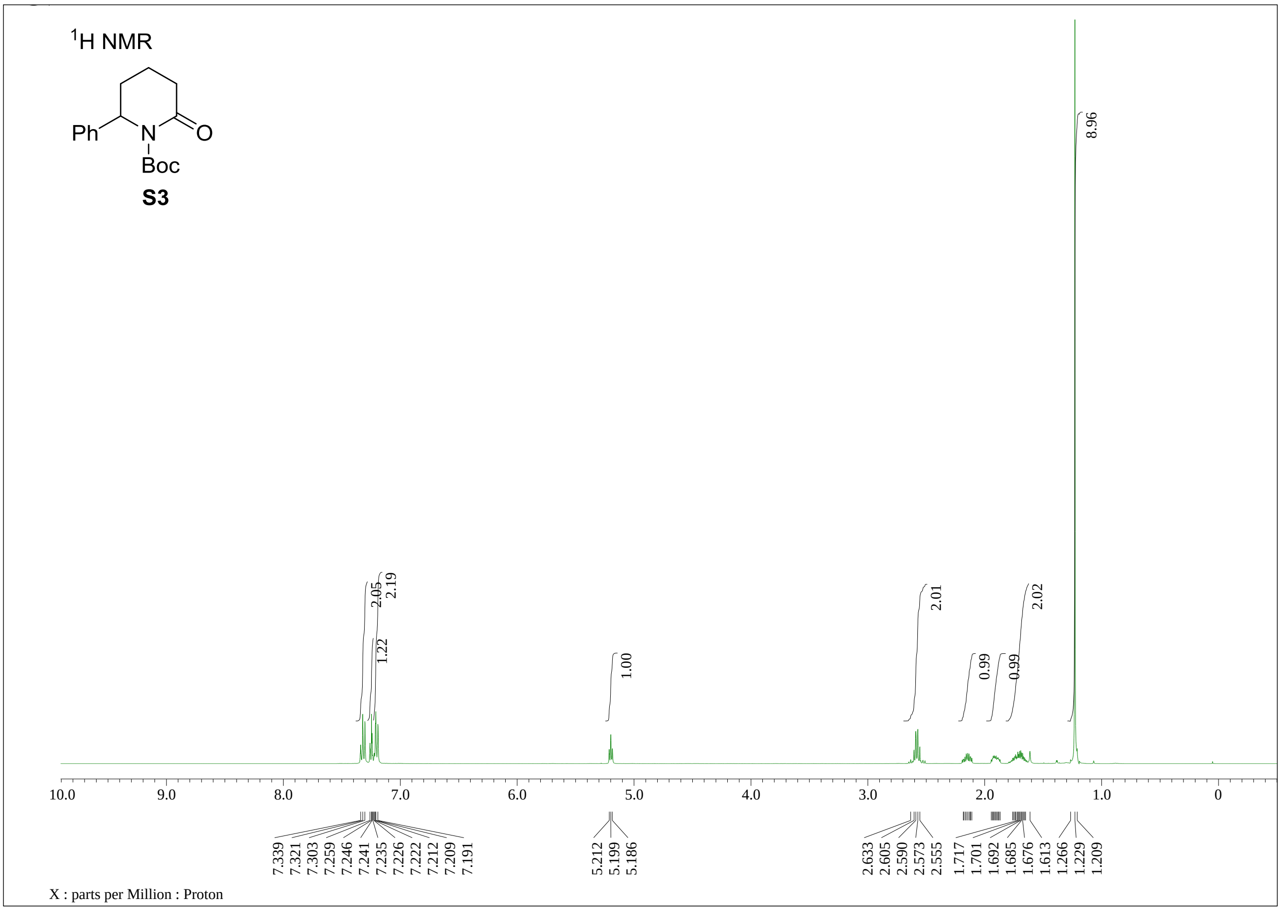

1H NMR
Ph
N
O
Boc
S3
X : parts per Million : Proton

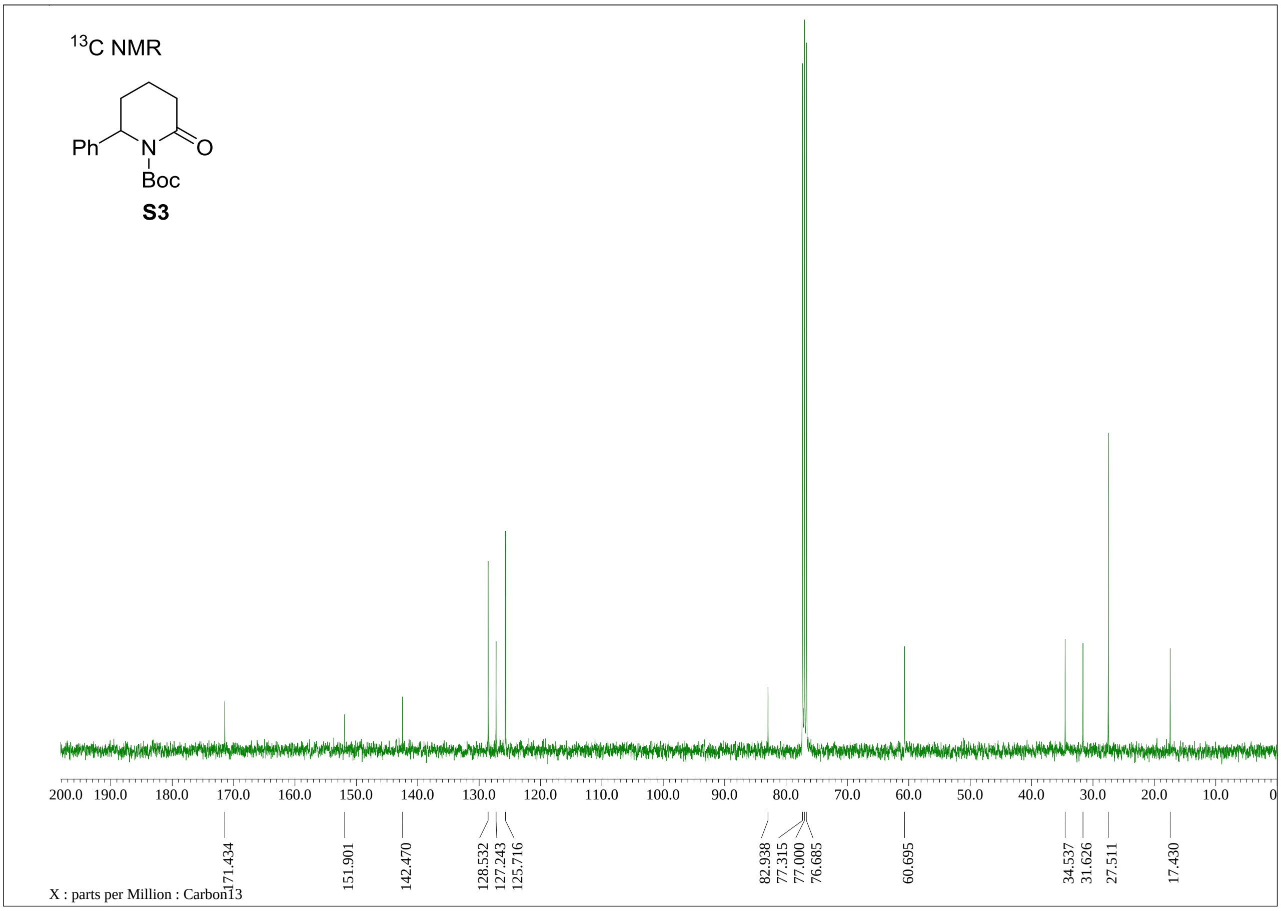

13C NMR
Ph
N
O
Boc
S3
X : parts per Million : Carbon13

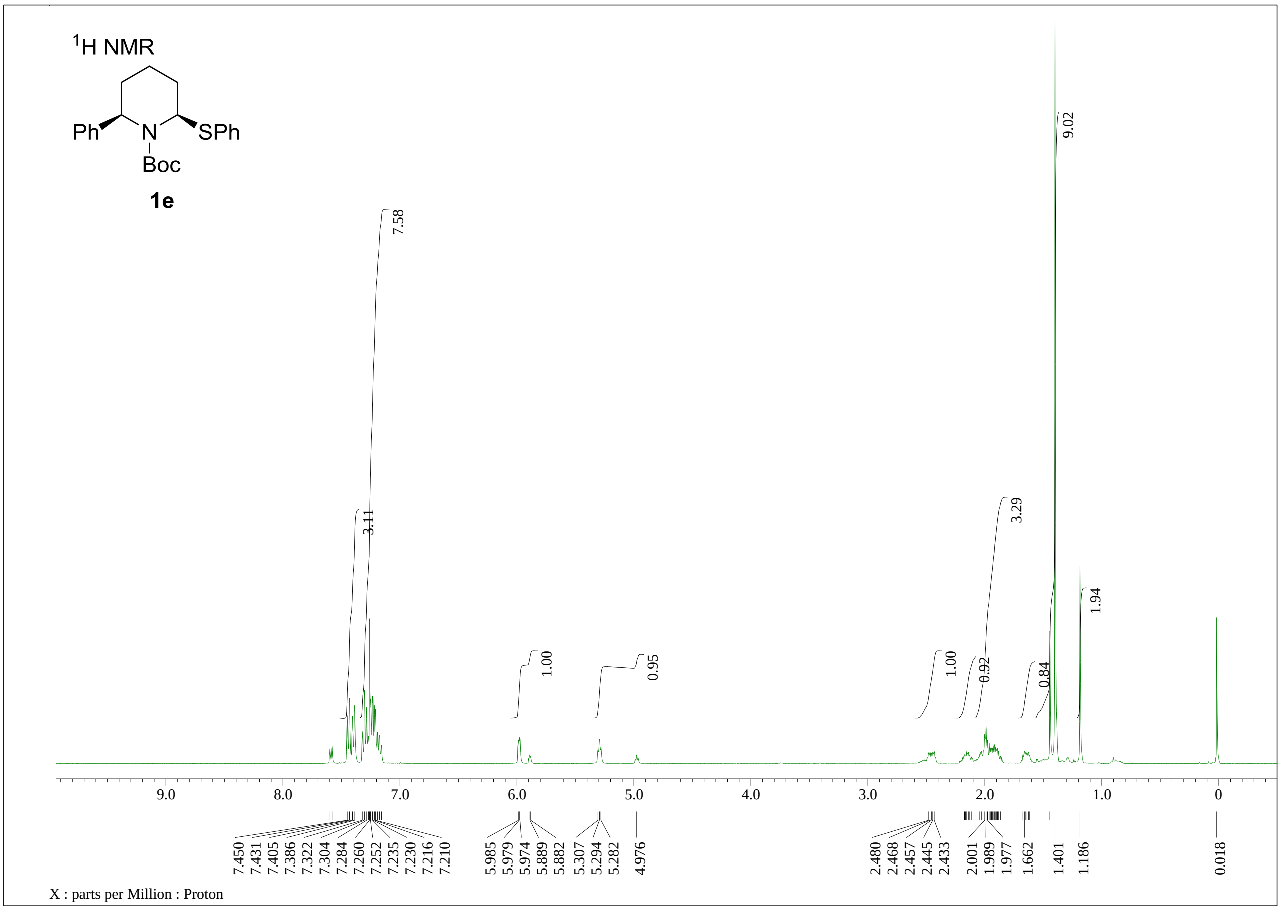

1H NMR
Ph
N
SPh
Boc
1e
7.58
3.11
1.00
0.95
1.00
0.92
3.29
0.84
9.02
1.94
9.0
8.0
7.0
6.0
5.0
4.0
3.0
2.0
1.0
0
7.450
7.431
7.405
7.386
7.322
7.304
7.284
7.260
7.252
7.235
7.230
7.216
7.210
5.985
5.979
5.974
5.889
5.882
5.307
5.294
5.282
4.976
2.480
2.468
2.457
2.445
2.433
2.001
1.989
1.977
1.662
1.401
1.186
0.018
X : parts per Million : Proton

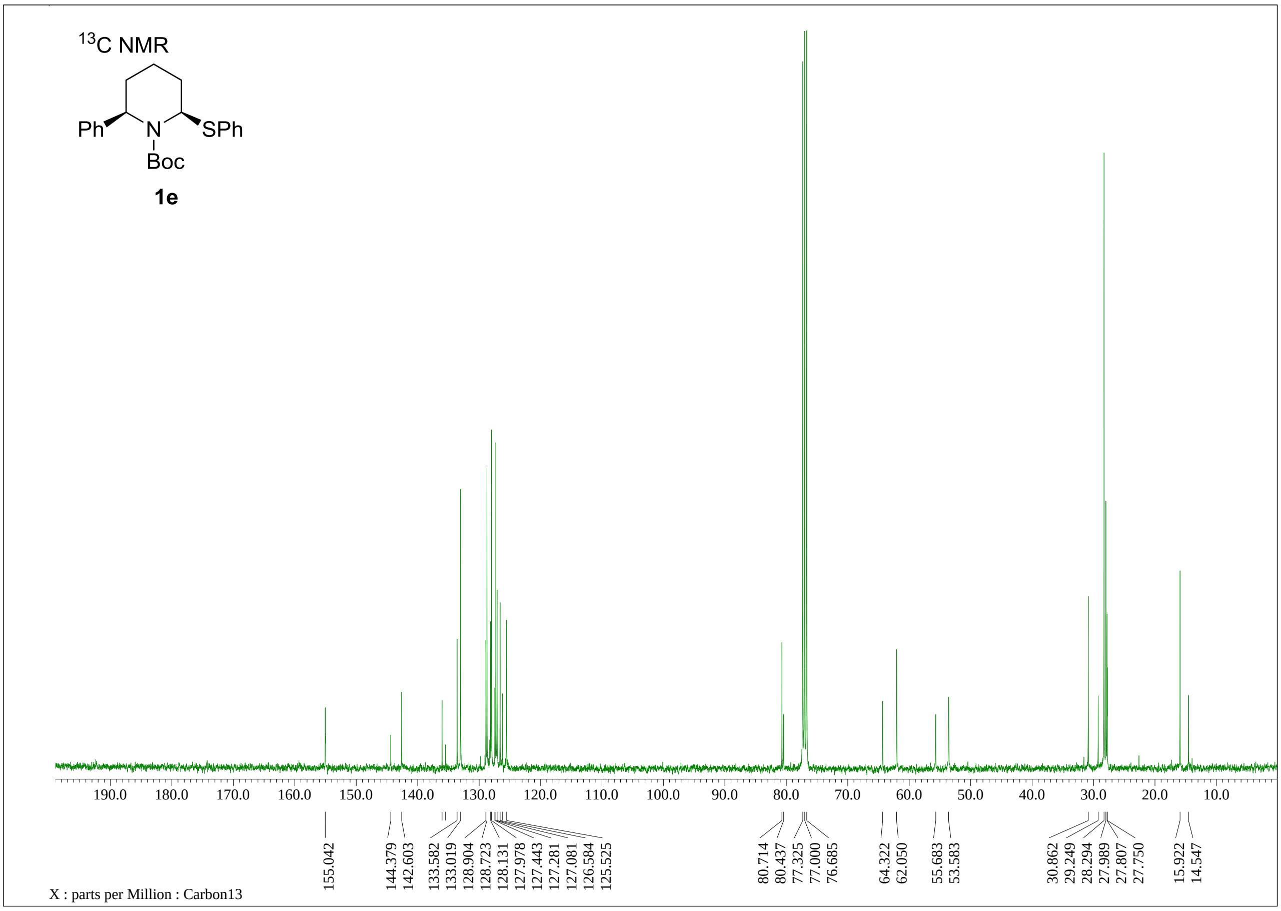

13C NMR
Ph
N
SPh
Boc
1e
190.0
180.0
170.0
160.0
150.0
140.0
130.0
120.0
110.0
100.0
90.0
80.0
70.0
60.0
50.0
40.0
30.0
20.0
10.0
155.042
144.379
142.603
133.582
133.019
128.904
128.723
128.131
127.978
127.443
127.281
127.081
126.584
125.525
80.714
80.437
77.325
77.000
76.685
64.322
62.050
55.683
53.583
30.862
29.249
28.294
27.989
27.807
27.750
15.922
14.547
X : parts per Million : Carbon13

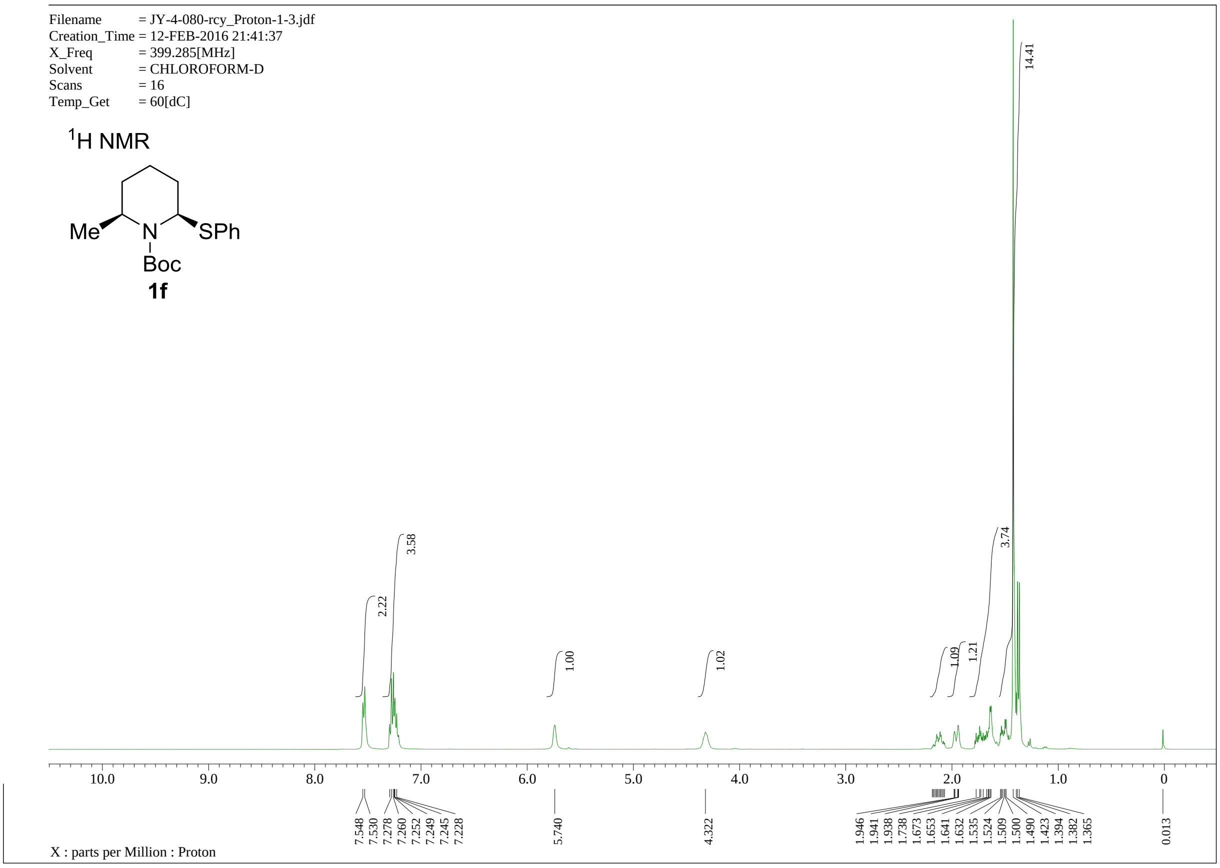
Filename = JY-4-080-rcy_Proton-1-3.jdf
Creation_Time = 12-FEB-2016 21:41:37
X_Freq = 399.285[MHz]
Solvent = CHLOROFORM-D
Scans = 16
Temp_Get = 60[dC]
1H NMR
Me
N
SPh
Boc
1f
2.22
3.58
1.00
1.02
1.09
1.21
3.74
14.41
10.0 9.0 8.0 7.0 6.0 5.0 4.0 3.0 2.0 1.0 0
7.548 7.530 7.278 7.260 7.252 7.249 7.245 7.228
5.740
4.322
1.946 1.941 1.938 1.738 1.673 1.653 1.641 1.632 1.535 1.524 1.509 1.500 1.490 1.423 1.394 1.382 1.365
0.013
X : parts per Million : Proton

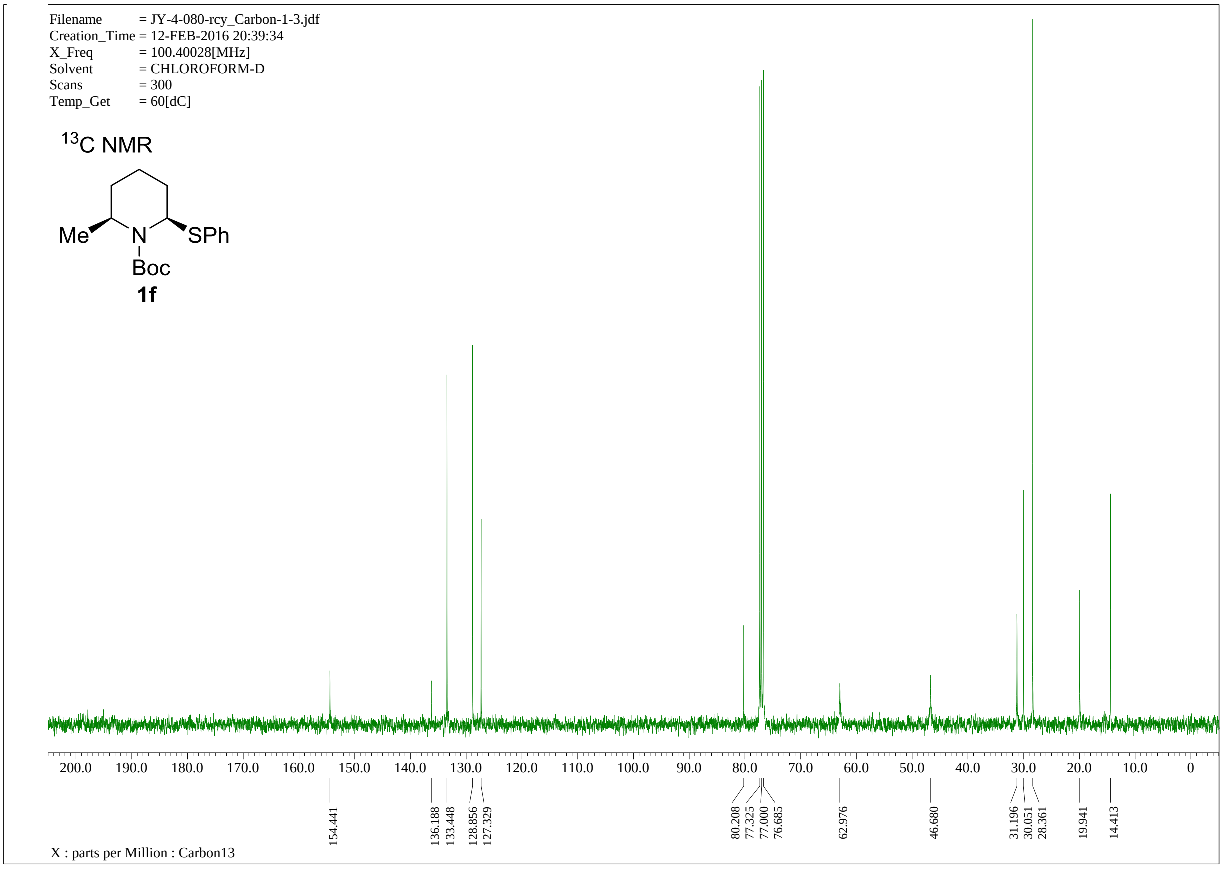
Filename = JY-4-080-rcy_Carbon-1-3.jdf
Creation_Time = 12-FEB-2016 20:39:34
X_Freq = 100.40028[MHz]
Solvent = CHLOROFORM-D
Scans = 300
Temp_Get = 60[dC]
13C NMR
Me
N
SPh
Boc
1f
200.0 190.0 180.0 170.0 160.0 150.0 140.0 130.0 120.0 110.0 100.0 90.0 80.0 70.0 60.0 50.0 40.0 30.0 20.0 10.0 0
154.441
136.188 133.448 128.856 127.329
80.208 77.325 77.000 76.685
62.976
46.680
31.196 30.051 28.361
19.941
14.413
X : parts per Million : Carbon13

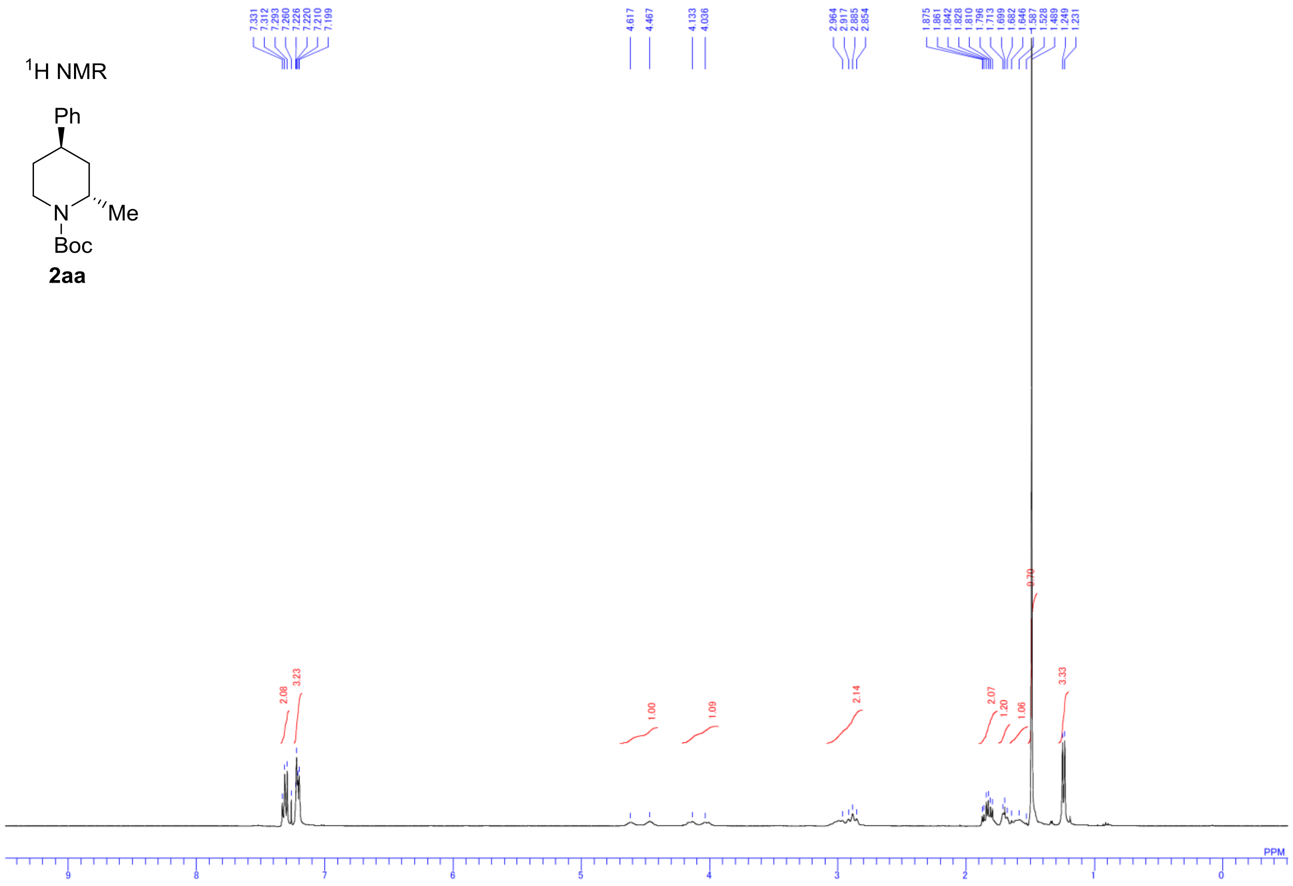

^{1}H NMR
Ph
N
Me
Boc
2aa

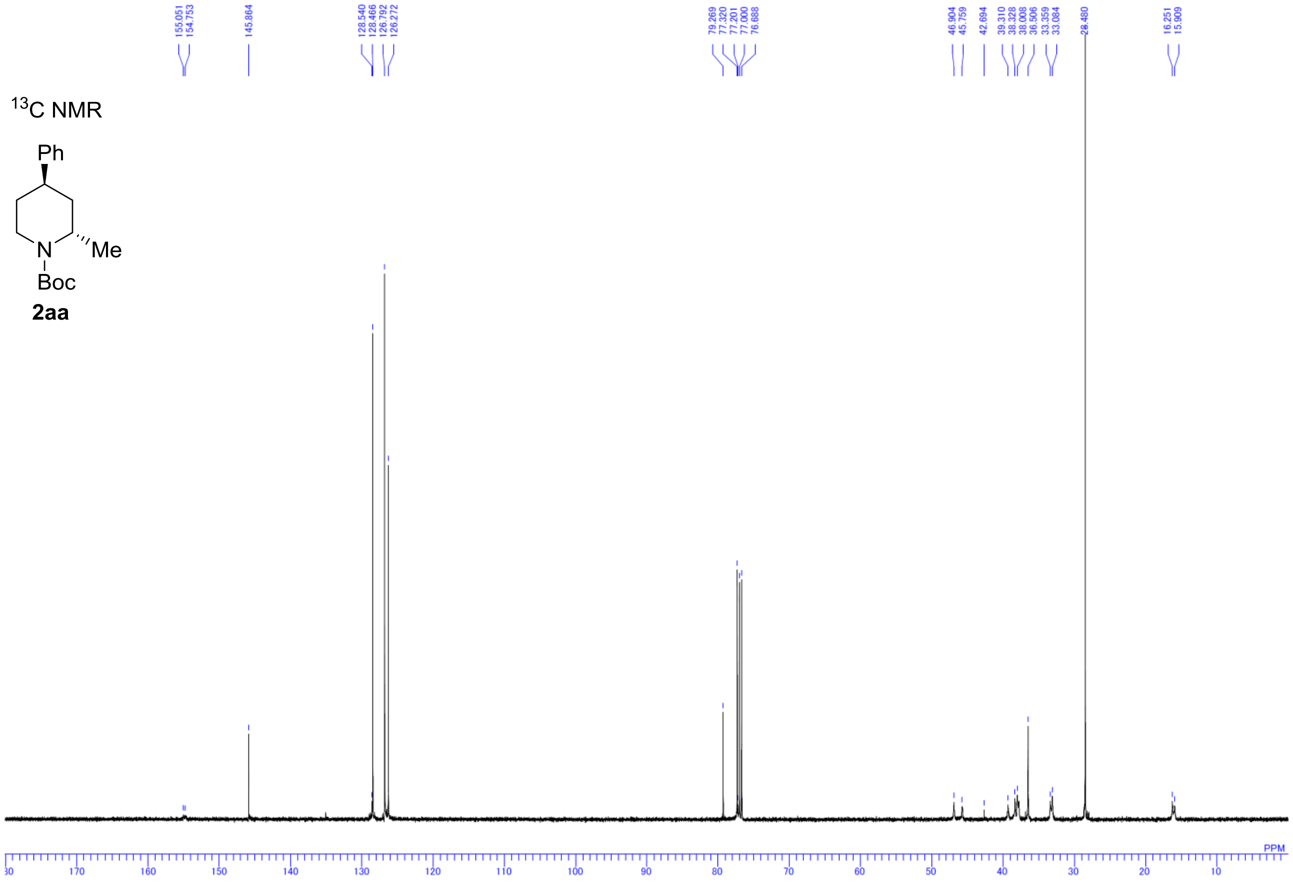

^{13}C NMR
Ph
N
Me
Boc
2aa

^{1}H NMR

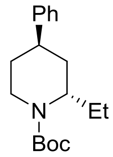

2ab

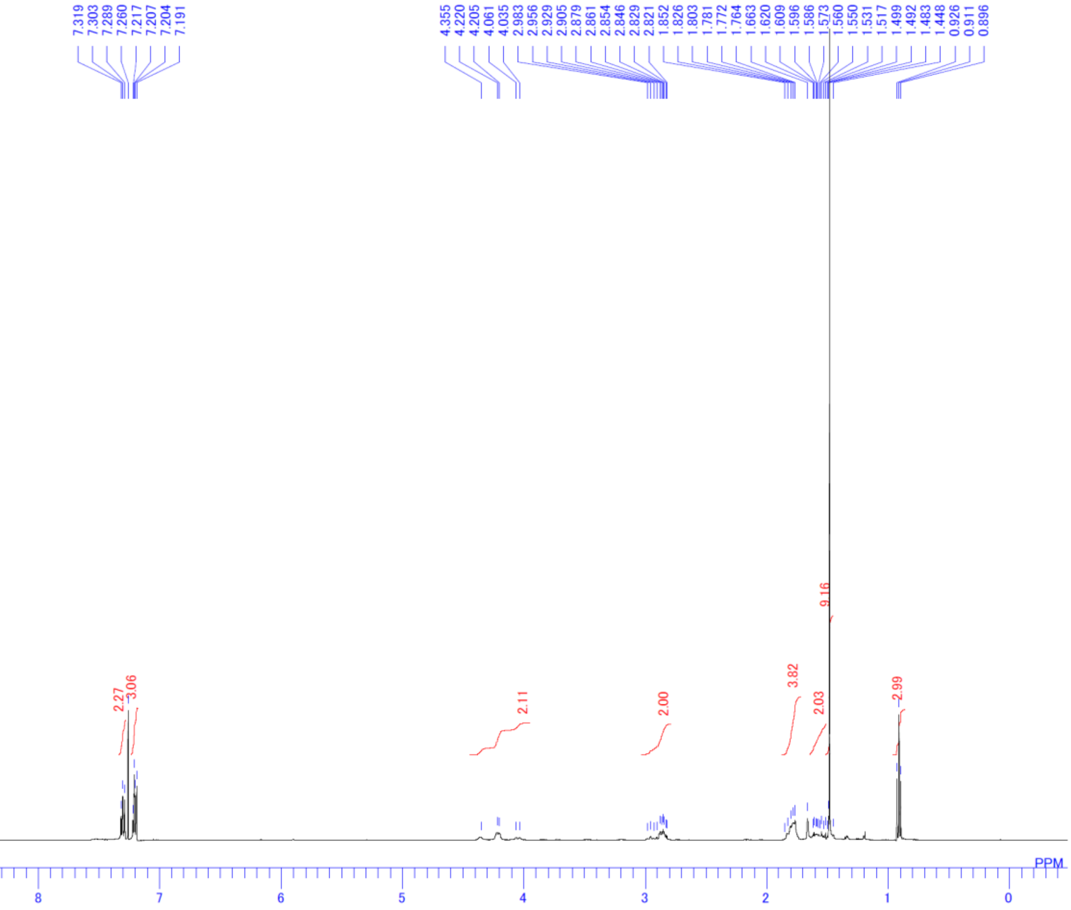

^{13}C NMR

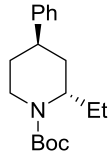

2ab

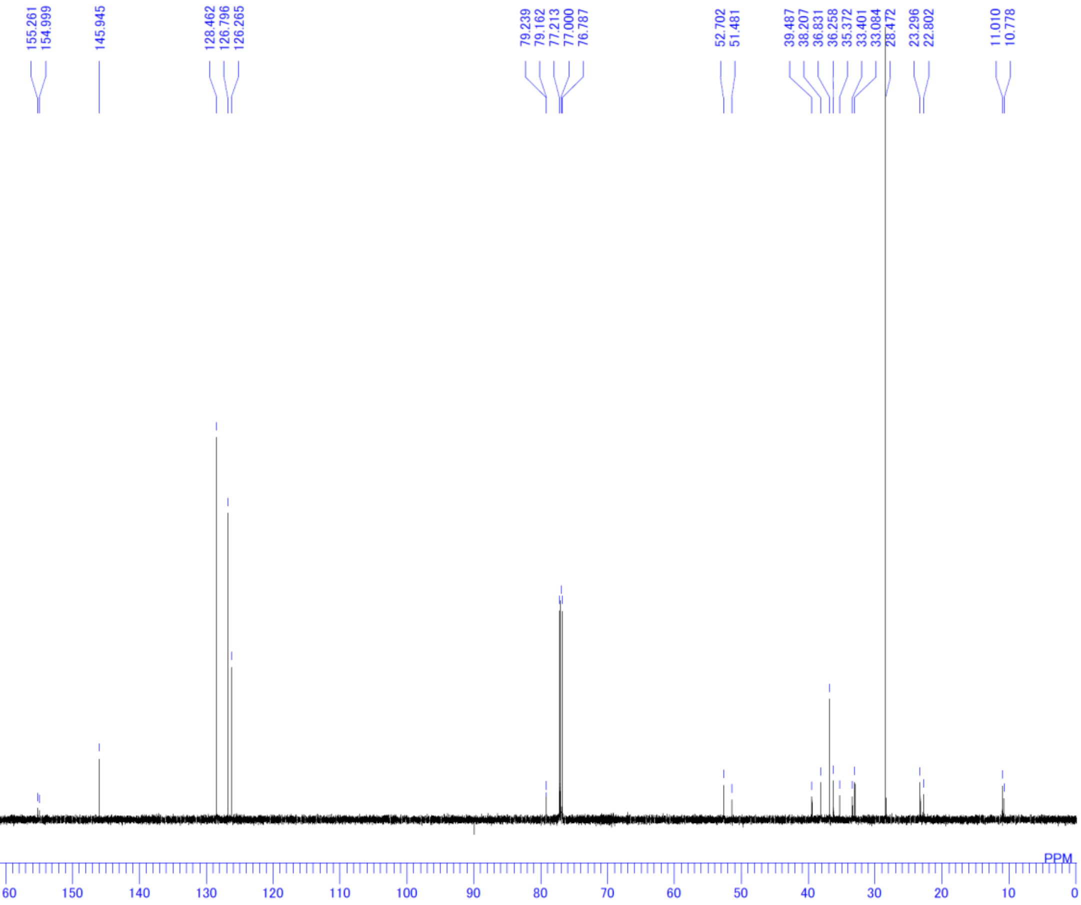

^1H NMR

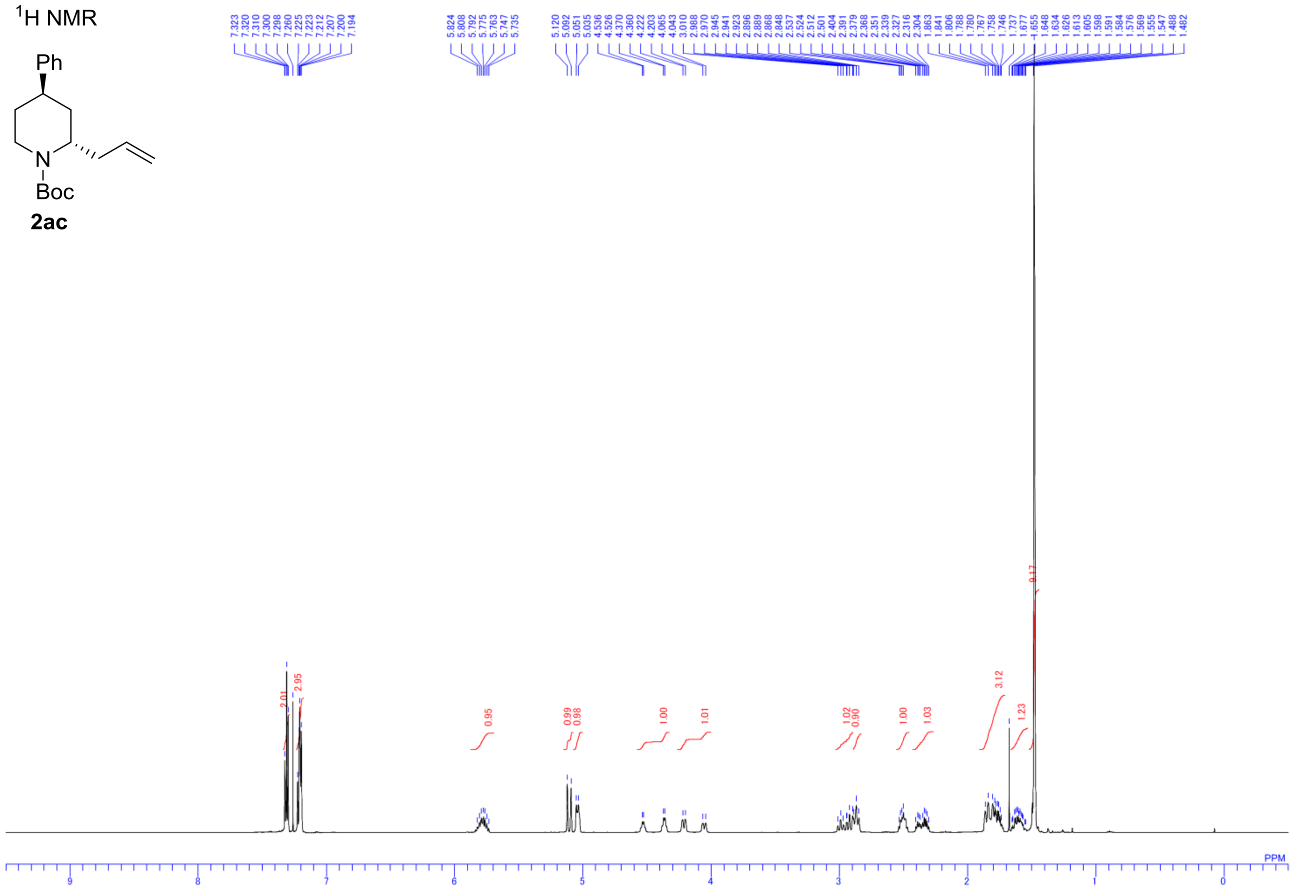

^{13}C NMR

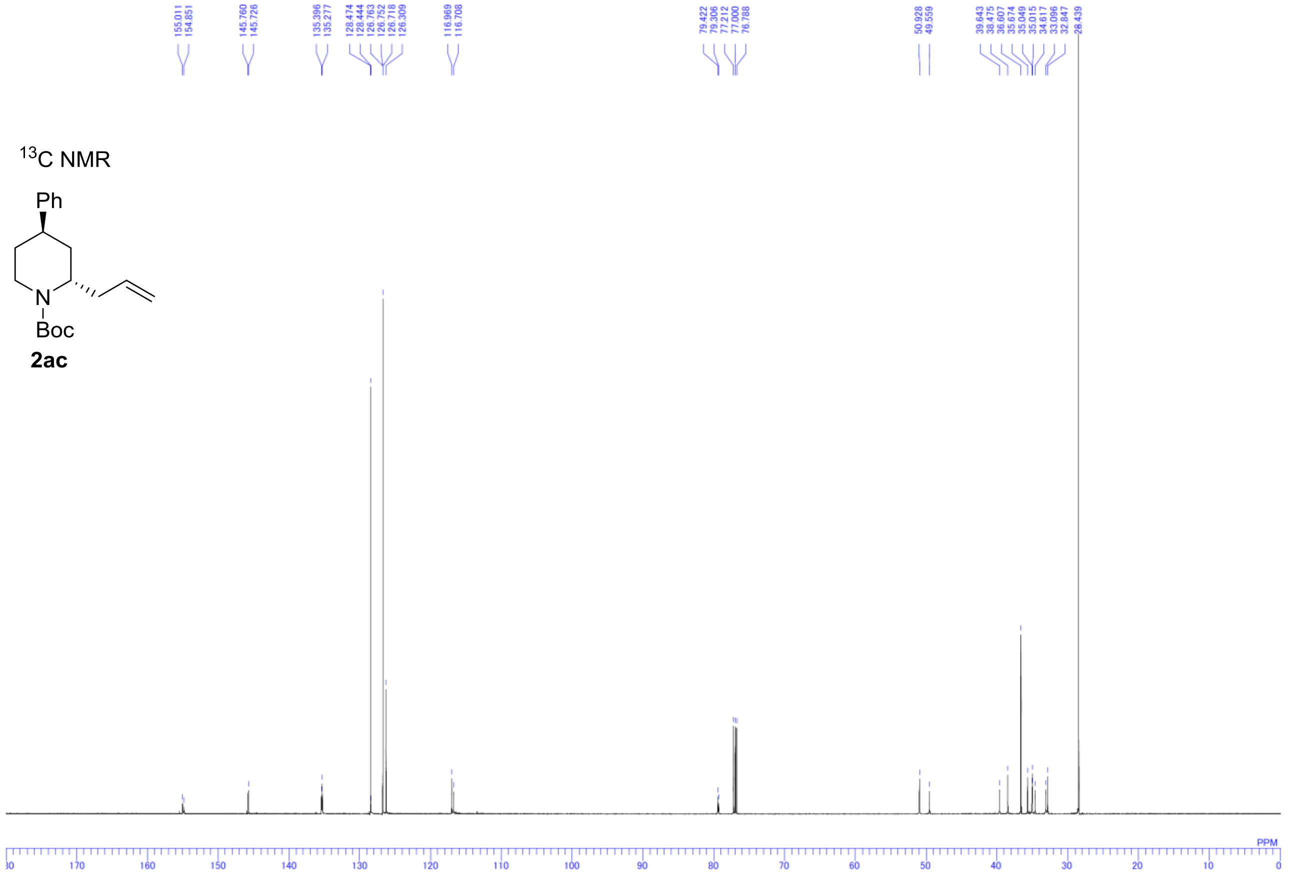

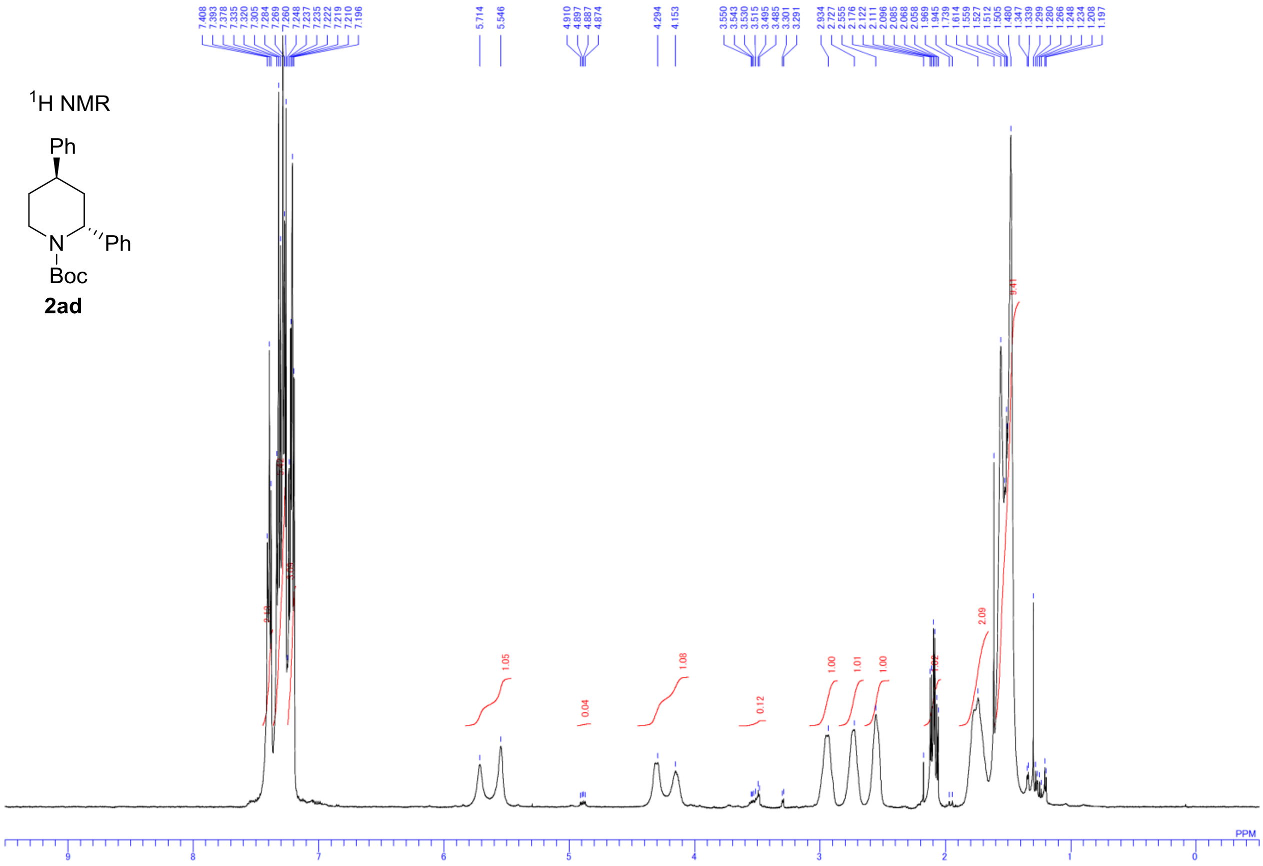

7.408
7.393
7.378
7.335
7.320
7.305
7.284
7.269
7.260
7.248
7.237
7.235
7.222
7.219
7.210
7.196
5.714
5.546
4.910
4.897
4.887
4.874
4.294
4.153
3.550
3.543
3.530
3.515
3.495
3.485
3.301
3.291
2.934
2.727
2.555
2.176
2.122
2.111
2.096
2.085
2.068
2.058
1.969
1.945
1.739
1.614
1.559
1.527
1.512
1.505
1.480
1.347
1.339
1.299
1.280
1.266
1.248
1.234
1.208
1.197
^{1}H NMR
Ph
N
Ph
Boc
2ad
PPM

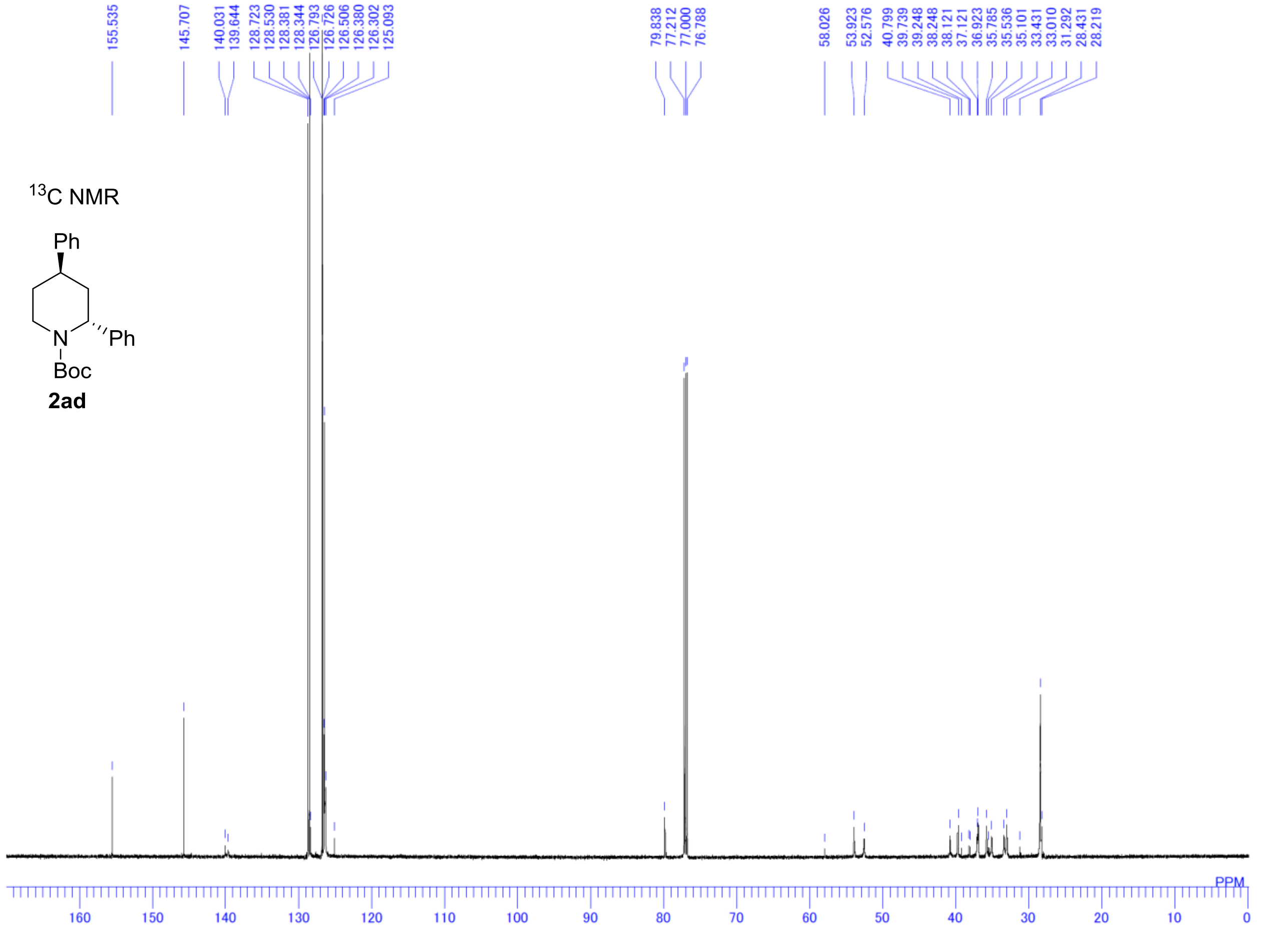

155.535
145.707
140.031
139.644
128.723
128.530
128.381
128.344
126.793
126.726
126.506
126.380
126.302
125.093
79.838
77.212
77.000
76.788
58.026
53.923
52.576
40.799
39.739
39.248
38.248
38.121
37.121
36.923
35.785
35.536
35.101
33.431
33.010
31.292
28.431
28.219
^{13}C NMR
Ph
N
Ph
Boc
2ad
PPM
160 150 140 130 120 110 100 90 80 70 60 50 40 30 20 10 0

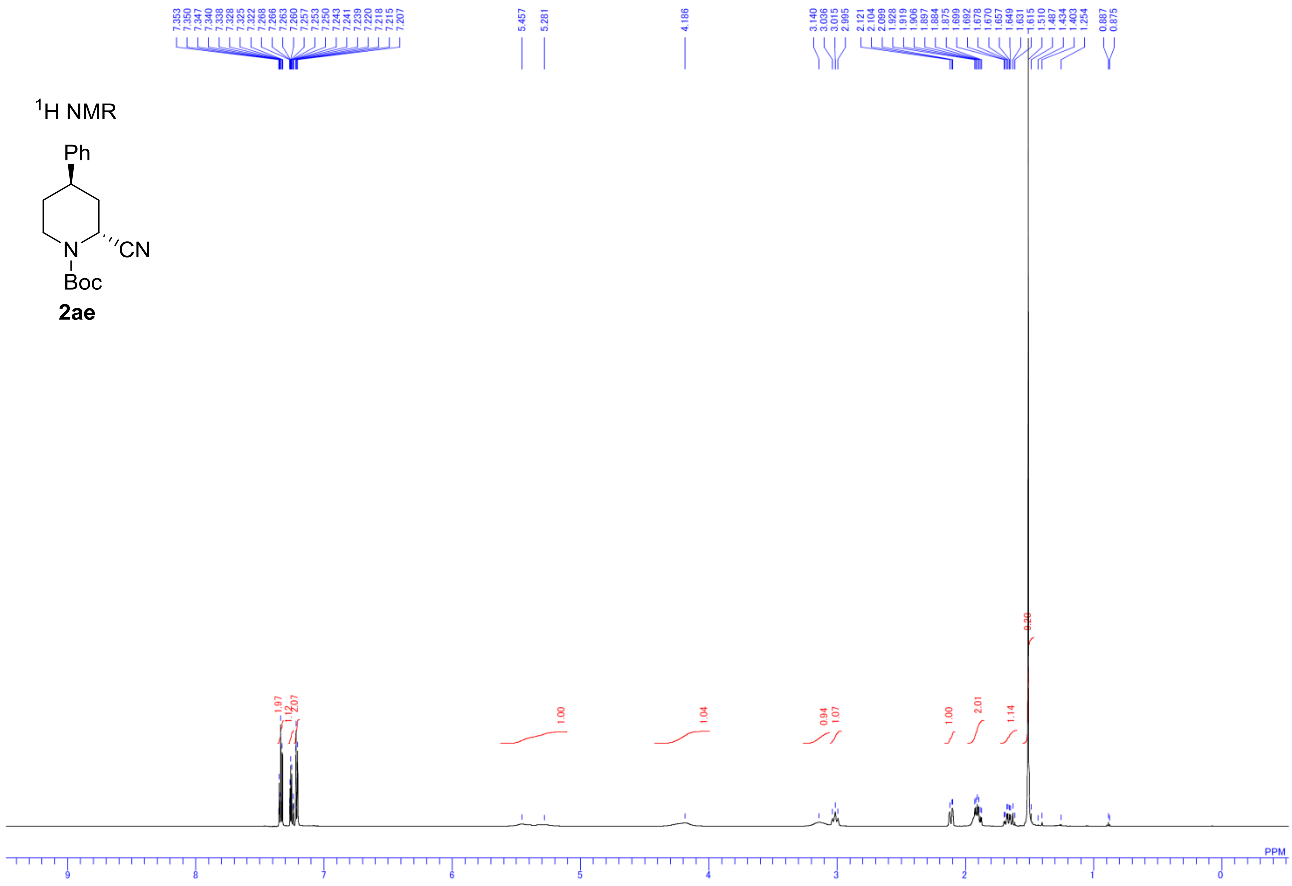
7.353 7.350 7.347 7.340 7.338 7.328 7.325 7.322 7.268 7.266 7.263 7.260 7.257 7.253 7.250 7.243 7.241 7.239 7.220 7.218 7.215 7.207
5.457 5.281
4.186
3.140 3.036 3.015 2.995 2.121 2.104 2.099 1.928 1.919 1.906 1.897 1.884 1.875 1.699 1.692 1.678 1.670 1.657 1.649 1.631 1.615 1.510 1.487 1.434 1.403 1.254 0.887 0.875
^{1}H NMR
Ph
N
CN
Boc
2ae
1.97 1.12 2.07 1.00 1.04 0.94 1.07 1.00 2.01 1.14 9.20
PPM

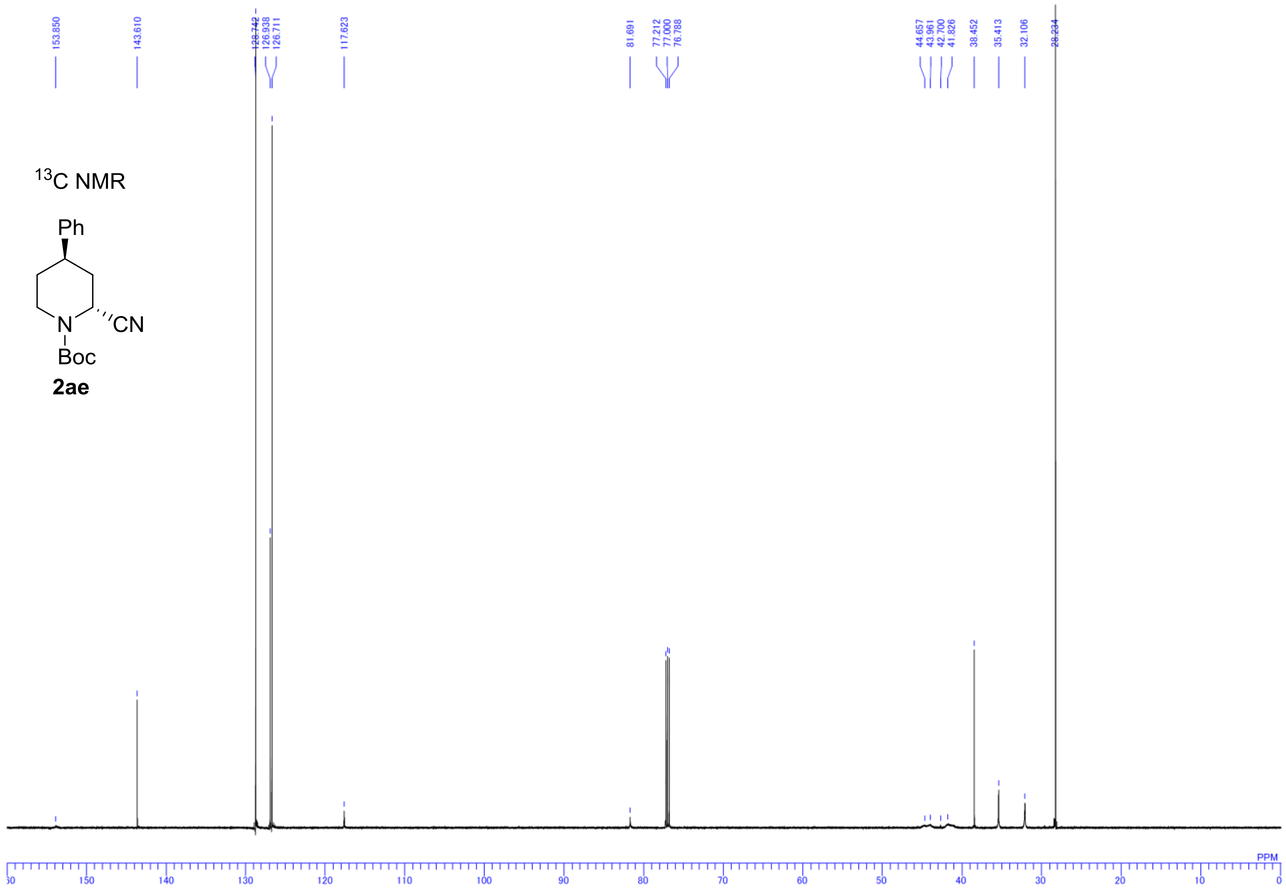
153.850
143.610
128.742 126.938 126.711
117.623
81.691
77.212 77.000 76.788
44.657 43.961 42.700 41.826
38.452
35.413
32.106
28.234
^{13}C NMR
Ph
N
CN
Boc
2ae
PPM

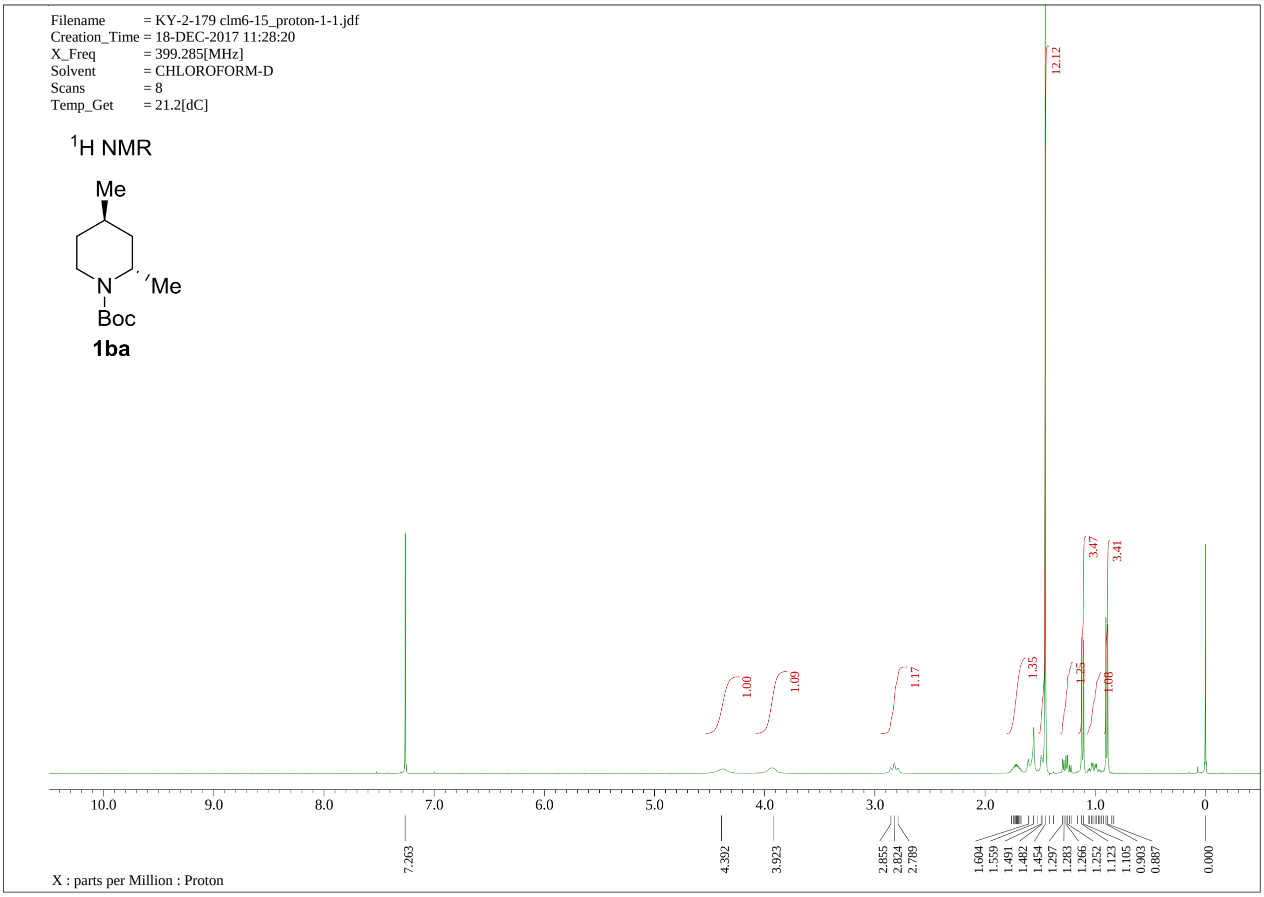
Filename = KY-2-179 clm6-15_proton-1-1.jdf
Creation_Time = 18-DEC-2017 11:28:20
X_Freq = 399.285[MHz]
Solvent = CHLOROFORM-D
Scans = 8
Temp_Get = 21.2[dC]
1H NMR
Me
N
Me
Boc
1ba
12.12
3.47
3.41
1.00
1.09
1.17
1.35
1.25
1.08
10.0
9.0
8.0
7.0
6.0
5.0
4.0
3.0
2.0
1.0
0
7.263
4.392
3.923
2.855
2.824
2.789
1.604
1.559
1.491
1.482
1.454
1.297
1.283
1.266
1.252
1.123
1.105
0.903
0.887
0.000
X : parts per Million : Proton

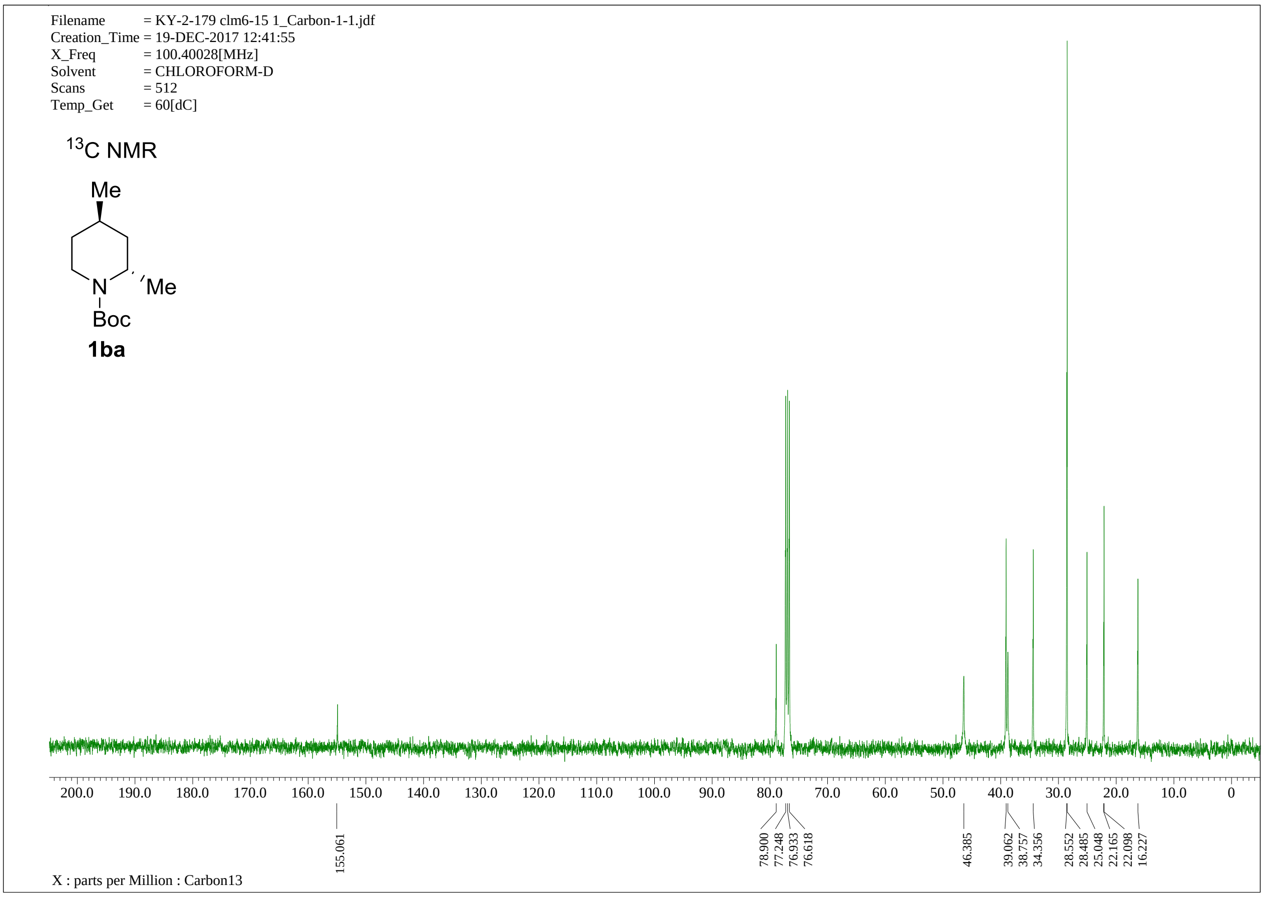
Filename = KY-2-179 clm6-15 1_Carbon-1-1.jdf
Creation_Time = 19-DEC-2017 12:41:55
X_Freq = 100.40028[MHz]
Solvent = CHLOROFORM-D
Scans = 512
Temp_Get = 60[dC]
13C NMR
Me
N
Me
Boc
1ba
200.0
190.0
180.0
170.0
160.0
150.0
140.0
130.0
120.0
110.0
100.0
90.0
80.0
70.0
60.0
50.0
40.0
30.0
20.0
10.0
0
155.061
78.900
77.248
76.933
76.618
46.385
39.062
38.757
34.356
28.552
28.485
25.048
22.165
22.098
16.227
X : parts per Million : Carbon13

^{1}H NMR

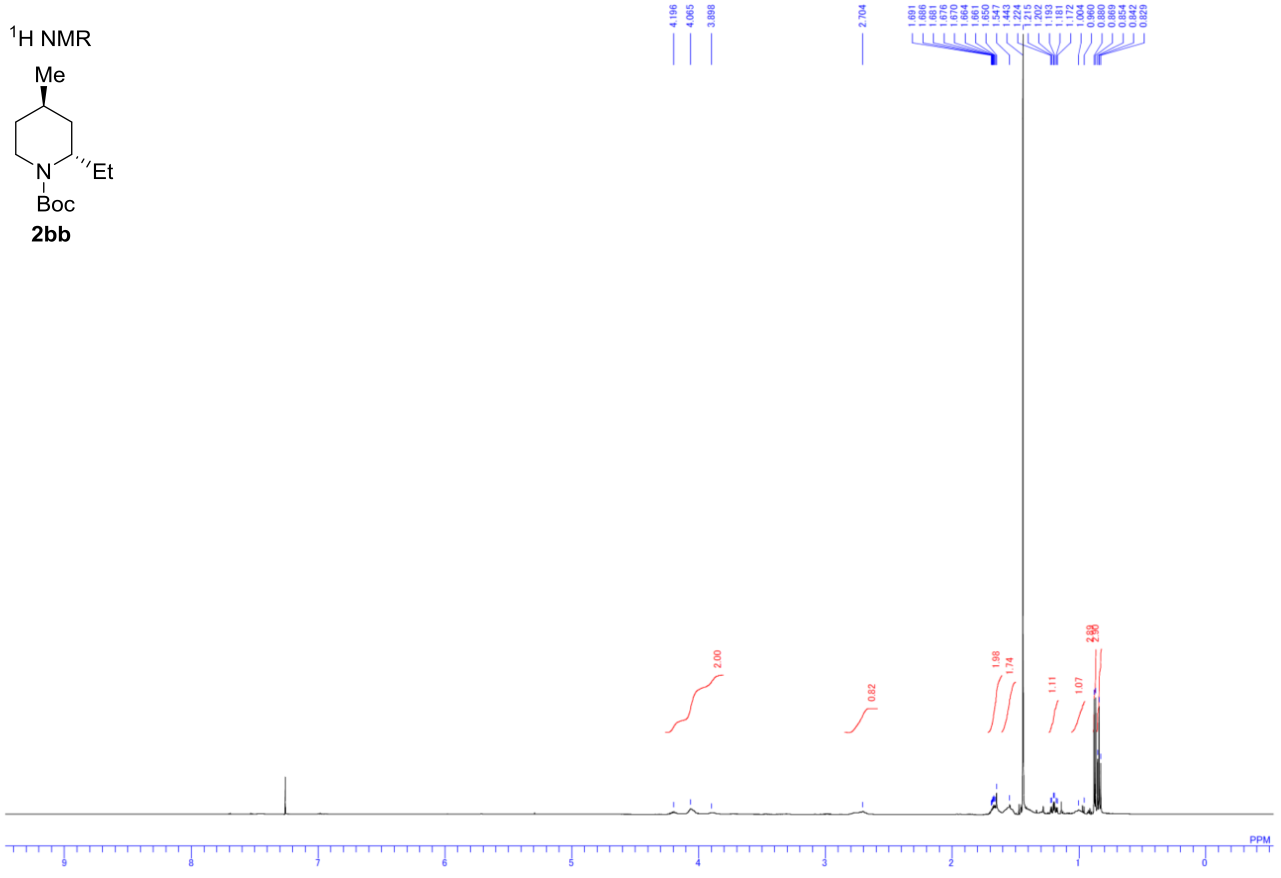

2bb

^{13}C NMR

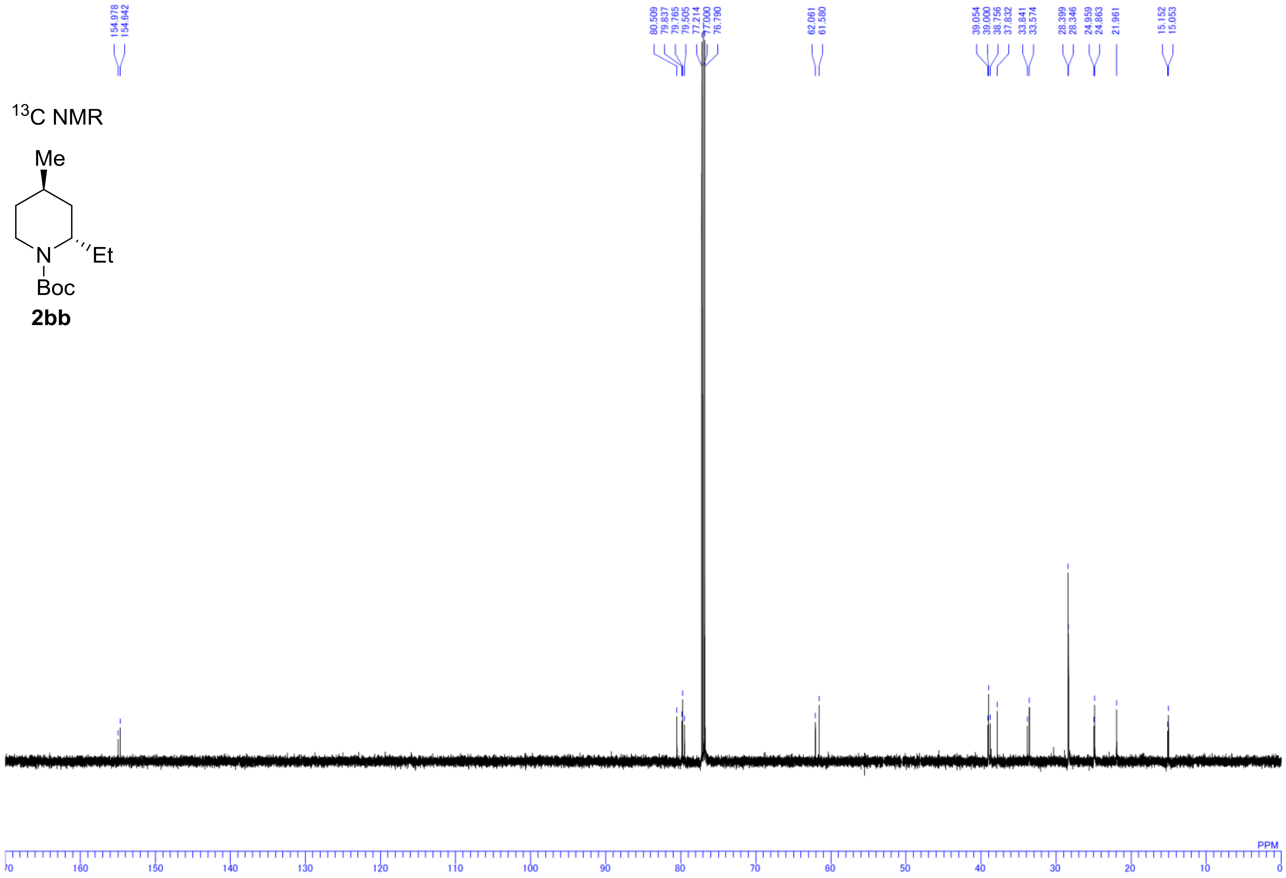

2bb

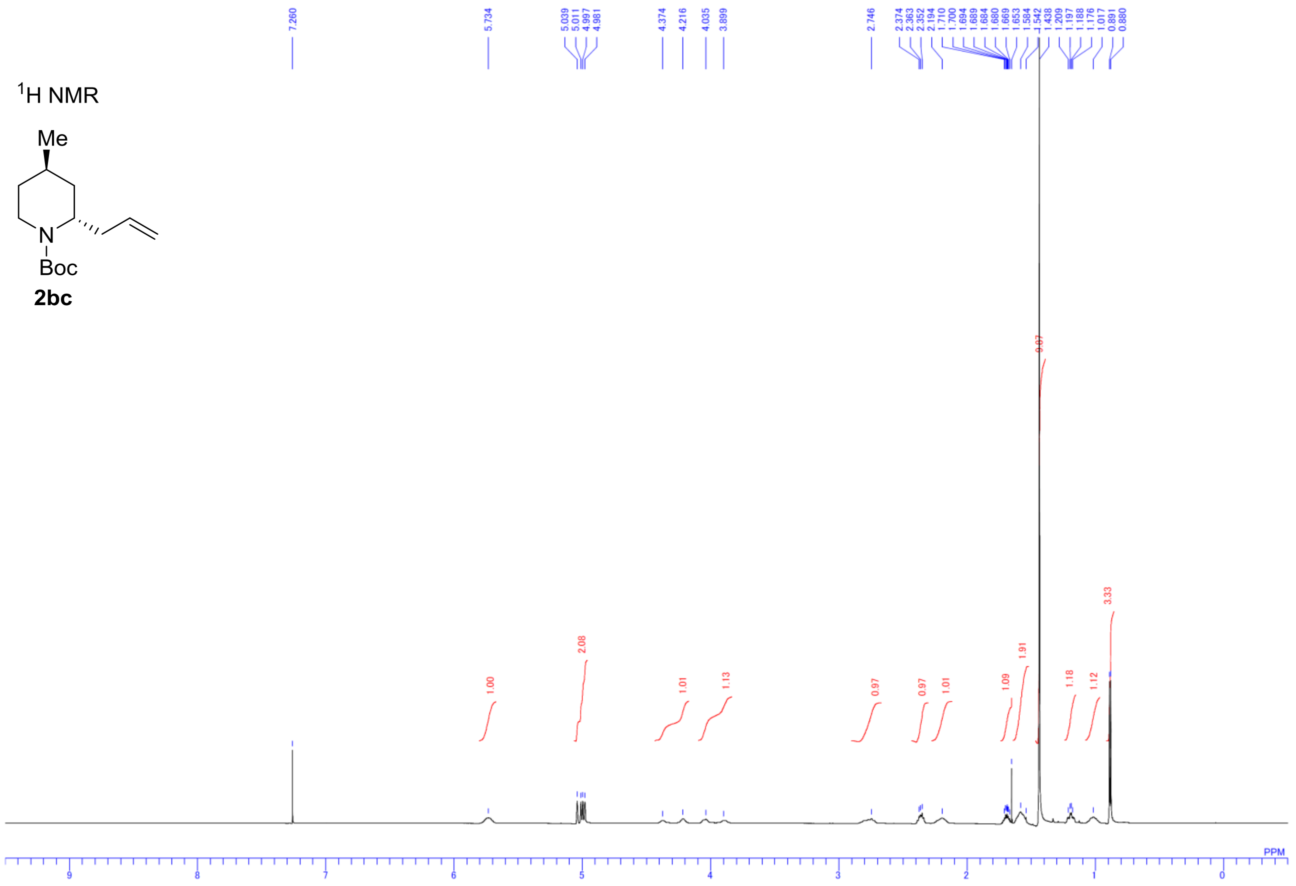
1H NMR
Me
N
Boc
2bc

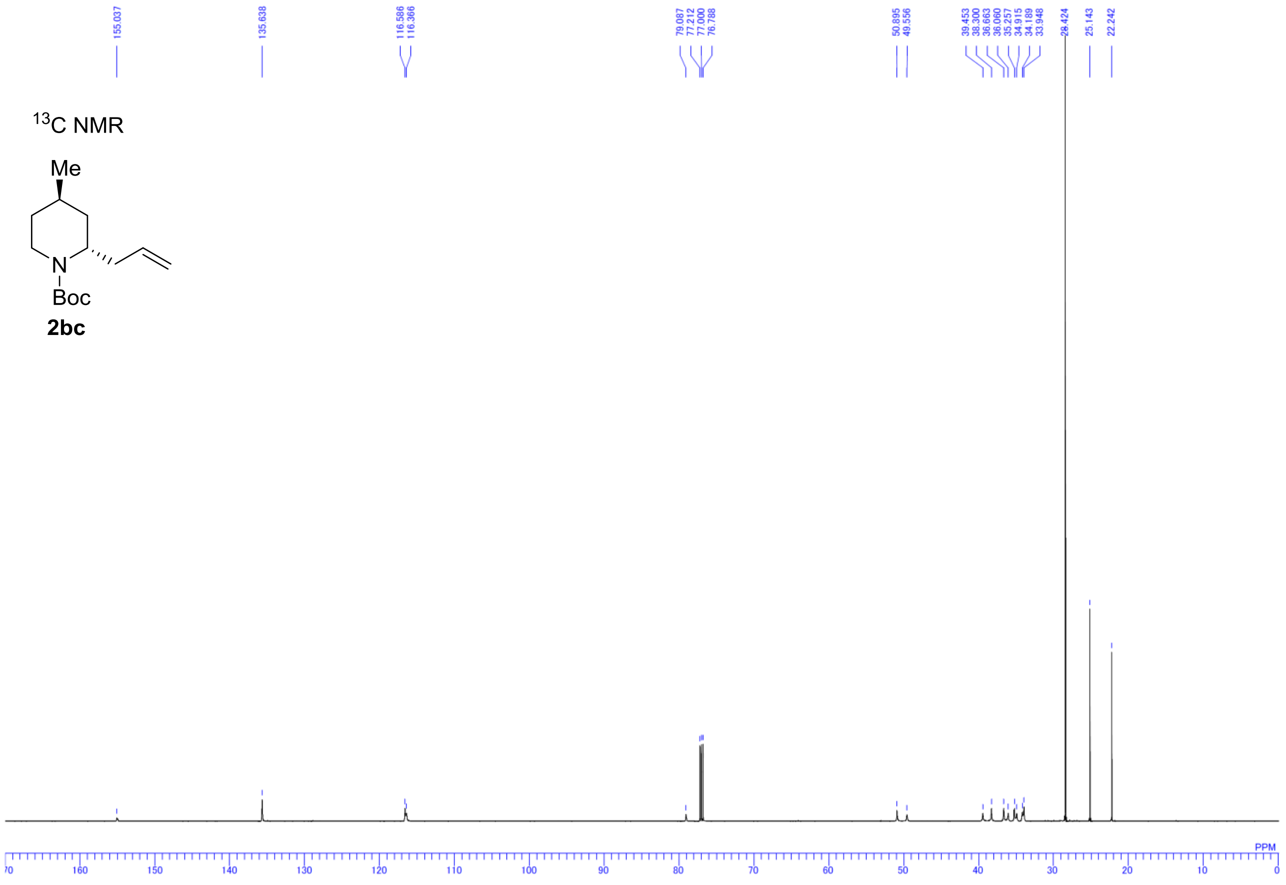
13C NMR
Me
N
Boc
2bc

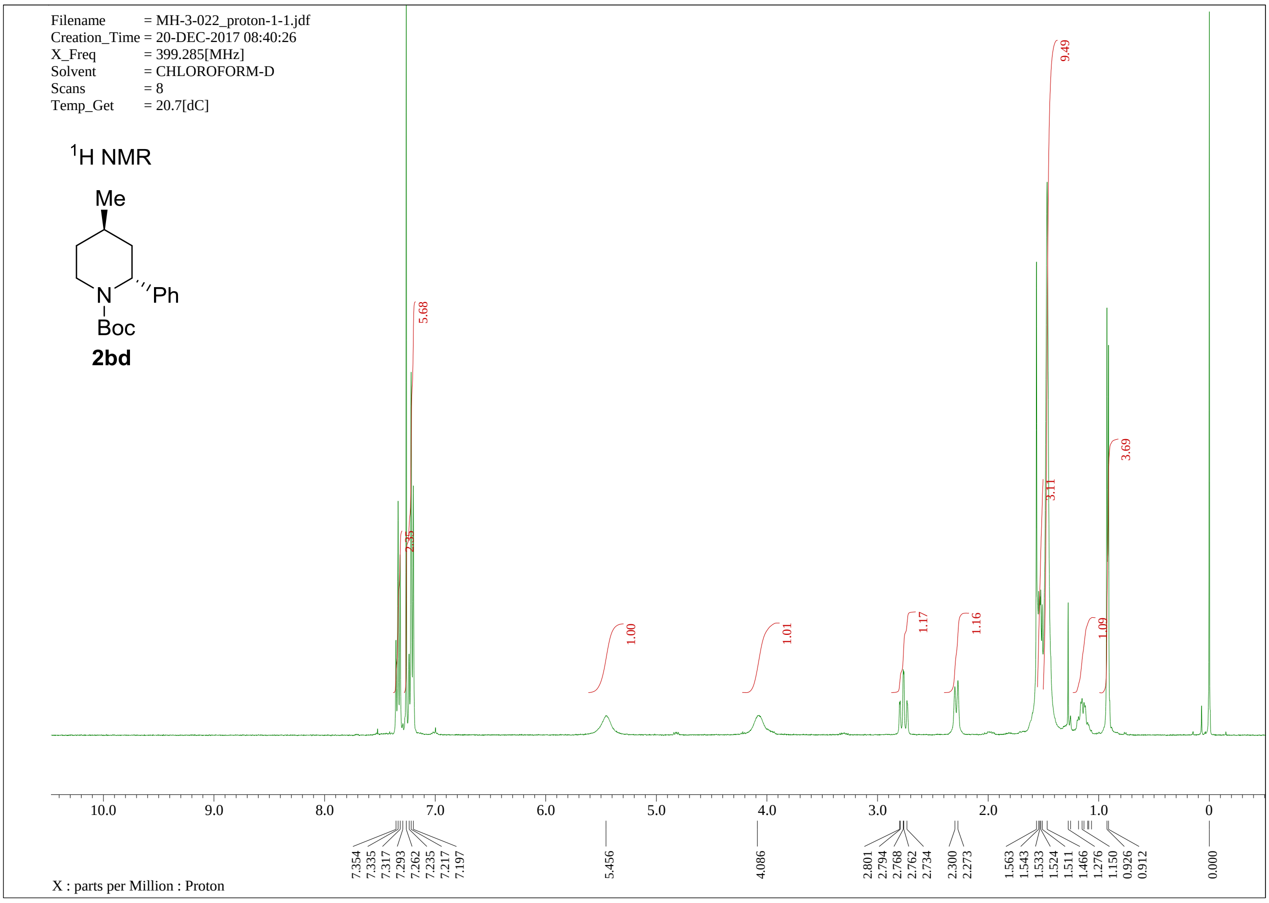
Filename = MH-3-022_proton-1-1.jdf
Creation_Time = 20-DEC-2017 08:40:26
X_Freq = 399.285[MHz]
Solvent = CHLOROFORM-D
Scans = 8
Temp_Get = 20.7[dC]
1H NMR
Me
N
Ph
Boc
2bd
9.49
5.68
3.69
3.11
2.35
1.00
1.01
1.17
1.16
1.09
10.0
9.0
8.0
7.0
6.0
5.0
4.0
3.0
2.0
1.0
0
7.354
7.335
7.317
7.293
7.262
7.235
7.217
7.197
5.456
4.086
2.801
2.794
2.768
2.762
2.734
2.300
2.273
1.563
1.543
1.533
1.524
1.511
1.466
1.276
1.150
0.926
0.912
0.000
X : parts per Million : Proton

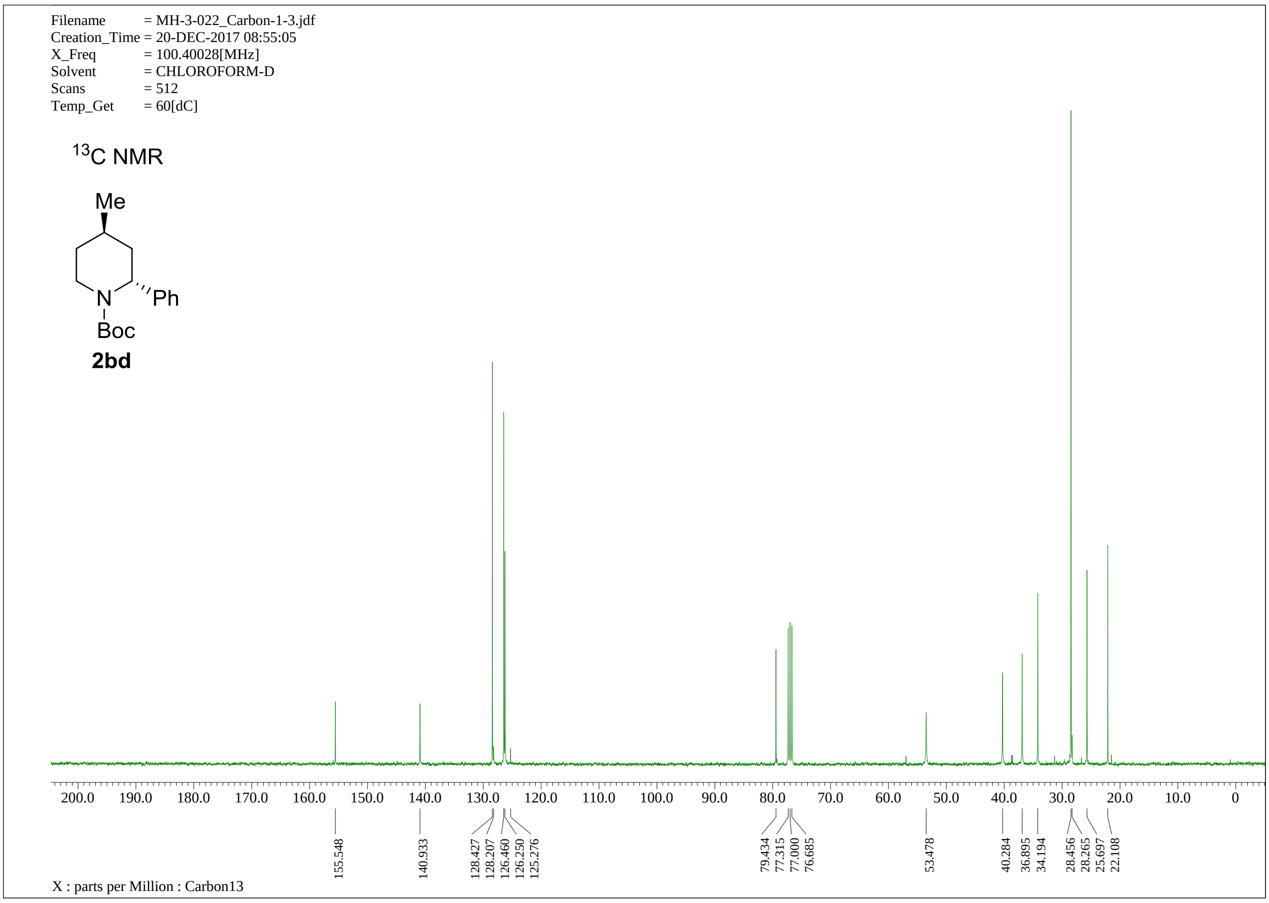
Filename = MH-3-022_Carbon-1-3.jdf
Creation_Time = 20-DEC-2017 08:55:05
X_Freq = 100.40028[MHz]
Solvent = CHLOROFORM-D
Scans = 512
Temp_Get = 60[dC]
13C NMR
Me
N
Ph
Boc
2bd
200.0
190.0
180.0
170.0
160.0
150.0
140.0
130.0
120.0
110.0
100.0
90.0
80.0
70.0
60.0
50.0
40.0
30.0
20.0
10.0
0
155.548
140.933
128.427
128.207
126.460
126.250
125.276
79.434
77.315
77.000
76.685
53.478
40.284
36.895
34.194
28.456
28.265
25.697
22.108
X : parts per Million : Carbon13

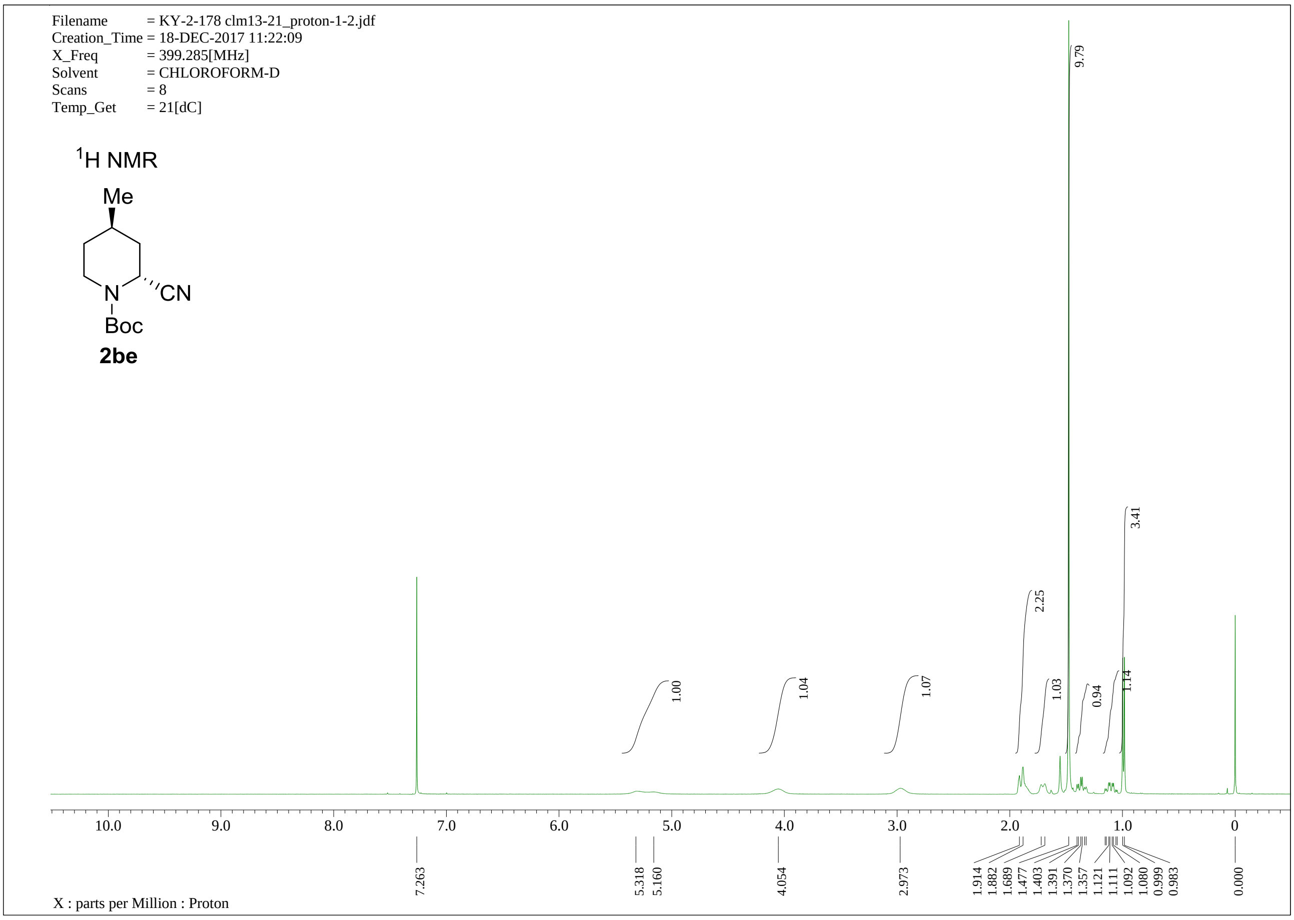
Filename = KY-2-178 clm13-21_proton-1-2.jdf
Creation_Time = 18-DEC-2017 11:22:09
X_Freq = 399.285[MHz]
Solvent = CHLOROFORM-D
Scans = 8
Temp_Get = 21[dC]
1H NMR
Me
N
CN
Boc
2be
9.79
3.41
2.25
1.00
1.04
1.07
1.03
0.94
1.14
10.0
9.0
8.0
7.0
6.0
5.0
4.0
3.0
2.0
1.0
0
7.263
5.318
5.160
4.054
2.973
1.914
1.882
1.689
1.477
1.403
1.391
1.370
1.357
1.121
1.111
1.092
1.080
0.999
0.983
0.000
X : parts per Million : Proton

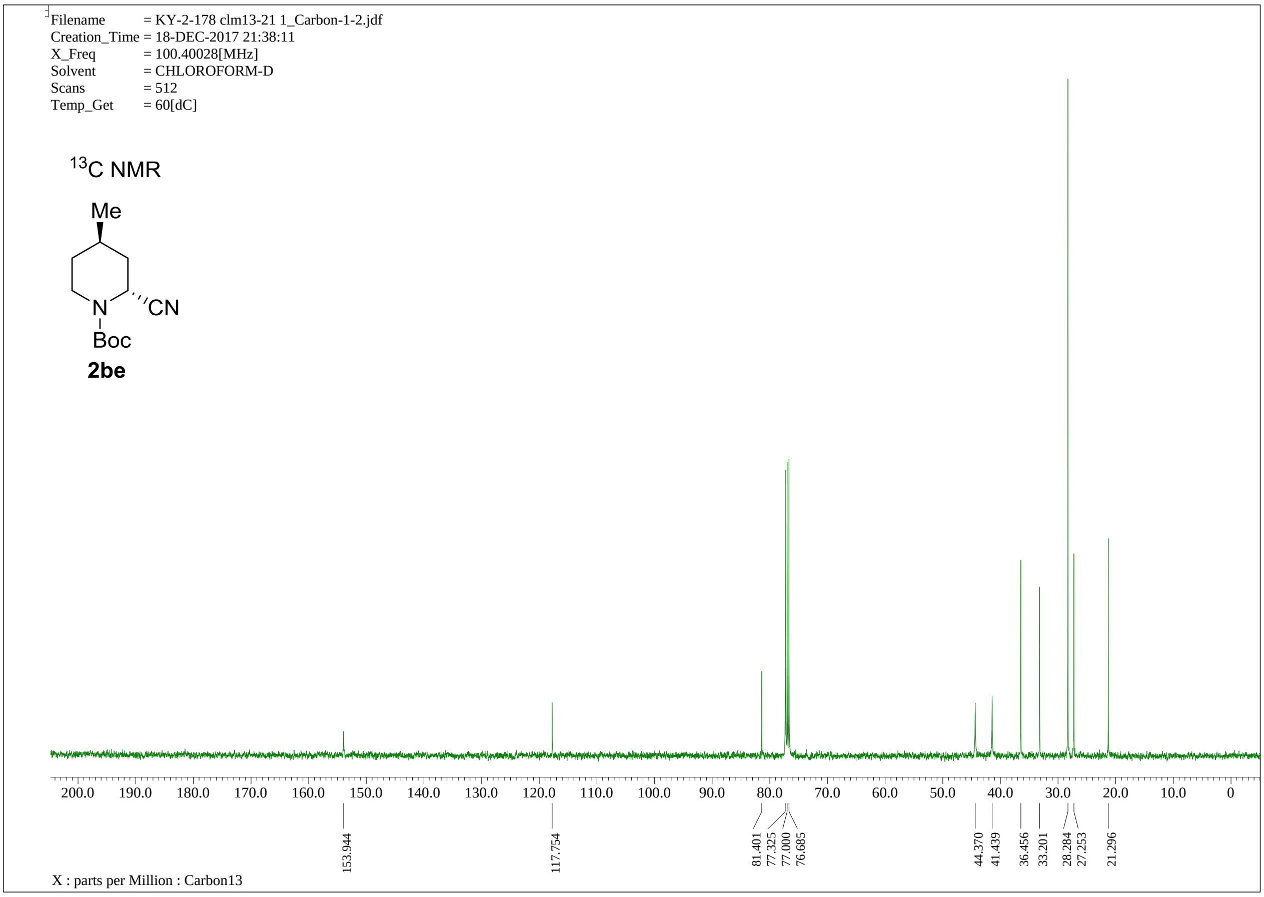
Filename = KY-2-178 clm13-21 1_Carbon-1-2.jdf
Creation_Time = 18-DEC-2017 21:38:11
X_Freq = 100.40028[MHz]
Solvent = CHLOROFORM-D
Scans = 512
Temp_Get = 60[dC]
13C NMR
Me
N
CN
Boc
2be
200.0
190.0
180.0
170.0
160.0
150.0
140.0
130.0
120.0
110.0
100.0
90.0
80.0
70.0
60.0
50.0
40.0
30.0
20.0
10.0
0
153.944
117.754
81.401
77.325
77.000
76.685
44.370
41.439
36.456
33.201
28.284
27.253
21.296
X : parts per Million : Carbon13

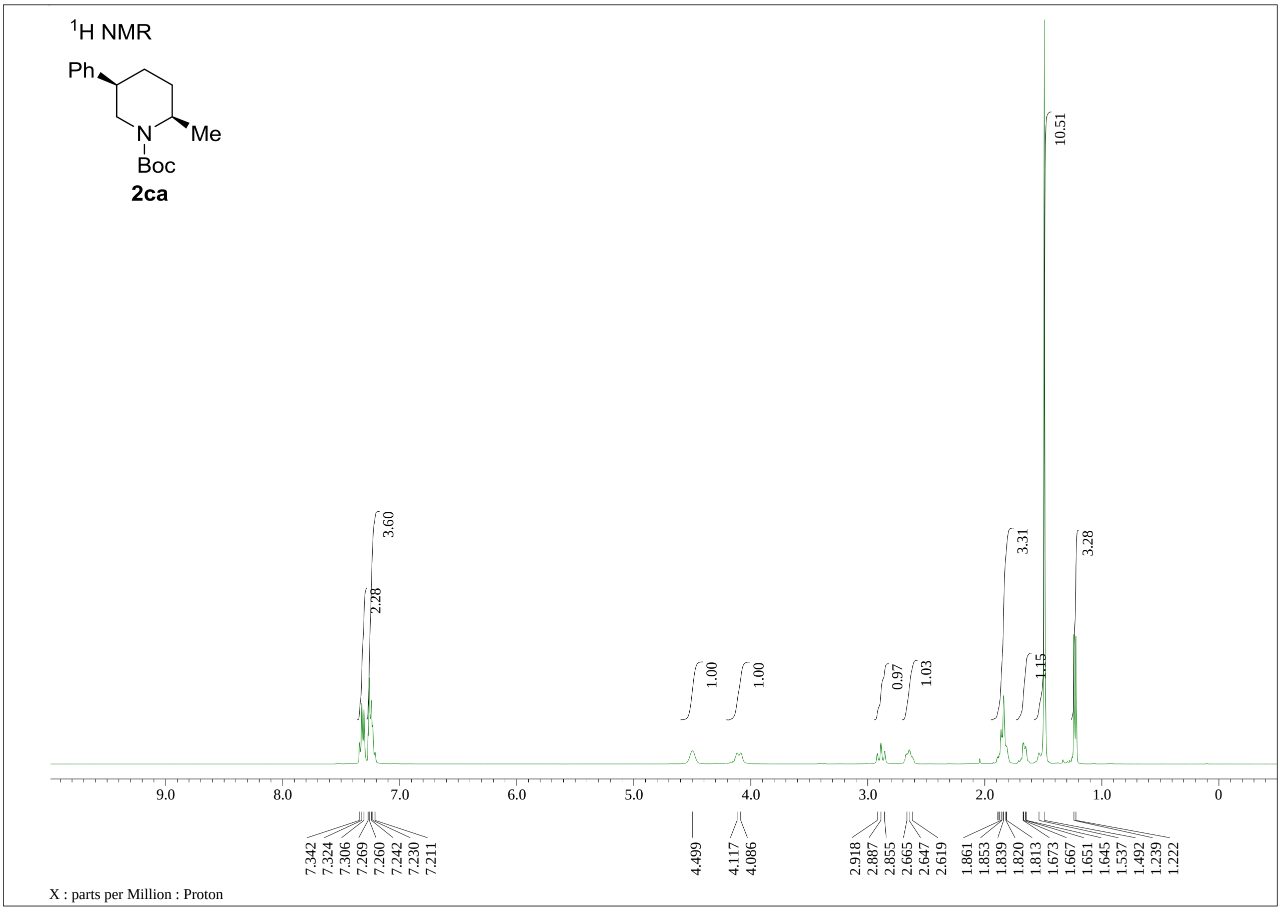

^{1}H NMR
Ph
N
Me
Boc
2ca
10.51
3.60
2.28
1.00
1.00
0.97
1.03
3.31
1.15
3.28
9.0
8.0
7.0
6.0
5.0
4.0
3.0
2.0
1.0
0
7.342
7.324
7.306
7.269
7.260
7.242
7.230
7.211
4.499
4.117
4.086
2.918
2.887
2.855
2.665
2.647
2.619
1.861
1.853
1.839
1.820
1.813
1.673
1.667
1.651
1.645
1.537
1.492
1.239
1.222
X : parts per Million : Proton

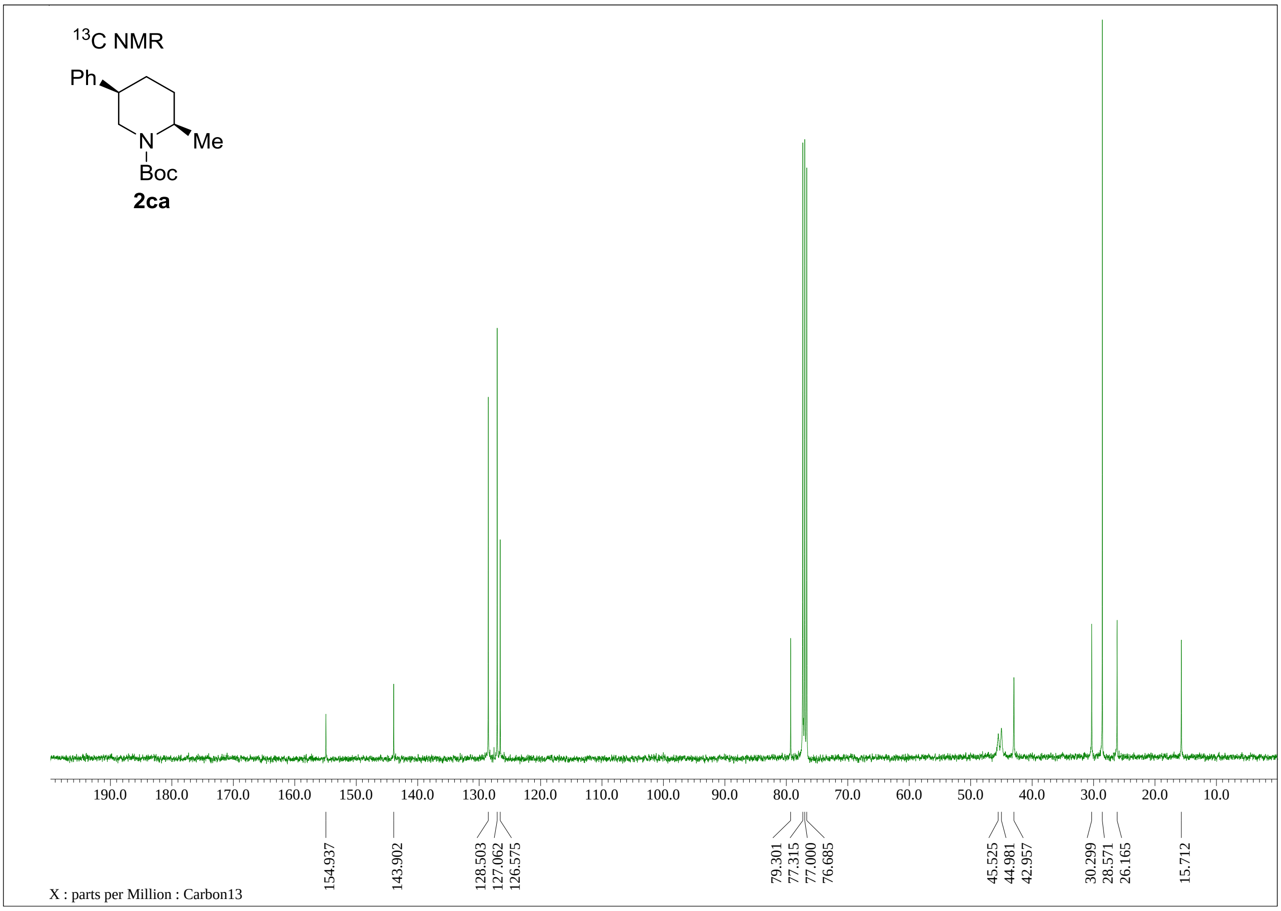

^{13}C NMR
Ph
N
Me
Boc
2ca
190.0
180.0
170.0
160.0
150.0
140.0
130.0
120.0
110.0
100.0
90.0
80.0
70.0
60.0
50.0
40.0
30.0
20.0
10.0
154.937
143.902
128.503
127.062
126.575
79.301
77.315
77.000
76.685
45.525
44.981
42.957
30.299
28.571
26.165
15.712
X : parts per Million : Carbon13

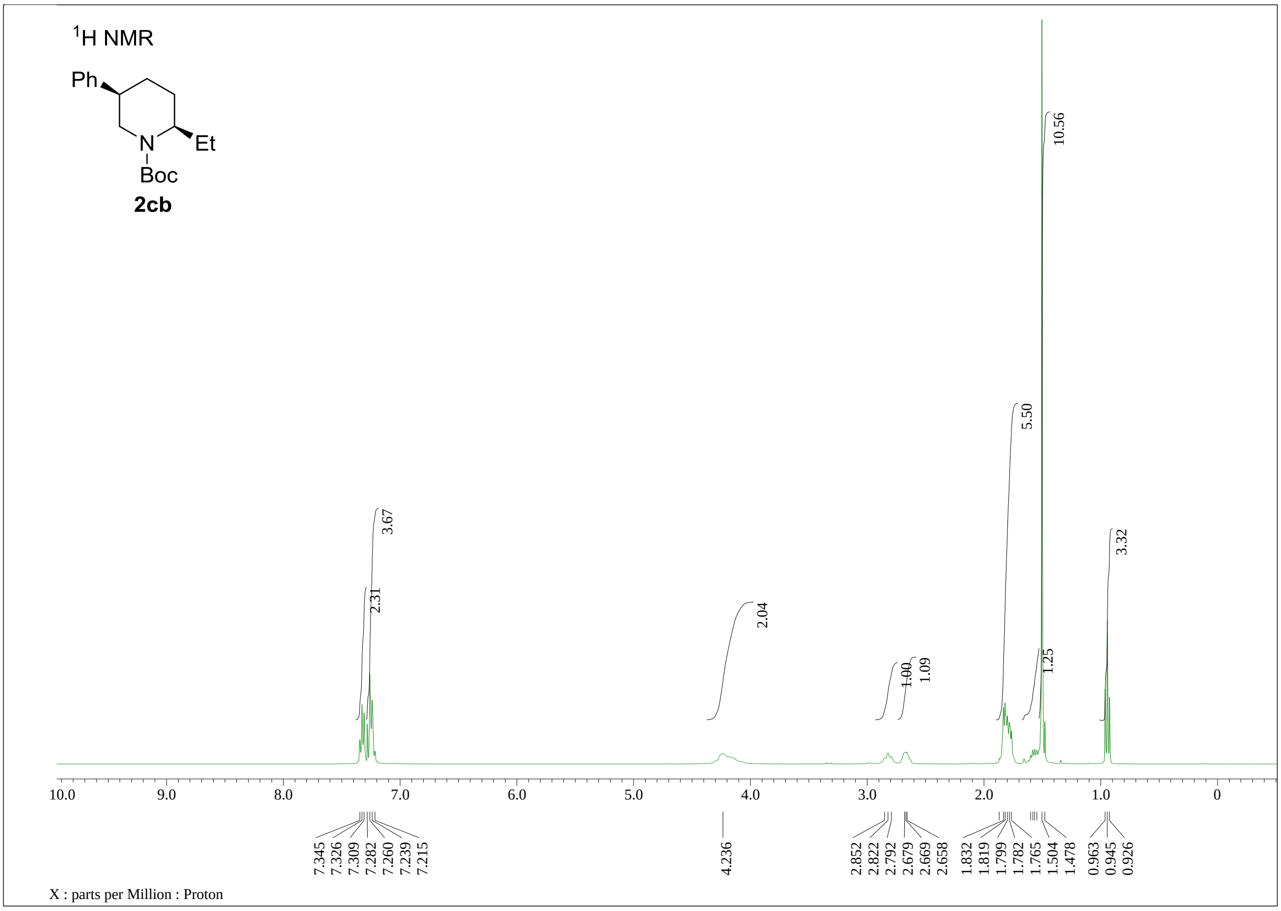

1H NMR
Ph
N
Et
Boc
2cb
10.56
5.50
3.67
2.31
2.04
1.00
1.09
1.25
3.32
10.0
9.0
8.0
7.0
6.0
5.0
4.0
3.0
2.0
1.0
0
7.345
7.326
7.309
7.282
7.260
7.239
7.215
4.236
2.852
2.822
2.792
2.679
2.669
2.658
1.832
1.819
1.799
1.782
1.765
1.504
1.478
0.963
0.945
0.926
X : parts per Million : Proton

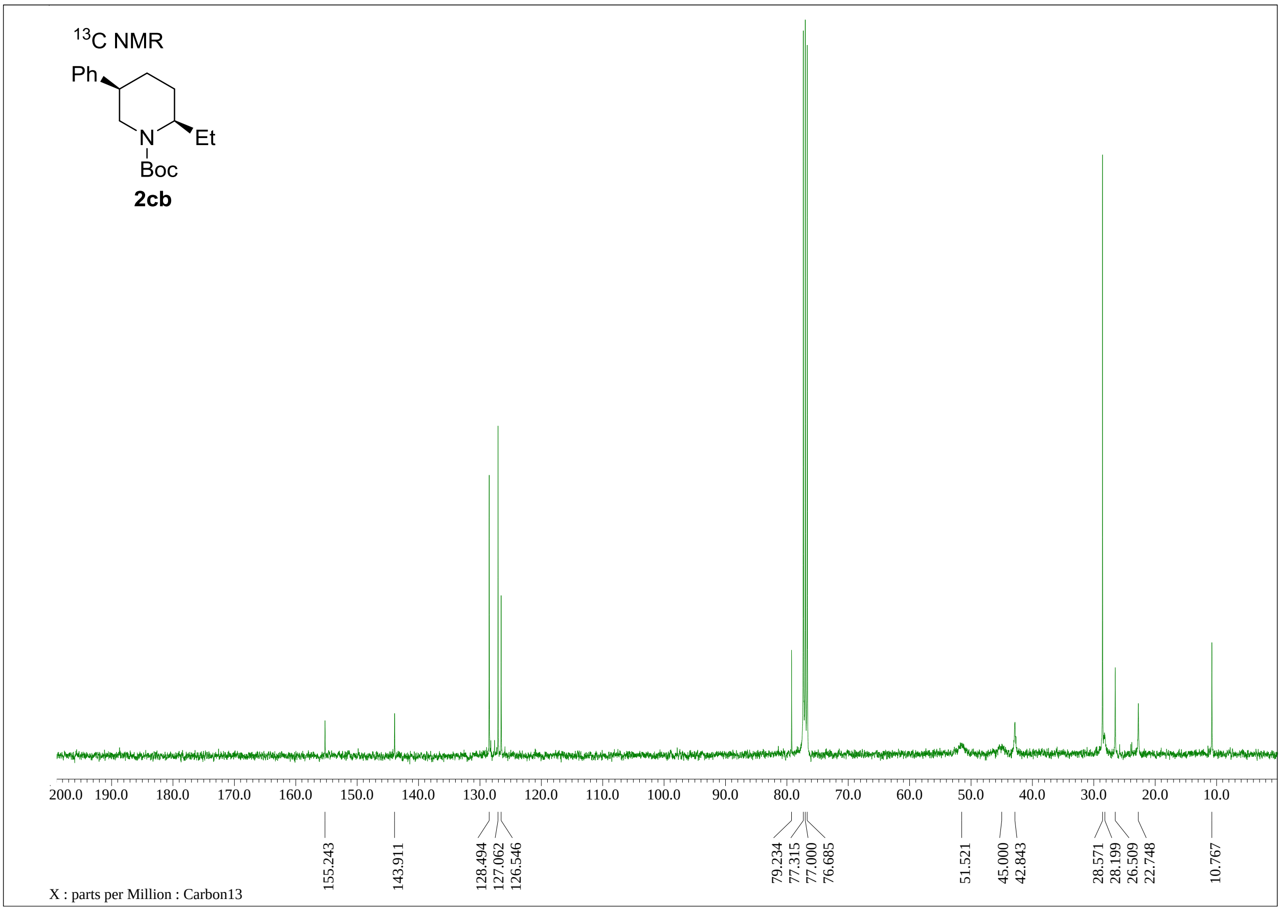

13C NMR
Ph
N
Et
Boc
2cb
200.0
190.0
180.0
170.0
160.0
150.0
140.0
130.0
120.0
110.0
100.0
90.0
80.0
70.0
60.0
50.0
40.0
30.0
20.0
10.0
155.243
143.911
128.494
127.062
126.546
79.234
77.315
77.000
76.685
51.521
45.000
42.843
28.571
28.199
26.509
22.748
10.767
X : parts per Million : Carbon13

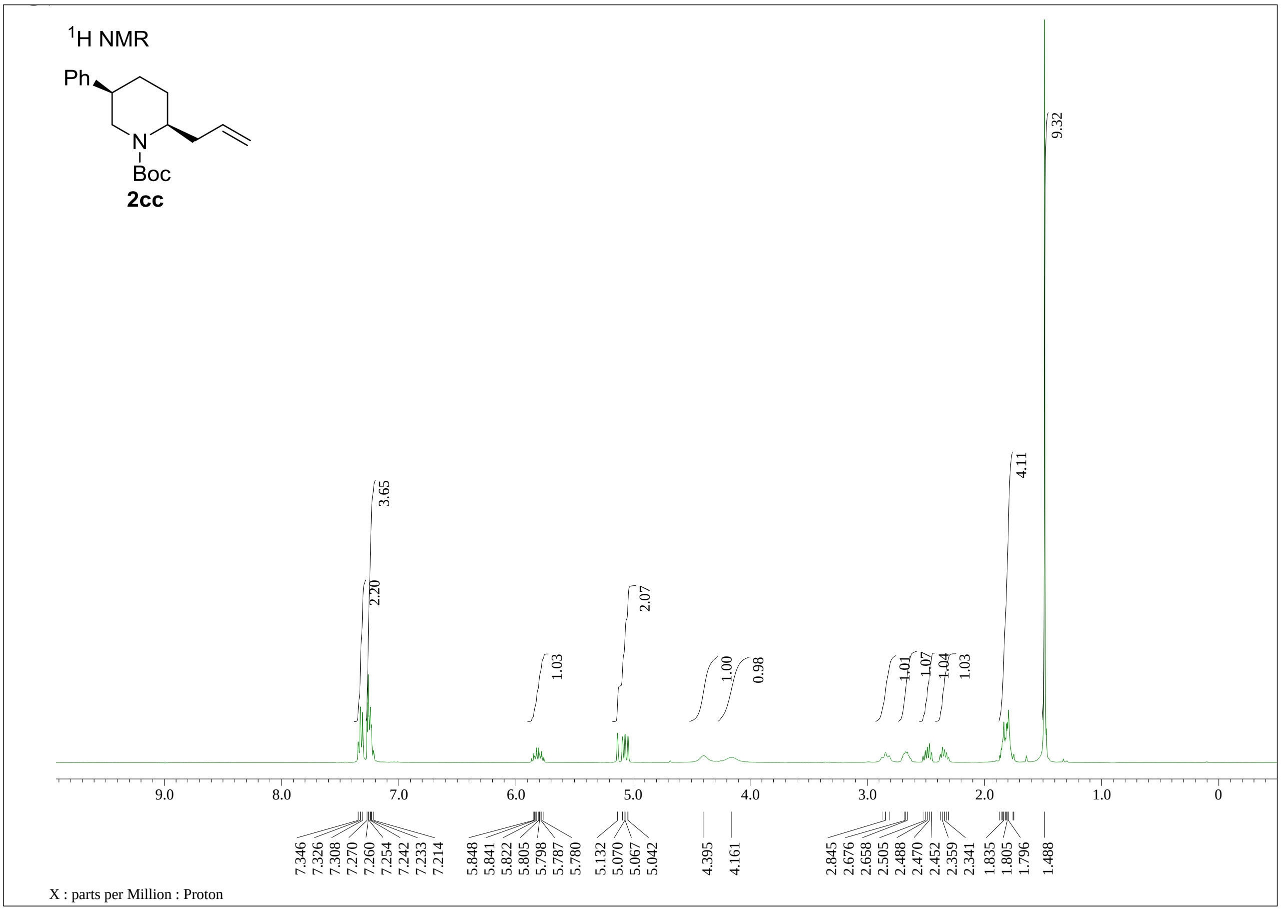
1H NMR
Ph
N
Boc
2cc
3.65
2.20
1.03
2.07
1.00
0.98
1.01
1.07
1.04
1.03
4.11
9.32
9.0
8.0
7.0
6.0
5.0
4.0
3.0
2.0
1.0
0
7.346
7.326
7.308
7.270
7.260
7.254
7.242
7.233
7.214
5.848
5.841
5.822
5.805
5.798
5.787
5.780
5.132
5.070
5.067
5.042
4.395
4.161
2.845
2.676
2.658
2.505
2.488
2.470
2.452
2.359
2.341
1.835
1.805
1.796
1.488
X : parts per Million : Proton

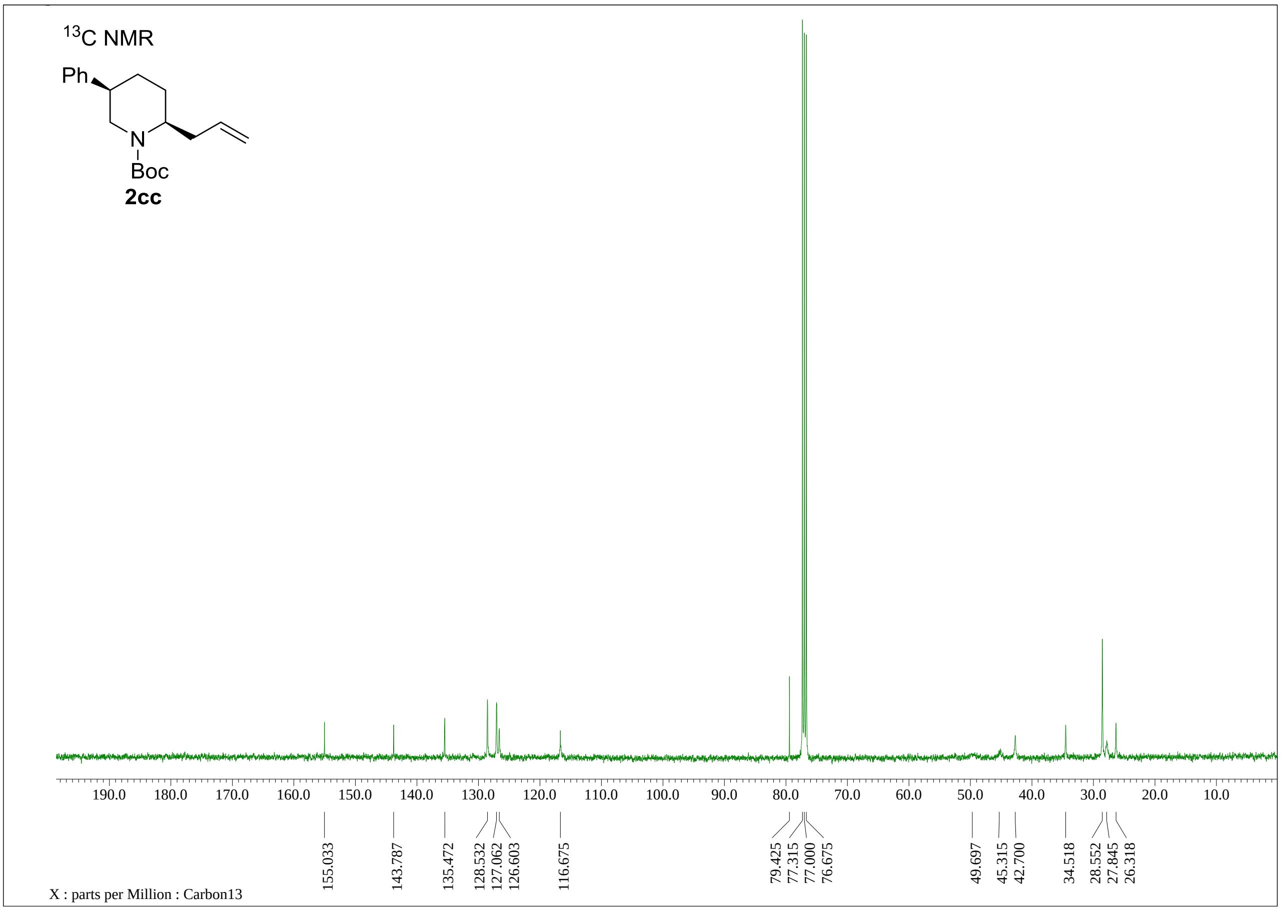
13C NMR
Ph
N
Boc
2cc
190.0
180.0
170.0
160.0
150.0
140.0
130.0
120.0
110.0
100.0
90.0
80.0
70.0
60.0
50.0
40.0
30.0
20.0
10.0
155.033
143.787
135.472
128.532
127.062
126.603
116.675
79.425
77.315
77.000
76.675
49.697
45.315
42.700
34.518
28.552
27.845
26.318
X : parts per Million : Carbon13

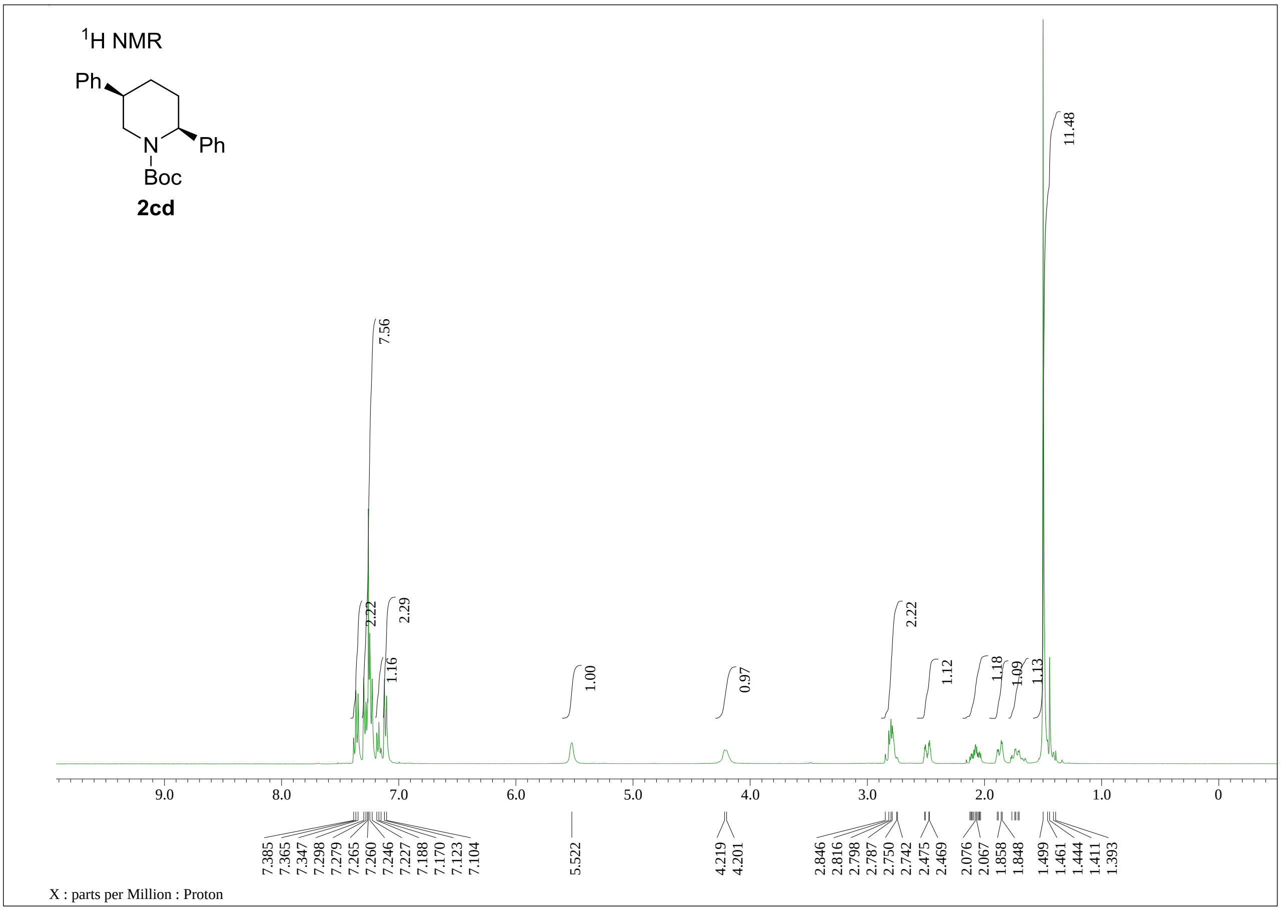

¹H NMR
Ph
N
Ph
Boc
2cd
7.56
2.22
2.29
1.16
1.00
0.97
2.22
1.12
1.18
1.09
1.13
11.48
9.0 8.0 7.0 6.0 5.0 4.0 3.0 2.0 1.0 0
7.385 7.365 7.347 7.298 7.279 7.265 7.260 7.246 7.227 7.188 7.170 7.123 7.104
5.522
4.219 4.201
2.846 2.816 2.798 2.787 2.750 2.742 2.475 2.469 2.076 2.067 1.858 1.848
1.499 1.461 1.444 1.411 1.393
X : parts per Million : Proton

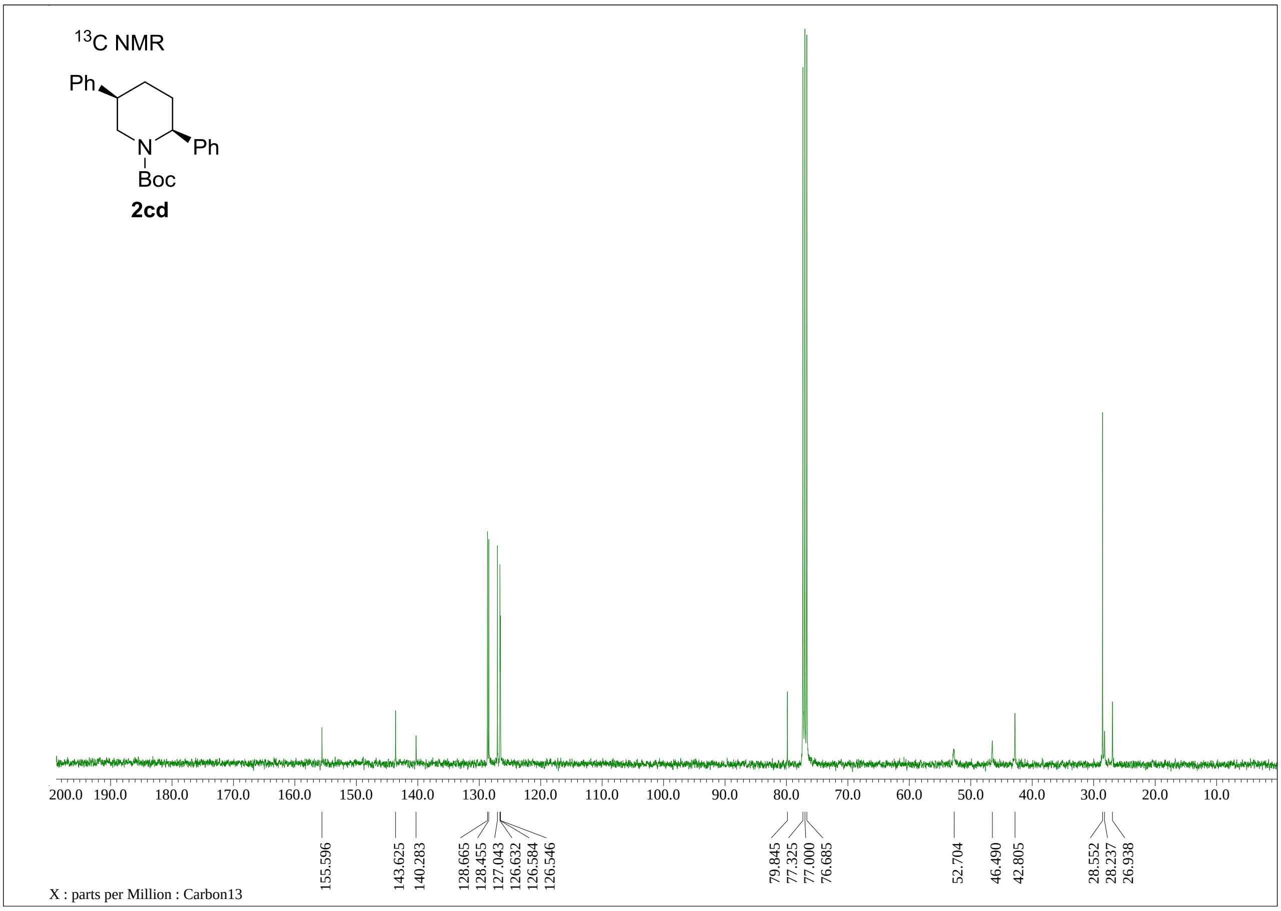

¹³C NMR
Ph
N
Ph
Boc
2cd
200.0 190.0 180.0 170.0 160.0 150.0 140.0 130.0 120.0 110.0 100.0 90.0 80.0 70.0 60.0 50.0 40.0 30.0 20.0 10.0
155.596
143.625 140.283
128.665 128.455 127.043 126.632 126.584 126.546
79.845 77.325 77.000 76.685
52.704
46.490 42.805
28.552 28.237 26.938
X : parts per Million : Carbon13

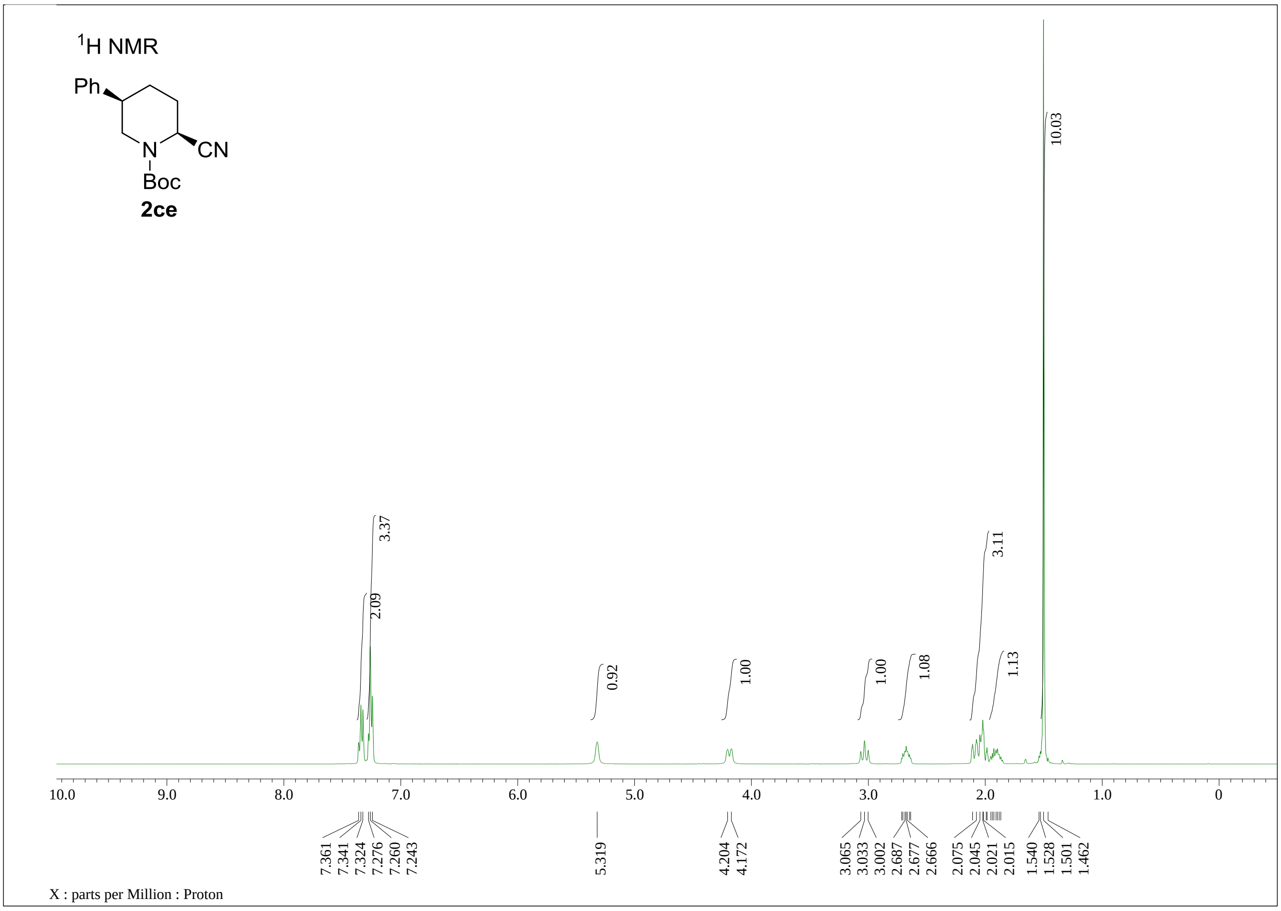

^{1}H NMR
Ph
N
CN
Boc
2ce
3.37
2.09
0.92
1.00
1.00
1.08
3.11
1.13
10.03
10.0 9.0 8.0 7.0 6.0 5.0 4.0 3.0 2.0 1.0 0
7.361 7.341 7.324 7.276 7.260 7.243
5.319
4.204 4.172
3.065 3.033 3.002 2.687 2.677 2.666
2.075 2.045 2.021 2.015
1.540 1.528 1.501 1.462
X : parts per Million : Proton

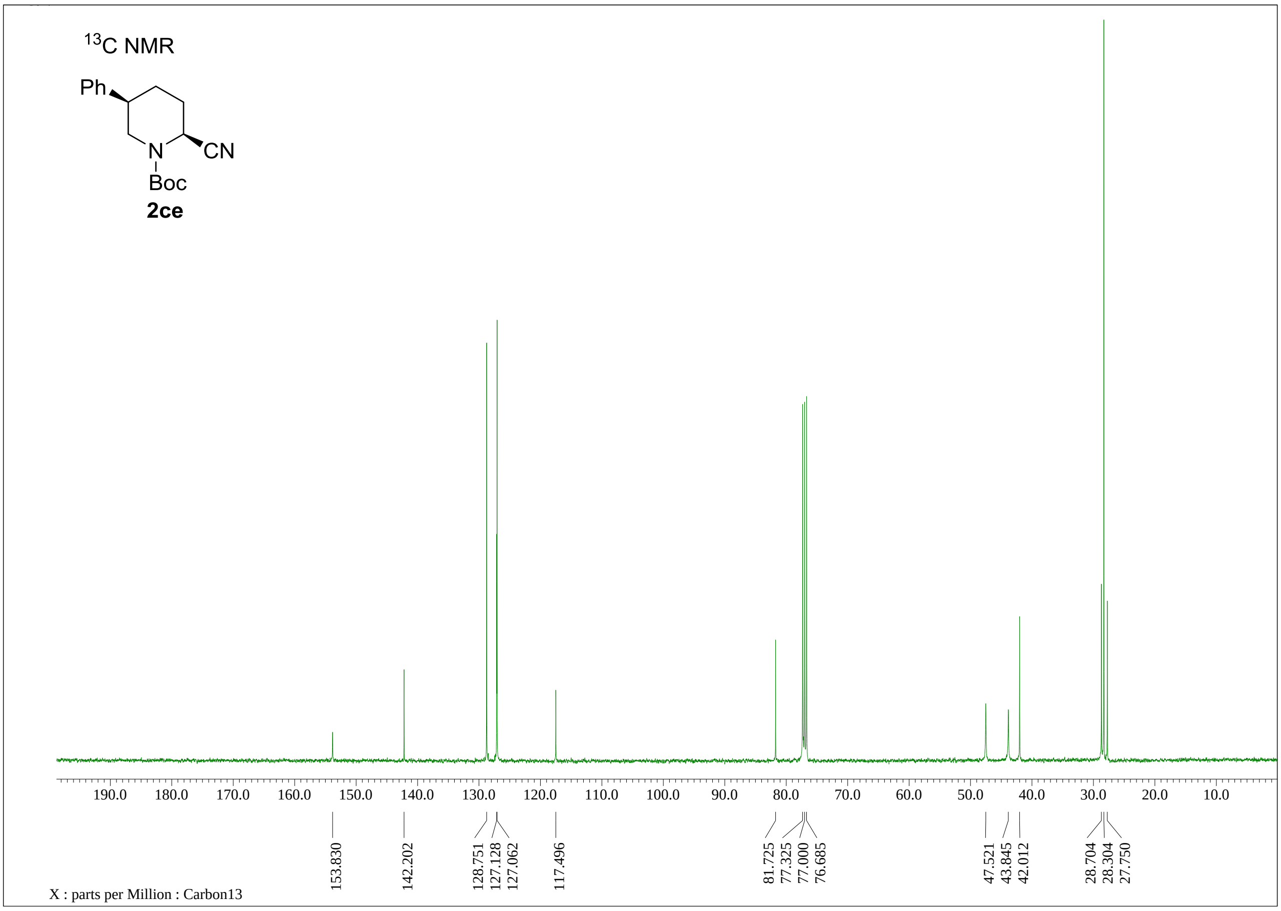

^{13}C NMR
Ph
N
CN
Boc
2ce
190.0 180.0 170.0 160.0 150.0 140.0 130.0 120.0 110.0 100.0 90.0 80.0 70.0 60.0 50.0 40.0 30.0 20.0 10.0
153.830
142.202
128.751 127.128 127.062
117.496
81.725 77.325 77.000 76.685
47.521 43.845 42.012
28.704 28.304 27.750
X : parts per Million : Carbon13

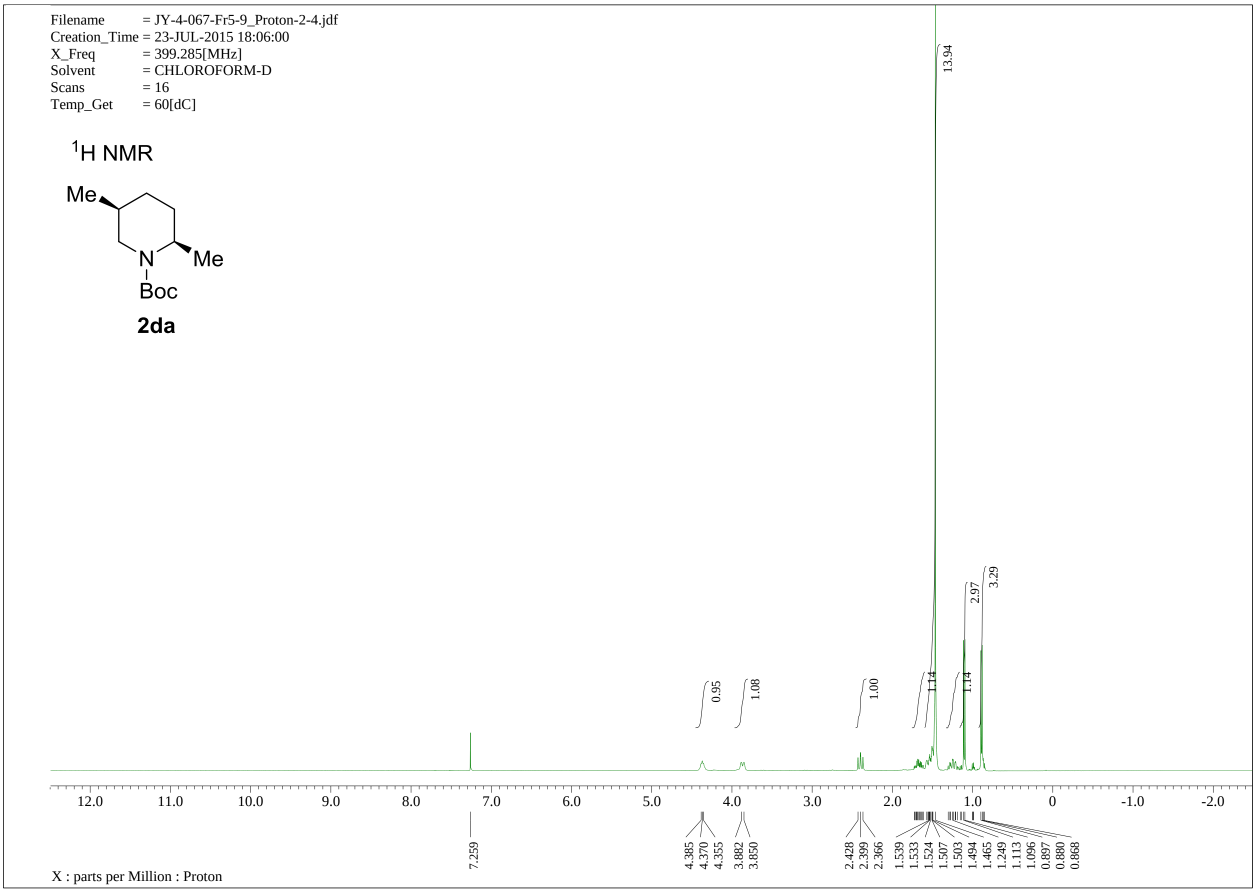
Filename = JY-4-067-Fr5-9_Proton-2-4.jdf
Creation_Time = 23-JUL-2015 18:06:00
X_Freq = 399.285[MHz]
Solvent = CHLOROFORM-D
Scans = 16
Temp_Get = 60[dC]
^{1}H NMR
Me
Me
N
Boc
2da
13.94
3.29
2.97
0.95
1.08
1.00
1.14
1.14
7.259
4.385 4.370 4.355 3.882 3.850
2.428 2.399 2.366 1.539 1.533 1.524 1.507 1.503 1.494 1.465 1.249 1.113 1.096 0.897 0.880 0.868
X : parts per Million : Proton

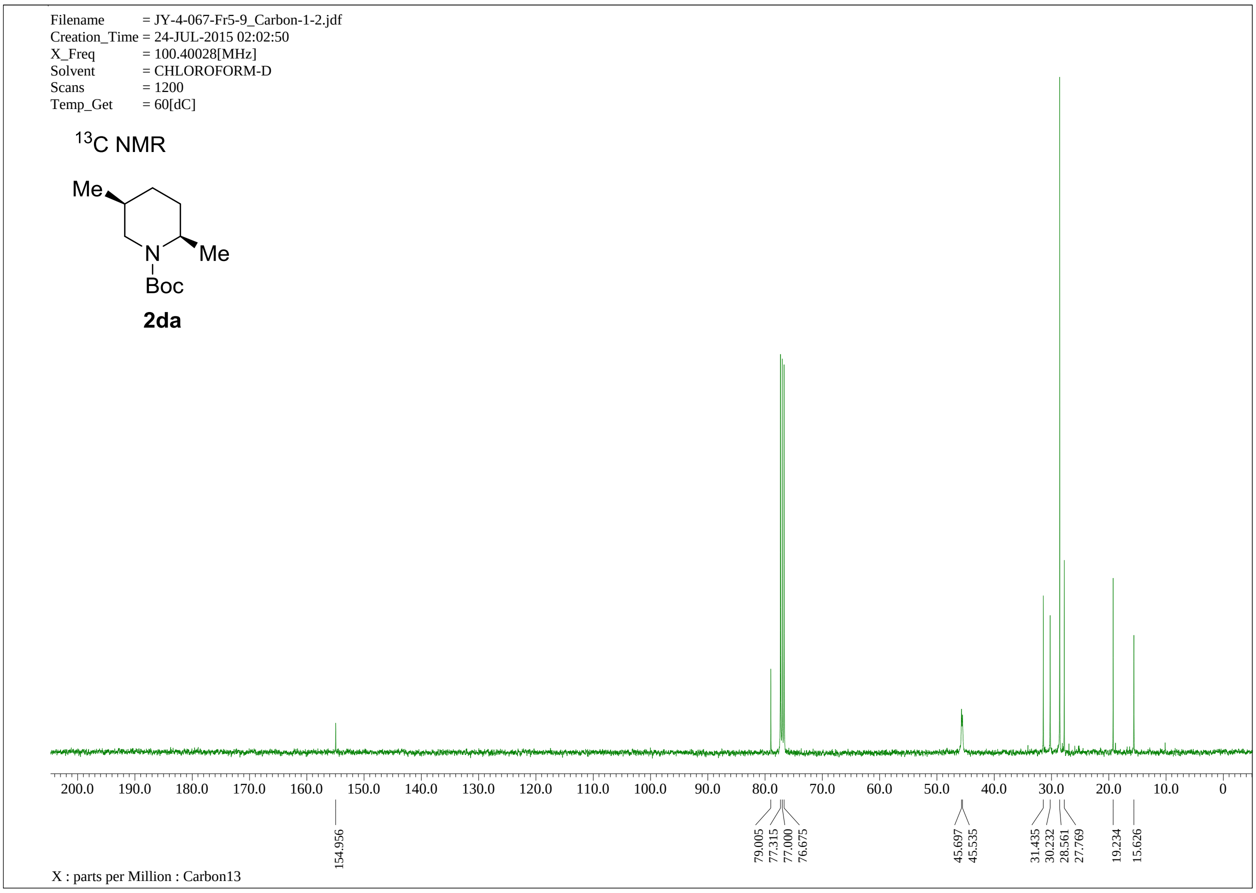
Filename = JY-4-067-Fr5-9_Carbon-1-2.jdf
Creation_Time = 24-JUL-2015 02:02:50
X_Freq = 100.40028[MHz]
Solvent = CHLOROFORM-D
Scans = 1200
Temp_Get = 60[dC]
^{13}C NMR
Me
Me
N
Boc
2da
154.956
79.005 77.315 77.000 76.675
45.697 45.535
31.435 30.232 28.561 27.769
19.234 15.626
X : parts per Million : Carbon13

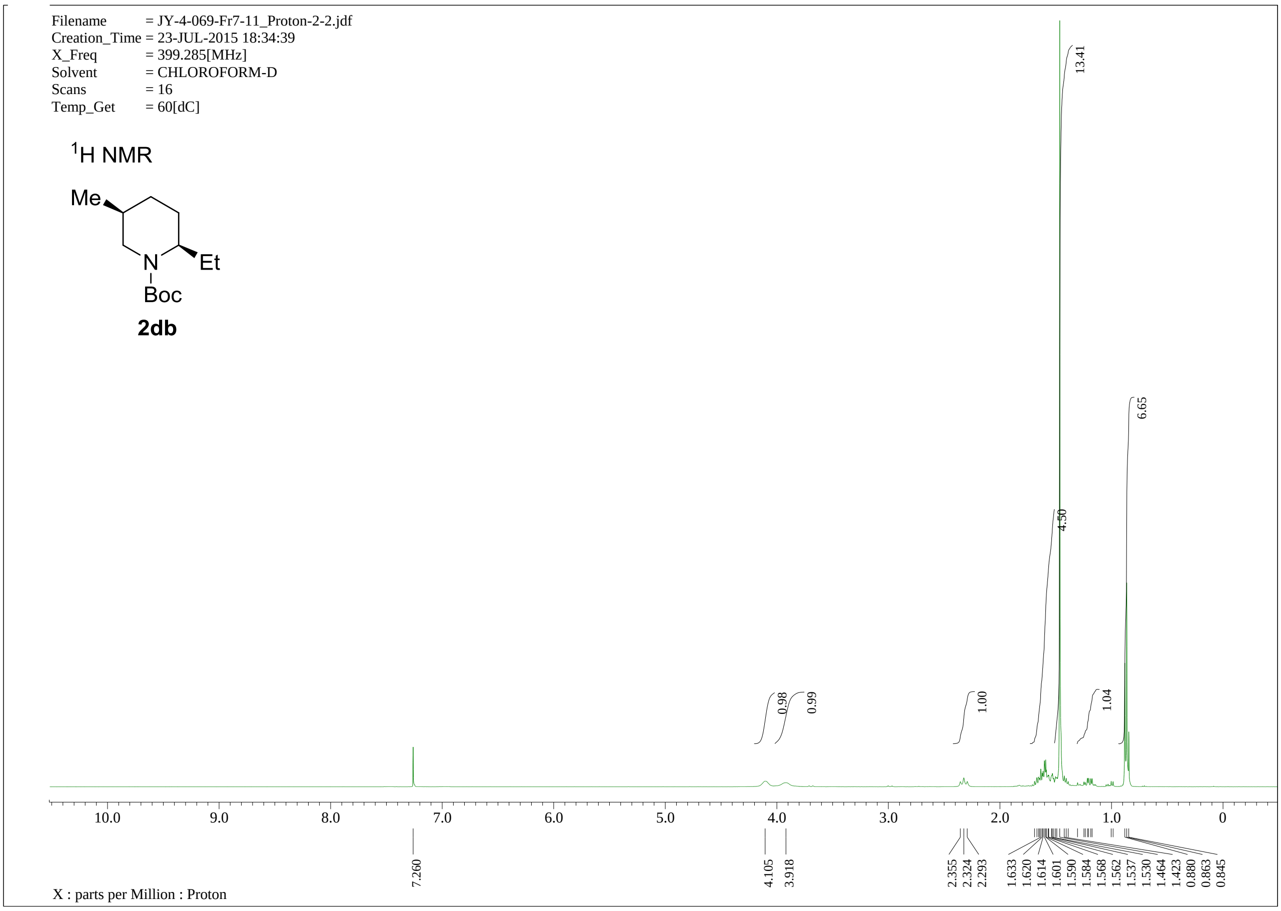
Filename = JY-4-069-Fr7-11_Proton-2-2.jdf
Creation_Time = 23-JUL-2015 18:34:39
X_Freq = 399.285[MHz]
Solvent = CHLOROFORM-D
Scans = 16
Temp_Get = 60[dC]
1H NMR
Me
N
Et
Boc
2db
13.41
6.65
4.50
0.98
0.99
1.00
1.04
10.0 9.0 8.0 7.0 6.0 5.0 4.0 3.0 2.0 1.0 0
7.260
4.105
3.918
2.355
2.324
2.293
1.633
1.620
1.614
1.601
1.590
1.584
1.568
1.562
1.537
1.530
1.464
1.423
0.880
0.863
0.845
X : parts per Million : Proton

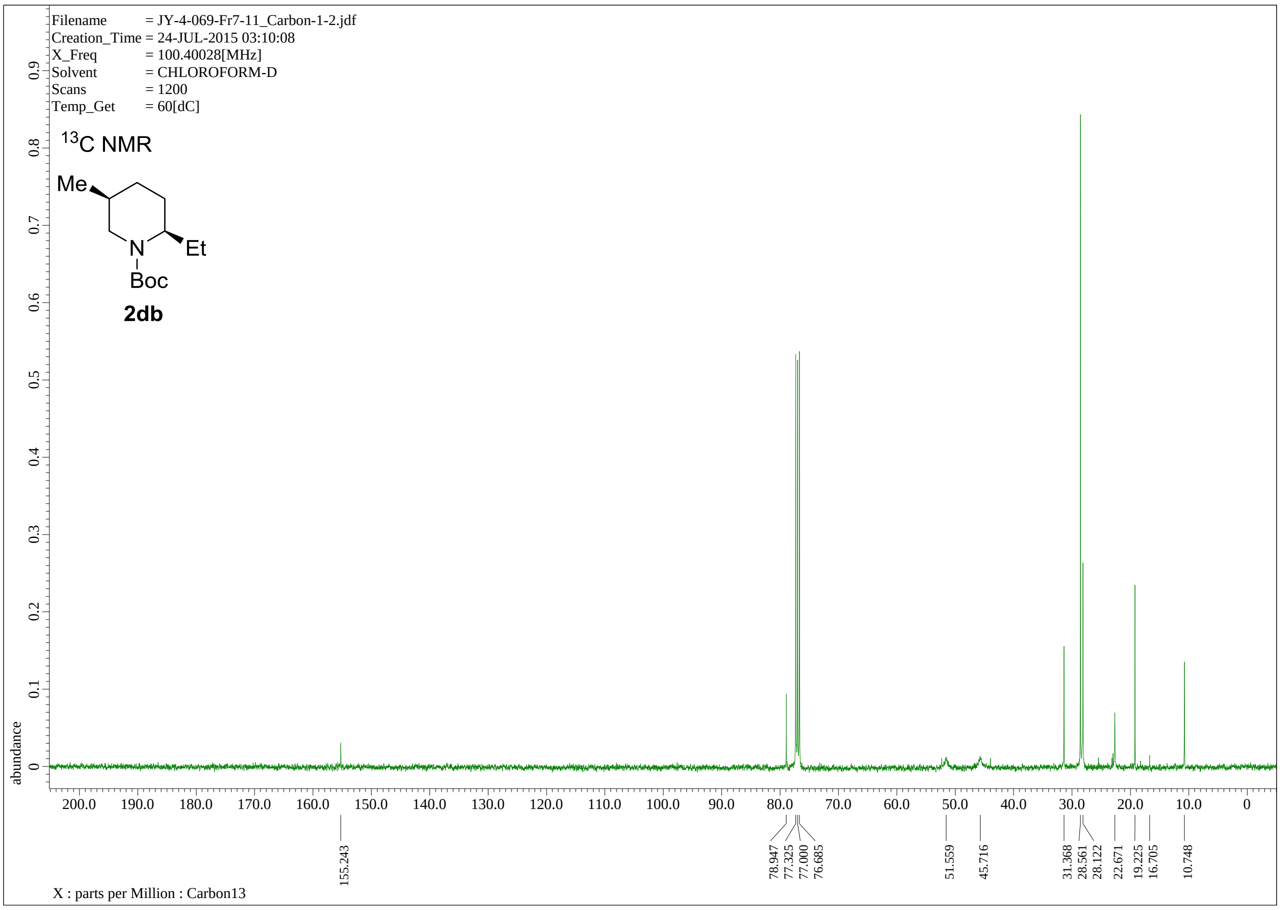
Filename = JY-4-069-Fr7-11_Carbon-1-2.jdf
Creation_Time = 24-JUL-2015 03:10:08
X_Freq = 100.40028[MHz]
Solvent = CHLOROFORM-D
Scans = 1200
Temp_Get = 60[dC]
13C NMR
Me
N
Et
Boc
2db
abundance
0 0.1 0.2 0.3 0.4 0.5 0.6 0.7 0.8 0.9
200.0 190.0 180.0 170.0 160.0 150.0 140.0 130.0 120.0 110.0 100.0 90.0 80.0 70.0 60.0 50.0 40.0 30.0 20.0 10.0 0
155.243
78.947
77.325
77.000
76.685
51.559
45.716
31.368
28.561
28.122
22.671
19.225
16.705
10.748
X : parts per Million : Carbon13

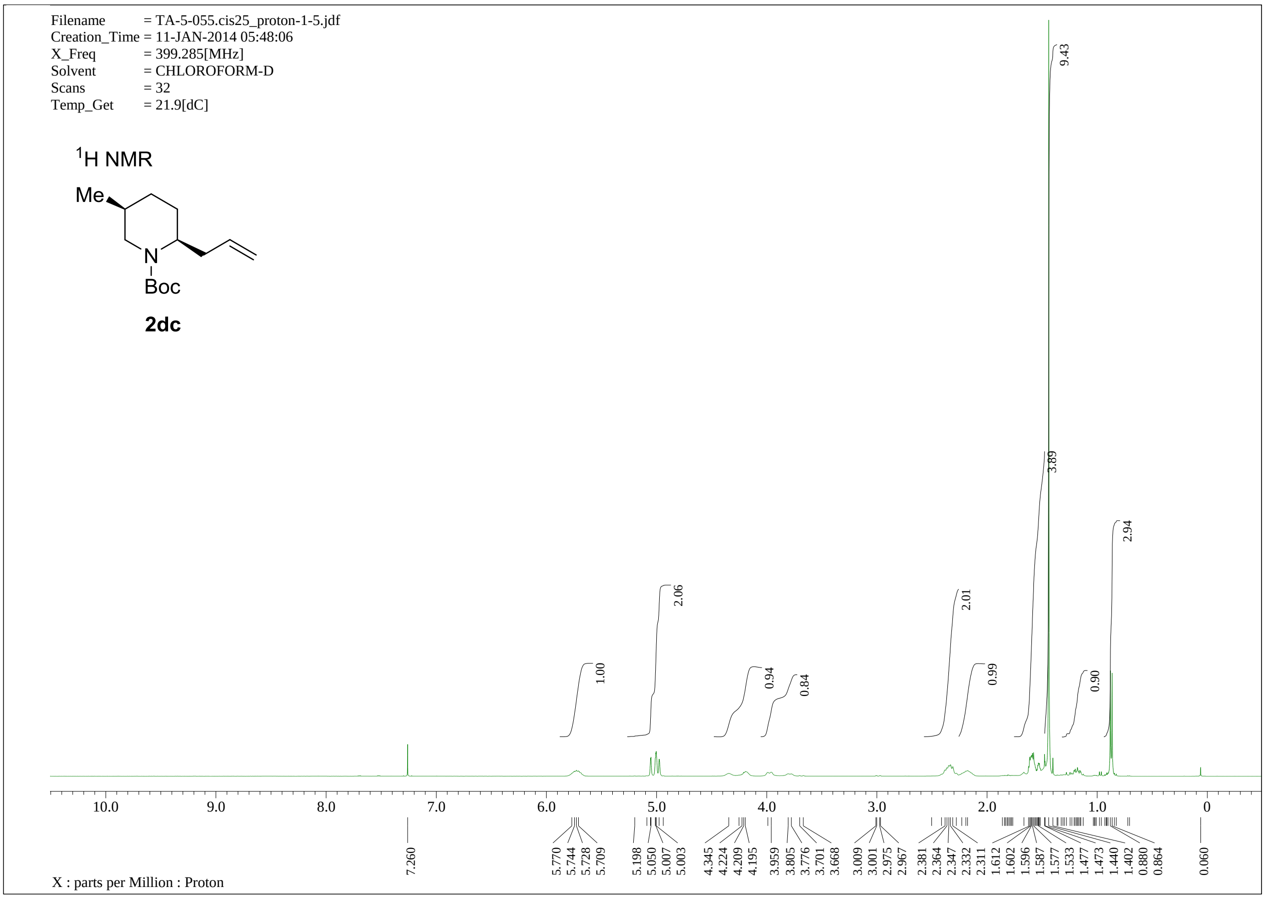

Filename = TA-5-055.cis25_proton-1-5.jdf
Creation_Time = 11-JAN-2014 05:48:06
X_Freq = 399.285[MHz]
Solvent = CHLOROFORM-D
Scans = 32
Temp_Get = 21.9[dC]
1H NMR
Me
N
Boc
2dc
9.43
3.89
2.94
2.06
2.01
1.00
0.94
0.84
0.99
0.90
10.0
9.0
8.0
7.0
6.0
5.0
4.0
3.0
2.0
1.0
0
7.260
5.770
5.744
5.728
5.709
5.198
5.050
5.007
5.003
4.345
4.224
4.209
4.195
3.959
3.805
3.776
3.701
3.668
3.009
3.001
2.975
2.967
2.381
2.364
2.347
2.332
2.311
1.612
1.602
1.596
1.587
1.577
1.533
1.477
1.473
1.440
1.402
0.880
0.864
0.060
X : parts per Million : Proton

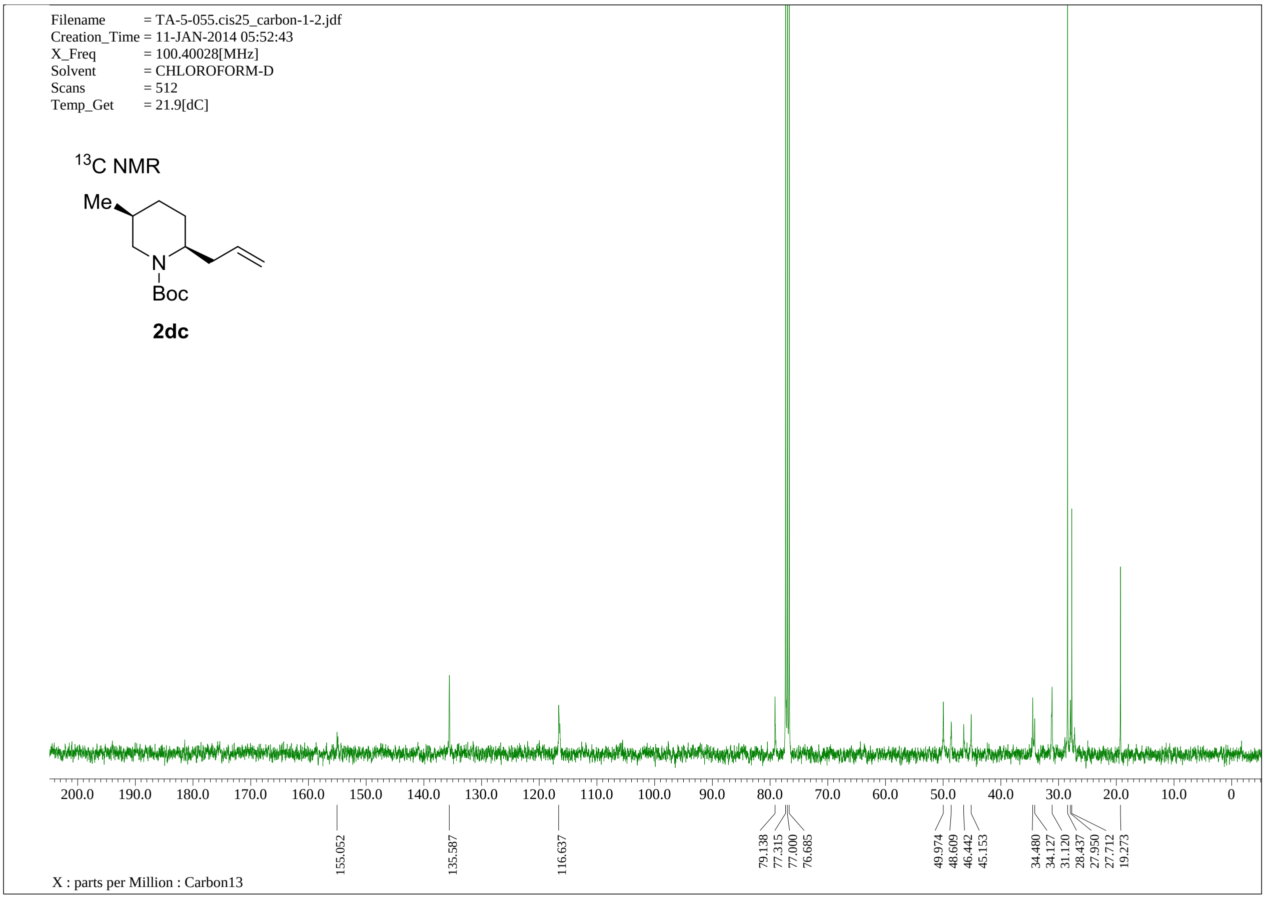

Filename = TA-5-055.cis25_carbon-1-2.jdf
Creation_Time = 11-JAN-2014 05:52:43
X_Freq = 100.40028[MHz]
Solvent = CHLOROFORM-D
Scans = 512
Temp_Get = 21.9[dC]
13C NMR
Me
N
Boc
2dc
200.0
190.0
180.0
170.0
160.0
150.0
140.0
130.0
120.0
110.0
100.0
90.0
80.0
70.0
60.0
50.0
40.0
30.0
20.0
10.0
0
155.052
135.587
116.637
79.138
77.315
77.000
76.685
49.974
48.609
46.442
45.153
34.480
34.127
31.120
28.437
27.950
27.712
19.273
X : parts per Million : Carbon13

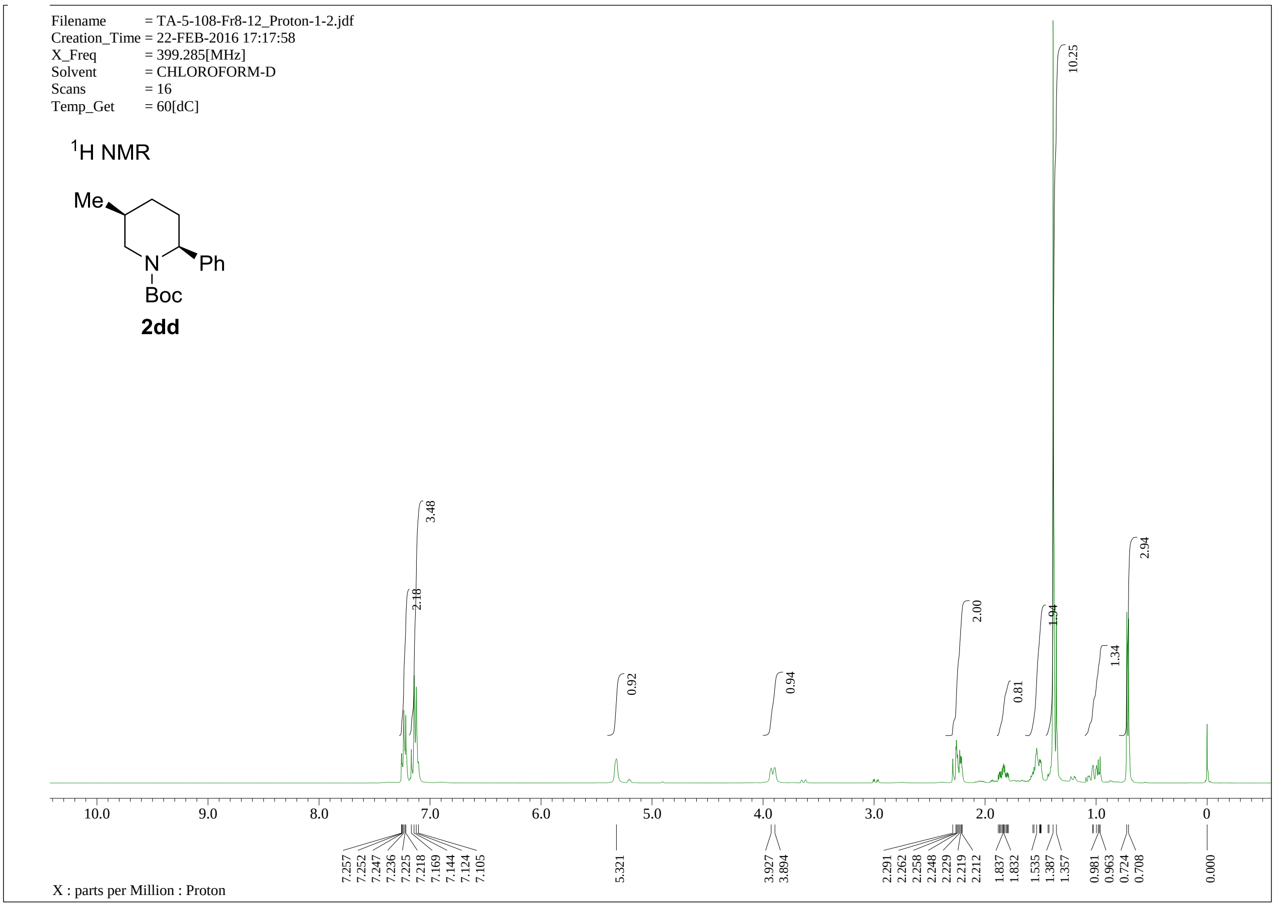
Filename = TA-5-108-Fr8-12_Proton-1-2.jdf
Creation_Time = 22-FEB-2016 17:17:58
X_Freq = 399.285[MHz]
Solvent = CHLOROFORM-D
Scans = 16
Temp_Get = 60[dC]
1H NMR
Me
N
Ph
Boc
2dd
X : parts per Million : Proton

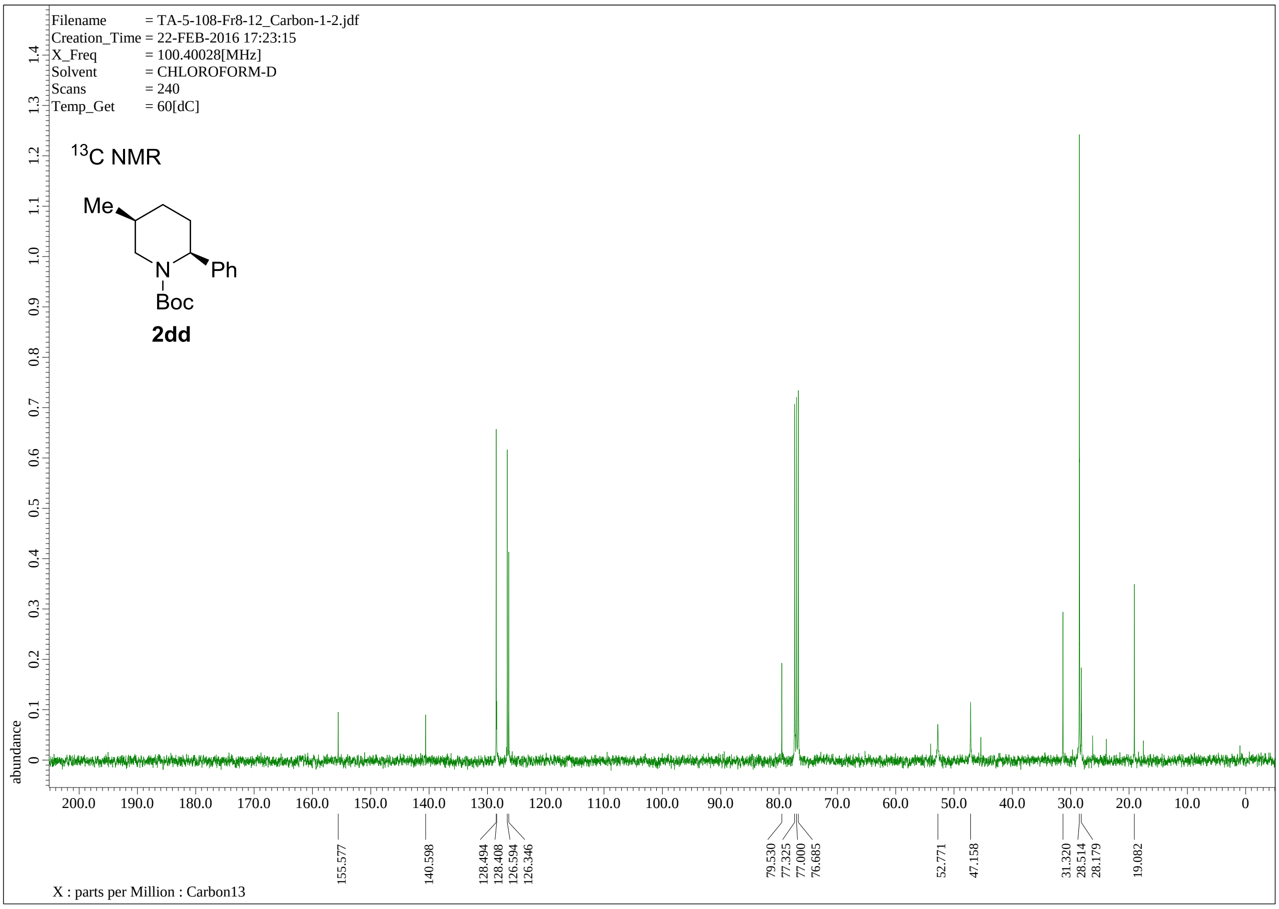
Filename = TA-5-108-Fr8-12_Carbon-1-2.jdf
Creation_Time = 22-FEB-2016 17:23:15
X_Freq = 100.40028[MHz]
Solvent = CHLOROFORM-D
Scans = 240
Temp_Get = 60[dC]
13C NMR
Me
N
Ph
Boc
2dd
abundance
155.577
140.598
128.494
128.408
126.594
126.346
79.530
77.325
77.000
76.685
52.771
47.158
31.320
28.514
28.179
19.082
X : parts per Million : Carbon13

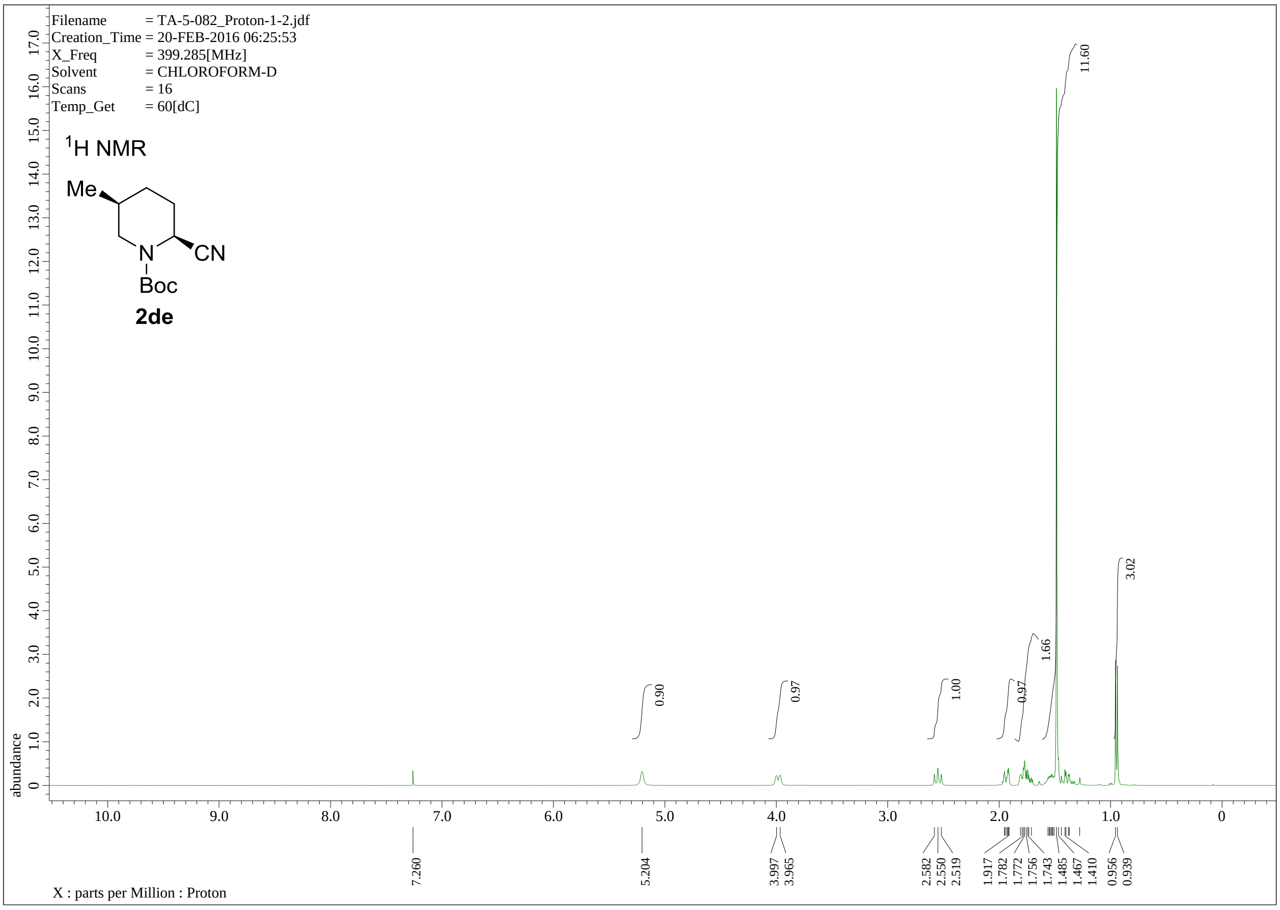
Filename = TA-5-082_Proton-1-2.jdf
Creation_Time = 20-FEB-2016 06:25:53
X_Freq = 399.285[MHz]
Solvent = CHLOROFORM-D
Scans = 16
Temp_Get = 60[dC]
1H NMR
Me
N
CN
Boc
2de
0.90
0.97
1.00
0.97
1.66
11.60
3.02
7.260
5.204
3.997
3.965
2.582
2.550
2.519
1.917
1.782
1.772
1.756
1.743
1.485
1.467
1.410
0.956
0.939
X : parts per Million : Proton
abundance

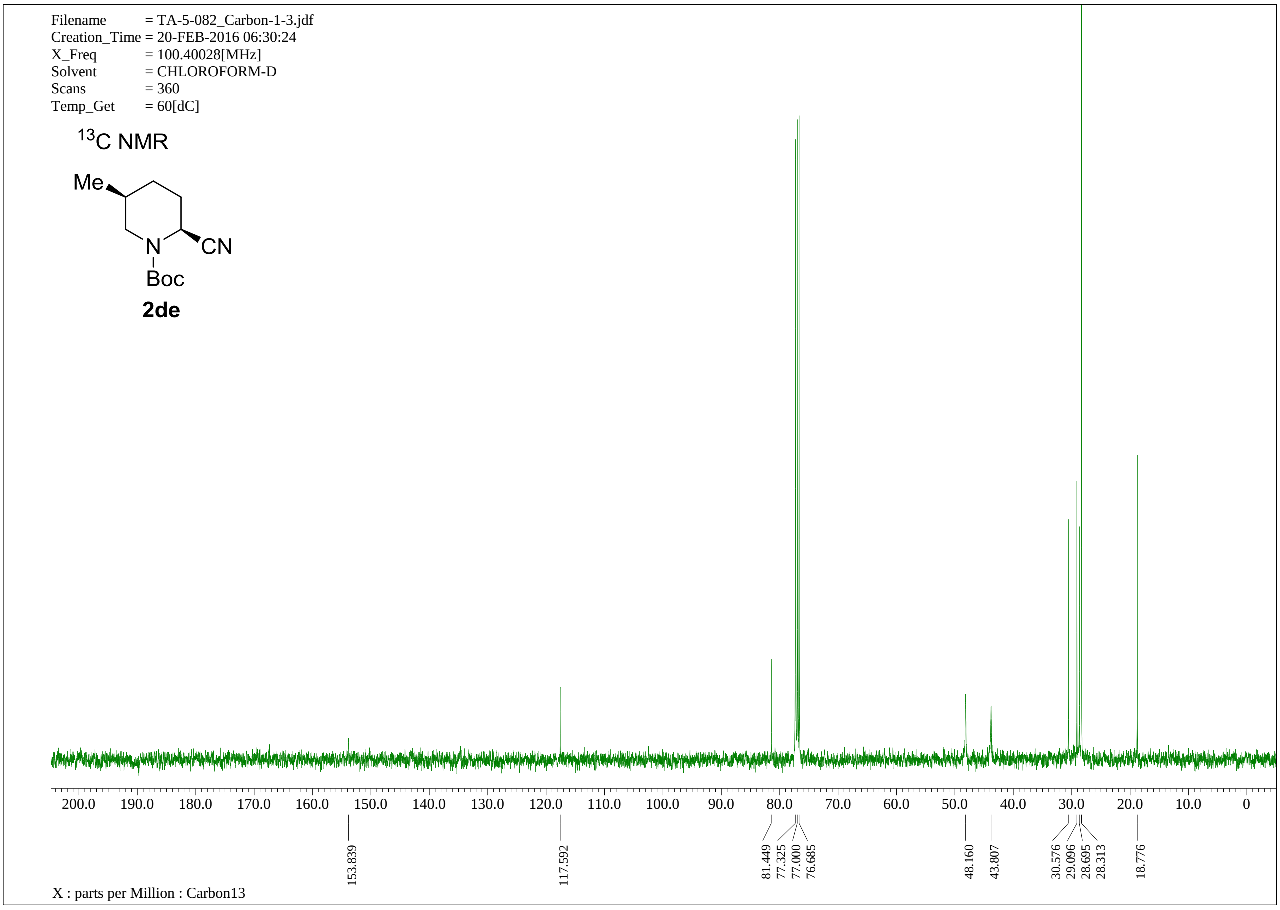
Filename = TA-5-082_Carbon-1-3.jdf
Creation_Time = 20-FEB-2016 06:30:24
X_Freq = 100.40028[MHz]
Solvent = CHLOROFORM-D
Scans = 360
Temp_Get = 60[dC]
13C NMR
Me
N
CN
Boc
2de
153.839
117.592
81.449
77.325
77.000
76.685
48.160
43.807
30.576
29.096
28.695
28.313
18.776
X : parts per Million : Carbon13

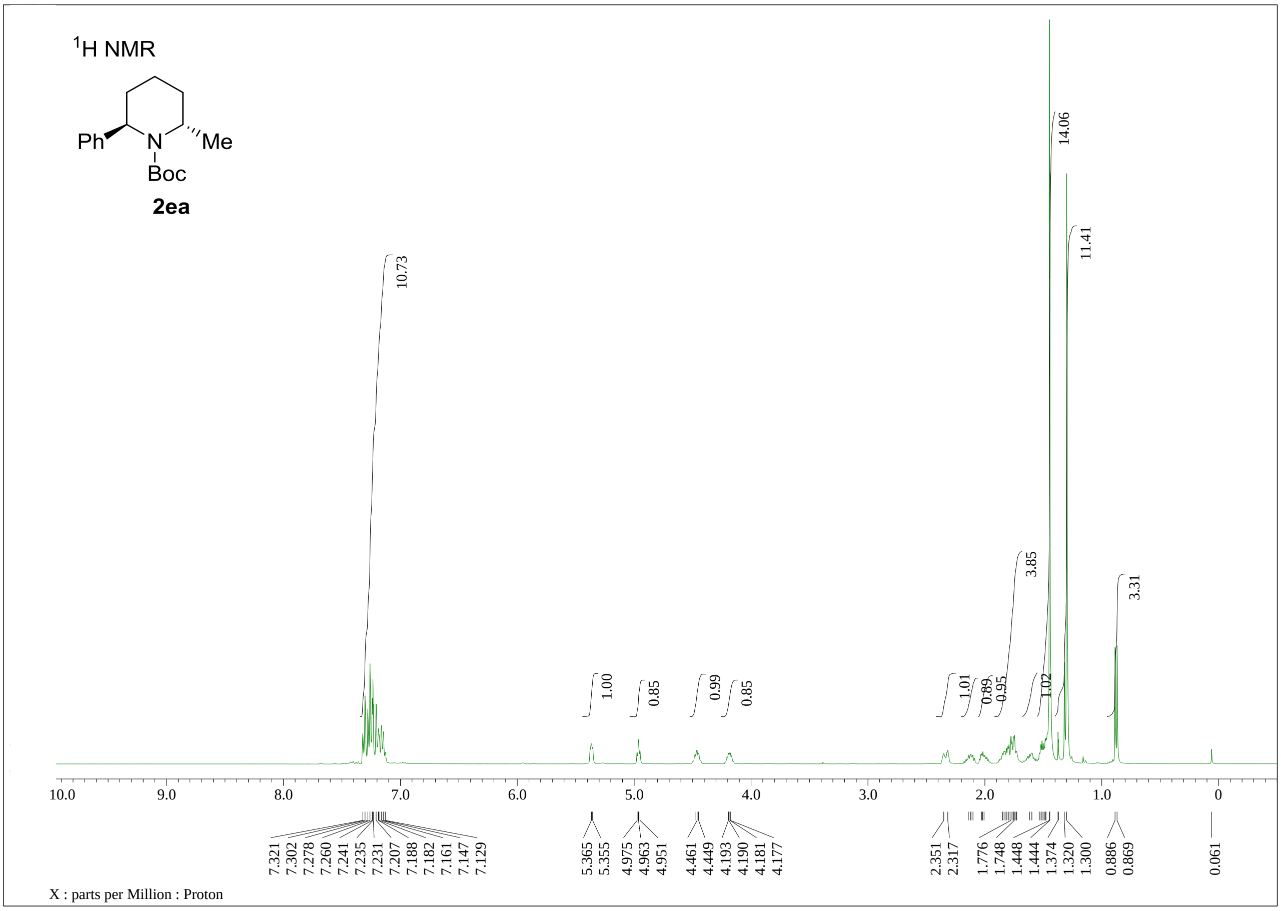

1H NMR
Ph
N
Me
Boc
2ea
10.73
14.06
11.41
3.85
3.31
1.00
0.85
0.99
0.85
1.01
0.89
0.95
1.02
10.0 9.0 8.0 7.0 6.0 5.0 4.0 3.0 2.0 1.0 0
7.321 7.302 7.278 7.260 7.241 7.235 7.231 7.207 7.188 7.182 7.161 7.147 7.129
5.365 5.355 4.975 4.963 4.951
4.461 4.449 4.193 4.190 4.181 4.177
2.351 2.317 1.776 1.748 1.448 1.444 1.374 1.320 1.300 0.886 0.869
0.061
X : parts per Million : Proton

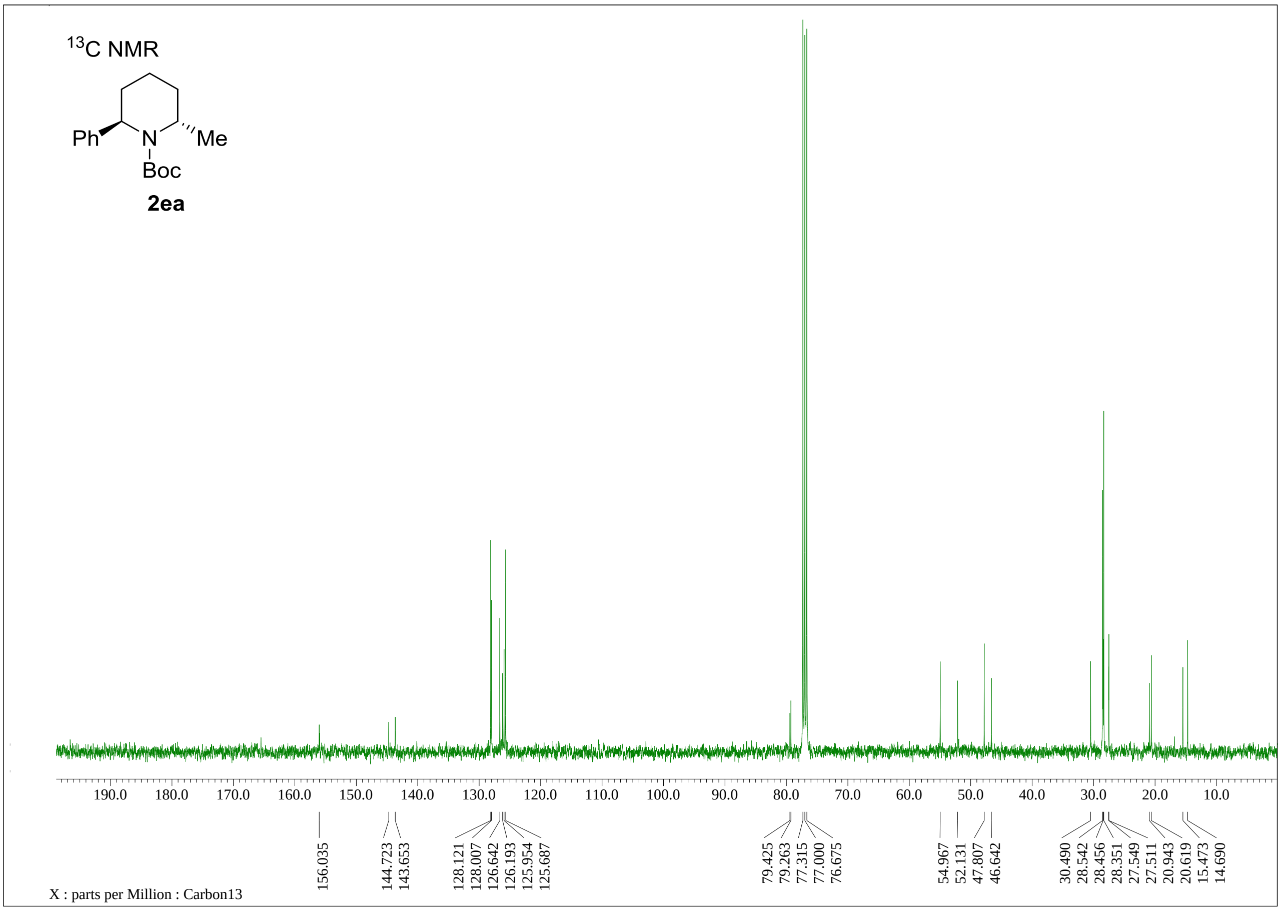

13C NMR
Ph
N
Me
Boc
2ea
190.0 180.0 170.0 160.0 150.0 140.0 130.0 120.0 110.0 100.0 90.0 80.0 70.0 60.0 50.0 40.0 30.0 20.0 10.0
156.035
144.723 143.653
128.121 128.007 126.642 126.193 125.954 125.687
79.425 79.263 77.315 77.000 76.675
54.967 52.131 47.807 46.642
30.490 28.542 28.456 28.351 27.549 27.511 20.943 20.619 15.473 14.690
X : parts per Million : Carbon13

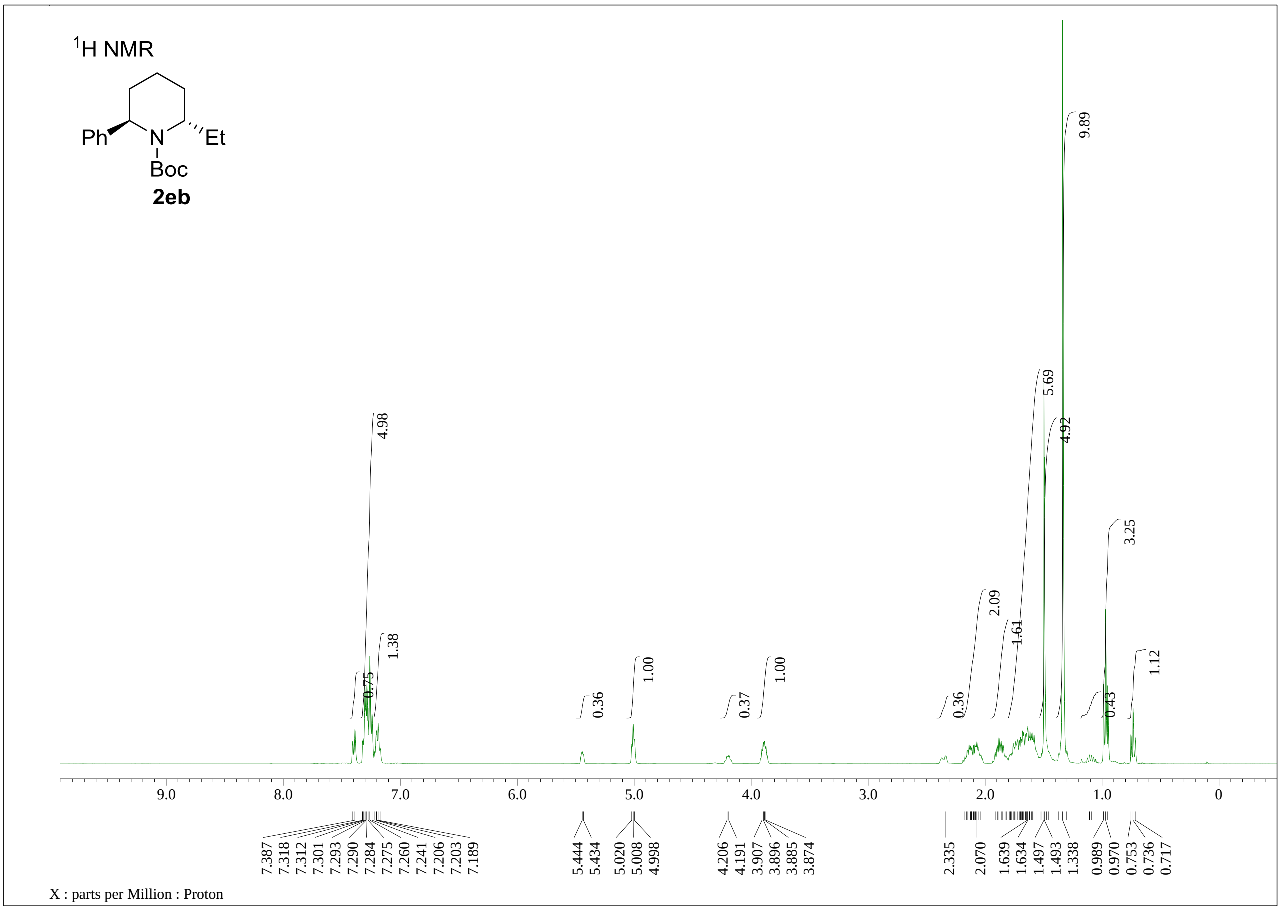
^{1}H NMR
Ph
N
Et
Boc
2eb
7.387 7.318 7.312 7.301 7.293 7.290 7.284 7.275 7.260 7.241 7.206 7.203 7.189
5.444 5.434 5.020 5.008 4.998
4.206 4.191 3.907 3.896 3.885 3.874
2.335 2.070 1.639 1.634 1.497 1.493 1.338
0.989 0.970 0.753 0.736 0.717
X : parts per Million : Proton

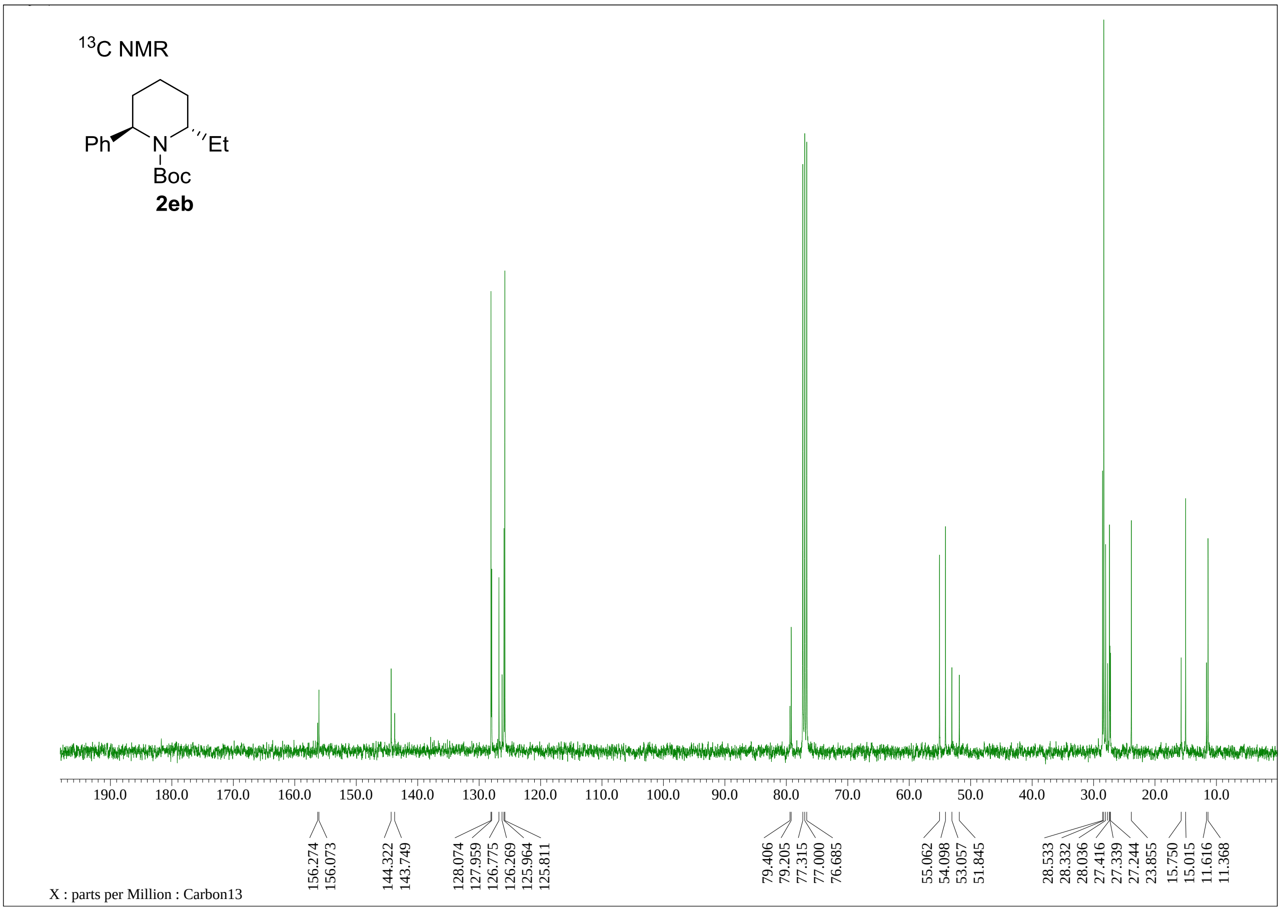
^{13}C NMR
Ph
N
Et
Boc
2eb
156.274 156.073
144.322 143.749
128.074 127.959 126.775 126.269 125.964 125.811
79.406 79.205 77.315 77.000 76.685
55.062 54.098 53.057 51.845
28.533 28.332 28.036 27.416 27.339 27.244 23.855
15.750 15.015 11.616 11.368
X : parts per Million : Carbon13

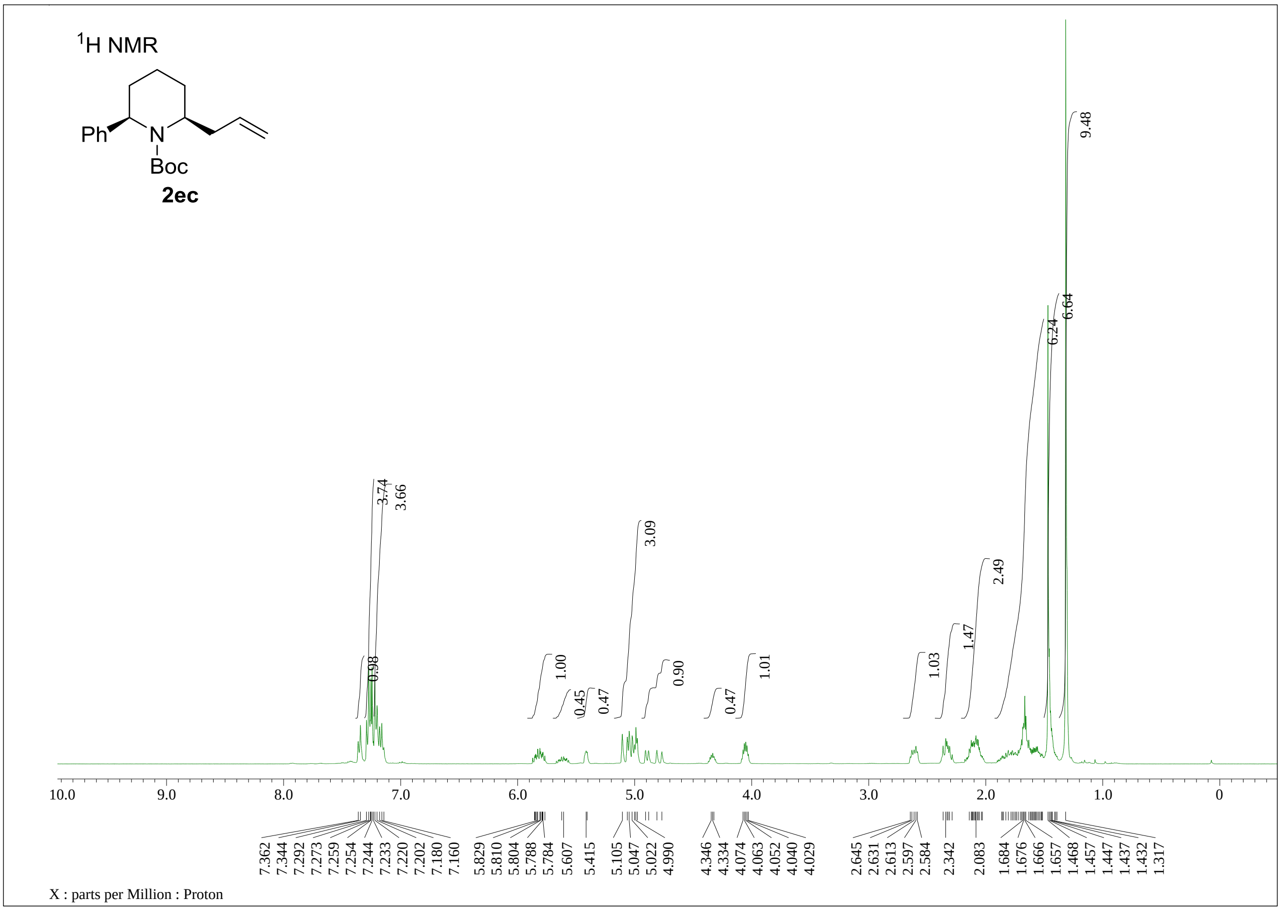
1H NMR
Ph
N
Boc
2ec
3.74
3.66
0.98
1.00
0.45
0.47
3.09
0.90
0.47
1.01
1.03
1.47
2.49
6.24
6.64
9.48
10.0 9.0 8.0 7.0 6.0 5.0 4.0 3.0 2.0 1.0 0
7.362 7.344 7.292 7.273 7.259 7.254 7.244 7.233 7.220 7.202 7.180 7.160
5.829 5.810 5.804 5.788 5.784 5.607 5.415
5.105 5.047 5.022 4.990
4.346 4.334 4.074 4.063 4.052 4.040 4.029
2.645 2.631 2.613 2.597 2.584 2.342 2.083
1.684 1.676 1.666 1.657 1.468 1.457 1.447 1.437 1.432 1.317
X : parts per Million : Proton

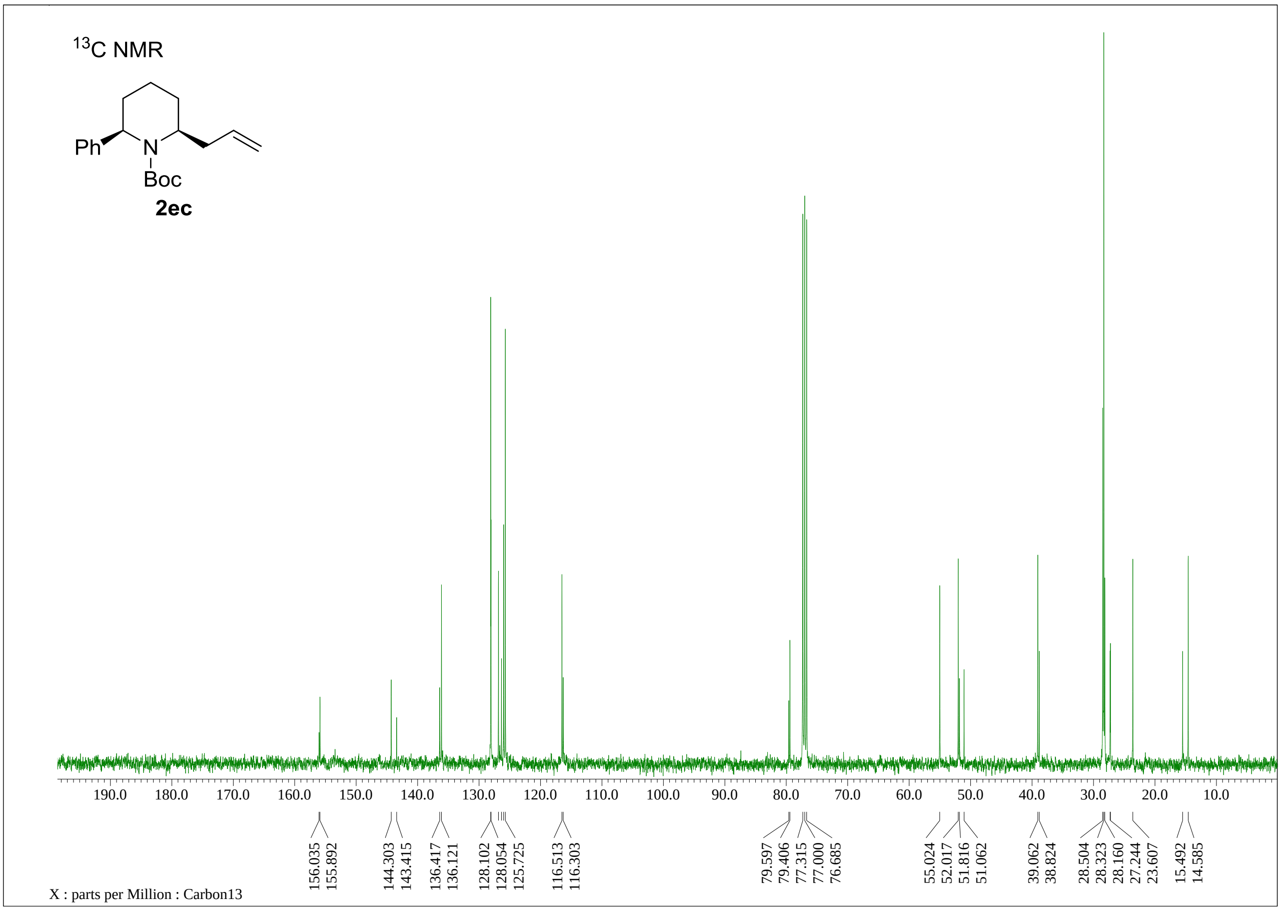
13C NMR
Ph
N
Boc
2ec
190.0 180.0 170.0 160.0 150.0 140.0 130.0 120.0 110.0 100.0 90.0 80.0 70.0 60.0 50.0 40.0 30.0 20.0 10.0
156.035 155.892
144.303 143.415
136.417 136.121
128.102 128.054 125.725
116.513 116.303
79.597 79.406 77.315 77.000 76.685
55.024 52.017 51.816 51.062
39.062 38.824
28.504 28.323 28.160 27.244 23.607
15.492 14.585
X : parts per Million : Carbon13

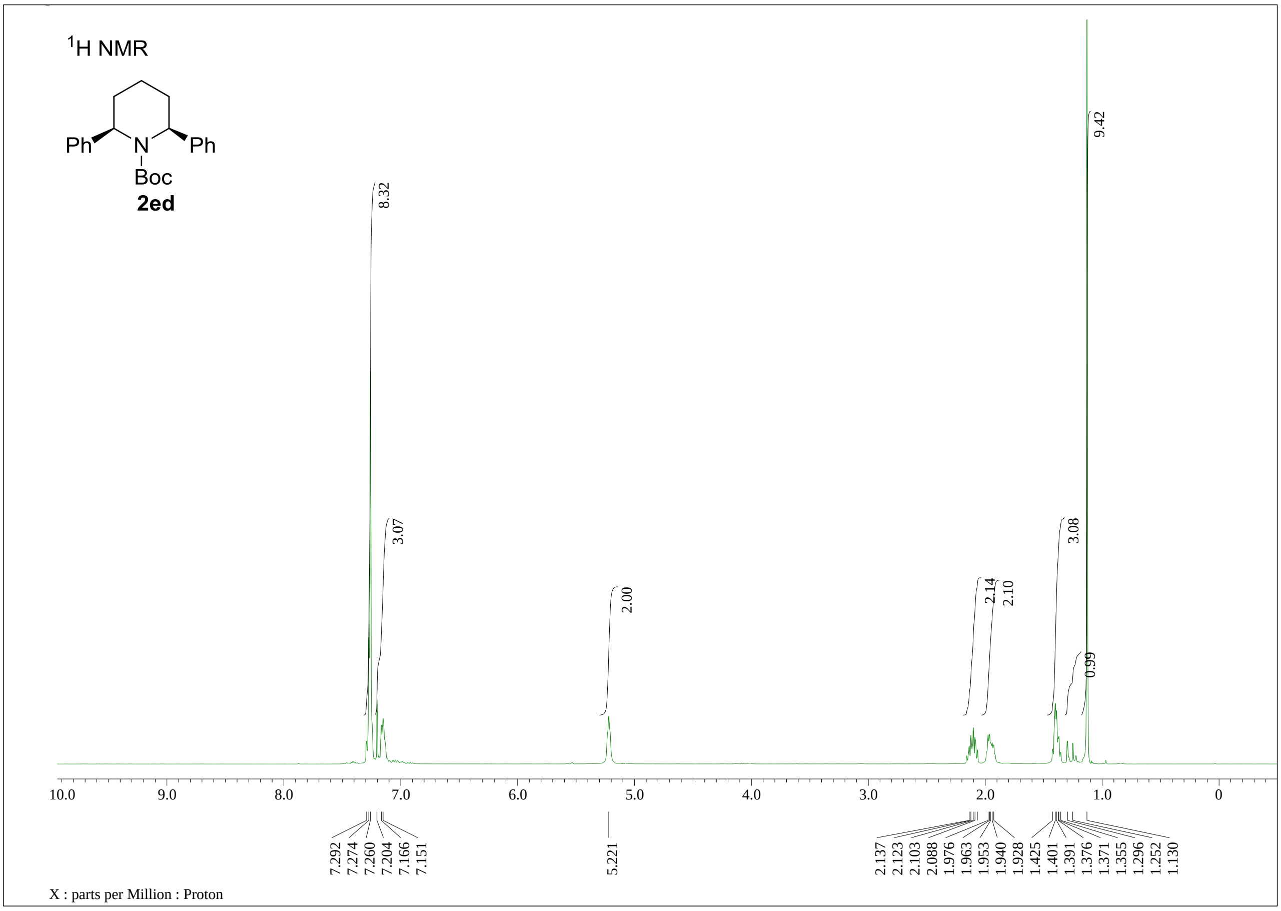
1H NMR
Ph
N
Ph
Boc
2ed
8.32
3.07
2.00
2.14
2.10
3.08
0.99
9.42
10.0 9.0 8.0 7.0 6.0 5.0 4.0 3.0 2.0 1.0 0
7.292 7.274 7.260 7.204 7.166 7.151
5.221
2.137 2.123 2.103 2.088 1.976 1.963 1.953 1.940 1.928 1.425 1.401 1.391 1.376 1.371 1.355 1.296 1.252 1.130
X : parts per Million : Proton

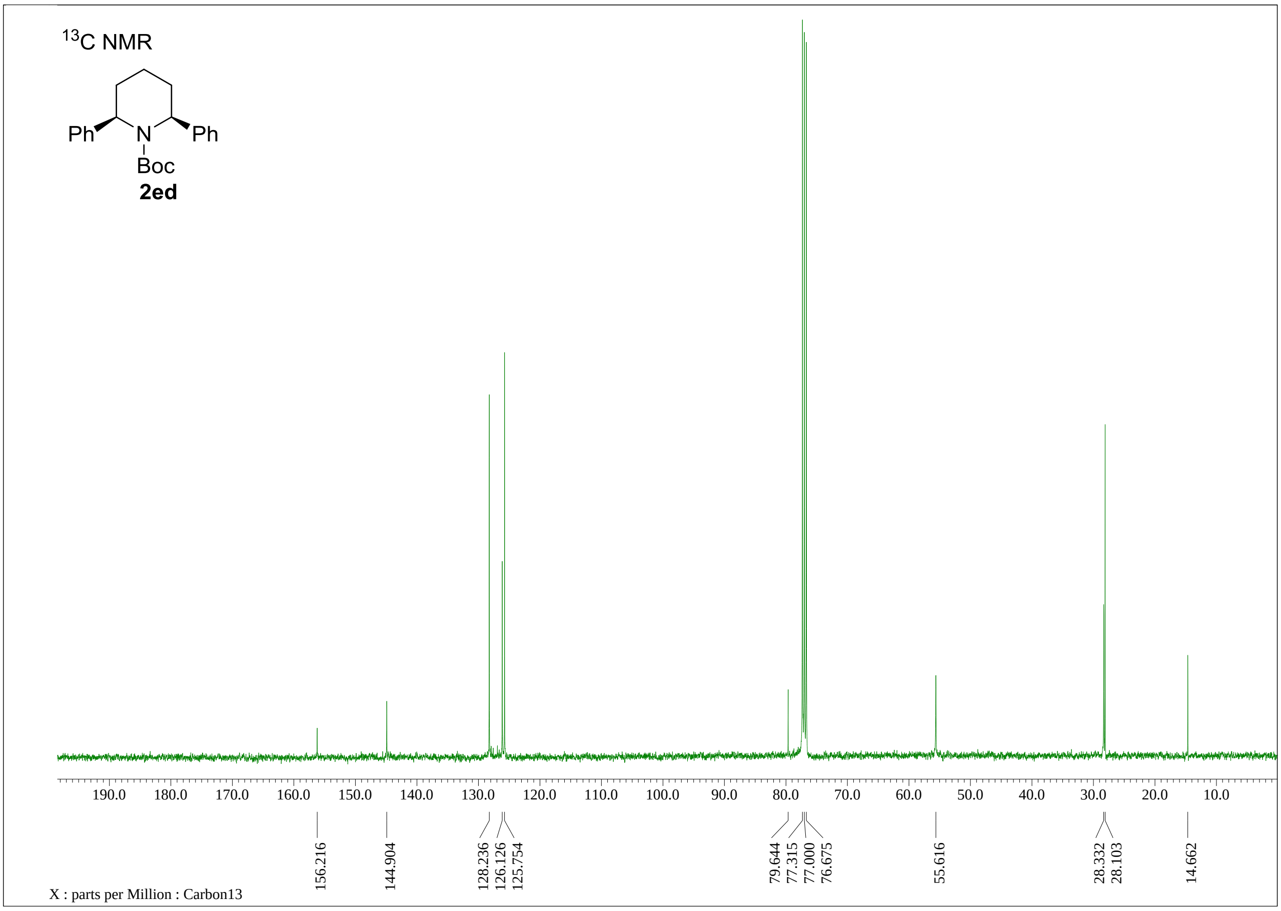
13C NMR
Ph
N
Ph
Boc
2ed
190.0 180.0 170.0 160.0 150.0 140.0 130.0 120.0 110.0 100.0 90.0 80.0 70.0 60.0 50.0 40.0 30.0 20.0 10.0
156.216
144.904
128.236 126.126 125.754
79.644 77.315 77.000 76.675
55.616
28.332 28.103
14.662
X : parts per Million : Carbon13

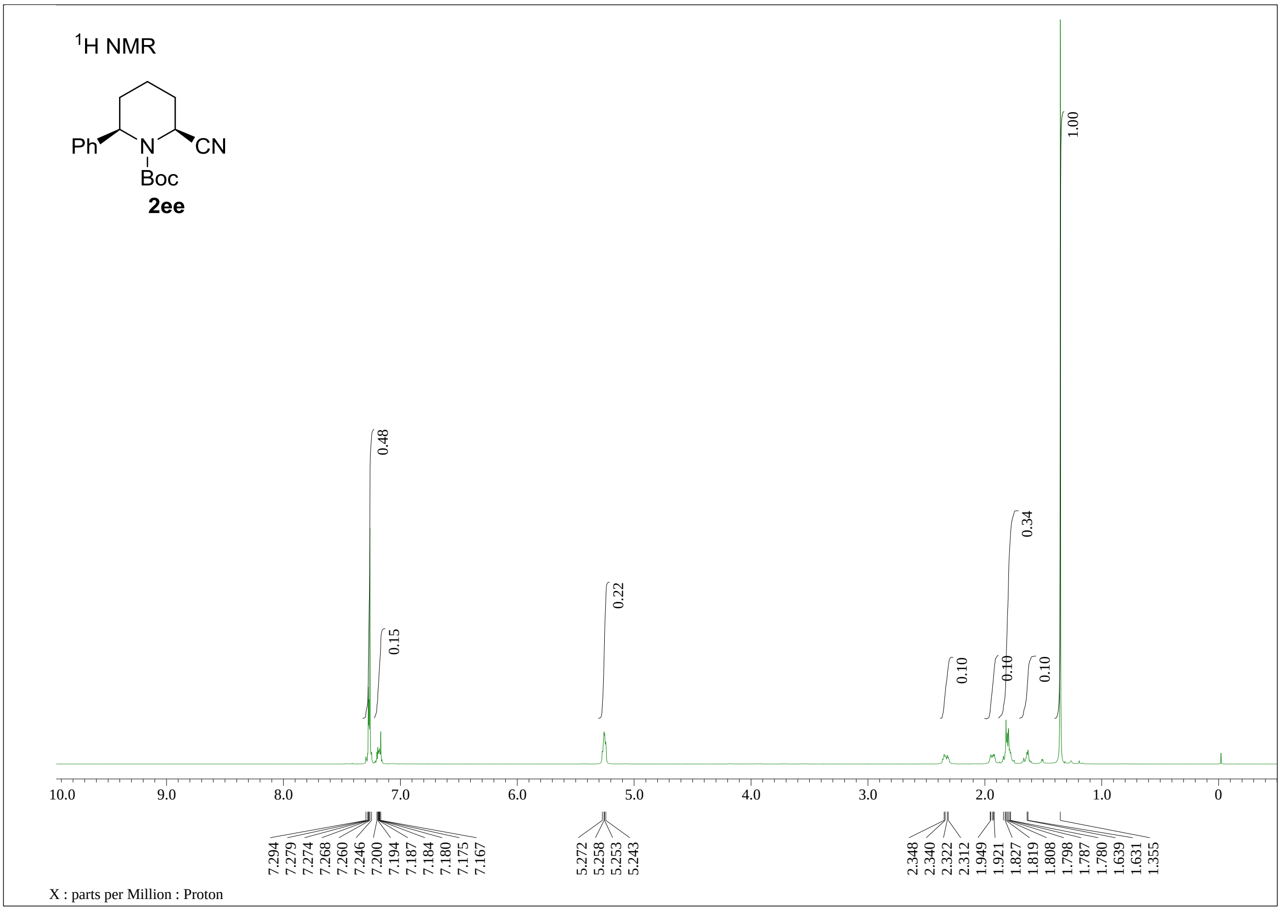

1H NMR
Ph
N
CN
Boc
2ee
0.48
0.15
0.22
0.10
0.10
0.10
0.34
1.00
10.0 9.0 8.0 7.0 6.0 5.0 4.0 3.0 2.0 1.0 0
7.294 7.279 7.274 7.268 7.260 7.246 7.200 7.194 7.187 7.184 7.180 7.175 7.167
5.272 5.258 5.253 5.243
2.348 2.340 2.322 2.312 1.949 1.921 1.827 1.819 1.808 1.798 1.787 1.780 1.639 1.631 1.355
X : parts per Million : Proton

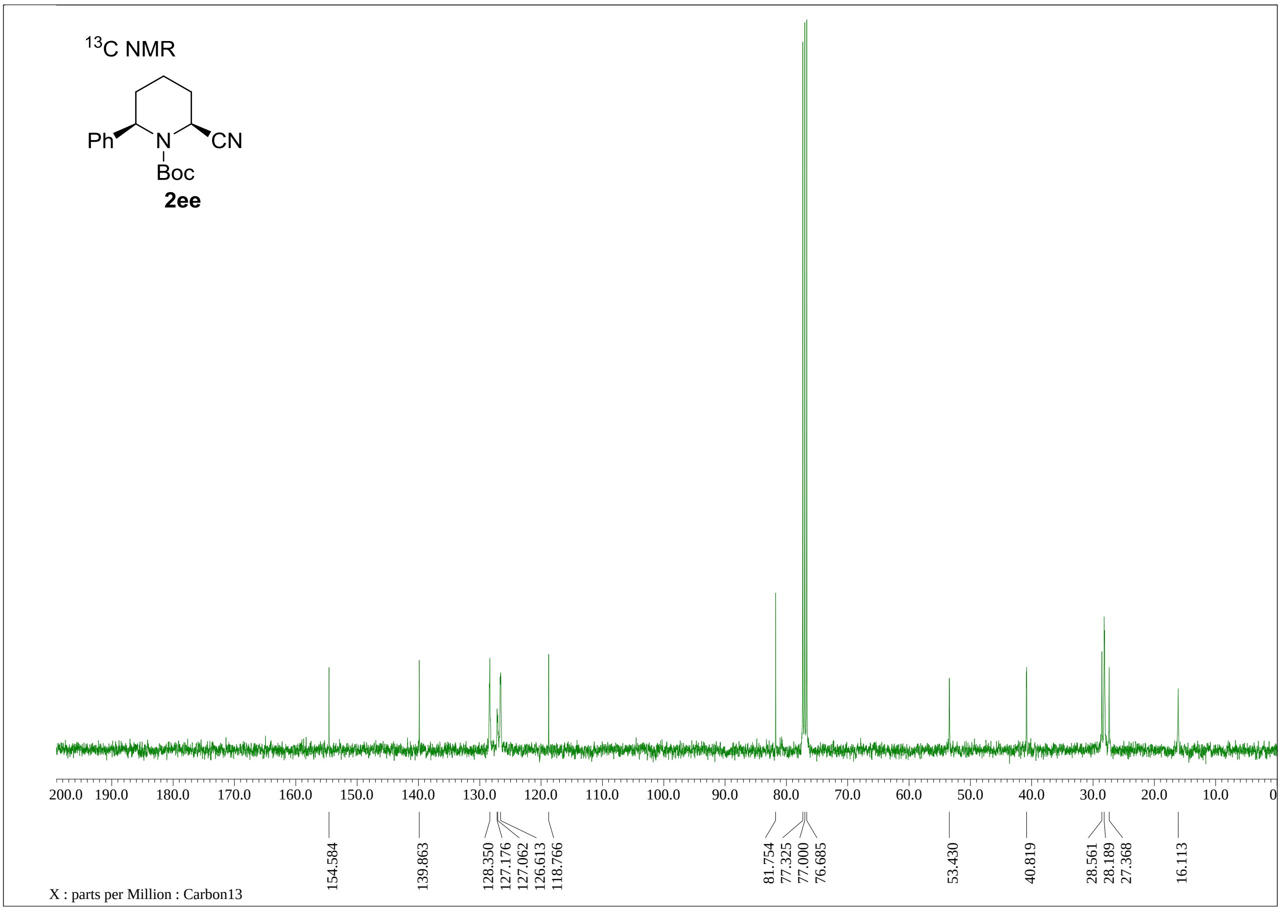

13C NMR
Ph
N
CN
Boc
2ee
200.0 190.0 180.0 170.0 160.0 150.0 140.0 130.0 120.0 110.0 100.0 90.0 80.0 70.0 60.0 50.0 40.0 30.0 20.0 10.0 0
154.584
139.863
128.350 127.176 127.062 126.613 118.766
81.754 77.325 77.000 76.685
53.430
40.819
28.561 28.189 27.368
16.113
X : parts per Million : Carbon13

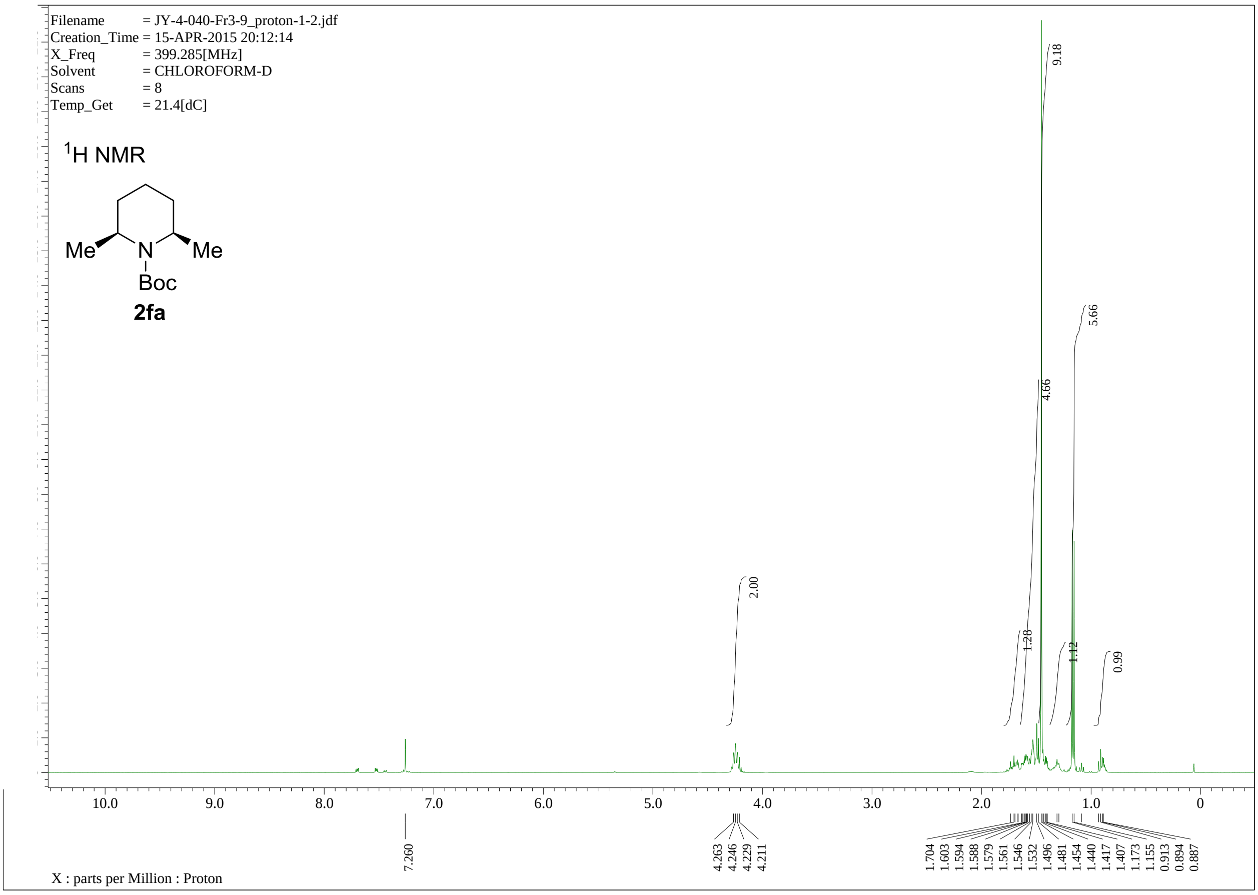
Filename = JY-4-040-Fr3-9_proton-1-2.jdf
Creation_Time = 15-APR-2015 20:12:14
X_Freq = 399.285[MHz]
Solvent = CHLOROFORM-D
Scans = 8
Temp_Get = 21.4[dC]
1H NMR
Me
N
Me
Boc
2fa
9.18
5.66
4.66
2.00
1.28
1.12
0.99
10.0
9.0
8.0
7.0
6.0
5.0
4.0
3.0
2.0
1.0
0
7.260
4.263
4.246
4.229
4.211
1.704
1.603
1.594
1.588
1.579
1.561
1.546
1.532
1.496
1.481
1.454
1.440
1.417
1.407
1.173
1.155
0.913
0.894
0.887
X : parts per Million : Proton

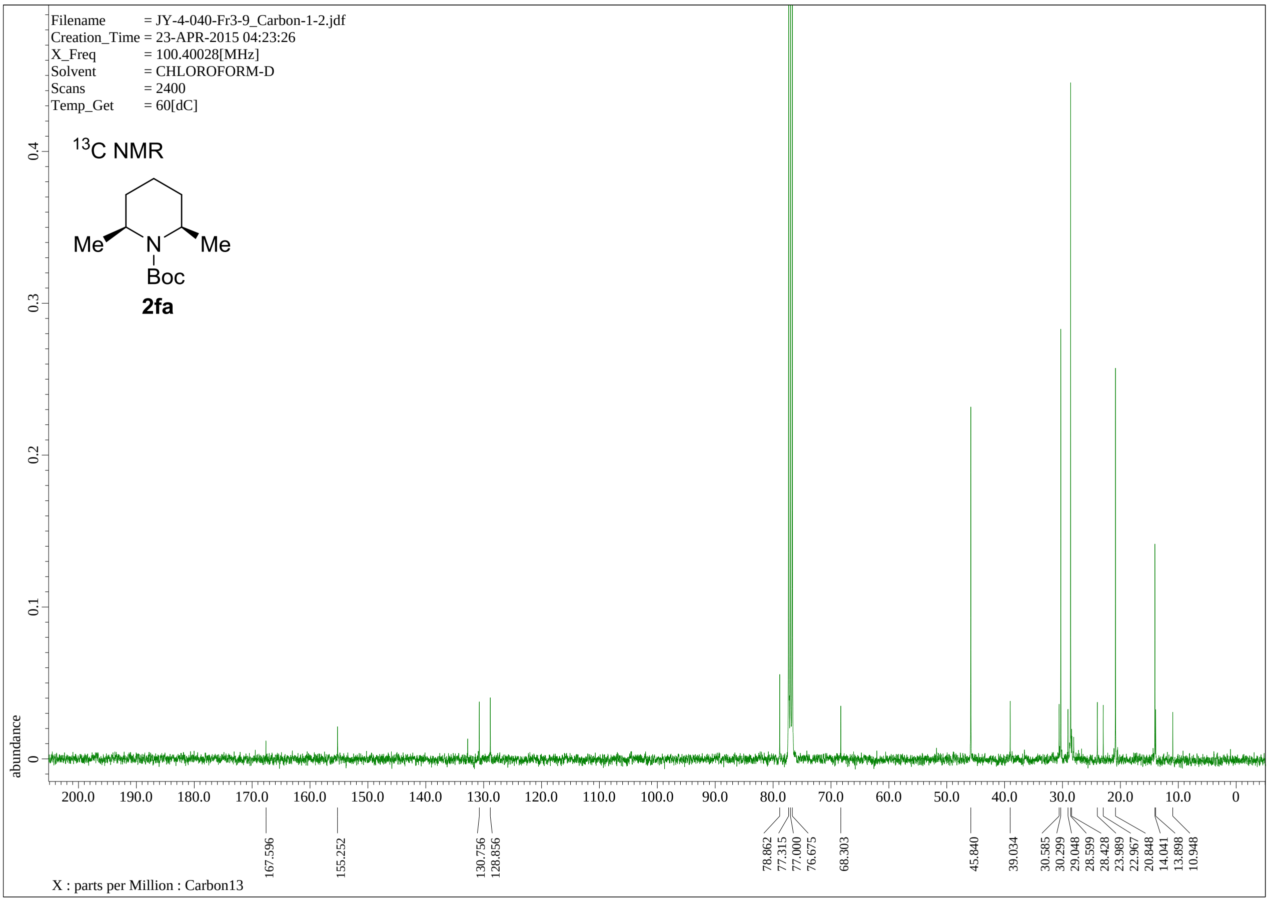
Filename = JY-4-040-Fr3-9_Carbon-1-2.jdf
Creation_Time = 23-APR-2015 04:23:26
X_Freq = 100.40028[MHz]
Solvent = CHLOROFORM-D
Scans = 2400
Temp_Get = 60[dC]
13C NMR
Me
N
Me
Boc
2fa
abundance
0
0.1
0.2
0.3
0.4
200.0
190.0
180.0
170.0
160.0
150.0
140.0
130.0
120.0
110.0
100.0
90.0
80.0
70.0
60.0
50.0
40.0
30.0
20.0
10.0
0
167.596
155.252
130.756
128.856
78.862
77.315
77.000
76.675
68.303
45.840
39.034
30.585
30.299
29.048
28.599
28.428
23.989
22.967
20.848
14.041
13.898
10.948
X : parts per Million : Carbon13

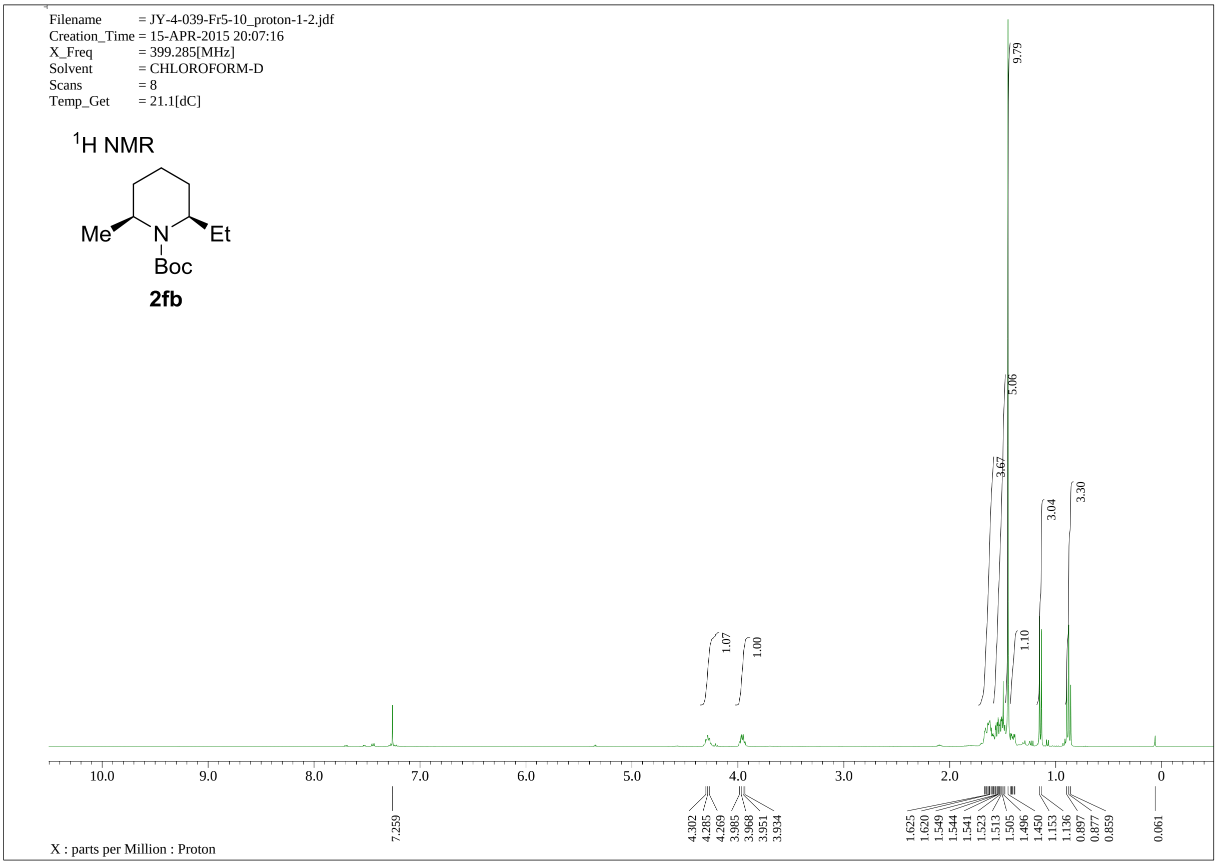
Filename = JY-4-039-Fr5-10_proton-1-2.jdf
Creation_Time = 15-APR-2015 20:07:16
X_Freq = 399.285[MHz]
Solvent = CHLOROFORM-D
Scans = 8
Temp_Get = 21.1[dC]
1H NMR
Me
N
Et
Boc
2fb
9.79
5.06
3.67
3.04
3.30
1.07
1.00
1.10
10.0
9.0
8.0
7.0
6.0
5.0
4.0
3.0
2.0
1.0
0
7.259
4.302
4.285
4.269
3.985
3.968
3.951
3.934
1.625
1.620
1.549
1.544
1.541
1.523
1.513
1.505
1.496
1.450
1.153
1.136
0.897
0.877
0.859
0.061
X : parts per Million : Proton

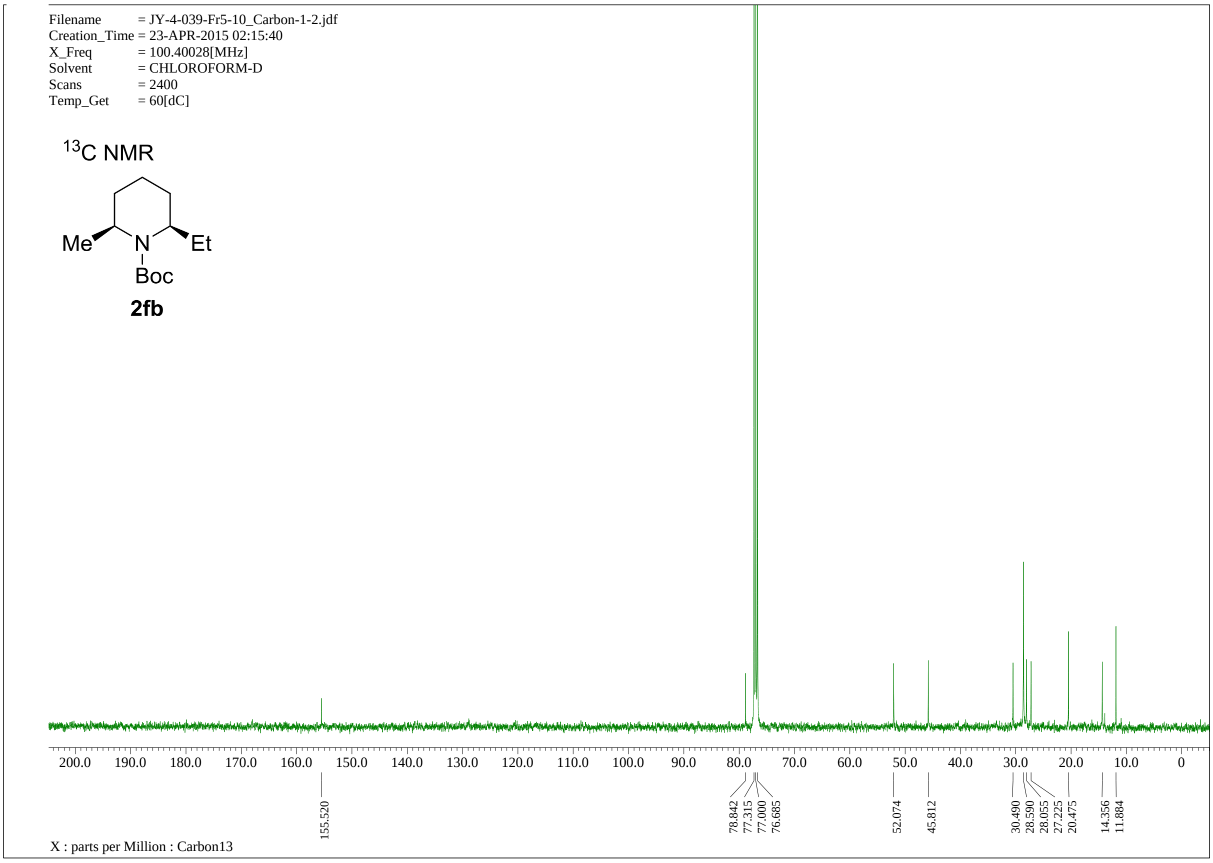
Filename = JY-4-039-Fr5-10_Carbon-1-2.jdf
Creation_Time = 23-APR-2015 02:15:40
X_Freq = 100.40028[MHz]
Solvent = CHLOROFORM-D
Scans = 2400
Temp_Get = 60[dC]
13C NMR
Me
N
Et
Boc
2fb
200.0
190.0
180.0
170.0
160.0
150.0
140.0
130.0
120.0
110.0
100.0
90.0
80.0
70.0
60.0
50.0
40.0
30.0
20.0
10.0
0
155.520
78.842
77.315
77.000
76.685
52.074
45.812
30.490
28.590
28.055
27.225
20.475
14.356
11.884
X : parts per Million : Carbon13

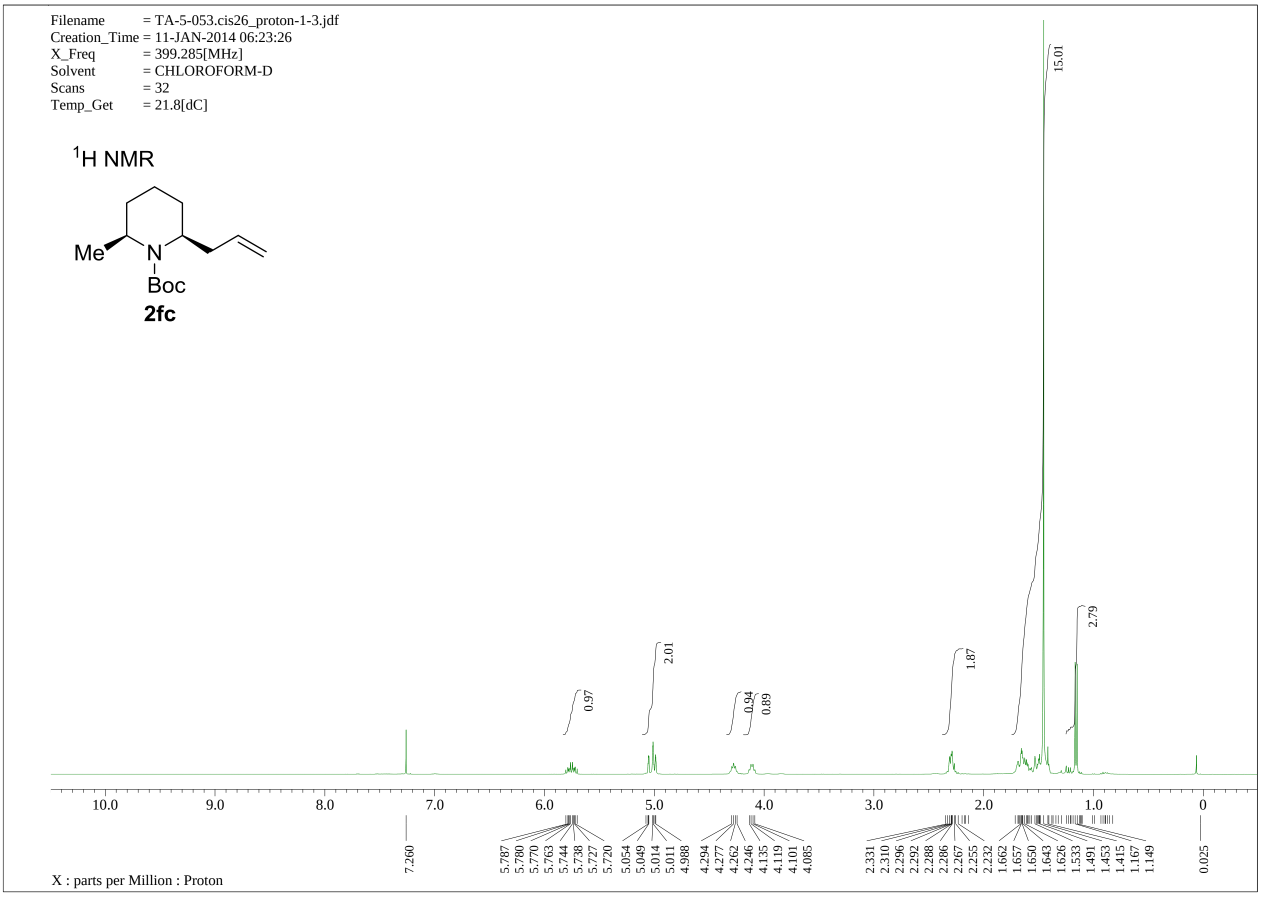
Filename = TA-5-053.cis26_proton-1-3.jdf
Creation_Time = 11-JAN-2014 06:23:26
X_Freq = 399.285[MHz]
Solvent = CHLOROFORM-D
Scans = 32
Temp_Get = 21.8[dC]
1H NMR
Me
N
Boc
2fc
15.01
2.79
2.01
1.87
0.97
0.94
0.89
7.260
5.787 5.780 5.770 5.763 5.744 5.738 5.727 5.720 5.054 5.049 5.014 5.011 4.988 4.294 4.277 4.262 4.246 4.135 4.119 4.101 4.085
2.331 2.310 2.296 2.292 2.288 2.286 2.267 2.255 2.232 1.662 1.657 1.650 1.643 1.626 1.533 1.491 1.453 1.415 1.167 1.149
0.025
X : parts per Million : Proton

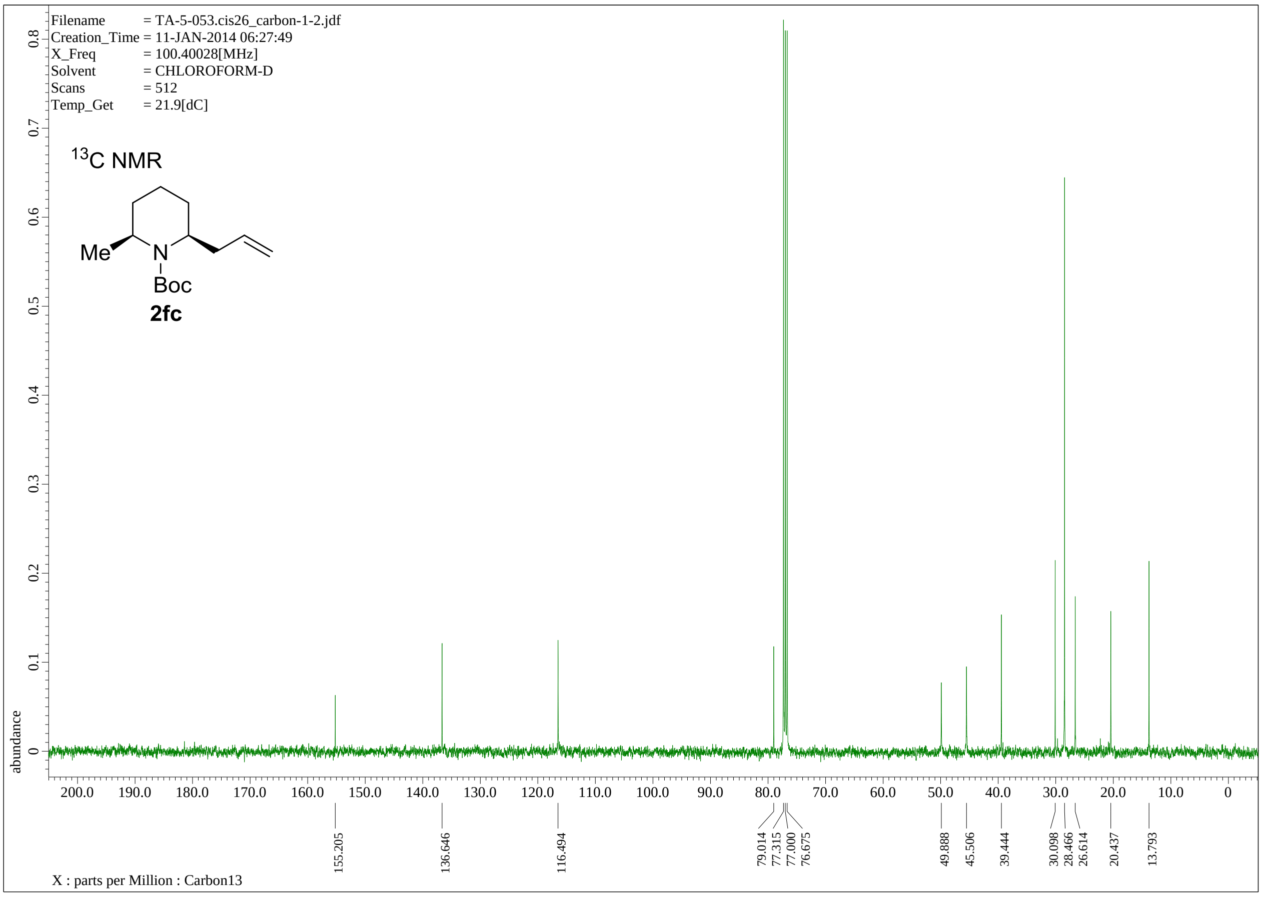
Filename = TA-5-053.cis26_carbon-1-2.jdf
Creation_Time = 11-JAN-2014 06:27:49
X_Freq = 100.40028[MHz]
Solvent = CHLOROFORM-D
Scans = 512
Temp_Get = 21.9[dC]
13C NMR
Me
N
Boc
2fc
abundance
155.205
136.646
116.494
79.014 77.315 77.000 76.675
49.888
45.506
39.444
30.098 28.466 26.614
20.437
13.793
X : parts per Million : Carbon13

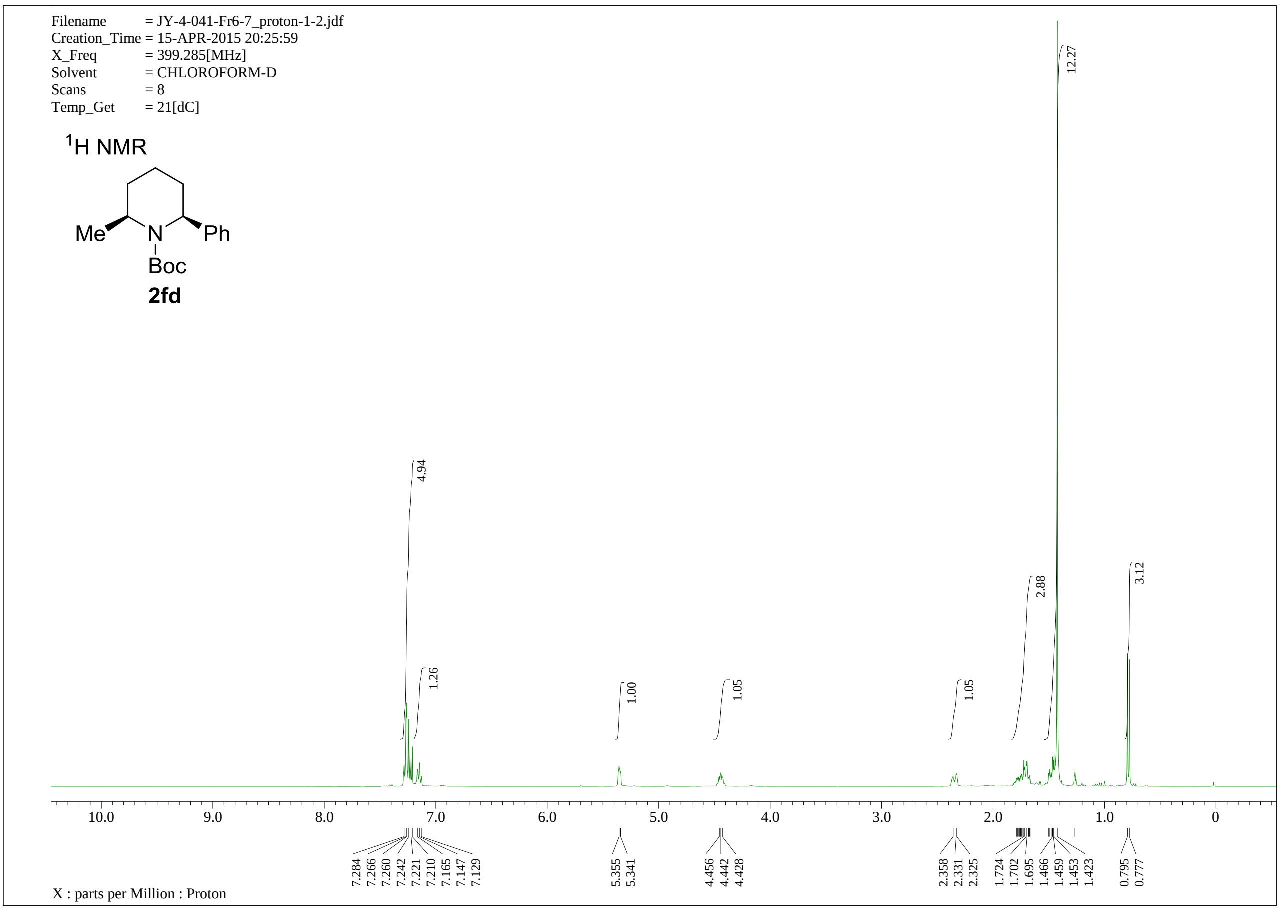
Filename = JY-4-041-Fr6-7_proton-1-2.jdf
Creation_Time = 15-APR-2015 20:25:59
X_Freq = 399.285[MHz]
Solvent = CHLOROFORM-D
Scans = 8
Temp_Get = 21[dC]
1H NMR
Me
N
Ph
Boc
2fd
X : parts per Million : Proton

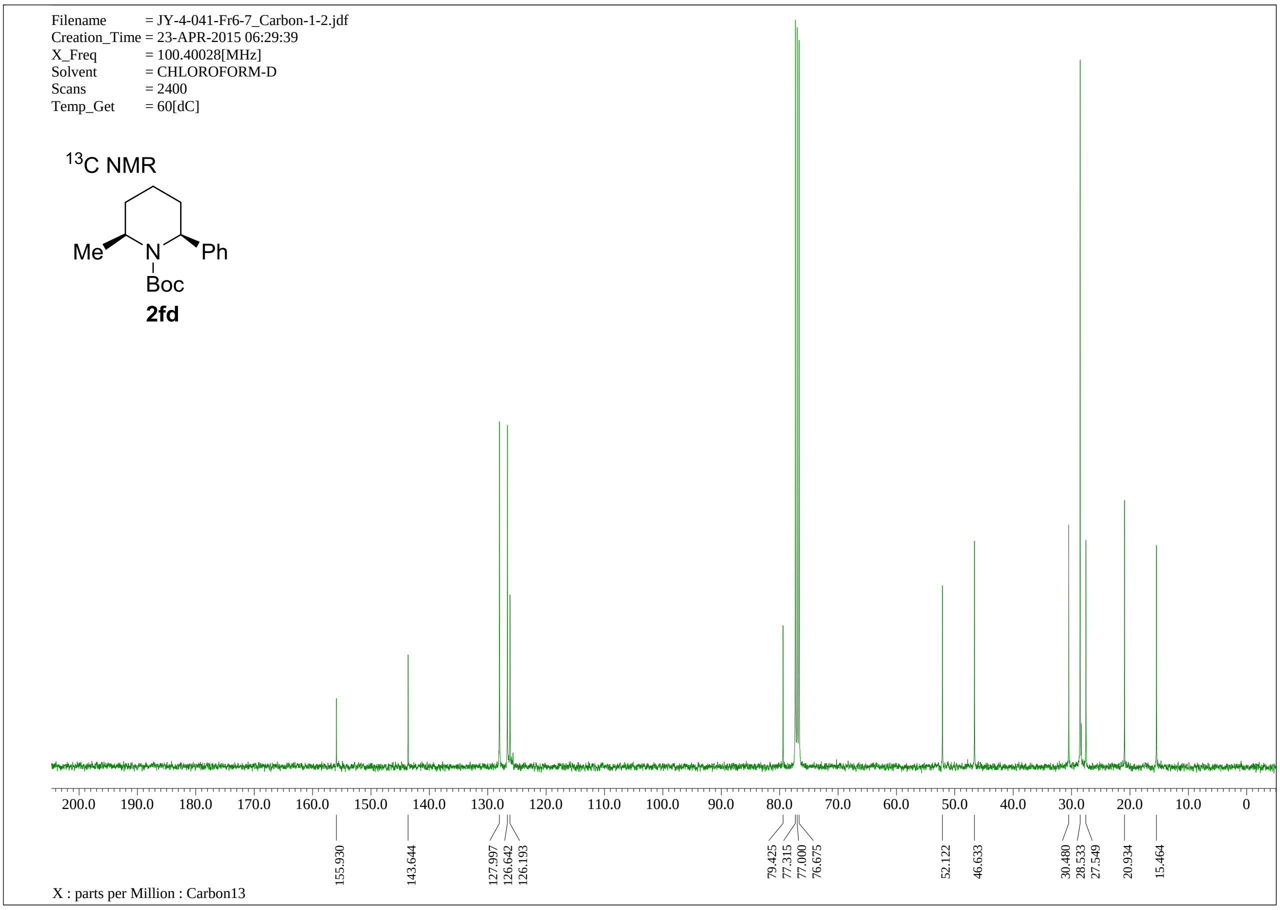
Filename = JY-4-041-Fr6-7_Carbon-1-2.jdf
Creation_Time = 23-APR-2015 06:29:39
X_Freq = 100.40028[MHz]
Solvent = CHLOROFORM-D
Scans = 2400
Temp_Get = 60[dC]
13C NMR
Me
N
Ph
Boc
2fd
155.930
143.644
127.997
126.642
126.193
79.425
77.315
77.000
76.675
52.122
46.633
30.480
28.533
27.549
20.934
15.464
X : parts per Million : Carbon13

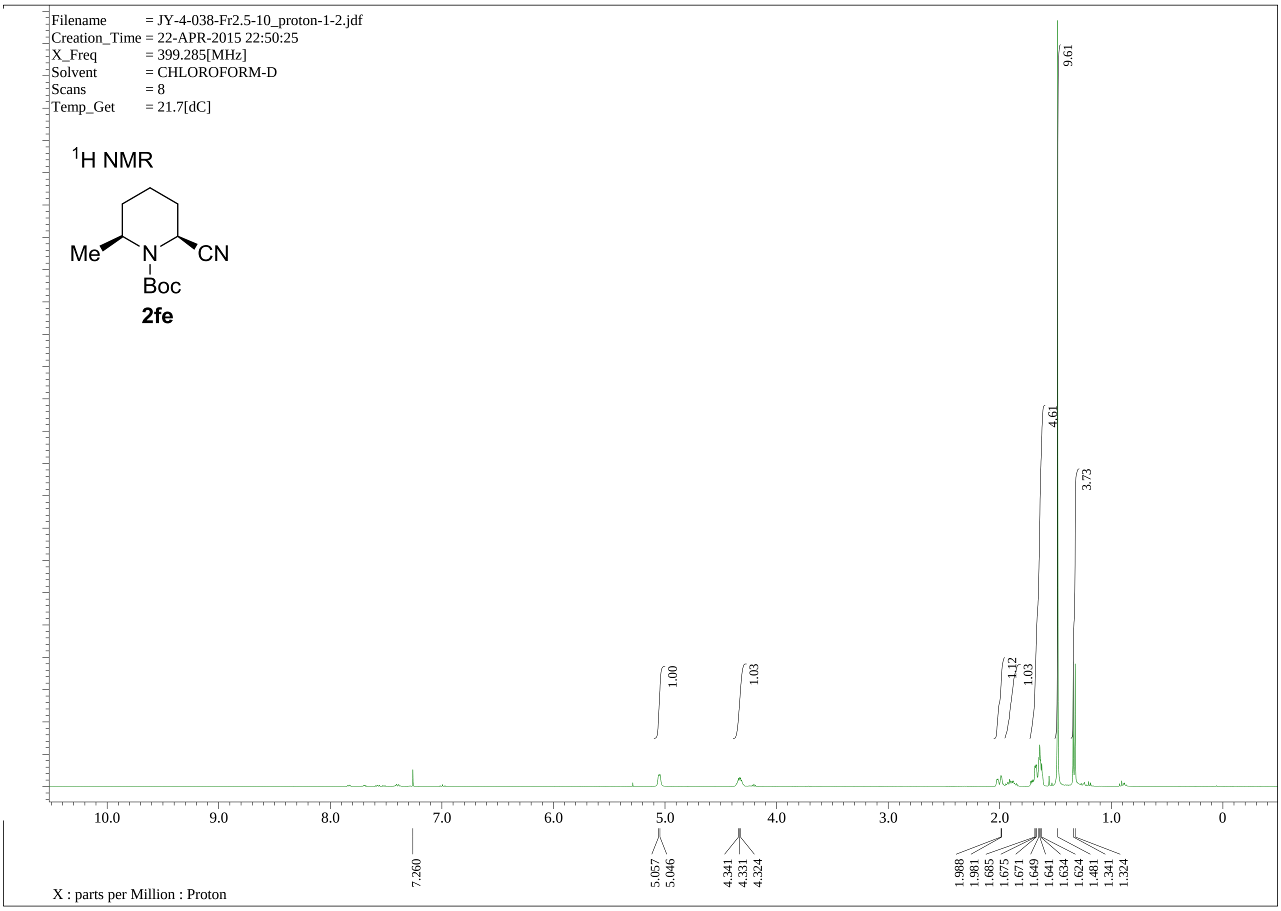

Filename = JY-4-038-Fr2.5-10_proton-1-2.jdf
Creation_Time = 22-APR-2015 22:50:25
X_Freq = 399.285[MHz]
Solvent = CHLOROFORM-D
Scans = 8
Temp_Get = 21.7[dC]
1H NMR
Me
N
CN
Boc
2fe
9.61
4.61
3.73
1.00
1.03
1.12
1.03
10.0 9.0 8.0 7.0 6.0 5.0 4.0 3.0 2.0 1.0 0
7.260
5.057
5.046
4.341
4.331
4.324
1.988
1.981
1.685
1.675
1.671
1.649
1.641
1.634
1.624
1.481
1.341
1.324
X : parts per Million : Proton

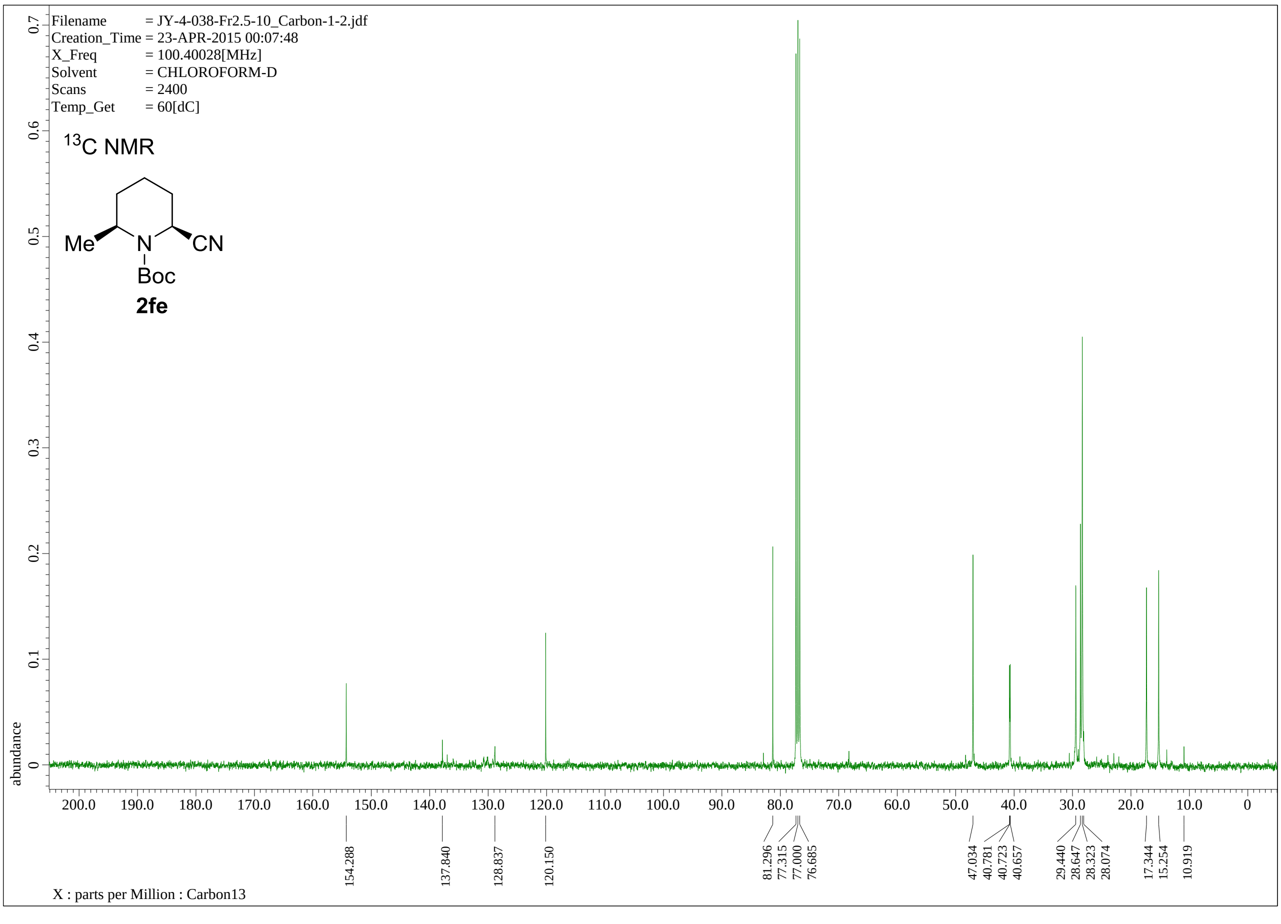

Filename = JY-4-038-Fr2.5-10_Carbon-1-2.jdf
Creation_Time = 23-APR-2015 00:07:48
X_Freq = 100.40028[MHz]
Solvent = CHLOROFORM-D
Scans = 2400
Temp_Get = 60[dC]
13C NMR
Me
N
CN
Boc
2fe
abundance
0 0.1 0.2 0.3 0.4 0.5 0.6 0.7
200.0 190.0 180.0 170.0 160.0 150.0 140.0 130.0 120.0 110.0 100.0 90.0 80.0 70.0 60.0 50.0 40.0 30.0 20.0 10.0 0
154.288
137.840
128.837
120.150
81.296
77.315
77.000
76.685
47.034
40.781
40.723
40.657
29.440
28.647
28.323
28.074
17.344
15.254
10.919
X : parts per Million : Carbon13

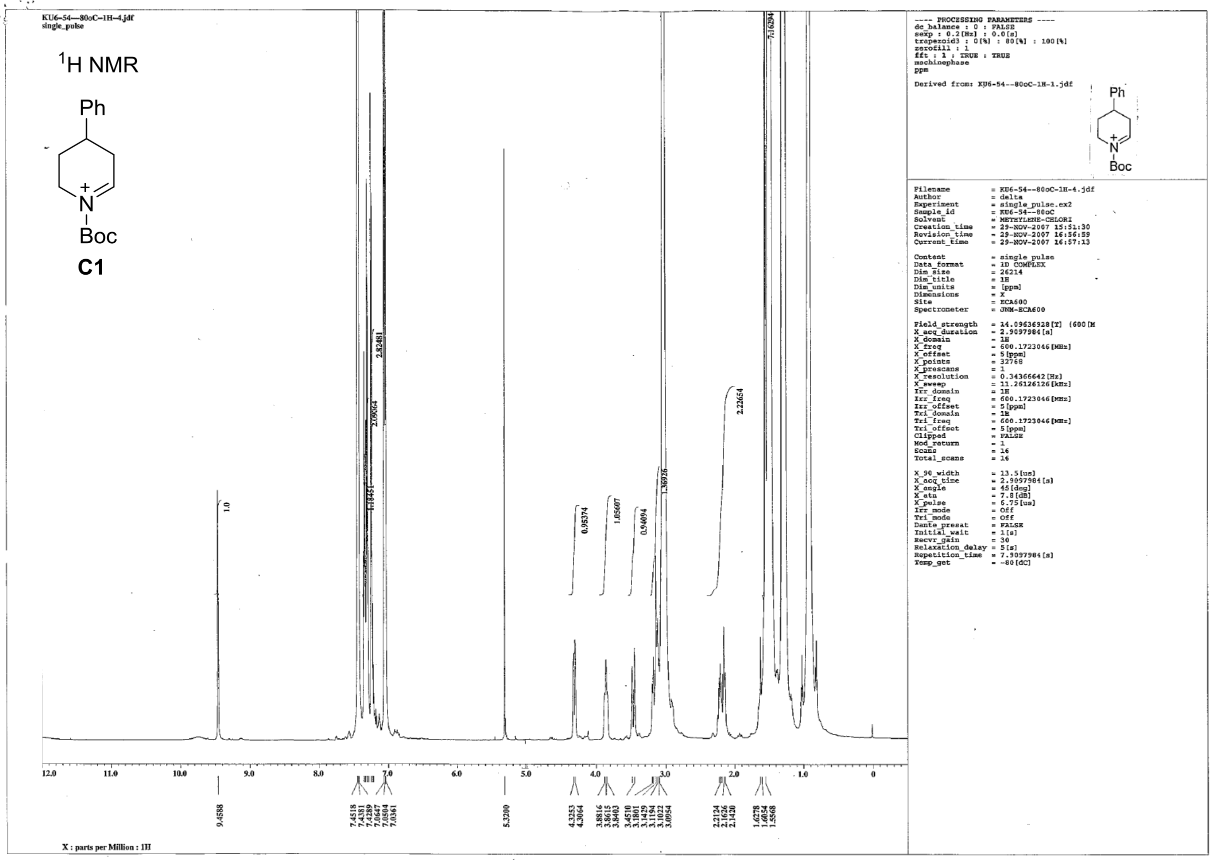
KU6-54--80oC-1H-4.jdf
single_pulse
1H NMR
Ph
N
Boc
C1
X : parts per Million : 1H

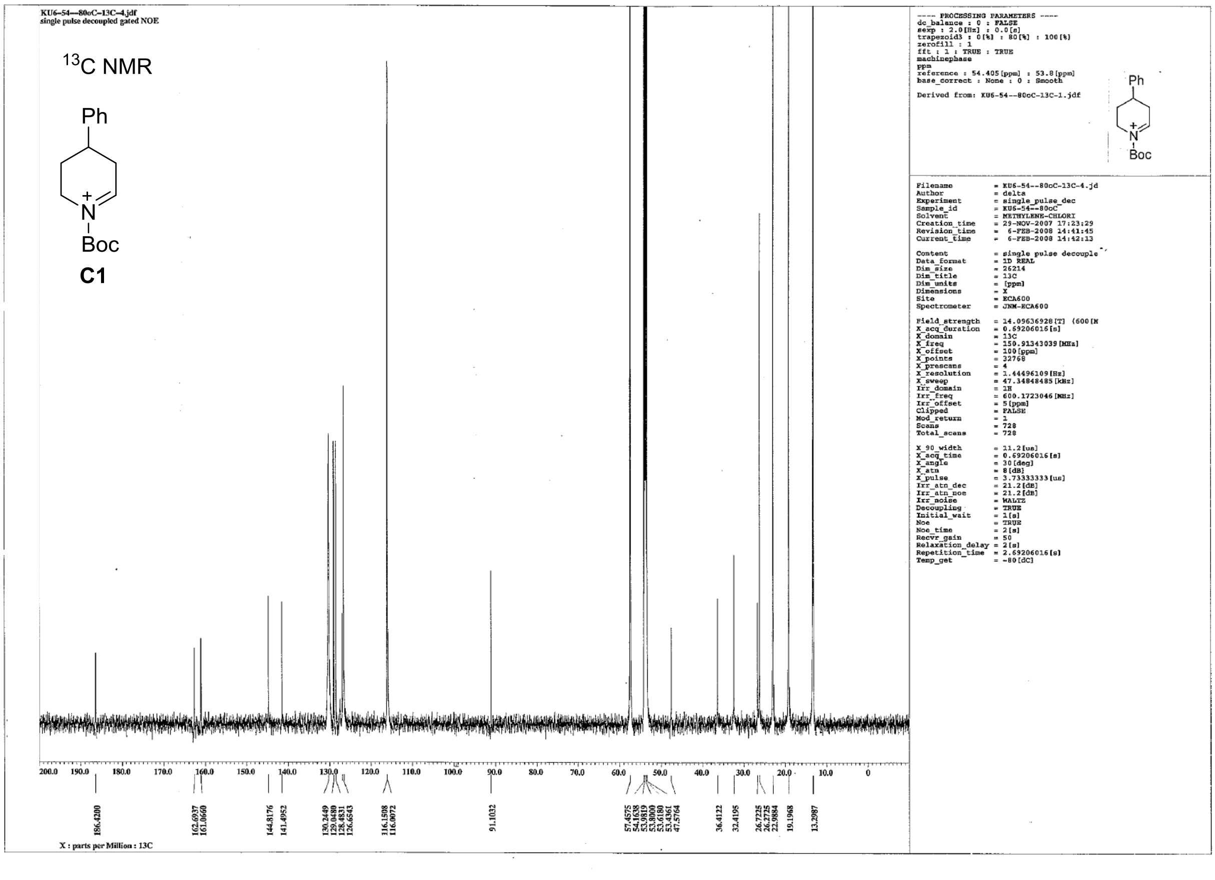
KU6-54--80oC-13C-4.jdf
single pulse decoupled gated NOE
13C NMR
Ph
N
Boc
C1
X : parts per Million : 13C

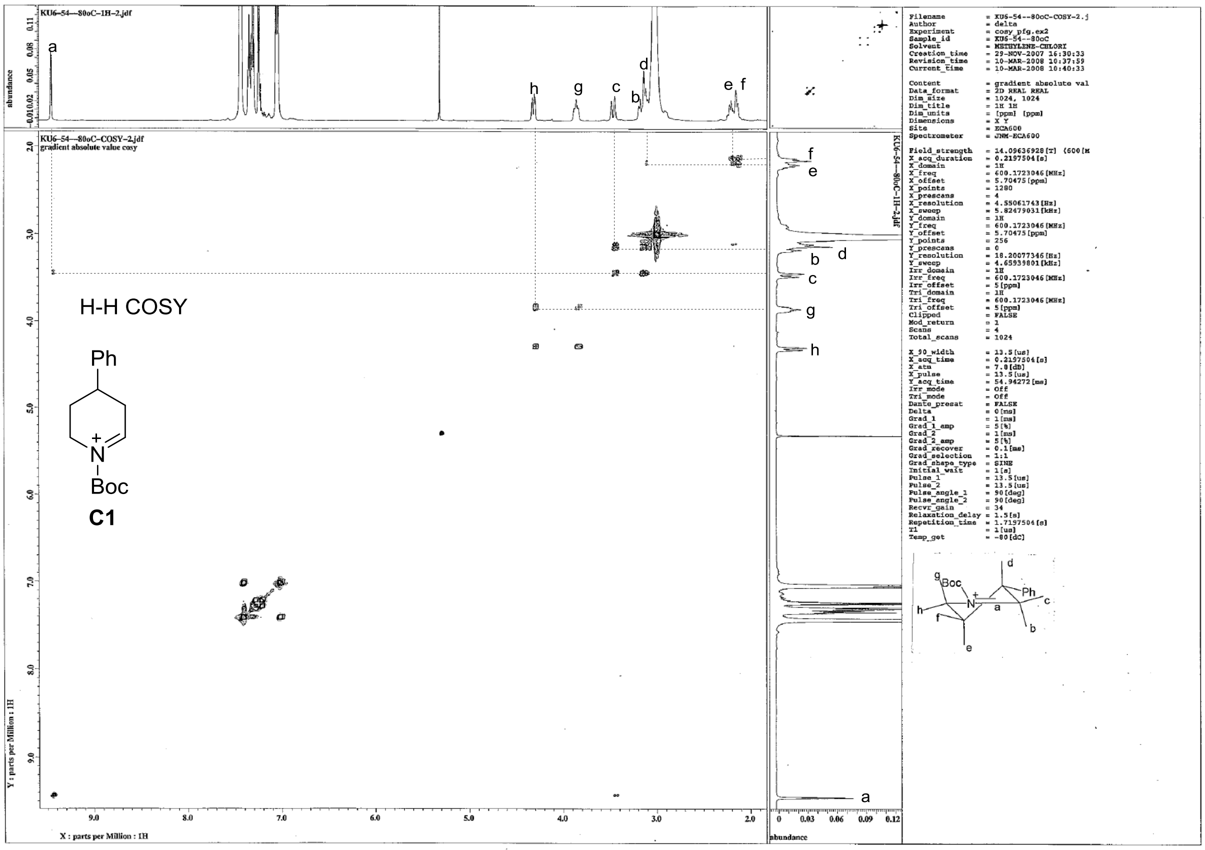

ROE of **C1**

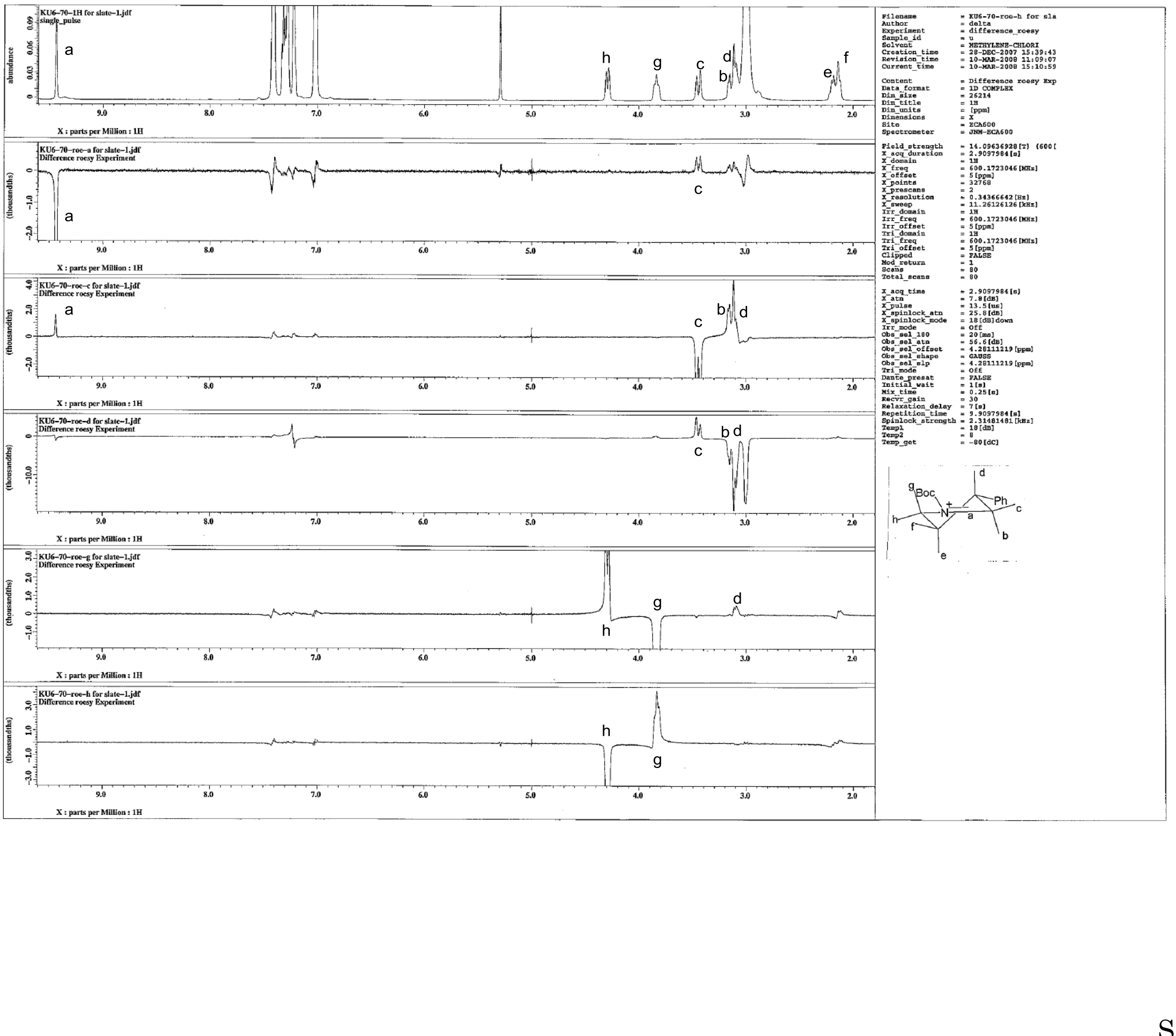

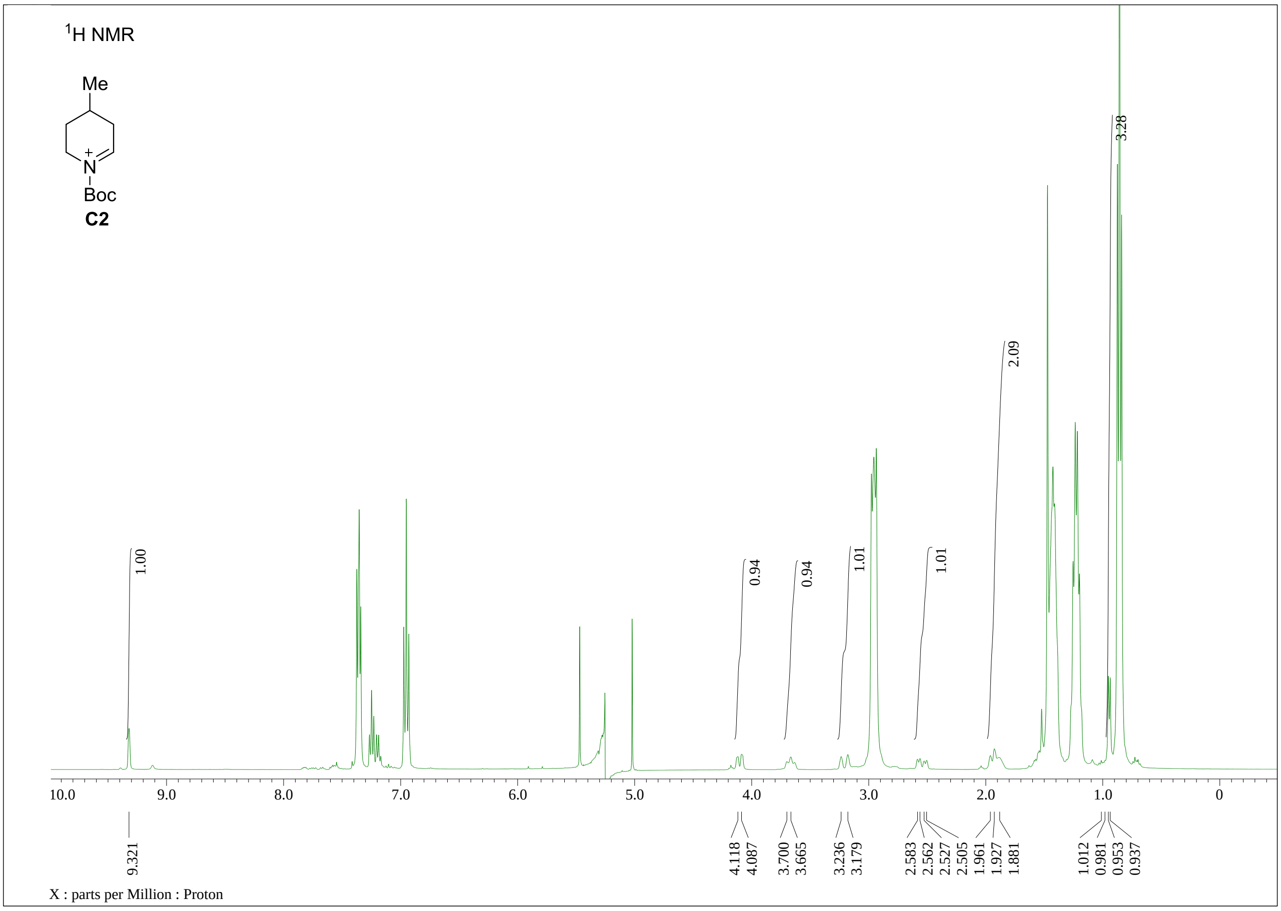

1H NMR
Me
+
N
Boc
C2
1.00
0.94
0.94
1.01
1.01
2.09
3.28
10.0
9.0
8.0
7.0
6.0
5.0
4.0
3.0
2.0
1.0
0
9.321
4.118
4.087
3.700
3.665
3.236
3.179
2.583
2.562
2.527
2.505
1.961
1.927
1.881
1.012
0.981
0.953
0.937
X : parts per Million : Proton

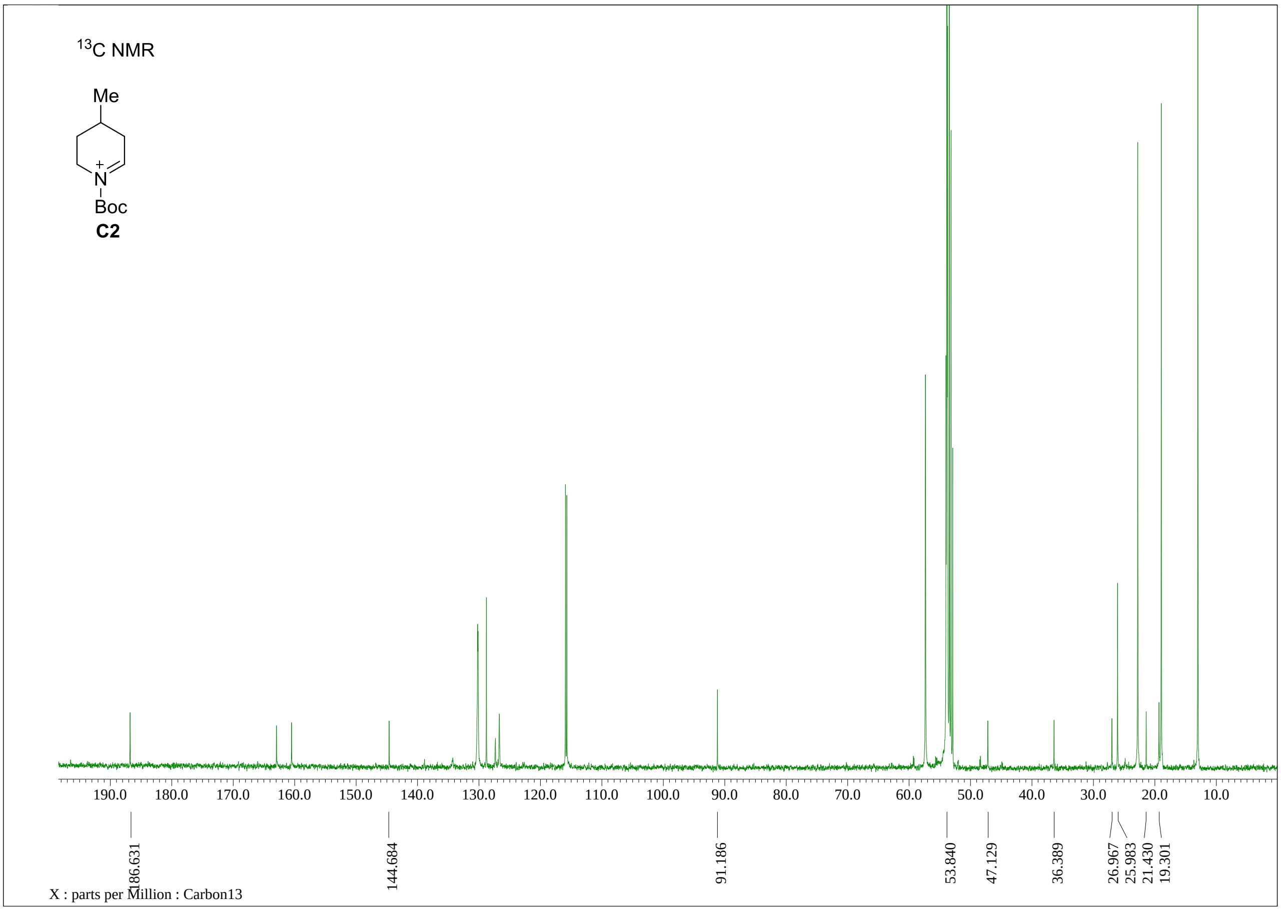

13C NMR
Me
+
N
Boc
C2
190.0
180.0
170.0
160.0
150.0
140.0
130.0
120.0
110.0
100.0
90.0
80.0
70.0
60.0
50.0
40.0
30.0
20.0
10.0
186.631
144.684
91.186
53.840
47.129
36.389
26.967
25.983
21.430
19.301
X : parts per Million : Carbon13

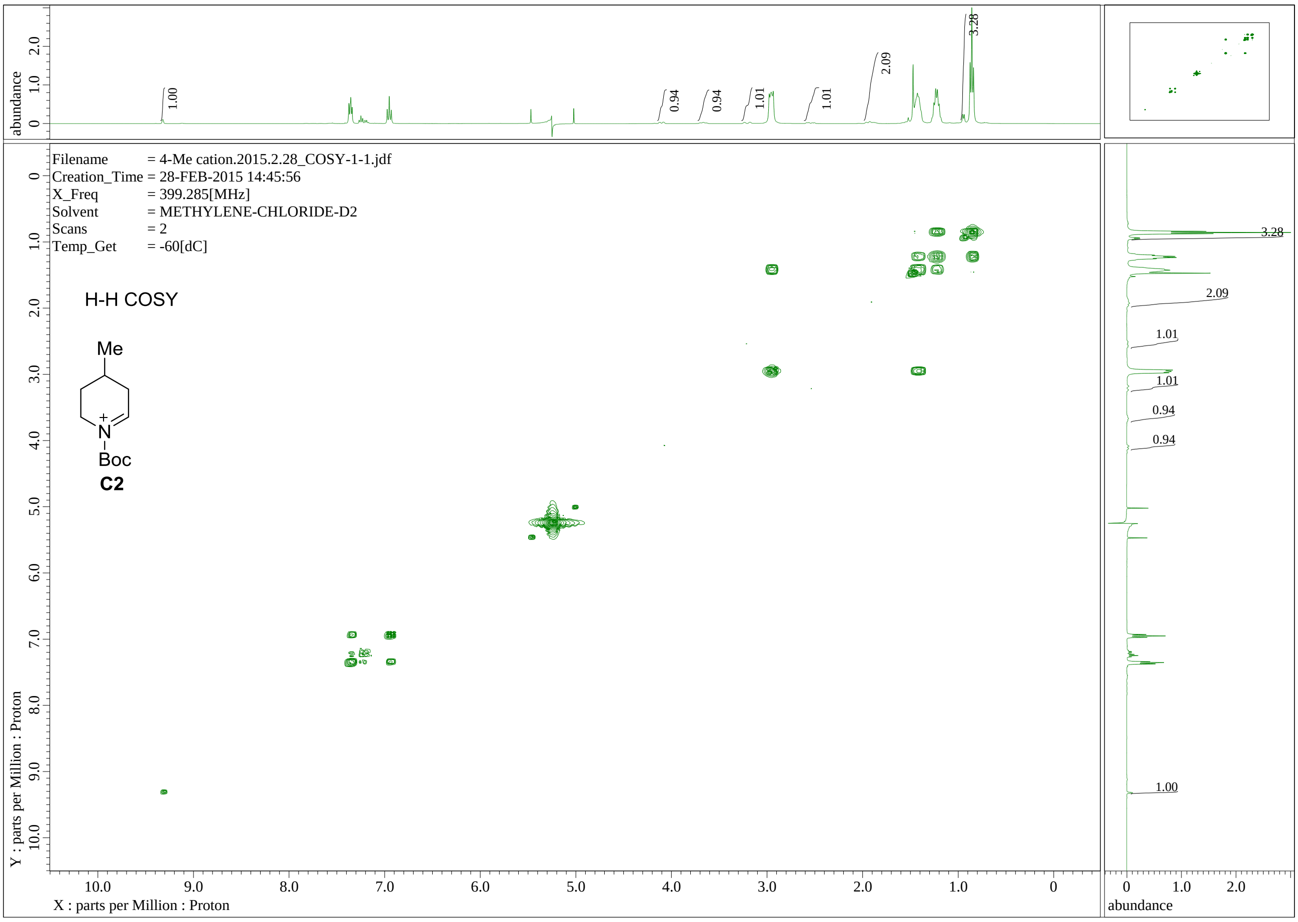
Filename = 4-Me cation.2015.2.28_COSY-1-1.jdf
Creation_Time = 28-FEB-2015 14:45:56
X_Freq = 399.285[MHz]
Solvent = METHYLENE-CHLORIDE-D2
Scans = 2
Temp_Get = -60[dC]
H-H COSY
Me
N
Boc
C2
X : parts per Million : Proton
Y : parts per Million : Proton
abundance

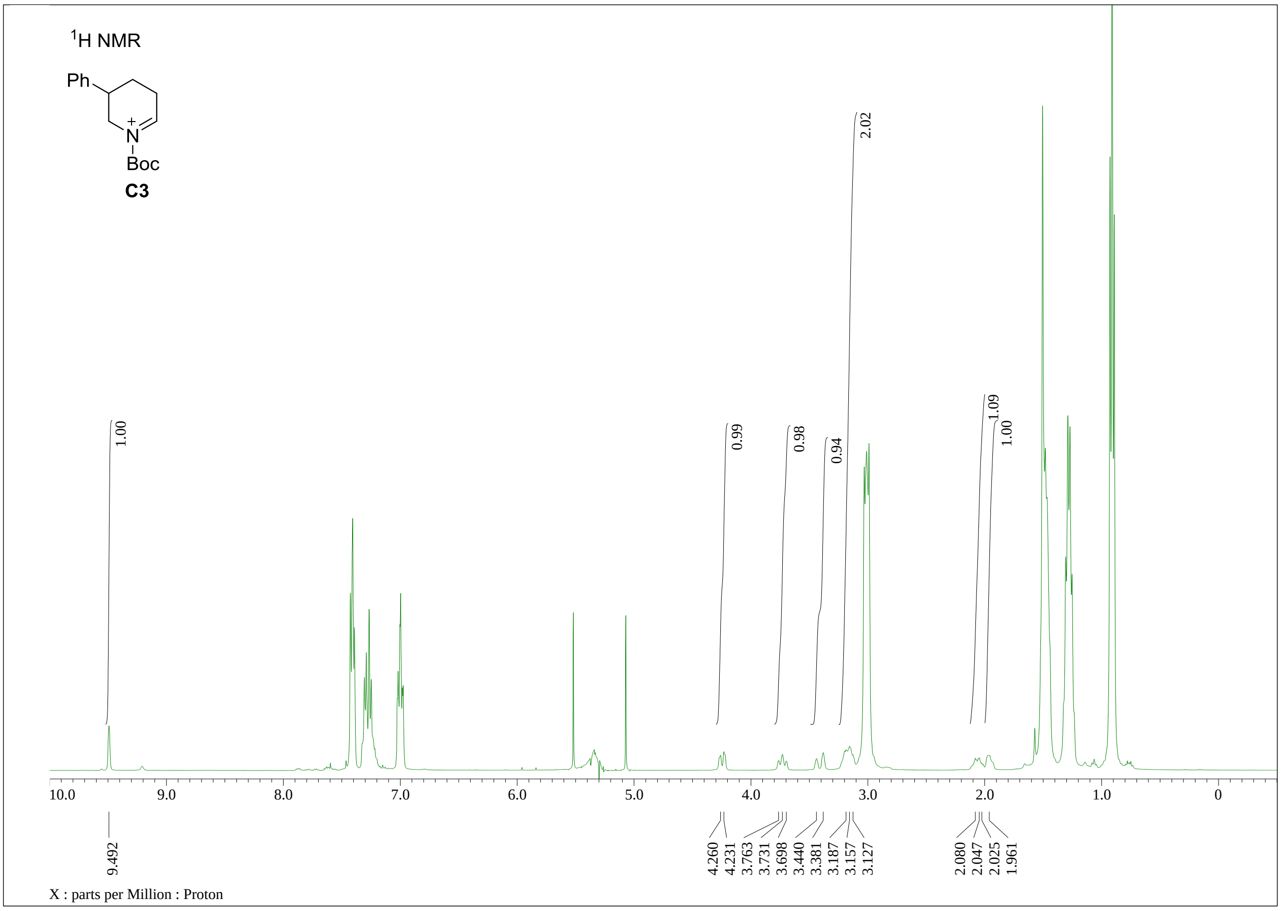

1H NMR
Ph
N
Boc
C3
1.00
0.99
0.98
0.94
2.02
1.09
1.00
10.0
9.0
8.0
7.0
6.0
5.0
4.0
3.0
2.0
1.0
0
9.492
4.260
4.231
3.763
3.731
3.698
3.440
3.381
3.187
3.157
3.127
2.080
2.047
2.025
1.961
X : parts per Million : Proton

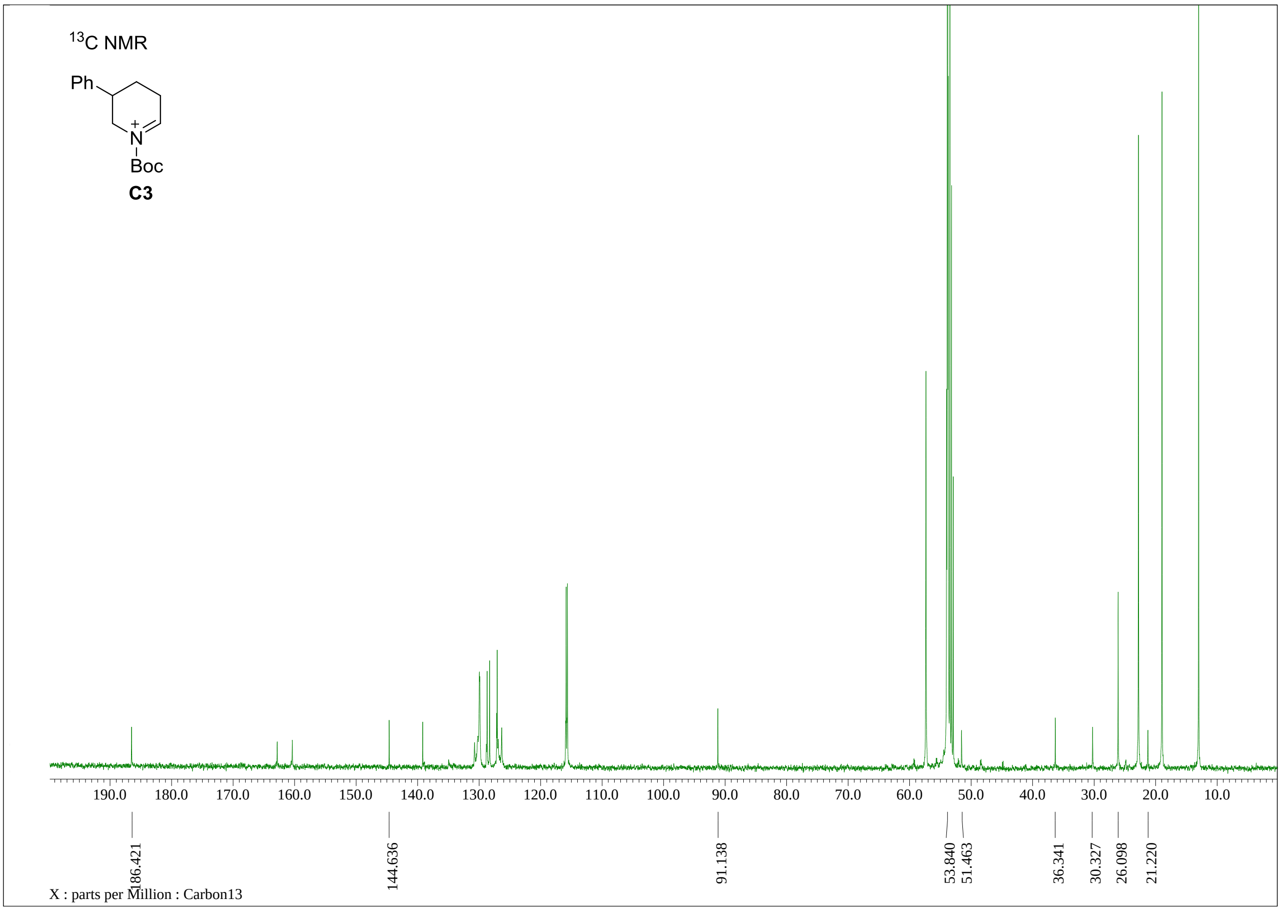

13C NMR
Ph
N
Boc
C3
190.0
180.0
170.0
160.0
150.0
140.0
130.0
120.0
110.0
100.0
90.0
80.0
70.0
60.0
50.0
40.0
30.0
20.0
10.0
186.421
144.636
91.138
53.840
51.463
36.341
30.327
26.098
21.220
X : parts per Million : Carbon13

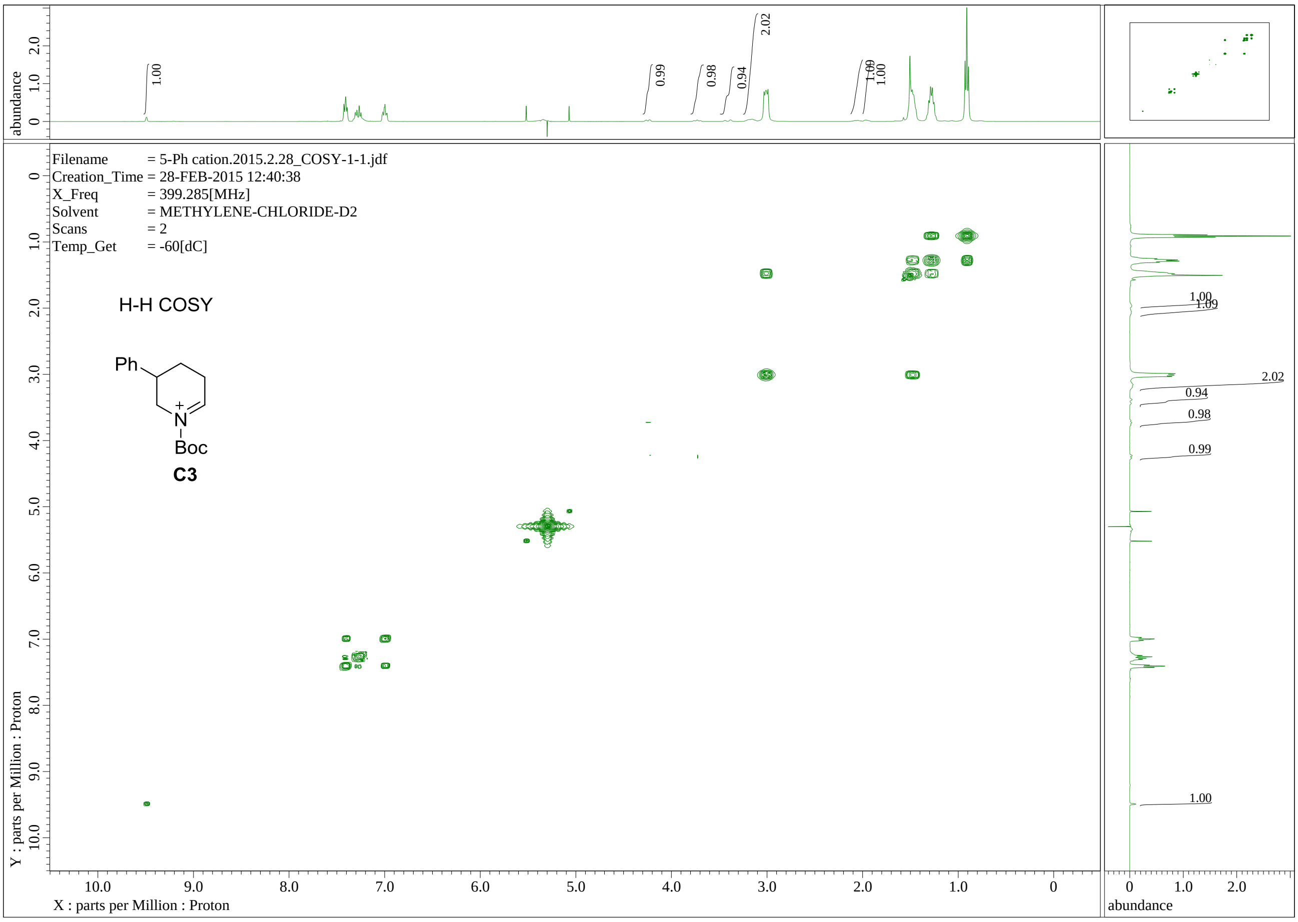
Filename = 5-Ph cation.2015.2.28_COSY-1-1.jdf
Creation_Time = 28-FEB-2015 12:40:38
X_Freq = 399.285[MHz]
Solvent = METHYLENE-CHLORIDE-D2
Scans = 2
Temp_Get = -60[dC]
H-H COSY
Ph
N
Boc
C3
X : parts per Million : Proton
Y : parts per Million : Proton
abundance

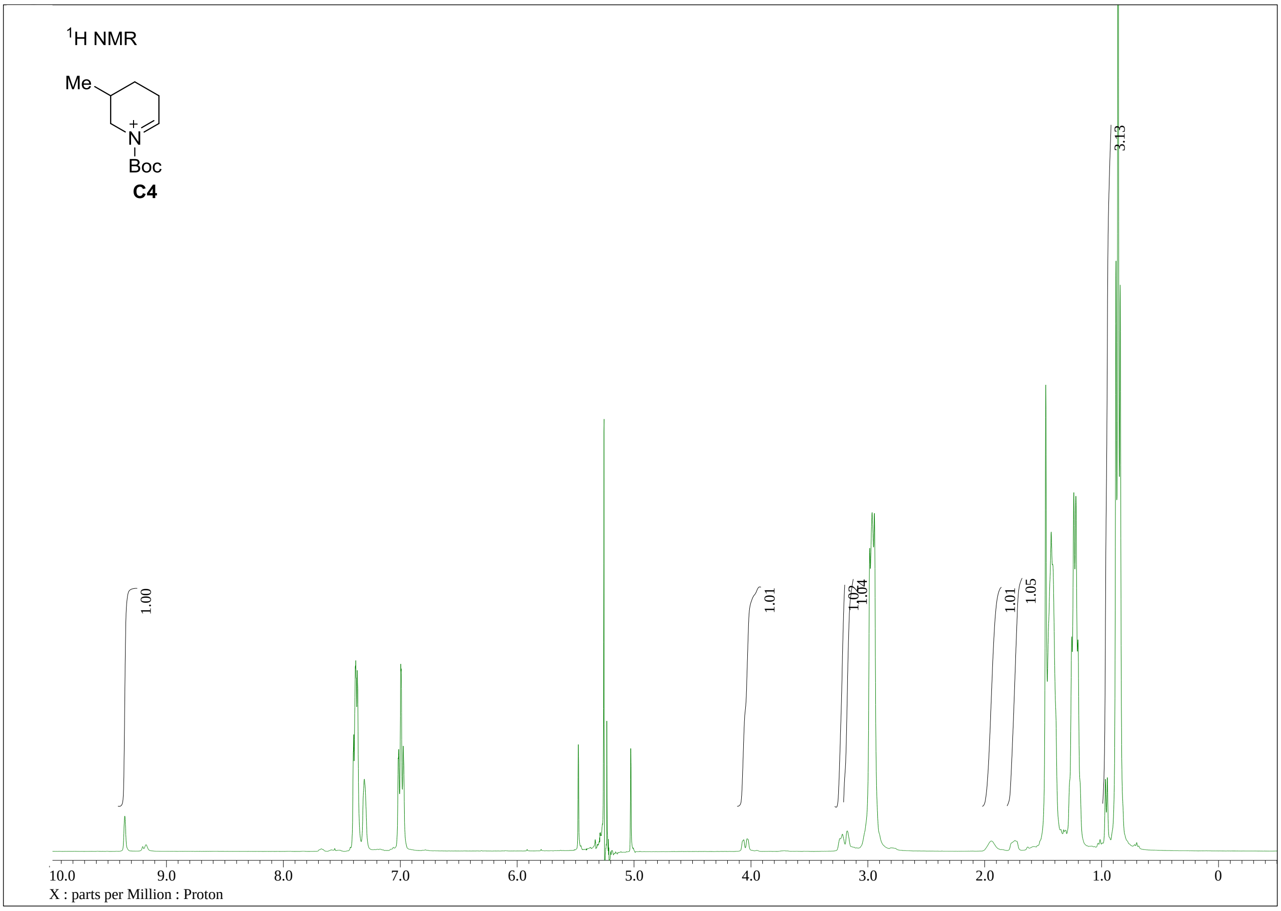
1H NMR
Me
N
Boc
C4
1.00
1.01
1.02
1.04
1.01
1.05
3.13
10.0
9.0
8.0
7.0
6.0
5.0
4.0
3.0
2.0
1.0
0
X : parts per Million : Proton

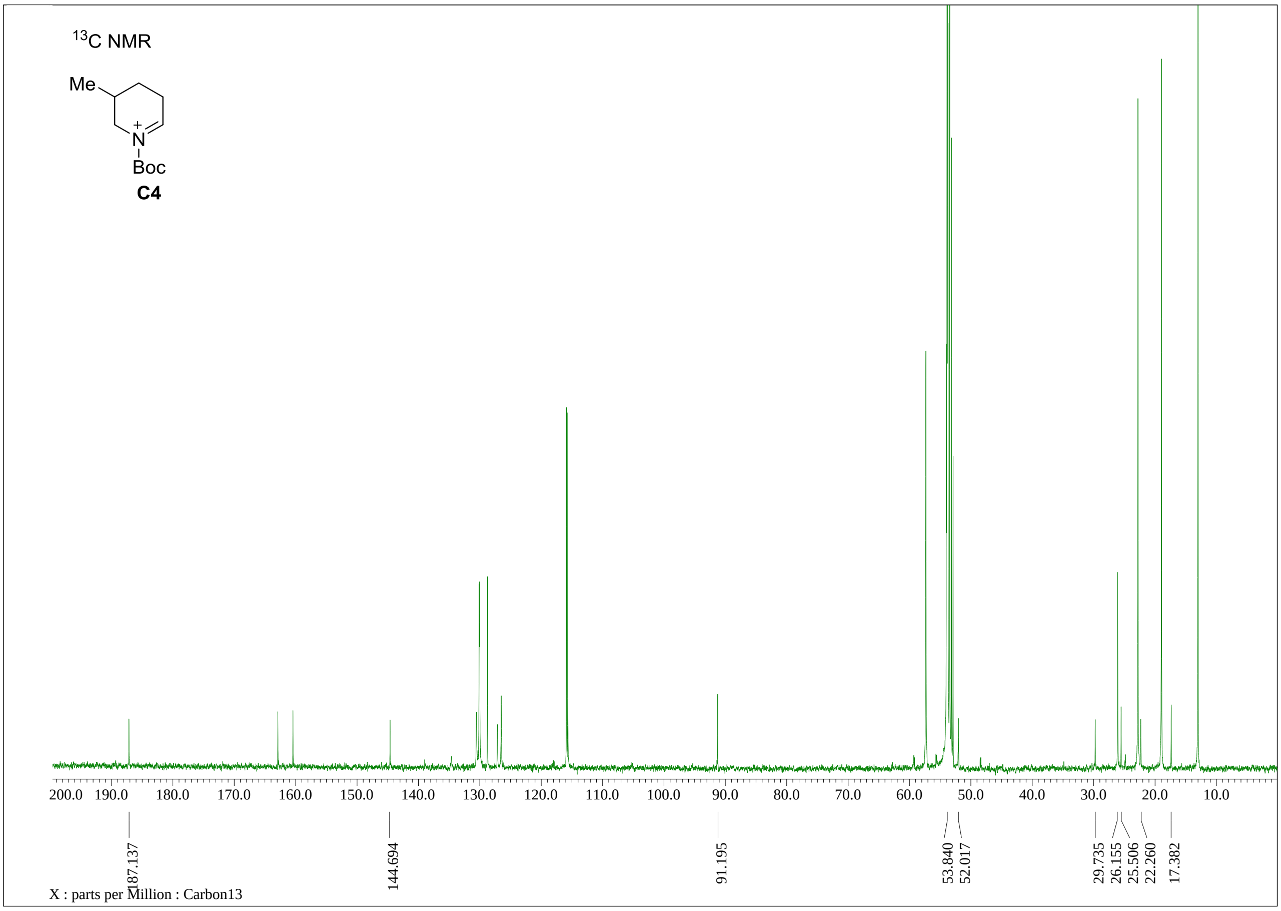
13C NMR
Me
N
Boc
C4
200.0
190.0
180.0
170.0
160.0
150.0
140.0
130.0
120.0
110.0
100.0
90.0
80.0
70.0
60.0
50.0
40.0
30.0
20.0
10.0
187.137
144.694
91.195
53.840
52.017
29.735
26.155
25.506
22.260
17.382
X : parts per Million : Carbon13

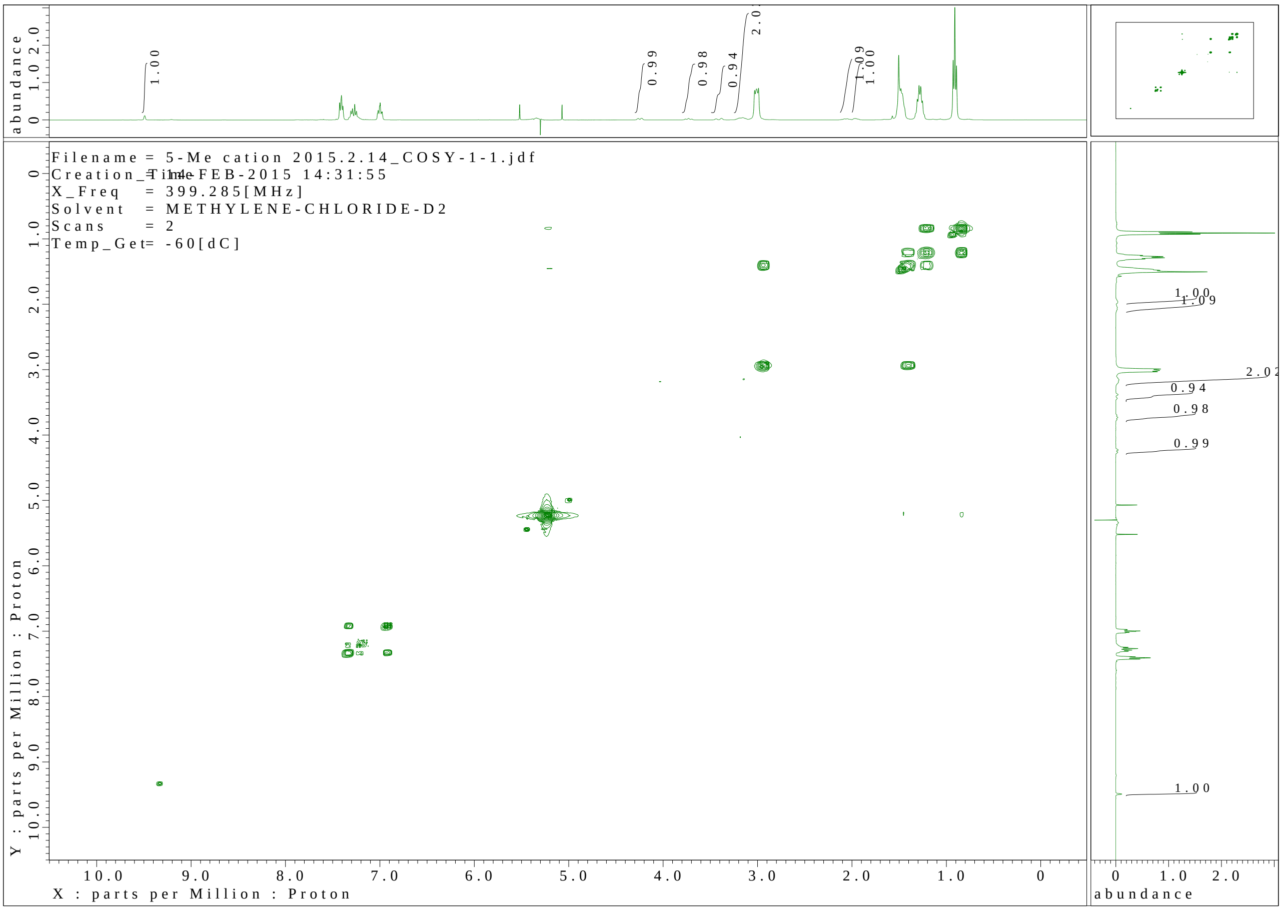
Filename = 5-Me cation 2015.2.14_COSY-1-1.jdf
X_Freq = 399.285[MHz]
Solvent = METHYLENE-CHLORIDE-D2
Scans = 2
Temp_Get= -60[dC]
X : parts per Million : Proton
Y : parts per Million : Proton
abundance

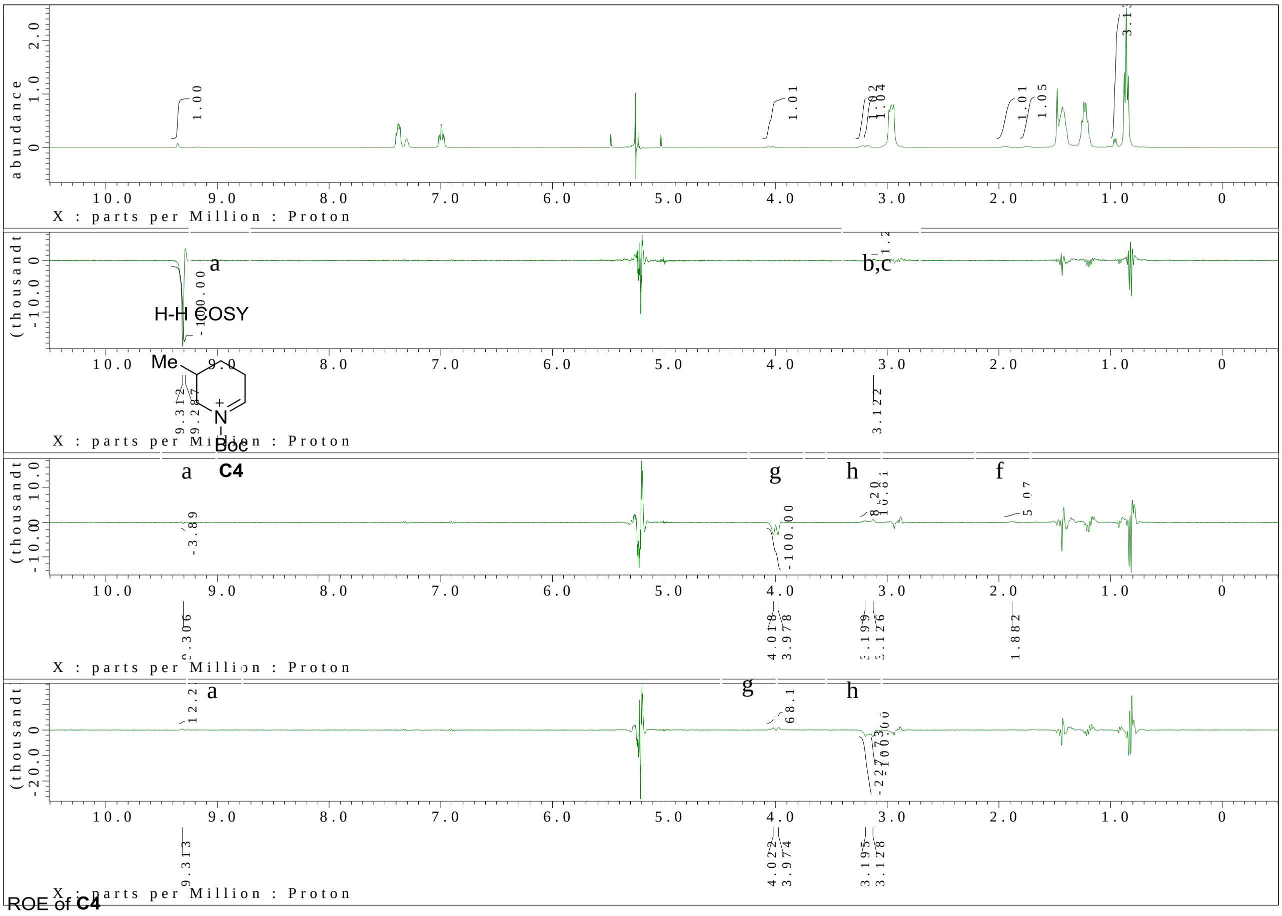
X : parts per Million : Proton
abundance
(thousandt
a
b,c
H-H COSY
Me
N
Boc
C4
g
h
f
ROE of C4

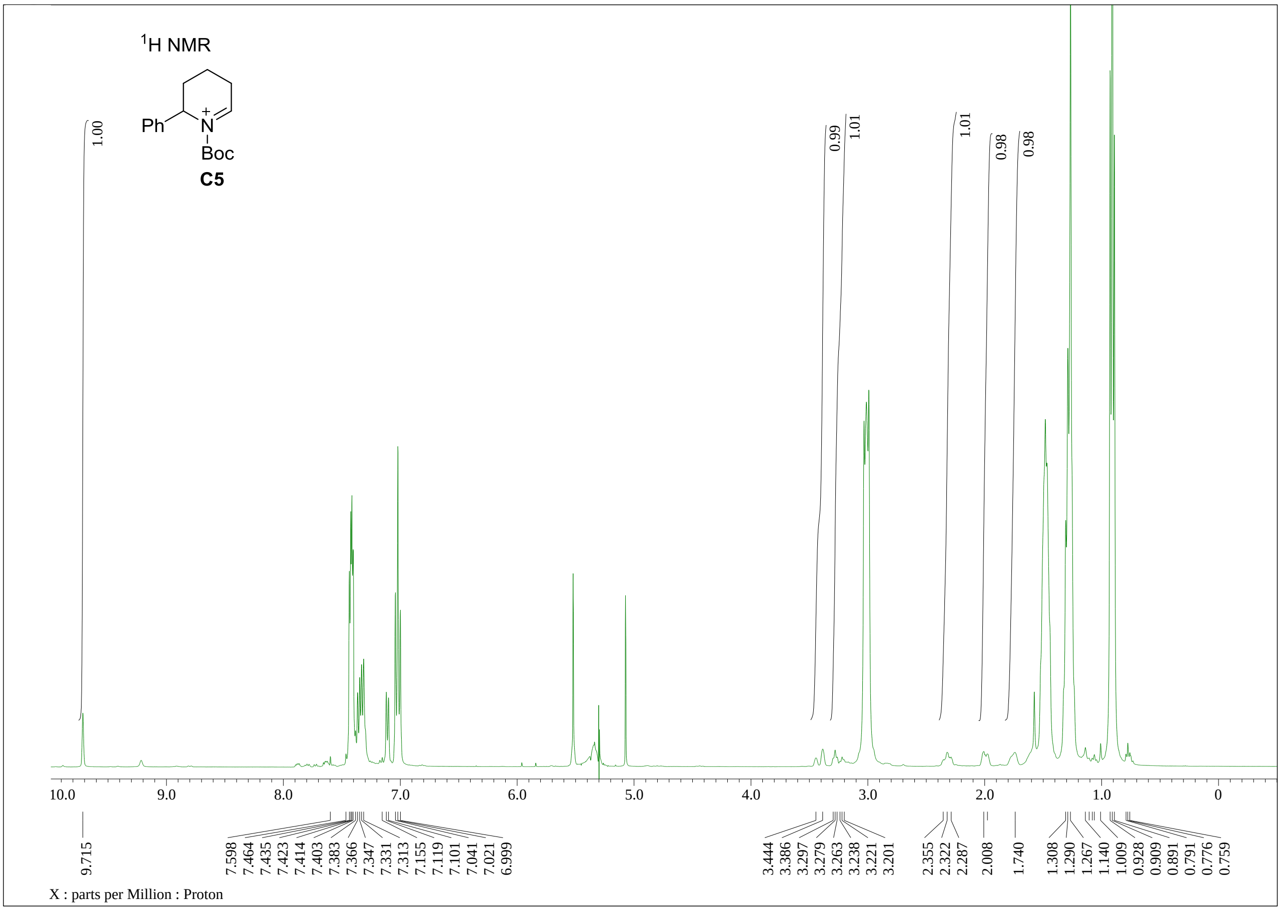

1H NMR
Ph
N
Boc
C5
1.00
0.99
1.01
1.01
0.98
0.98
10.0
9.0
8.0
7.0
6.0
5.0
4.0
3.0
2.0
1.0
0
9.715
7.598
7.464
7.435
7.423
7.414
7.403
7.383
7.366
7.347
7.331
7.313
7.155
7.119
7.101
7.041
7.021
6.999
3.444
3.386
3.297
3.279
3.263
3.238
3.221
3.201
2.355
2.322
2.287
2.008
1.740
1.308
1.290
1.267
1.140
1.009
0.928
0.909
0.891
0.791
0.776
0.759
X : parts per Million : Proton

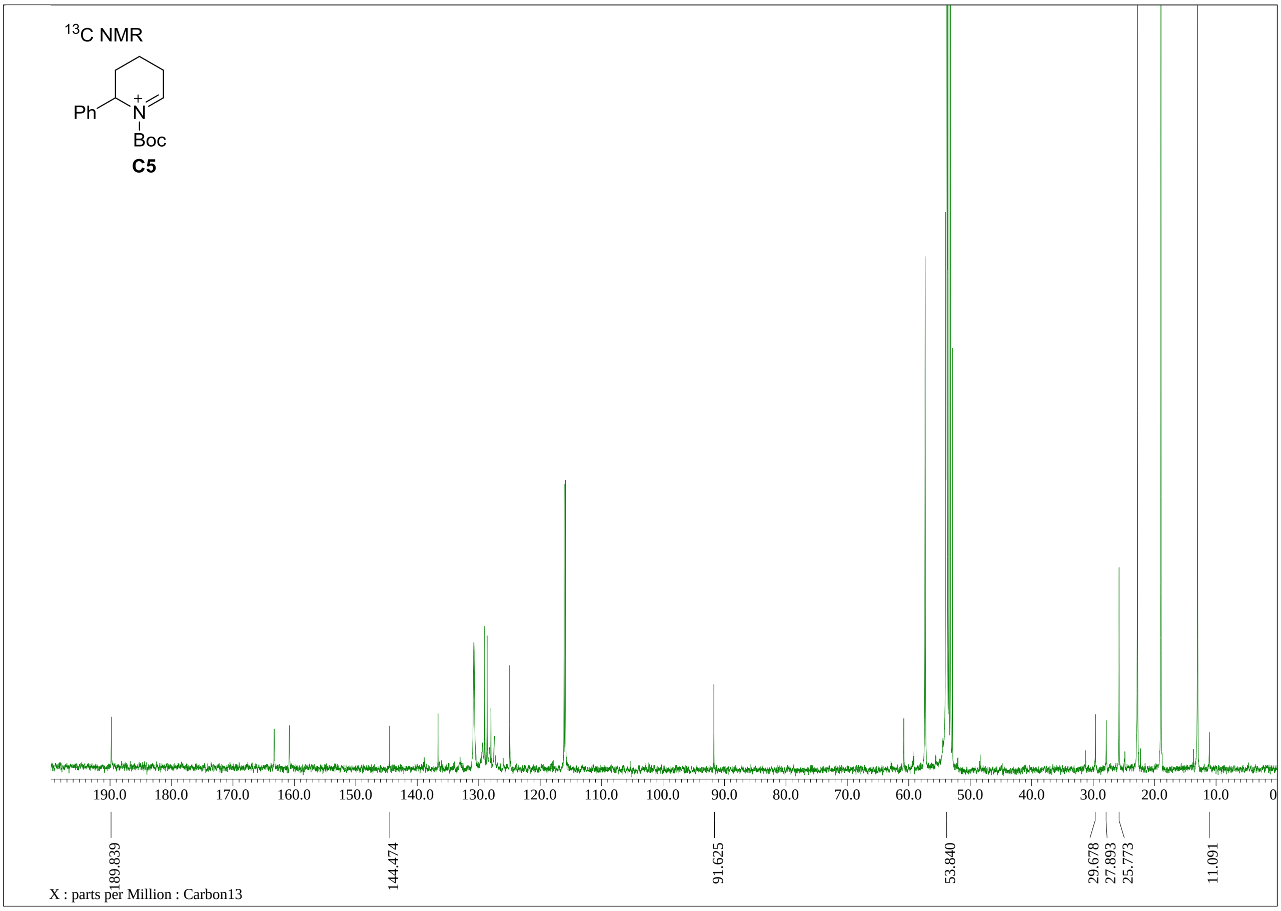

13C NMR
Ph
N
Boc
C5
190.0
180.0
170.0
160.0
150.0
140.0
130.0
120.0
110.0
100.0
90.0
80.0
70.0
60.0
50.0
40.0
30.0
20.0
10.0
0
189.839
144.474
91.625
53.840
29.678
27.893
25.773
11.091
X : parts per Million : Carbon13

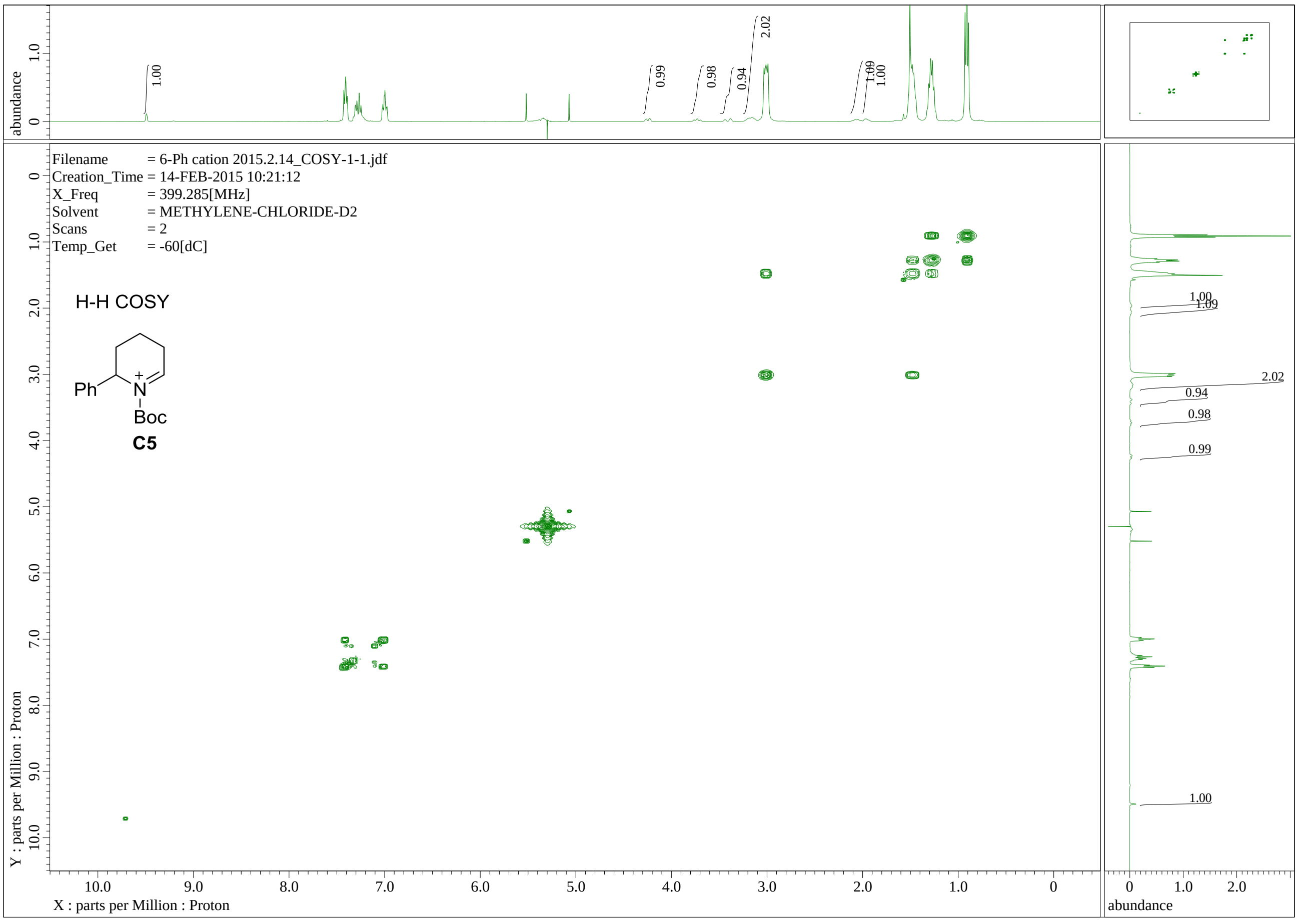
Filename = 6-Ph cation 2015.2.14_COSY-1-1.jdf
Creation_Time = 14-FEB-2015 10:21:12
X_Freq = 399.285[MHz]
Solvent = METHYLENE-CHLORIDE-D2
Scans = 2
Temp_Get = -60[dC]
H-H COSY
Ph
N
Boc
C5
X : parts per Million : Proton
Y : parts per Million : Proton
abundance

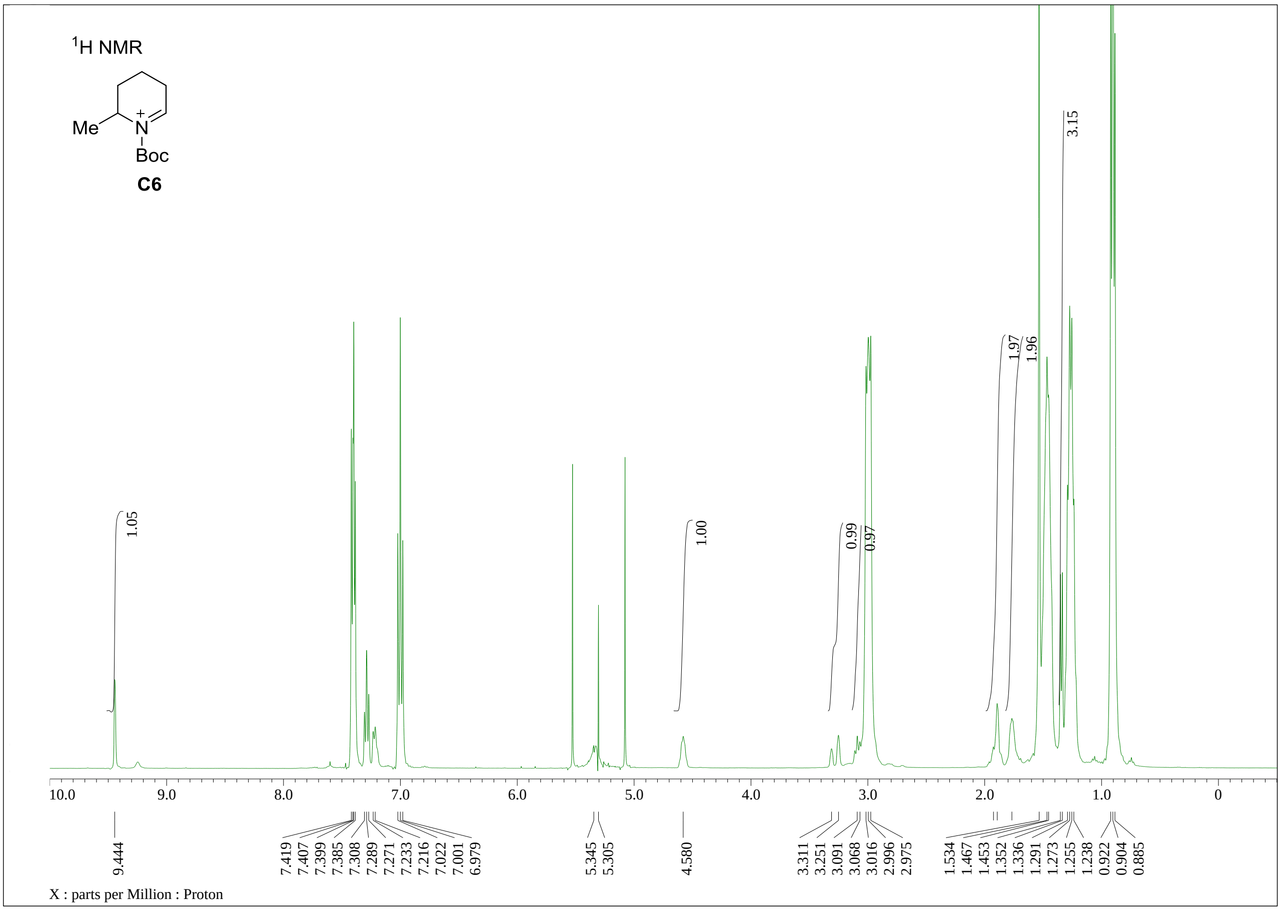

^{1}H NMR
Me
N
Boc
C6
1.05
1.00
0.99
0.97
1.97
1.96
3.15
10.0 9.0 8.0 7.0 6.0 5.0 4.0 3.0 2.0 1.0 0
9.444
7.419 7.407 7.399 7.385 7.308 7.289 7.271 7.233 7.216 7.022 7.001 6.979
5.345 5.305
4.580
3.311 3.251 3.091 3.068 3.016 2.996 2.975
1.534 1.467 1.453 1.352 1.336 1.291 1.273 1.255 1.238
0.922 0.904 0.885
X : parts per Million : Proton

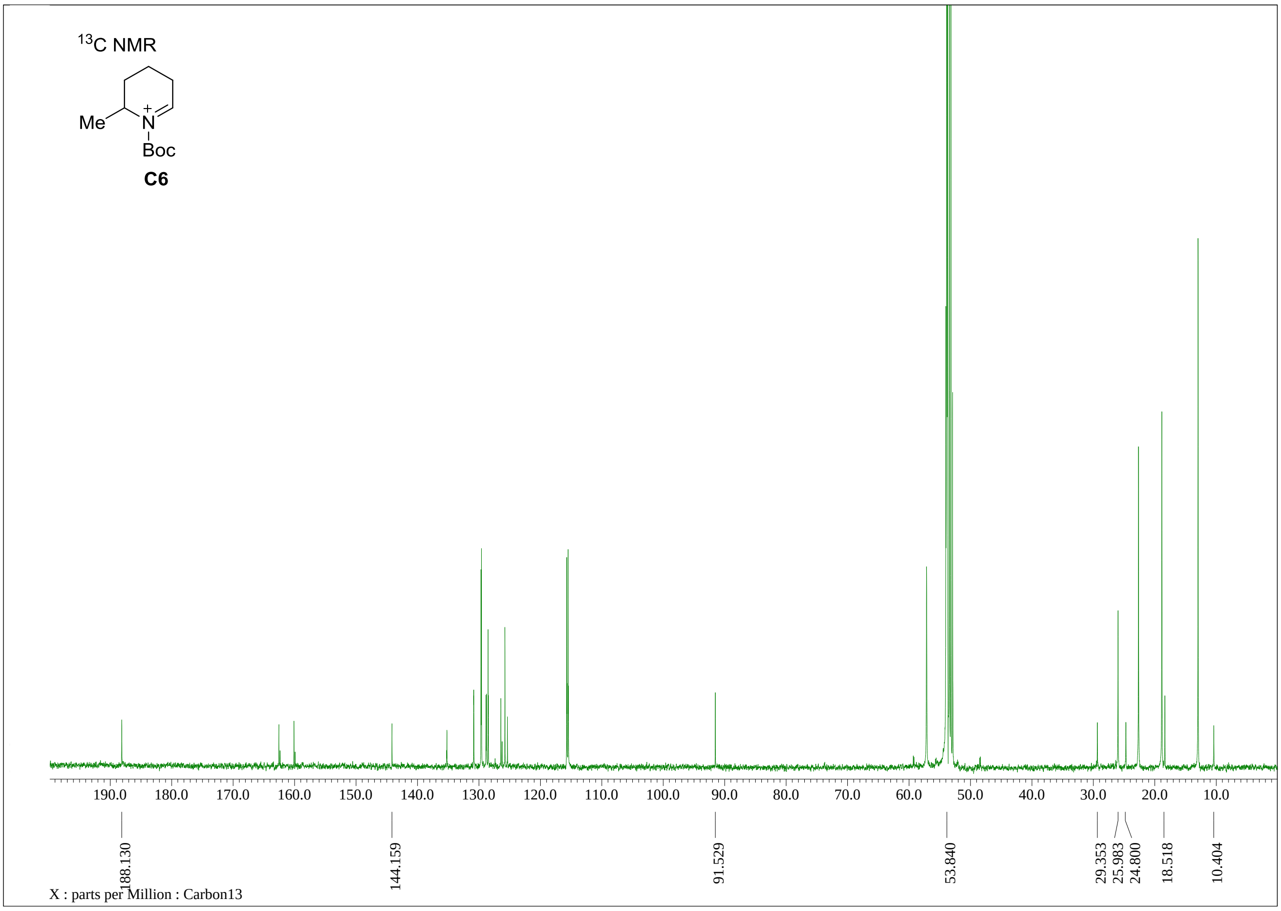

^{13}C NMR
Me
N
Boc
C6
190.0 180.0 170.0 160.0 150.0 140.0 130.0 120.0 110.0 100.0 90.0 80.0 70.0 60.0 50.0 40.0 30.0 20.0 10.0
188.130
144.159
91.529
53.840
29.353 25.983 24.800
18.518
10.404
X : parts per Million : Carbon13

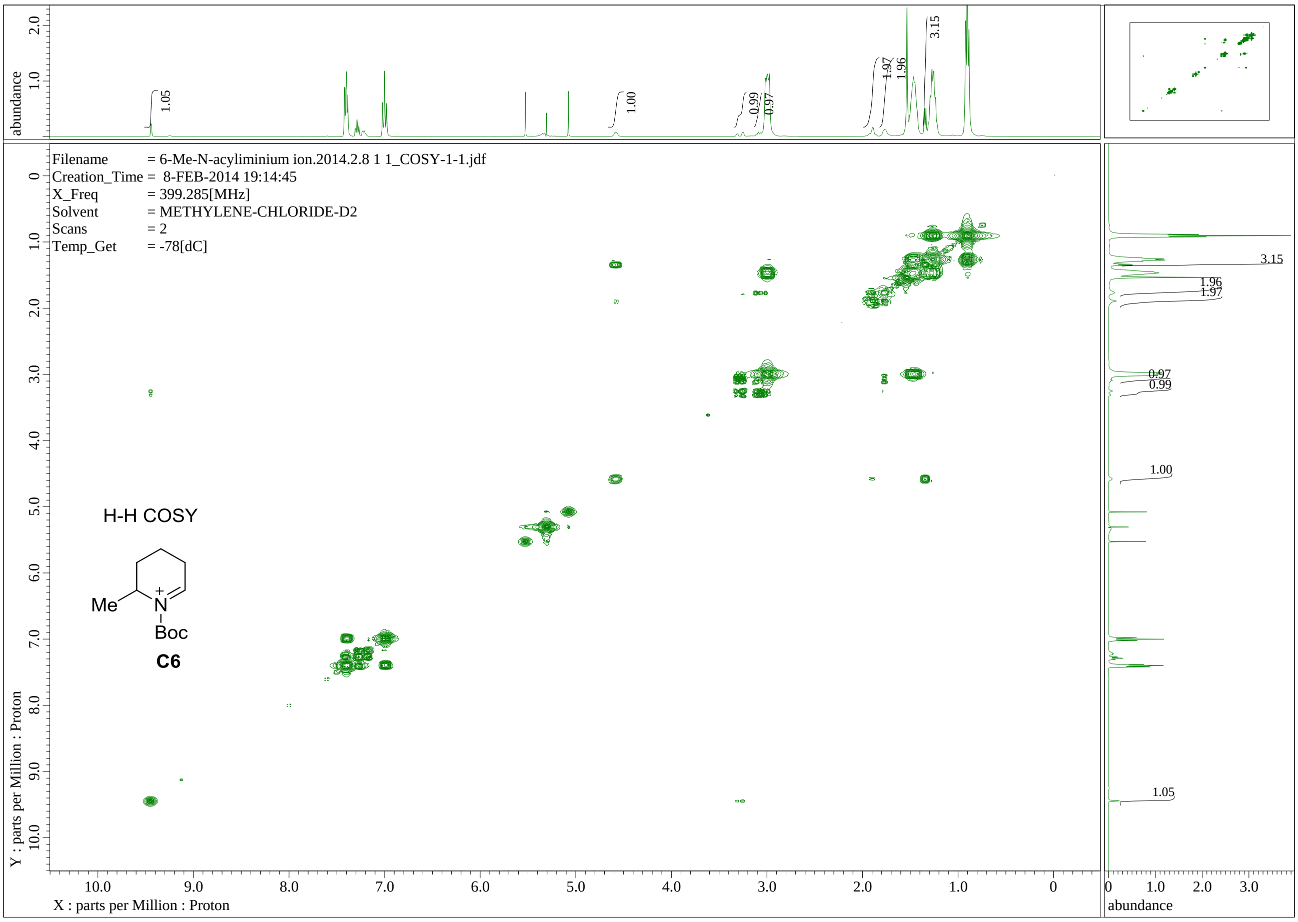

Filename = 6-Me-N-acyliminium ion.2014.2.8 1 1_COSY-1-1.jdf
Creation_Time = 8-FEB-2014 19:14:45
X_Freq = 399.285[MHz]
Solvent = METHYLENE-CHLORIDE-D2
Scans = 2
Temp_Get = -78[dC]
H-H COSY
Me
N
+
Boc
C6
abundance
X : parts per Million : Proton
Y : parts per Million : Proton
1.05
1.00
0.99
0.97
1.97
1.96
3.15

5. References

(1) Leino, R.; Lönnqvist, J. E. *Tetrahedron Lett.* **2004**, *45*, 8489.

(2) David, G.; Oscar, P.; Miguel, Y. *J. Org. Chem.* **2013**, *78*, 3647.

(3) Franklin, A. D.; Joanna M. S. *Tetrahedron Lett.* **1998**, *39*, 5951.

(4) Ethel, C.; Garnier, E. C.; Lanny S.; Liebeskind, L. S. *J. Am. Chem. Soc*. **2008**, *130*, 7449

(5) Dieter R. K.; Sharma, R. R. *J. Org. Chem.* **1996**, *61*, 4180

(6) Matsumoto, K.; Ueoka, K.; Suzuki, S.; Suga, S.; Yoshida, J. *Tetrahedron* **2009**, *65*, 10901.

(7) *SIR2014*, Burla, M. C.; Caliandro, R.; Carrozzini, B.; Cascarano, G. L.; Giacovazzo, C.; Mallamo, M.; Mazzone, A.; Polidori, G. *J. Appl. Crystallogr.* **2015**, *48*, 306.

(8) *SHELX*, Program for the Solution of Crystal Structures (a) Sheldrick, G. M.; University of Göttingen: Göttingen, Germany, 2014. (b) Sheldrick, G.M. *Acta Crystallogr. Sect. A* **2008**, *64*, 112.

(9)Yadokari-XG, Software for Crystal Structure Analyses, K. Wakita (2001); Release of Software (Yadokari-XG 2009) for Crystal Structure Analyses, Kabuto, C.; Akine, S.; Nemoto, T.; Kwon, E. *J. Cryst. Soc. Jpn.* **2009**, *51*, 218.

(10) Farrugia, L. J. *J. Appl. Crystallogr.* **2012**, *45*, 849.

(11) *Gaussian 09, Revision E.01*, Frisch, M. J.; Trucks, G. W.; Schlegel, H. B.; Scuseria, G. E.; Robb, M. A.; Cheeseman, J. R.; Scalmani, G.; Barone, V.; Mennucci, B.; Petersson, G. A.; Nakatsuji, H.; Caricato, M.; Li, X.; Hratchian, H. P.; Izmaylov, A. F.; Bloino, J.; Zheng, G.; Sonnenberg, J. L.; Hada, M.; Ehara, M.; Toyota, K.; Fukuda, R.; Hasegawa, J.; Ishida, M.; Nakajima, T.; Honda, Y.; Kitao, O.; Nakai, H.; Vreven, T.; Montgomery, J. A., Jr.; Peralta, J. E.; Ogliaro, F.; Bearpark, M.; Heyd, J. J.; Brothers, E.; Kudin, K. N.; Staroverov, V. N.; Kobayashi, R.; Normand, J.; Raghavachari, K.; Rendell, A.; Burant, J. C.; Iyengar, S. S.; Tomasi, J.; Cossi, M.; Rega, N.; Millam, N. J.; Klene, M.; Knox, J. E.; Cross, J. B.; Bakken, V.; Adamo, C.; Jaramillo, J.; Gomperts, R.; Stratmann, R. E.; Yazyev, O.; Austin, A. J.; Cammi, R.; Pomelli, C.; Ochterski, J. W.; Martin, R. L.; Morokuma, K.; Zakrzewski, V. G.; Voth, G. A.; Salvador, P.; Dannenberg, J. J.; Dapprich, S.; Daniels, A. D.; Farkas, Ö.; Foresman, J. B.; Ortiz, J. V.; Cioslowski, J.; Fox, D. J. Gaussian, Inc., Wallingford CT, 2009.

(12) (a) Becke, A. D. *J. Chem. Phys.* **1993**, *98*, 5648. (b) Lee, C.; Yang, W.; Parr, R. G. *Phys. Rev. B* **1988**, *37*, 785. (c) Becke, A. D. *J. Chem. Phys.* **1993**, *98*, 1372–1377. (d) Stephens, P. J.; Devlin, F. J.; Chabalowski, C. F.; Frisch, M. J. *J. Phys. Chem.* **1994**, *98*, 11623.

(13) Legault, C. Y. CYLView, 1.0b; Université de Sherbrooke, Canada, 2009; http://www.cylview.org.
